# Simplified one-pot ^18^F-labeling of biomolecules with *in situ* generated fluorothiophosphate synthons in high molar activity

**DOI:** 10.7150/thno.79452

**Published:** 2023-01-01

**Authors:** Hongzhang Yang, Lei Zhang, Huanhuan Liu, Yunming Zhang, Zhaobiao Mou, Xueyuan Chen, Jingchao Li, Fengming He, Zijing Li

**Affiliations:** 1 Center for Molecular Imaging and Translational Medicine, State Key Laboratory of Molecular Vaccinology and Molecular Diagnostics, Department of Laboratory Medicine, School of Public Heath, Xiamen University, Xiamen, Fujian 361102, China.; 2 Tianjin Engineering Technology Center of Chemical Wastewater Source Reduction and Recycling, School of Science, Tianjin Chengjian University, Tianjin 300384, China.; 3 School of Pharmaceutical Sciences, Xiamen University, Xiamen, Fujian 361102, China.

**Keywords:** radiolabeling, radiosynthon, fluorine-18, fluorothiophosphate, positron emission tomography probe

## Abstract

**Rationale:** Conventional ^18^F-labeling methods that demand substrate pre-modification or lengthy radiosynthesis procedures have impeded the visualization and translation of numerous biomolecules, as biomarkers or ligands, using modern positron emission tomography techniques *in vivo*. Moreover, ^18^F-labeled biomolecules in high molar activity (A_m_) that are indispensable for sensitive imaging could be only achieved under strict labeling conditions.

**Methods:** Herein, ^18^F-labeled fluorothiophosphate (FTP) synthons in high A_m_ have been generated rapidly *in situ* in reaction solutions with < 5% water *via* nucleophilic substitution by wet [^18^F]F^-^, which required minimal processing from cyclotron target water.

**Results:** Various ^18^F-labeled FTP synthons have been prepared in 30 sec at room temperature with high radiochemical yields > 75% (isolated, non-decay-corrected). FTP synthons with unsaturated hydrocarbon or activated ester group can conjugate with typical small molecules, peptides, proteins, and metallic nanoparticles. 337-517 GBq μmol^-1^ A_m_ has been achieved for ^18^F-labeled c(RGDyK) peptide using an automatic module with 37-74 GBq initial activity.

**Conclusion:** The combination of high ^18^F-fluorination efficiency of FTP synthons and following mild conjugation condition provides a universal simplified one-pot ^18^F-labeling method for broad unmodified biomolecular substrates.

## Introduction

Positron emission tomography (PET) is a non-invasive, real-time functional imaging technique that provides abundant physiological and biochemical information. It relies on the spatiotemporal tracing of molecular probes composed of functional scaffolds and positron-emitting nuclides 1, 2. Biomolecules, including small molecules, peptides and proteins, come from a treasury house of lead compounds with high specificity and low immunogenicity 3-5. ^18^F (*t*_1/2_ = 109.7 min, 97% β^+^, maximum positron energy 0.64 MeV) is the most popular positron-emitting nuclide due to its favorable chemical, nuclide properties and production feasibility 6. Numerous target-specific biomolecules merit ^18^F-labeling with optimum radiochemical parameters for pre-clinical and clinical PET imaging evaluation 7-10.

To achieve the mild ^18^F-labeling of biomolecules, two/multi-step/indirect approaches have been widely applied, which go through the incorporation of ^18^F into a prosthetic/linker synthon and then the gentle coupling of the synthon to the lead-compound biomolecules 4, 11-21 (**Table 1**). Since the hydration effect eliminates the reactivity/nucleophilicity of [^18^F]F^-^ in aqueous media directly obtained from a cyclotron, radiosynthons with ^18^F at one end and an active group at the other end usually need to be prepared in a dried organic medium with heating, followed by deprotection. Alternative methods are one-step/direct, mild ^18^F-labeling *via* ^18^F/^19^F-exchange or Al^18^F chelation in aqueous solutions at a specific pH/temperature on non-carbon-centered prostheses that are pre-coupled to biomolecules 10, 22-24 (**Table 1**). Nevertheless, universally accessible, highly efficient ^18^F-labeling methods that do not require the pre-coupling of a prosthetic group, time-consuming alterations of reaction conditions, purification of intermediates, and use of inseparable precursors for high molar activity (A_m_) are still in open request 25.

Due to the low heat of formation and high bond energy of P-F bonds, varied P(V) compounds exhibit a capacity for rapid mild ^18^F-labeling *via* ^18/19^F isotope exchange in both aprotic and protic solvents 24. To challenge the low A_m_ due to isotopic dilution, separable ^18^F-labeled fluorothiophosphates (FTPs) are synthesized *via* spontaneous [^18^F]F^-^ nucleophilic substitution and thiirane elimination on oxydithiaphospholane 2-sulfide precursors in this study. Preliminary density functional theory (DFT) calculation predicts a thermodynamically favorable fluorination pathway overcoming low activation but high hydrolysis energy barriers and at room temperature (RT), thereby forming the basis for rapid site-specific substitution by hydrated F^-^. The inclusion of a -SH group is supposed to improve stability through coulombic repulsion and attribute to the moderate lipophilicity that can prevent the non-specific binding of the ^18^F-labeled biomolecules. Comprehensive screening for condition/substrate scopes provides further insights regarding the key parameters affecting the ^18^F-labeling efficiency.

With rapidly *in situ* generated FTP synthons in high A_m_, a simplified one-pot ^18^F-labeling procedure for unmodified biomolecules is ready to be adopted. The oxydithiaphospholane 2-sulfide precursors can be efficiently ^18^F-labeled during eluting with aqueous [^18^F]F^-^ solution before mild conjugation with biomolecular substrates, skipping sophisticated automation. Typical peptide and protein biomolecules, such as c(RGDyK), human serum albumin (HSA) and nanobody 5F7, which are medically significant, are proof-of-concept lead compounds that exhibit solvent, temperature, or pH sensitivity. Selected ^18^F-labeled small molecular FTPs are also synthesized as phosphate tracers that exhibit a high level of similarity to the original phosphates, with regard to both stereo structures and biochemical interactions.

## Results

### Computational study

**Figure 1:**
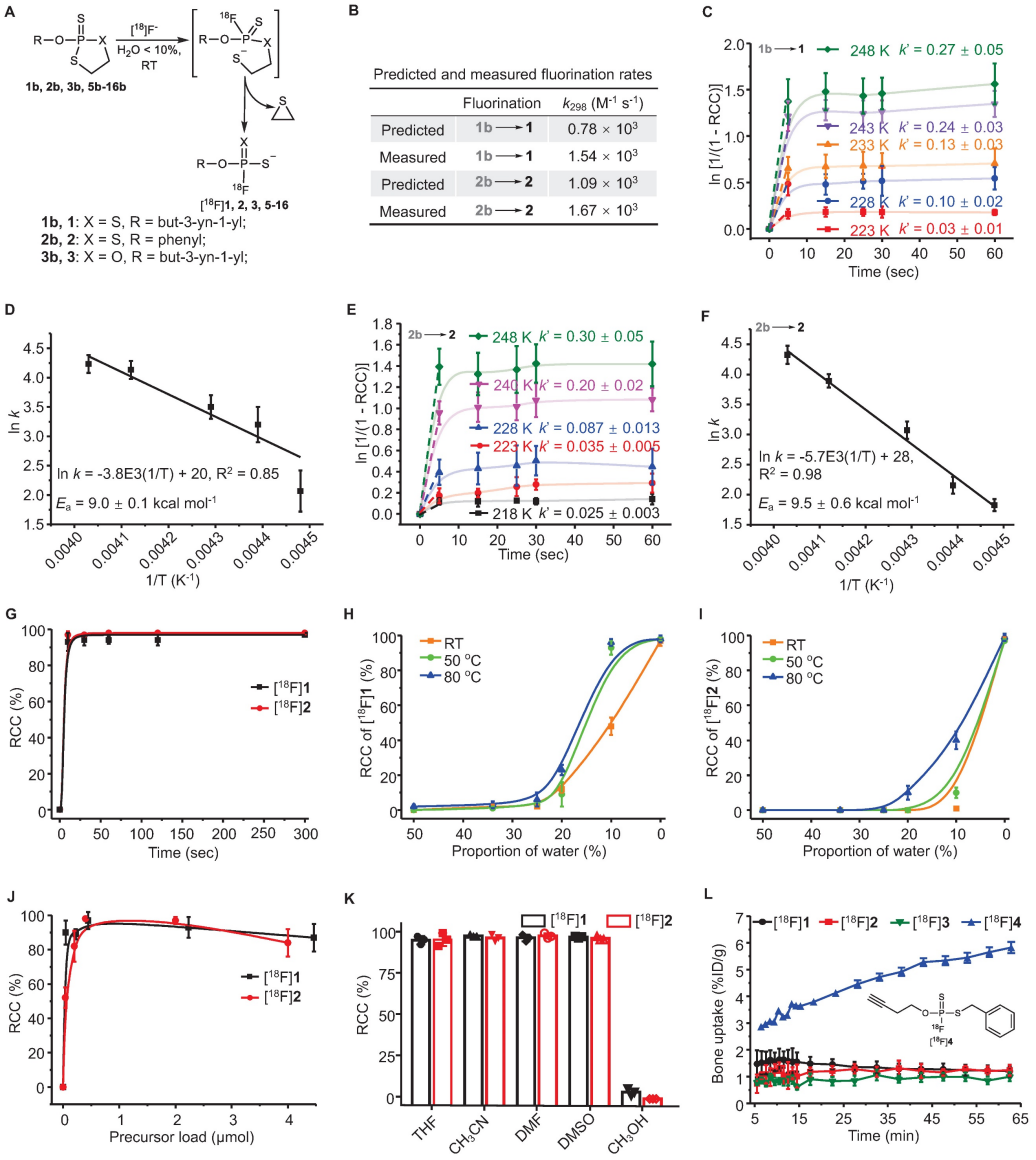
**Analysis of rapid kinetics and condition scopes of the ^18^F-labeling of FTPs.** (**A**) Reaction scheme showing the fluorination of FTPs. (**B**) Reaction rate constant (*k*) values that were predicted by density functional calculations at B3LYP/6-311+G* level of theory (at 298 K) and *k* values that were measured at 298 K. The pseudo-first order initial rate constant (*k*´) of [^18^F]**1** (**C**) and [^18^F]**2** (**E**) were determinated from the kinetic curves reflecting the change in ln [1/(1 - RCC)] in DMF at different temperatures. The *E*_a_ of [^18^F]**1** (**D**) and [^18^F]**2** (**F**) were determinated from Arrhenius plots of ln *k* against 1/T. (**G**) RCCs of [^18^F]**1** and [^18^F]**2** at different time periods from 10 to 300 sec in acetonitrile at RT. RCCs of [^18^F]**1** (**H**) and [^18^F]**2** (**I**) at different temperatures and water contents (The solvents are mixtures of acetonitrile and water, v/v. “0%” means no additional water is added.) post 30 sec. (**J**) RCCs of [^18^F]**1** and [^18^F]**2** with different precursor loads in acetonitrile post 30 sec at RT. (**K**) RCCs of [^18^F]**1** and [^18^F]**2** in different solvents post 30 sec at RT. (**L**) The time-bone uptake curves of [^18^F]**1**, [^18^F]**2**, [^18^F]**3**, and [^18^F]**4** during 0-60 min post-injection. Bone uptake was measured with microPET in healthy mice (n = 3).

DFT calculations were performed at the B3LYP/6-311+G* level in water. A three-step addition-elimination pathway was identified as a plausible reaction pathway 26. The rate-determining step of nucleophilic attacking by hydrated F^-^ was predicted to overcome the free-energy barriers of 16.5 and 17.4 kcal mol^-1^ at 298.15 K for substrates** 1b** and** 2b** (**Figure 1B**, **Figure S1**).

### Chemistry

To synthesize the aliphatic (**1**, **5**-**9**) and aromatic (**2**, **10**-**14**) FTPs with either electron-donating (**10**-**12**) or electron-withdrawing (**13**, **14**) substituents, 1,3,2-dithiaphospholanes (**1a**, **2a**, **5a**-**14a**) were first obtained through the phosphitylation of *N,N*-diethyl-1,3,2-dithiaphospholan-2-amine (**19**), in the presence of an alcohol/phenol and S-ethylthiotetrazole (**Figure 1A**, **Scheme S1**). Dithiaphospholanes were oxidized with elemental sulfur to obtain oxydithiaphospholane 2-sulfide substrates (**1b**, **2b**, **5b**-**13b**) at yields exceeding 50%. When these substrates were treated with an excess of tetrabutylammonium fluoride (TBAF) in tetrahydrofuran (THF), **1**, **2**, **5**-**13** were obtained at yields exceeding 95% in 2 min. In addition, **1b** and** 2b** were entirely consumed upon treatment with an equivalent amount of TBAF in THF in less than 2 min, as shown by ^31^P NMR spectroscopy results (**Figure S2**). Same routes were used to synthesize 2-(but-3-yn-1-yloxy)-1,3,2-oxathiaphospholane 2-sulfide (**3b**) and *O*-(but-3-yn-1-yl) phosphorfluoridothioate (**3**, a monothio-derivative). The oxidation of **14a** with elemental sulfur failed to obtain **14b**. FTPs with typical active groups, *N*-hydroxysuccinimide ester (NHS) for **15**, cyclooctene for **16**, were fluorinated from **15b** (**Scheme S3**) and **16b** (**Scheme S4**) at yields exceeding 95%. **17b** and **18b** were treated with TBAF, and then deprotected to obtain **17**-**18**, two bioactive phosphate analogs (**Scheme S5**, **S6**).

### Measurement of fluorination kinetics and energetics

Two simple substrates, *O*-(But-3-yn-1-yl) [^18^F]phosphorofluoridodithioate ([^18^F]**1**) and *O*-phenyl [^18^F]phosphorofluoridodithioate ([^18^F]**2**), were used as model compounds to evaluate fluorination kinetics and activation energies (*E*_a_). The pseudo-first order model is applied since the trace amount [^18^F]F^-^ is negligible compared to the precursors. The pseudo-first order initial rate constant (*k*´) under different temperatures were calculated from the exponential fit equation (**Figure 1C**,** 1E**) and then divided by the concentration of **1b** or** 2b** to determine the second-order rate constants (*k*). For the conversion of **1b** to** 1**, *k*_223_ = 8.53 L mol^-1^ s^-1^, *k*_228_ = 25.66 L mol^-1^ s^-1^,* k*_233_ = 34.93 L mol^-1^ s^-1^,* k*_243_ = 61.20 L mol^-1^ s^-1^,* k*_248_ = 69.38 L mol^-1^ s^-1^. For the conversion of **2b** to** 2**, *k*_218_ = 5.91 L mol^-1^ s^-1^, *k*_223_ = 8.60 L mol^-1^ s^-1^,* k*_228_ = 19.72 L mol^-1^ s^-1^,* k*_240_ = 47.85 L mol^-1^ s^-1^, and* k*_248_ = 66.20 L mol^-1^ s^-1^. The values of *E*_a_ were measured to be 9.0 ± 0.1 kcal mol^-1^ for the conversion of **1b** to** 1**, and 9.5 ± 0.6 kcal mol^-1^ for the conversion of **2b** to** 2** from Arrhenius plots (ln *k* versus T^-1^) (**Figure 1D**-**F**).

### Radiochemistry

^18^F-Labeling conditions were optimized in aprotic solvents containing graded proportions of water for different reaction durations (10-300 sec) under specific temperatures (RT-80 ℃). Non-decay-corrected radiochemical conversions (RCCs) were detected using both radio-TLC and radio-HPLC (F^-^ adsorption might occur to radio-HPLC column) at continuous time points (n = 3). The activity adsorption by the vial/glass was measured to be ∼10% of the total initial activity. RCC values were 98 ± 5% for [^18^F]**1** and 99 ± 4% for [^18^F]**2** after incubation for 30 sec at RT in anhydrous acetonitrile, with > 90% of RCCs being achieved in just 10 sec (**Figure 1G**). Although the RCC values decreased with the increasing of solvent water contents, satisfactory RCC values of 48 ± 5% for [^18^F]**1** and 10 ± 3% for [^18^F]**2** could be achieved in a mixture of acetonitrile and water (v/v = 9/1) at RT. RCC values of 98 ± 2% for [^18^F]**1** and 40 ± 5% for [^18^F]**2** was achieved using a mixture of acetonitrile and water (v/v = 9/1) at 80 ℃ (**Figure 1H**-**I**). Substrate **2b** showed higher sensitivity to water than **1b**, in consistence with the calculated and experimental fluorination kinetics and energetics. After assessing precursor loads of 0.04-4.5 μmol/100 μL, the optimal precursor load was determined to be 0.20-2.00 μmol/100 μL (**Figure 1J**). This ^18^F-labeling method exhibited high efficiency in aprotic solvents (**Figure 1K**).

### Stabilities of ^18^F-labeled FTPs and precursors

Each oxydithiaphospholane 2-sulfide substrate and representative FTP was incubated for 2 h in a mixture of acetonitrile and water (v/v = 1/9) with pH values of 1 to 13. The HPLC analysis results showed that the substrates could tolerate acids and weak bases but were unstable in strong alkaline solutions (**Figure S33**, **S34**). High stabilities of the FTP motif were observed in both acidic and alkaline solutions (pH values of 1 to 13, **Figure S35**, **S36**), which is critical for deprotection reaction in some occasion. The extent of defluorination (bone uptake, resistant to enzymatic hydrolysis *in vivo*) of [^18^F]**1** and [^18^F]**2** was insignificant in microPET imaging evaluation (n = 3) (**Figure 1L**, **S48**). While [^18^F]**1** was detected only in a small percentage of the parent compound in the urine, 5 min after administering an i.v. injection, [^18^F]**2** existed in the urine mainly in the form of the parent compound, as shown in **Figure S45**, **S46**. All procedures and animal use and care procedures have been approved by the Animal Care and Use Committee of Xiamen University.

A subsequent study showed that equally high stabilities were observed both *in vitro* and *in vivo*, if at least one O atom of phosphate was substituted by S atom, such as *O*-(but-3-yn-1-yl) [^18^F]phosphorofluoridothioate ([^18^F]**3**) and [^18^F]**1**. However, [^18^F]**3** was cleared rapidly from the kidney probably due to its higher hydrophilicity, which might lead to insufficient internalization (**Figure S47**, **S48**). Defluorination occurred slowly in *S*-benzyl *O*-(but-3-yn-1-yl) [^18^F]phosphorofluoridothioate ([^18^F]**4**) *in vitro* and* in vivo* (bone uptake reached ~6 %ID g^-1^ at 60 min), where the -SH moiety was substituted intentionally with an alkylthio group (**Figure 1L**, **S48**). This suggests that the coulombic force contributes to the high stability of FTPs. The biodistribution study confirmed the high *in vivo* stabilities of ^18^F-labeled FTPs, where [^18^F]**1**, [^18^F]**2**, [^18^F]**3**, and [^18^F]**15** ([^18^F]FTP-NHS, a typical *N*-hydroxysuccinimide ester synthon) all exhibited only background bone uptakes (0.9-1.1 %ID g^-1^) at 2 h post injection (**Figure S49**).

### Structure scope of ^18^F-labeled FTPs

**Figure 2:**
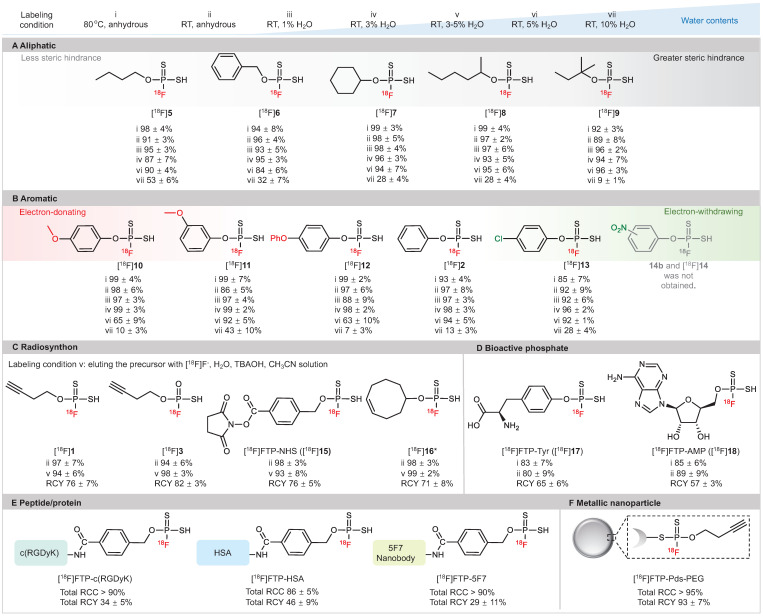
** Substrate structure scope of ^18^F-labeled FTPs.** Labeling condition i: 0.1 mg precursor, [^18^F]KF/K_222_ (1-5 mCi), 100 μL anhydrous acetonitrile, 80 °C, 30 sec. Labeling condition ii: 0.1 mg precursor, [^18^F]KF/K_222_ (1-5 mCi), 100 μL anhydrous acetonitrile, RT, 30 sec. Labeling condition iii: 0.1 mg precursor, [^18^F]F^-^ in cyclotron target water (1 μL, 0.3-0.5 mCi), 100 μL acetonitrile, RT, 30 sec. Labeling condition iv: 0.1 mg precursor, [^18^F]F^-^ in cyclotron target water (3 μL, 1-3 mCi), 100 μL acetonitrile, RT, 30 sec. Labeling condition v: 0.1 mg precursor, [^18^F]F^-^ in cyclotron target water (3 μL, 1-3 mCi), tetrabutylammonium hydroxide (0.52 mg), 100 μL acetonitrile, RT, 30 sec. Labeling condition vi: 0.1 mg precursor, [^18^F]F^-^ in cyclotron target water (5 μL, 1-5 mCi), 100 μL acetonitrile, RT, 30 sec. Labeling condition vii: 0.1 mg precursor, [^18^F]F^-^ in cyclotron target water (10 μL, 1-5 mCi) and acetonitrile 90 μL, RT, 30 sec. Each ^18^F-labeling reaction was performed thrice with all reported RCCs determined by radio-HPLC or radio-TLC. RCCs mean ± standard deviation values. (**A**) Aliphatic substrates with different levels of steric hindrance. (**B**) Aromatic substrates with different electron density distributions. (**C**) ^18^F-Labeled FTPs as radiosynthons. (**D**) Small molecular FTPs as phosphate analogs. (**E**) ^18^F-Labeled peptide/protein biomolecules generated using FTP radiosynthons. (**F**) ^18^F-Labeled metallic nanoparticle generated using an FTP radiosynthon. * Cis-cyclooctene was used as a substitute.

As illustrated in **Figure 2**, high RCC values of 88-98% were observed in anhydrous acetronitrile with a wide substrate scope of ^18^F-labeled FTPs, including alkanes, alkynes, heterocycles, halides, amino acid derivatives and nucleotide derivatives (condition i, ii). Notably, despite the absence of a phase transfer reagent, RCCs of 87-99% were observed with the use of wet [^18^F]F^-^ in solvents with 1-3% water (condition iii, iv). The RCCs decreased gradually with an increase in the water content; when the water content was 5% (condition vi) and 10% (condition vii), RCCs were 63-96% and 7-53%, respectively. ^18^F-Labeled FTPs with functional groups, such as [^18^F]**1**, [^18^F]**3**, [^18^F]**15** and [^18^F]**16**, could be coupled to biomolecules and acted as ready-to-use radiosynthons. High RCC values > 95% and high radiochemical yield (RCY, isolated yields, non-decay-corrected, n = 3) values > 75% were observed for these FTP synthons that were *in situ* generated with wet [^18^F]F^-^ at RT (condition v, in solvents with 3-5% water). The A_m_ of [^18^F]**15** ([^18^F]FTP-NHS) reached 128.2 GBq μmol^-1^ by manual labeling with 3.7-5.6 GBq initail activity. Biologically active phosphate analogs, such as the ^18^F-labeled L-Tyr phosphate (p-Tyr) mimic ([^18^F]FTP-Tyr, [^18^F]**17**) and the ^18^F-labeled adenosine monophosphate mimic ([^18^F]FTP-AMP, [^18^F]**18**), were labeled directly with RCYs > 55%.

### [^18^F]FTP-c(RGDyK)

**Figure 3:**
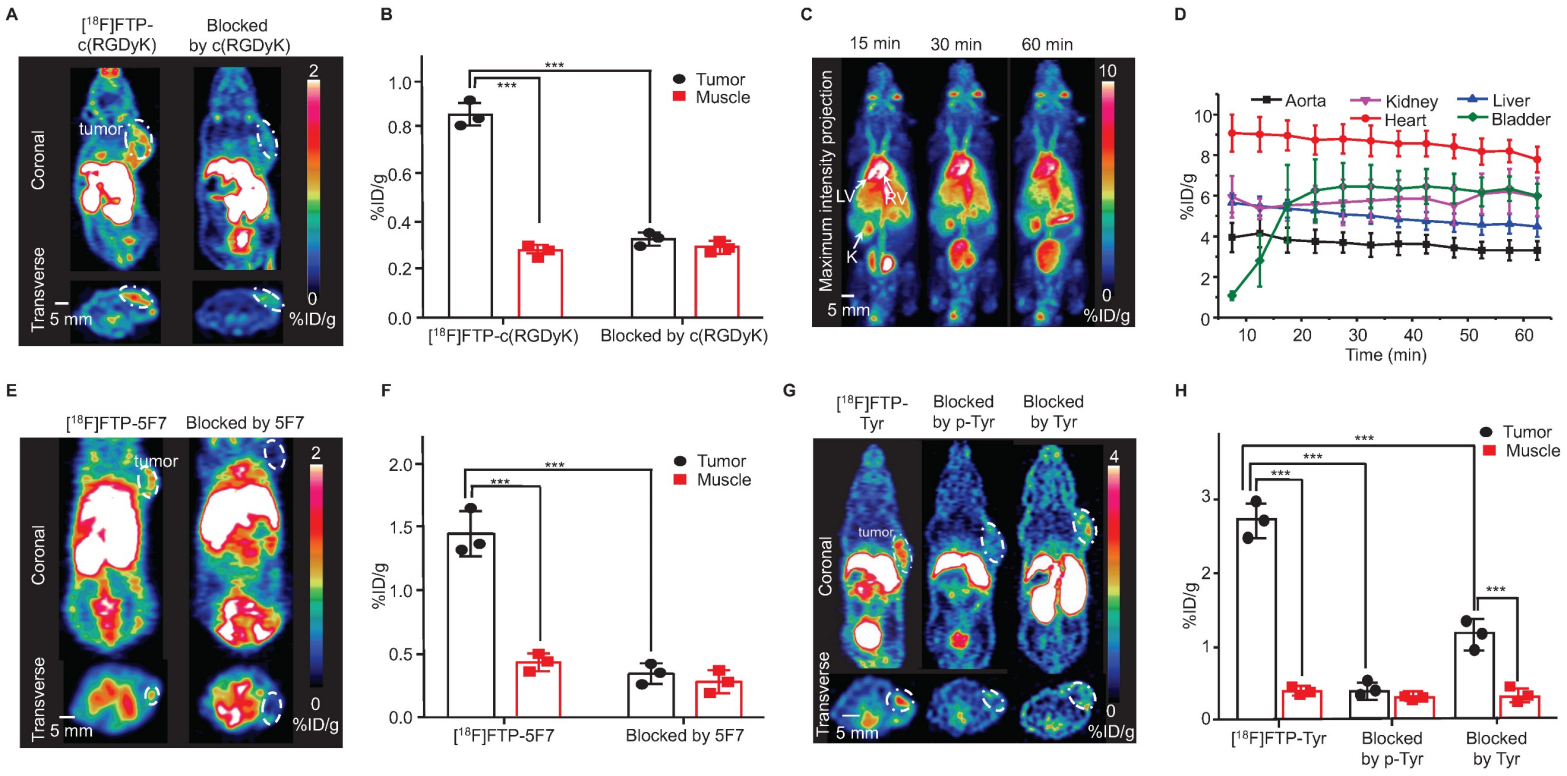
** Selected^ 18^F-labeled biomolecules *via* FTPs and their microPET imaging.** (**A**) MicroPET images of [^18^F]FTP-c(RGDyK) showing specific accumulation in 4T1 xenografts at 15 min post-injection (The white dotted line indicates the tumor margin). (**B**) Quantitative PET imaging results. Data represent the mean ± standard deviation values (n = 3), ***P < 0.001. (**C**) Dynamic microPET images of healthy female mice reconstructed at 15, 30, and 60 min after administering an [^18^F]FTP-HSA injection. LV: left ventricle; RV: right ventricle; K: kidneys. (**D**) Time-activity curves of [^18^F]FTP-HSA in indicated organs. Uptake values are presented in terms of mean ± standard deviation values (n = 3). (**E**) MicroPET images of [^18^F]FTP-5F7 showing specific accumulation in MDA-MB-453 xenografts at 60 min post-injection (The white dotted line indicates the tumor margin). (**F**) Quantitative PET imaging results. Data represent the mean ± standard deviation values (n = 3), ***P < 0.001. (**G**) MicroPET images showing the specific accumulation of [^18^F]FTP-Tyr in B16 xenografts at 15 min post-injection (the white dotted line indicates the tumor margin). (**H**) Quantitative PET imaging results. Data represent the mean ± standard deviation values (n = 3), ***P < 0.001.

The Arg-Gly-Asp (RGD) sequence exhibits high affinity and selectivity for alpha(v)beta3 integrin, which is a significant peptide-based ligand 27. Under mild coupling conditions, i.e., 10 min, 37 ℃, borate buffer (pH = 8.0), [^18^F]FTP-NHS was efficiently conjugated with c(RGDyK) to obtain [^18^F]FTP-c(RGDyK). The total RCC values were ~90% determined by radio-HPLC, and the average RCY value was 34 ± 5% and A_m_ value was 107 GBq μmol^-1^ after manually labeling with 5.6 GBq initial activity (**Figure S25**,** S31**). The purification of [^18^F]FTP-c(RGDyK) using a C18 cartridge followed by HPLC resulted in > 98% radiochemical purity (RCP) (**Figure S20**). An automated procedure involving a commercial multifunctional radiosynthesis module was established (**Figure S24**). The total auto-synthesis time was ~60 min, and the RCY was 39 ± 8%, with an A_m_ value of 337-517 GBq μmol^-1^ (initial activity 37-74 GBq). The microPET/CT imaging data indicated that [^18^F]FTP-c(RGDyK) was accumulated specifically within 4T1 xenografts. The average tumor uptake of this monomeric RGD tracer, based on the whole tumor region of interest (ROI), was 1.30 ± 0.28 %ID g^-1^, with a tumor-to-muscle ratio of 2.38 at 15 min post-injection (**Figure 3A**-**B**), in comparison to a value of roughly 1.8 %ID g^-1^ for [^18^F]AMBF_3_ labeled trimeric RGD 19.

### [^18^F]FTP-HSA

HSA, a heat-sensitive protein, has routinely been radiolabeled and used to evaluate plasma distribution volumes in specific organs, quantify cardiac function-related parameters, and assess vascular “leakage” in pathological tissues 28. [^18^F]FTP-HSA was obtained *via* [^18^F]FTP-NHS after 10 min conjugation in borate buffer (pH = 8.0) at 40 ℃, with an overall RCC of 83 ± 5% (n = 3). The average RCY value was 46 ± 9% (RCP > 99%) after purification by a PD-10 column (GE Healthcare Bio-Science AB) (**Figure S21**). As shown by the dynamic microPET/CT images in **Figure 3C**, the central vasculature, including the cardiac ventricular chambers, were clearly visualized. There was no apparent radioactivity in the skeleton, which suggested the absence of significant amounts of free [^18^F]F^-^ and other metabolites (**Figure 3D**).

### [^18^F]FTP-5F7

Nanobodies (VHH, 12-15 kDa), the antigen-binding fragments of heavy-chain-only antibodies derived from *Camelidae*, have biological half-lives of 1-2 h that are comparative with ^18^F 29, 30. Herein, we explored the feasibility of utilizing the ^18^F-labeled 5F7 anti-HER2 nanobody as a probe for evaluating the HER2 expression. [^18^F]FTP-5F7 was obtained *via* [^18^F]FTP-NHS after 10 min conjugation in borate buffer (pH = 8.0) at RT. Overall RCCs values were determined *via* radio-HPLC to be ~90%, and the average RCY value was 29 ± 11% (RCP > 95%) after purification by a size exclusion chromatography column (**Figure S22**). MicroPET/CT images of mice with HER2-overexpressing MDA-MB-453 xenografts demonstrated the rapid tumor accumulation (1.40 ± 0.22%) and clearance of [^18^F]FTP-5F7 from the background. Pre-treatment with excessive amounts of 5F7 substantially reduced the tumor uptake in the background (**Figure 3E**-**F**).

### [^18^F]FTP-Pds-PEG

The use of radiolabeled nanoparticles for molecular imaging has attracted broad attention due to their large functional surface area and easy-to-control surface chemistry, which allows them to be tailored for the purpose of personalized cancer management 31. The sulfhydryl groups on FTPs can form stable coordinate covalent bonds, which can be used to attach various metal nanoparticles efficiently. PEGylated Pd nanosheets (Pd-PEGs) with an average size of 40 nm were conjugated with [^18^F]**1** upon stirring the reaction mixture for 15 min at RT. The overall RCYs of [^18^F]FTP-Pds-PEG were > 90% after ultrafiltration (same as measured by radio-TLC, RCP > 99%, **Figure S23**). 2 h after administering an i.v. injection, a significant tumor uptake of 4.87 ± 0.31 %ID g^-1^ based on the whole tumor ROI was illustrated by microPET/CT imaging, as well as a high tumor-to-muscle ratio of 3.4 in subcutaneous 4T1 tumors (**Figure S56**).

### [^18^F]FTP-Tyr

Evidence has shown that greater uptake rate, solubility, and oxidative stability were observed during the uptake of L-DOPA phosphate and p-Tyr by melanomas than those observed with L-DOPA and L-Tyr 32, 33. Thus, they were recruited as melanogenesis markers for the early diagnosis of melanomas. In this study, [^18^F]FTP-Tyr was synthesized as a non-hydrolytic (overcoming the *in vivo* enzymatic-instabilities of phosphates) p-Tyr analog with an RCC of 80 ± 9% and A_m_ of > 3.91 GBq μmol^-1^ (manual labeling; initial activity 1.48-1.85 GBq; FTP-Tyr exhibited weak UV absorption to determine the A_m_; **Figure S29**). The results of the *in vitro* cell uptake study revealed that the significant specific uptake of [^18^F]FTP-Tyr by a B16 tumor within 120 min, with maximum uptake occurring at 30 min. This uptake could be either entirely inhibited by p-Tyr or partially inhibited by L-Tyr (**Figure S53**). More evidence for the similarity between FTP-Tyr and p-Tyr was provided in the supplementary material (**Figure S50**-**S52**, **S54**, **S55**). MicroPET/CT imaging of [^18^F]FTP-Tyr was performed in B16 xenograft mice, and specific accumulation in tumors and a tumor-to-muscle ratio of 6.4 were observed at 30 min after administering an i.v. injection. This specific accumulation was blocked by p-Tyr and only partially blocked by Tyr as well, indicating the specific uptake of [^18^F]FTP-Tyr by melanomas (**Figure 3G**-**H**).

## Discussion

The easily oxidized trivalent phosphorus intermediates (**19**, **20**, **1a**-**3a, 5a**-**18a**, monitored by ^31^P NMR) were not isolated and directly treated with sulfur to obtain the separable pentavalent precursors liable to oxidation (**1b**-**3b**, **5b**-**13b**, **15b**-**18b**) with total yields ranged from 10-70%. Functional groups exhibiting higher steric repulsion and stronger electron withdrawal are unfavorable for precursor synthesis, e.g., the unformed **14b** and **14**. Although the predicted three-step pathway mechanism requires further experimental evidence, the extremely rapid fluorination kinetics were in accordance with the experimental rate constants and pseudo-first order analysis. Ultimately, the high ^18^F-fluorination efficiency at RT was attributed to the low free-energy barriers and trace amount [^18^F]F^-^. Favorable energy barriers also resulted in a higher selectivity for F^-^ over other nucleophiles, such as, OH^-^, which is many-fold more in quantity in wet solvents. Thus, the routine azeotropic drying processing of [^18^F]F^-^ can be simplified to evaporating processing to obtain reaction solutions that contain less than 5% water in automatic production.

Non-structure-biased ^18^F-labeled FTPs were generated at high RCCs within seconds in solvents with a water content of < 5%, using a very low molar quantity/concentration of storage-stable precursors (0.1 mg/0.22-0.45 μmol per 100 μL). Varied ^18^F-labeled FTP synthons with respective functional/linker groups, e.g., unsaturated hydrocarbon or activated ester group, are accessible. Insignificant levels of defluorination but multiple degradation products were observed *in vivo* for [^18^F]**1** and [^18^F]**2**. The *in vivo* metabolic stabilities of ^18^F-labeled FTPs are attributed not only to the coulombic repulsion between the FTP group and anions, which protects the P-F bond from hydrolysis, but also the substitution of P=O with P=S, which enhances its resistance to enzymatic hydrolysis (**Figure 1L**, **S48**, **S51**).

A simplified ^18^F-labeling strategy has been consequently developed *via* the rapid *in situ* generation of instant FTP synthons that could couple with delicate biomolecules. This strategy does not require the pre-modification of biomolecules, preparation of dry [^18^F]F^-^, exposure of biomolecules to harsh conditions, and purification of radioactive intermediates. The negligible time-related costs associated with FTP synthon formation and application of the same mild conditions used for the conjugation step during multi-step labeling results in the high ^18^F-labeling efficiency. Typical medically significant biomolecules, such as c(RGDyK), HSA, and nanobody 5F7, which exhibit solvent/temperature/pH sensitivity, have been successfully labeled *via* FTP synthons either manually or automatically.

In contrast to the existing direct ^18^F-labeling methods that are mostly based on ^18^F/^19^F-isotope-exchange, due to the significant polarity difference between the more hydrophilic FTPs and the less hydrophilic precursors, they can be separated feasibly using chromatography, thus higher A_m_s values can be achieved for receptor imaging. Compared to the satisfactory A_m_ obtained *via* ^18^F/^19^F-exchange with high initial activity, ^18^F-labeled FTPs have been generated *via* nucleophilic substitution to give > 300 GBq mol^-1^ A_m_ with 37-74 GBq initial activity^18-20^. The ratio of A_m_ to initial activity (A_m_ achieved with certain amount of initial activity of ^18^F) under a certain molar amount of the precursor, instead of A_m_, was used an important parameter to reflect the ^18^F-fluorination capability of the labeling methods with certain chemical motif or precursor (**Table 1**). Enhanced hydrophilicity may also lead to an improved pharmacokinetic profile of the radiolabeled peptides by shifting hepatobiliary to renal clearance.

## Conclusion

Mild and efficient nucleophilic fluorination with [^18^F]F^-^ and separable precursors that allows site-specific ^18^F-labeling in high A_m_ are long pursued for highly sensitive PET imaging. Water-tolerant [^18^F]F^-^ nucleophilic fluorination, which was previously accessible mainly with isotope-exchange reactions, would reduce the failure risk of costly ^18^F-labeling in contrast to the ^18^F-labeling methods demanding strictly anhydrous condition. Taking this opportunity, this study describes a rapid, [^18^F]F^-^ nucleophilic radiolabeling method *via in situ* generated FTPs in non-anhydrous solvent, and the introduction of high-A_m_ FTPs as radiosynthons or tracers to facilitate the development of PET tracers from unmodified biomolecules and phosphate-based biomarkers. This sufficiently simple radiofluorination method in partially aqueous media using disposable and cheap labware may enable us to overcome the drawbacks associated with both indirect and direct ^18^F-labeling, and facilitate the development of a broadly applicable kit-like protocols for ^18^F-labeling of biomolecules.

## Experimental Section

### Materials

All the reagents we used in the synthesis and biology experiment were purchased from Energy Chemical Co., Ltd. (China) or J&K Co., Ltd. (China) and were used without further purification. Column chromatography purification was performed on silica gel (54-74 μm, Qingdao Haiyang Chemical Co., Ltd., China). Anhydrous dichloromethane, anhydrous tetrahydrofuran (THF), anhydrous dimethyl sulfoxide (DMSO), anhydrous acetonitrile and anhydrous dimethylformamide (DMF) were purchased from Energy Chemical Co., Ltd. (China) and used without further drying.

### Fluorination kinetics

In order to determine the fluorination kinetics and *E*_a_ for ^18^F-fluorination process, we decided to carry out a series of experiments at different temperatures. We found experimentally that the optimal temperature range to monitor the labeling reaction rate of [^18^F]**1** and [^18^F]**2** is between -55 °C and -25 °C, since the reaction is too fast at higher temperatures and too slow at lower temperatures to measure. Concentrations of precursors and in DMF solutions were kept constant at 3.82 × 10^-3^ M and 4.01 × 10^-3^ M, respectively. The RCCs under each individual temperature was monitored by radio-TLC. Dynamic RCCs were able to be monitored by TLC because we took 10 μL of the reaction mixture at the indicated time points, quenched it in 1.0 mL of water before TLC developing. Labeling efficiency graphs could be converted to line graphs of reaction time versus ln [1/(1-RCC)], whose slopes represent specific rate constants at specific temperatures. Pseudo-first order initial rate constants at different temperatures were calculated from the exponential fit equation, and then *k*' was divided by the concentration of **1b** or **2b** to determine the actual second-order rate constants. These rate constants were used to create an Arrhenius plot (ln *k* versus T^-1^) to calculate the *E*_a_, which was found to be 8.41 kcal mol^-1^ for ^18^F-labeling reaction of [^18^F]**1** and 8.98 kcal mol^-1^ for ^18^F-labeling reaction of [^18^F]**2**.

### General manual ^18^F-labeling procedures

**Labeling procedure I**: [^18^F]F^-^ was azeotropically dried as previously described 14. Briefly, [^18^F]F^-^ was produced *via* the ^18^O(p, n)^18^F reaction and delivered as [^18^F]F^-^ in [^18^O]H_2_O. [^18^F]F^-^ (0.185-1.85 GBq) was separated from ^18^O-enriched-water using QMA and subsequently released with by a solution of 8.0 mg kryptofix 222 (K_222_) and 1.0 mg K_2_CO_3_ in 1.0 mL of acetonitrile/H_2_O (4/1, v/v). The solution was azeotropically dried for three times (300 μL acetonitrile × 3) at 100 ℃ in a clean glass vial. 0.2 μmol precursor was dissolved in 100 μL acetonitrile (anhydrous or non-anhydrous) and added into the glass vial containing [^18^F]F^-^. The mixture was incubated at RT for 30 sec before quenching by 500 μL water. RCCs were analyzed by radio-HPLC or radio-TLC (n = 3). **Labeling procedure II** (with wet [^18^F]F^-^): 0.2 μmol precursor was dissolved in 50 μL acetonitrile. Aqueous [^18^F]F^-^ solution (diluted from target water, no K_222_ and K_2_CO_3_ added, 0.185-1.85 GBq) in appropriate volume was added into the solution. Then total reaction volume was adjusted to 100 μL with pure water and acetonitrile. The reaction vial was gently shaken at RT for 30 sec before quenching by adding 500 μL water. RCCs were analyzed by radio-HPLC or radio-TLC (n = 3).

### Radiosynthesis of [^18^F]FTP-Tyr

[^18^F]**17c** was synthesized from **17b** following **labeling procedure I**. [^18^F]**17c** was then dissolved in 100 μL MeOH followed by adding 100 μL NaOH (2.0 M) for 15 min. The reaction mixture was acidified by 1.0 M HCl and was dried under a stream of nitrogen. The resulting residue was dissolved in acetonitrile (100 μL). 5.0 M HCl or TFA (100 μL) was added and shaken at RT for 10 min. The solution was then neutralized to pH 7 by adding NaOH (1.0 M) and diluted with PBS and acetonitrile before being analyzed and purified on HPLC. The collected product was then put on the rotary evaporator to remove excess methanol from the solution. [^18^F]FTP-Tyr was dissolved in saline for PET imaging studies.

### Radiosynthesis of [^18^F]FTP-AMP

[^18^F]**18c** was synthesized from **18b** following **labeling procedure I**. Then 100 μL TFA was added to [^18^F]**18c** and stirred at RT for 5 min to deprotection. The solution was then neutralized to pH 7 by adding NaOH (1.0 M) and diluted with PBS and acetonitrile before being analyzed and purified on radio-HPLC. The collected product was then put on the rotary evaporator to remove excess acetonitrile from the solution. [^18^F]FTP-AMP was dissolved in saline for PET imaging studies.

### Radiosynthesis of [^18^F]FTP-NHS

0.1 mg precursor **15b** (0.2 μmol) was dissolved in dichloromethane (40 μL) and loaded onto a cotton ball (about 0.03 cm^3^). Then the dichloromethane was allowed to volatilize in a fume hood, and the small cotton carrying precursor **15b** was fitted into a pipette tip (for manual labeling, any tube-like part for automatic modules. [^18^F]**15** was synthesized from **18b** following **labeling procedure II**. No-carrier-added [^18^F]F^-^ was produced *via* the ^18^O(p, n)^18^F reaction and delivered as [^18^F]F^-^ in [^18^O]H_2_O. [^18^F]F^-^ (3 μL, 0.30-0.37 GBq) was added to 100 μL acetonitrile with tetrabutylammonium hydroxide (TBAOH, 0.52 mg/100 μL), which was used as the eluent later. The pipette tip carrying precursor **15b** was eluted by this mixed solution into a polypropylene tube and subsequently dried under a stream of nitrogen to afford [^18^F]**15** as a radiosynthon.

### Radiosynthesis of [^18^F]FTP-c(RGDyK)

1.0 mg (1.6 μmol) c(RGDyK) was dissolved in a mixture of 10 μL DMSO and 100 μL of borate buffer (pH = 8.0). This c(RGDyK) solution was then added into the vial containing [^18^F]**15**. After 10 min reaction at 37 ℃, the reaction mixture was diluted with 10.0 mL of H_2_O and loaded onto a light C18 cartridge. The cartridge was flushed twice with 10.0 mL of pure water to remove the unreacted [^18^F]F^-^ and 1.0 mL of ethanol to get crude product. The crude product was further purified by radio-HPLC. Purify condition: Waters XBridgeC-18 column (5 µm, 10 mm × 250 mm, USA). Phase A: PBS (0.02 mol L^-1^ pH = 7.4); phase B: HPLC grade acetonitrile; isocratic elution at 90% phase A and 10% phase B. Flow rate: 3.0 mL min^-1^. The HPLC fraction was dried under a stream of nitrogen at RT. [^18^F]FTP-c(RGDyK) was then dissolved in saline for injection.

### Radiosynthesis of [^18^F]FTP-HSA

1.0 mg of HSA in 0.1 M borate buffer (pH = 8.0, 2 mg mL^-1^, 100 μL) was added to a glass vial containing dried [^18^F]**15** and the mixture was incubated for 10 min at 40 ℃. [^18^F]FTP-HSA was purified by a PD-10 column (GE Healthcare Bio-Science AB) using PBS (pH = 7.4) as the eluent. A size exclusion chromatography (SEC) column was applied to analyze its RCP.

### Radiosynthesis of [^18^F]FTP-5F7

0.1 mg of 5F7 was dissolved in 100 μL of borate buffer (pH = 8.0) and added to [^18^F]**15**. The reaction mixture was incubated for 10 min at RT. The product was purified and then analyzed by radio-HPLC equipped with an Xtimate SEC-300 column (Welch, China).

### Radiosynthesis of [^18^F]FTP-Pds-PEG

^18^F-Labeled FTPs were prepared following the **Labeling procedure I**. Pd nanosheets with an average size of 40 nm (200 μg), as an example of metal nanoparticle, dissolved in thiol-polyethylene glycol (mPEG-SH) solution (2 mg in 100 μL water) to obtain PEGylated Pd nanosheets. Then, [^18^F]**1** (taking [^18^F]**1** as an example) was added to Pd-PEG and stirred for 15 min at RT to get [^18^F]FTP-Pds-PEG. RCCs was determined by radio-TLC analysis. [^18^F]**1** labeled Pd nanosheets with a high efficiency (about 93%). Then the mixture was subject to ultrafiltration (13000 rpm for 10 min, repeated 3 times) to remove unlabeled [^18^F]**1** and [^18^F]F^-^. RCPs was determined by radio-TLC analysis.

## Supplementary Material

Supplementary materials and methods, figures, tables.

## Figures and Tables

**Table 1:** Important parameters of selected ^18^F-labeling methods for biomolecules.

Tracer	Labeling strategy	Mechanism	T (°C)	Solvent (v/v)	pH	Total synthesis time (min)	Precursor load (μmol)	RCC & RCY	A_m_ (GBq μmol^-1^)/Starting activity (GBq)	Reference
[^18^F]FTP-c(RGDyK)	Instant FTP	S_N_^[a]^	RT	Borate buffer/DMSO (91/9)	8.0	15^[c]^ 60^[d]^	0.2	> 90%^[f]^ & 34 ± 5%^[g]^	49-107/3.7-5.6^[c]^ 337-517/37-74^[d]^	-
[^18^F]FTP-HSA	Instant FTP	S_N_	RT	Borate buffer	8.0	15^[c]^	0.2	86 ± 5%^[f]^ & 46 ± 9%^[g]^	26.37-39.14/0.185-1.85^[c]^	-
[^18^F]FTP-5F7	Instant FTP	S_N_	RT	Borate buffer	8.0	15^[c]^	0.2	> 90%^[f]^ & 29 ± 11%^[g]^	-	-
[^18^F]SFB-RGD	Multi-step	S_N_	RT	Borate buffer/DMSO	7.4	130^[d]^	13	13 ± 3%^[g]^	14.06 ± 4.07^[d]^/-^[h]^	(14)
[^18^F]FBOM-GSH	Multi-step	S_N_	RT	PBS	7.4	> 60^[d]^	30	22-30%^[f]^	-/-	(17)
(±)-[^18^F]AlFRESCA-HSA	One-step	Chelate reaction	RT	Ammonium acetate buffer	4	< 35^[c]^	5.64	35-53%^[f]^	79.92-85.10/1.258 ^[c]^	(10)
[^18^F]SiFA-peptide	One-step	IEX^[b]^	RT	H_2_O/CH_3_CN (10/90)	4	25^[c]^	0.07	55-65%^[f]^	2.96-5.18/0.178-0.248 ^[c]^	(22)
[^18^F]AMBF_3_-TrisRGD	One-step	IEX	80	Pyridazine-HCl buffer	2.5	12^[e]^	0.05-0.08	23 ± 5%^[g]^	111-148/29.6-37^[d]^	(23)
[^18^F]DBPOF-c(RGDyK)	One-step	IEX	RT	H_2_O/DMSO (95/5)	7	25^[c]^	1-3	15-25%^[f]^	2.22-4.81/0.821-1.64^[d]^	(24)

^[a]^ Nucleophilic substitution. ^[b]^ Isotope exchange. ^[c]^ The probe was manually synthesized. ^[d]^ The probe was synthesized by automated radiosynthesis module. ^[e]^ The probe was synthesized manually, 12 min was reported for the ^18^F-labeling step alone. ^[f]^ RCCs were detected by radio-HPLC. ^[g]^ RCYs were isolated yields. ^[h]^ Not found.

## Abbreviations

*E*_a_): Activation energy
RGD): Arg-Gly-Asp
DFT): density functional theory
DMF): dimethylformamide
DMSO): dimethyl sulfoxide
FTP): fluorothiophosphate
HSA): human serum albumin
NHS): *N*-hydroxysuccinimide ester
K_222_): kryptofix 222
A_m_): molar activity
Pd-PEGs): PEGylated Pd nanosheets
PET): positron emission tomography
*k*´): pseudo-first order initial rate constant
RCC): radiochemical conversion
RCP): radiochemical purity
RT): room temperature
RCY): radiochemical yield
*k*): second-order rate constant
SEC): size exclusion chromatography
TBAF): tetrabutylammonium fluoride
TBAOH): tetrabutylammonium hydroxide
THF): tetrahydrofuran
