# Supplementary material for: Simplified one-pot 18F-labeling of biomolecules with in situ generated fluorothiophosphate synthons in high molar activity
Source: Theranostics. 2023 Jan 1;13(2):472–82. doi: 10.7150/thno.79452 (PMC9830440; doi:10.7150/thno.79452)
Supplement: Supplementary file 1 — Supplementary materials and methods, figures, tables. [file thnov13p0472s1.pdf]

Supplementary material

Simplified One-pot ^{18}F-Labeling of Biomolecules with *in situ* Generated Fluorothiophosphate Synthons in High Molar Activity

Hongzhang Yang,[1] Lei Zhang,[2] Huanhuan Liu,[1] Yunming Zhang,[1] Zhaobiao Mou,[1] Xueyuan Chen,[1] Jingchao Li,[1] Fengming He,[3] and Zijing Li,[1]*

[1]Center for Molecular Imaging and Translational Medicine, State Key Laboratory of Molecular Vaccinology and Molecular Diagnostics, School of Public Health, Xiamen University, Xiamen, Fujian 361102, China.

[2]Tianjin Engineering Technology Center of Chemical Wastewater Source Reduction and Recycling, School of Science, Tianjin Chengjian University, Tianjin 300384, China.

[3]School of Pharmaceutical Sciences, Xiamen University, Xiamen, Fujian 361102, China.

***Corresponding author**
Zijing Li Ph.D., zijing.li@xmu.edu.cn

This PDF file includes:

Supplementary materials and methods

Schemes S1 to **S12**

Tables S1 to **S7**

Figures S1 to **S70**

Supplementary references

Appendix

Figures A1 to **A134** (Spectral data for the newly synthesized compounds)

Table of Contents

Supplementary materials and methods S5

1 General information S5

1.1 General reagent information S5

1.2 General chemical analysis information S5

1.3 General radiochemistry information S6

2 Synthesis S7

2.1 1, 2, 5–14 .... S7

2.2 *O*-(But-3-yn-1-yl) phosphorofluoridothioate (3) S19

2.3 *O*-(4-(((2,5-Dioxopyrrolidin-1-yl)oxy)carbonyl)benzyl) phosphorofluoridodithioate (15) S20

2.4 *O*-(Cyclooct-4-en-1-yl) phosphorofluoridodithioate (16) S23

2.5 (*S*)-*O*-(4-(2-Amino-2-carboxyethyl)phenyl) phosphorofluoridodithioate (FTP-Tyr, 17) S24

2.6 *O*-(((2*R*,3*S*,4*R*,5*R*)-5-(6-Amino-9H-purin-9-yl)-3,4-dihydroxytetrahydrofuran-2-yl)methyl) phosphorofluoridodithioate (FTP-AMP, 18) S25

3 Reaction pathways and free-energy profiles S29

4 Fluorination kinetics S32

4.1 Predicted fluorination kinetics by DFT S32

4.2 Fast fluorination measured by ^{31}P NMR S32

4.3 Experimental fluorination rates S33

5 Radiosynthesis of ^{18}F-labeled FTPs S35

5.1 General manual ^{18}F-labeling procedures S35

5.2 Optimization of ^{18}F-labeling conditions S35

5.2.1 Effect of labeling time S35

5.2.2 Effect of precursor loads S36

5.2.3 Effect of temperatures and solvent water contents S37

5.2.4 Effect of solvents S38

5.3 Identification of [^{18}F]1, [^{18}F]2, [^{18}F]5-[^{18}F]13, [^{18}F]16 .... S39

5.4 Radiosynthesis of ^{18}F-labeled *O*-(but-3-yn-1-yl) phosphorofluoridothioate ([^{18}F]3) as a radiosynthon S45

5.5 Radiosynthesis of *O*-(but-3-yn-1-yl) phosphorofluoridodithioate ([^{18}F]4) S46

5.6 Radiosynthesis of ^{18}F-labeled FTPs as PET tracers S47

5.6.1 Radiosynthesis of [^{18}F]FTP-Tyr S47

5.6.2 Radiosynthesis of [^{18}F]FTP-AMP S49

5.7 Radiosynthesis of ^{18}F-labeled biomolecules *via* ^{18}F-labeled FTP synthons S50

5.7.1 Radiosynthesis of [^{18}F]FTP-NHS ([^{18}F]15) S50

5.7.2 Radiosynthesis of [^{18}F]FTP-c(RGDyK) S51

5.7.3 Radiosynthesis of [^{18}F]FTP-HSA S52

5.7.4 Radiosynthesis of [^{18}F]FTP-5F7 .... S53

5.7.5 Radiosynthesis of [^{18}F]FTP-Pds-PEG S53

6 Automated radiosynthesis and quality control of [^{18}F]FTP-c(RGDyK) S55

7 Molar activity calculation S58

7.1 Calculation of A_m of [^{18}F]1 .... S58

7.2 Calculation of A_m of [^{18}F]2 .... S59

7.3 Calculation of A_m of [^{18}F]15 ([^{18}F]FTP-NHS) S60

7.4 Calculation of A_m of [^{18}F]FTP-Tyr S60

7.5 Calculation of A_m of [^{18}F]FTP-AMP S61

7.6 Calculation of A_m of [^{18}F]FTP-c(RGDyK) S62

7.7 Calculation of A_m of [^{18}F]FTP-HSA S63

8 Lipophilicity (Log *D*) S64

9 Stabilities of precursors and ^{18}F-labeled FTPs S65

9.1 Stabilities of precursors at different pHs S65

9.2 Stabilities of ^{18}F-labeled FTPs at different pHs S66

9.3 Stabilities of ^{18}F-labeled FTPs in PBS and serum S66

9.4 Storage stability of [^{18}F]FTP-c(RGDyK) in saline S71

9.5 The stabilities of [^{18}F]FTP-Pds-PEG *in vitro*......S72
9.6 Enzymatic stabilities of ^{18}F-labeled FTPs *in vitro*......S73
9.7 *In vivo* metabolic stabilities of ^{18}F-labeled FTPs......S73
9.8 *In vivo* stabilities of ^{18}F-labeled FTPs during dynamic PET imaging...S76
10 Biodistribution studies......S78
11 Similarity between the FTPs and phosphates......S79
11.1 Delocalized molecular orbital......S79
11.2 Molecular electrostatic potential......S80
11.3 Molecular docking......S80
11.4 Time-dependent cell uptake assay......S81
11.5 Cell proliferation assay......S81
11.6 Western Blot (WB)......S82
12 Animal models......S84
12.1 MDA-MB-453 cells xenograft animal model......S84
12.2 B16 cells xenograft animal model......S84
12.3 4T1 cells xenograft animal model......S85
13 MicroPET/CT imaging......S86
13.1 MicroPET/CT imaging with [^{18}F]FTP-c(RGDyK)......S86
13.2 MicroPET/CT imaging with [^{18}F]FTP-HSA......S86
13.3 MicroPET/CT imaging with [^{18}F]FTP-5F7......S86
13.4 MicroPET/CT imaging with [^{18}F]FTP-Tyr......S87
13.5 MicroPET/CT imaging with [^{18}F]FTP-Pds-PEG......S87
14 Radio-TLC and RCC data for ^{18}F-labeled products in Figure 2......S88
Supplementary references......S102
Appendix-Spectral data for new compounds......S104

Supplementary materials and methods

1 General information

1.1 General reagent information

All the reagents we used in the synthesis and biology experiment were purchased from Energy Chemical Co., Ltd. (China) or J&K Co., Ltd. (China) and were used without further purification. Column chromatography purification was performed on silica gel (54–74 μm, Qingdao Haiyang Chemical Co., Ltd., China). Anhydrous dichloromethane, anhydrous tetrahydrofuran (THF), anhydrous dimethyl sulfoxide (DMSO), anhydrous acetonitrile and anhydrous dimethylformamide (DMF) were purchased from Energy Chemical Co., Ltd. (China) and used without further drying.

1.2 General chemical analysis information

Thin layer chromatography (TLC) was performed on TLC Silica gel 60 F254 aluminum sheets (Merck, Germany), and visualized with short wave UV light (254 nm) or iodine staining.

Proton-1, carbon-13, fluorine-19 and phosphorus-31 nuclear magnetic resonance (^{1}H, ^{13}C, ^{19}F, ^{31}P NMR) spectra were recorded on an AS 400 MHz NMR spectrometer (ZhongKeNiuJin, China, ^{1}H NMR at 400 MHz, ^{13}C NMR at 100 MHz, ^{19}F NMR at 376 MHz, ^{31}P NMR at 162 MHz). Tetramethylsilane (TMS) was sometimes used as an internal standard for ^{1}H NMR, and all the chemical shifts were reported as δ values relative to the internal TMS. Chemical shifts for protons were reported in parts per million (ppm) downfield from TMS and were referenced to residual protium in the solvent (^{1}H NMR: $CDCl_3$ at 7.26 ppm, D_2O at 4.79 ppm, CD_3OD at 3.31 ppm, and DMSO-d_6 at 2.50 ppm). Chemical shifts for ^{13}C signals were referenced to the carbon resonances of the solvent peak (^{13}C NMR: $CDCl_3$ at 77.16 ppm, CD_3OD at 49.00 ppm, and DMSO-d_6 at 39.52 ppm). Multiplicity was defined by s (singlet), d (doublet), t (triplet), and m (multiplet). The coupling constants were reported in Hertz (Hz).

1.3 General radiochemistry information

Sep-Pak® light QMA cartridge (Waters, USA) were flushed with 5.0 mL $KHCO_3$ solution (0.5 mol L^{-1}), air, 10.0 mL water and air before use. Sep-Pak® Plus C18 cartridges (Waters, USA) were flushed with 5.0 mL alcohol, air, 10.0 mL water and air before use.

Radiochemical yields (RCYs, isolated yield, the amount of activity in the final product expressed as the percentage of starting activity) have been reported for all the proof-of-concept/potential PET tracers, while radiochemical conversions (RCCs) have been reported during the optimization of the ^{18}F-labeling methodology, which directly reflect the fluorination efficiency. Decay correction is a mathematical method of determining the amount of compound present given the amount of isotope remaining.

Non-decay-corrected RCYs, RCCs, and radiochemical purities (RCPs) were determined by radio-HPLC and radio-TLC. Radio-HPLC was performed on a Dionex Ulti-Mate 3000 HPLC (Thermo Fisher, USA) equipped with an SPD-20A UV detector (Thermo Fisher, USA) and a Gabi Star γ-radiation detector (Elysia Raytest, Germany). HPLC separations were achieved on a Waters XBridge C-18 column (5 μm, 10 mm × 250 mm, USA). HPLC analyses were achieved on a Nacalai Tesque Cosmosil 5C18-MS-II column (4.4 μm, 4.6 mm × 250 mm, Japan). Radio-TLC was performed on a Mini-Scan (Eckert & Ziegler, Germany) equipped with a Flow-Count (Bioscan, USA).

2 Synthesis

2.1 1, 2, 5–14

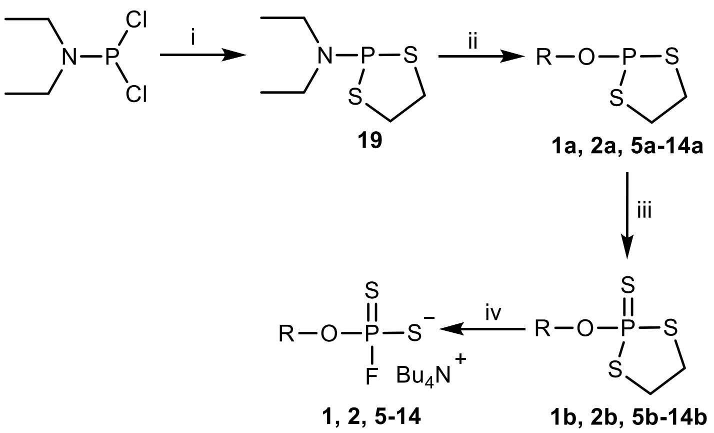

Scheme S1. General synthetic route of flouorothiophosphates (FTPs) **1**, **2**, **5–13** and the corresponding precursors. Reagents and conditions: (i) ethane-1,2-dithiol, benzene, RT, 2 h; (ii) alcohol, dichloromethane, RT, 3 h; (iii) S_8, dichloromethane, RT, overnight; (iv) TBAF, THF, RT, 2 min.

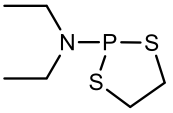

19

N,*N*-Diethyl-1,3,2-dithiaphospholan-2-amine (**19**):

1,1-Dichloro-*N,N*-diethylphosphanamine (1.00 g, 5.74 mmol) was dissolved in 40 mL anhydrous benzene and kept stirred in a reaction bottle. Ethane-1,2-dithiol (0.60 g, 6.37 mmol) and triethylamine (1.20 g, 11.86 mmol) dissolved in 10 mL of anhydrous benzene was added slowly from a constant pressure dropping funnel to the reaction bottle at 0 °C. The reaction mixture was then stirred at 0 °C for 30 min and slowly warmed up to RT over 2 h. The resultant white precipitate was isolated by filtration under an inert atmosphere, and the filtrate was concentrated and dried in vacuo to afford crude product **19** as a yellow oil. This crude product was used directly in the next procedure, because its instability in air do not allow further purification.[1]

^{31}P NMR (162 MHz, CDCl$_3$): δ 106.98.

1a, **2a**, **5a–14a** were synthesized following **General procedure A**:

19 was dissolved in 10 mL anhydrous CH_2Cl_2 under stirring. A solution of 5-(ethylthio)-1H-tetrazole (782 mg, 6.01 mmol) and an appropriate alcohol (6.00 mmol) in anhydrous CH_2Cl_2 (40.0 mL) was slowly added. The reaction mixture was stirred at RT for 3 h and immediately used in the next step without further disposition.

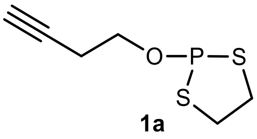

1a

2-(But-3-yn-1-yloxy)-1,3,2-dithiaphospholane (**1a**):

^{31}P NMR (162 MHz, $CDCl_3$): δ 148.93.

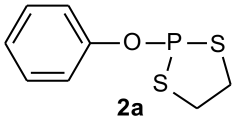

2a

2-Phenoxy-1,3,2-dithiaphospholane (**2a**):

^{31}P NMR (162 MHz, $CDCl_3$): δ 145.99.

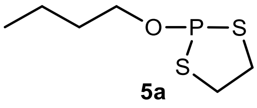

5a

2-Butoxy-1,3,2-dithiaphospholane (**5a**):

^{31}P NMR (162 MHz, $CDCl_3$): δ 148.98.

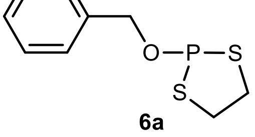

6a

2-(Benzyloxy)-1,3,2-dithiaphospholane (**6a**):

^{31}P NMR (162 MHz, $CDCl_3$): δ 146.87.

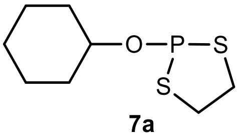

7a

2-(Cyclohexyloxy)-1,3,2-dithiaphospholane (**7a**):

^{31}P NMR (162 MHz, $CDCl_3$): δ 147.88.

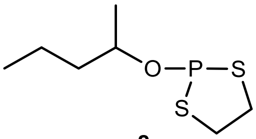

8a

2-(Pentan-2-yloxy)-1,3,2-dithiaphospholane (**8a**):

^{31}P NMR (162 MHz, $CDCl_3$): δ 148.94.

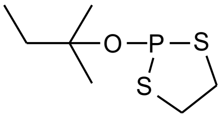

9a

2-(*tert*-Pentyloxy)-1,3,2-dithiaphospholane (**9a**):

^{31}P NMR (162 MHz, $CDCl_3$): δ 146.17.

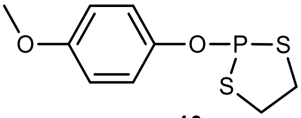

10a

2-(*p*-Tolyloxy)-1,3,2-dithiaphospholane (**10a**):

^{31}P NMR (162 MHz, $CDCl_3$): δ 151.61.

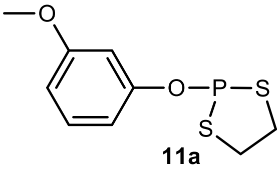

11a

2-(*m*-Tolyloxy)-1,3,2-dithiaphospholane (**11a**):

^{31}P NMR (162 MHz, $CDCl_3$): δ 154.51.

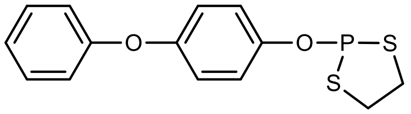

12a

2-(4-Phenoxyphenoxy)-1,3,2-dithiaphospholane (**12a**):

^{31}P NMR (162 MHz, CDCl$_3$): δ 156.23.

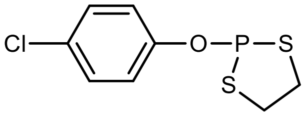

13a

2-(4-Chlorophenoxy)-1,3,2-dithiaphospholane (**13a**):

^{31}P NMR (162 MHz, CDCl$_3$): δ 159.77.

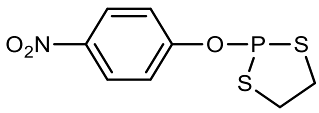

14a

2-(4-Nitrophenoxy)-1,3,2-dithiaphospholane (**14a**):

^{31}P NMR (162 MHz, CDCl$_3$): δ 160.02.

1b, **2b**, **5b–14b** were synthesized following **General procedure B**:

Dry elemental sulfur (100 mg) was added to a CH_2Cl_2 solution of **Xa** (**X** = **1**, **2**, **5**–**14**), and the reaction mixture was left stirring at RT overnight. The suspension was filtered, and the filtrate was concentrated in vacuo. The resulting crude residue was purified by column chromatography on silica gel to give **Xb**.

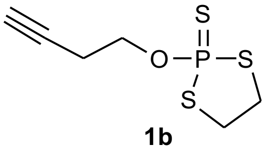

1b

2-(But-3-yn-1-yloxy)-1,3,2-dithiaphospholane 2-sulfide (**1b**): yield 62%.

^{1}H NMR (400 MHz, CDCl$_3$): δ 4.30–4.24 (m, 2H), 3.73–3.69 (m, 4H), 2.68 (t, 2H, J = 20.0 Hz), 2.07 (d, 1H, J = 27.5 Hz).

^{13}C NMR (101 MHz, CDCl$_3$): δ 79.45 (s), 70.50 (d, *J* = 8.8 Hz), 66.13 (s), 36.45 (s), 20.48 (d, *J* = 9.1 Hz).

^{31}P NMR (162 MHz, CDCl$_3$): δ 125.82.

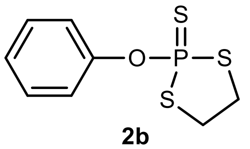

2b

2-Phenoxy-1,3,2-dithiaphospholane 2-sulfide (**2b**): yield 68%.

^{1}H NMR (400 MHz, CDCl$_3$): δ 7.43–7.39 (m, 2H), 7.32–7.26 (m, 2H), 3.81–3.61 (m, 4H).

^{13}C NMR (101 MHz, CDCl$_3$): δ 150.97 (d, *J* = 13.3 Hz), 129.58 (s), 125.87 (s), 122.04 (s), 41.92 (s).

^{31}P NMR (162 MHz, CDCl$_3$): δ 122.13.

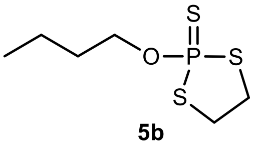

5b

2-Butoxy-1,3,2-dithiaphospholane 2-sulfide (**5b**): yield 58%.

^{1}H NMR (400 MHz, CDCl$_3$): δ 4.20–4.14 (m, 2H), 3.70–3.60 (m, 4H), 1.76–1.71 (m, 2H), 1.50–1.41 (m, 2H), 0.95 (t, *J* = 13.20 Hz, 3H).

^{13}C NMR (101 MHz, CDCl$_3$): δ 68.37 (s), 41.46 (s), 31.88 (s), 18.92 (s), 13.63 (s).

^{31}P NMR (162 MHz, CDCl$_3$): δ 124.59.

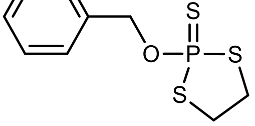

6b

2-(Benzyloxy)-1,3,2-dithiaphospholane 2-sulfide (**6b**): yield 70%.

^{1}H NMR (400 MHz, CDCl$_3$): δ 7.46–7.37 (m, 5H), 5.20 (d, *J* = 10.9 Hz, 2H), 3.76–3.60 (m, 4H).

^{13}C NMR (101 MHz, CDCl$_3$): δ 128.59 (d, *J* = 4.5 Hz), 128.33 (s), 69.75 (s), 41.42

(s).

^{31}P NMR (162 MHz, CDCl$_3$): δ 121.30.

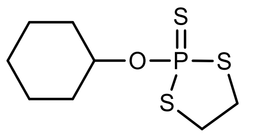

7b

2-(Cyclohexyloxy)-1,3,2-dithiaphospholane 2-sulfide (**7b**): yield 48%.

^{1}H NMR (400 MHz, CDCl$_3$): δ 4.73–4.64 (m, 1H), 3.82–3.54 (m, 4H), 2.00 (d, J = 12.2 Hz, 2H), 1.78 (d, J = 12.8 Hz, 2H), 1.63–1.54 (m, 3H), 1.37–1.28 (m, 3H).

^{13}C NMR (101 MHz, CDCl$_3$): δ 79.45 (d, J = 9.6 Hz), 41.42 (s), 33.29 (s), 25.11 (s), 23.63 (d, J = 13.3 Hz).

^{31}P NMR (162 MHz, CDCl$_3$): δ 118.14.

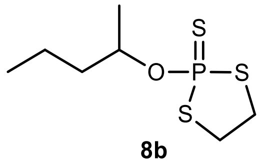

8b

2-(Pentan-2-yloxy)-1,3,2-dithiaphospholane 2-sulfide (**8b**): yield 32%.

^{1}H NMR (400 MHz, CDCl$_3$): δ 4.92–4.83 (tt, J = 12.7, 6.3 Hz, 1H), 3.74–3.63 (m, 4H), 1.83–1.67 (m, 1H), 1.59–1.53 (m, 6.6 Hz, 1H), 1.46–1.40 (m, 5H), 0.98 (t, J = 7.3 Hz, 3H).

^{13}C NMR (101 MHz, CDCl$_3$): δ 78.12 (d, J = 9.8 Hz), 41.49 (d, J = 13.6 Hz), 39.46 (d, J = 6.0 Hz), 21.46 (d, J = 3.1 Hz), 18.55 (s), 13.90 (s).

^{31}P NMR (162 MHz, CDCl$_3$): δ 118.86.

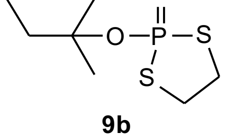

9b

2-(*tert*-Pentyloxy)-1,3,2-dithiaphospholane 2-sulfide (**9b**): yield 10%.

^{1}H NMR (400 MHz, CDCl$_3$): δ 3.55–3.45 (m, 4H), 1.65 (q, J = 7.4 Hz, 2H), 1.38 (s,

6H), 0.92 (t, J = 7.4 Hz, 3H).

^{13}C NMR (101 MHz, CDCl$_3$): δ 81.82 (d, J = 7.6 Hz), 40.42 (s), 35.61 (d, J = 6.0 Hz), 27.71 (d, J = 10.7 Hz), 8.50 (s).

^{31}P NMR (162 MHz, CDCl$_3$): δ 118.84.

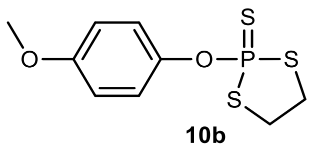

10b

2-(*p*-Tolyloxy)-1,3,2-dithiaphospholane 2-sulfide (**10b**): yield 64%.

^{1}H NMR (400 MHz, CDCl$_3$): δ 7.24–7.22 (m, 2H), 6.91 (d, J = 8.8 Hz, 2H), 3.84 (s, 3H), 3.78–3.57 (m, 4H).

^{13}C NMR (101 MHz, CDCl$_3$): δ 157.32 (s), 144.53 (m), 129.93 (d), 114.45 (d), 55.59 (s), 41.82 (s).

^{31}P NMR (162 MHz, CDCl$_3$): δ 118.31.

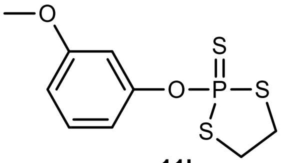

11b

2-(*m*-Tolyloxy)-1,3,2-dithiaphospholane 2-sulfide (**11b**): yield 57%.

^{1}H NMR (400 MHz, CDCl$_3$): δ 7.32–7.28 (t, 1H), 6.92–6.84 (m, 3H), 3.85 (s, 3H), 3.81–3.60 (m, 4H).

^{13}C NMR (101 MHz, CDCl$_3$): δ 160.49 (s), 151.79 (s), 129.79 (s), 114.01 (s), 111.17 (s), 107.95 (s), 55.35 (s), 41.77 (s).

^{31}P NMR (162 MHz, CDCl$_3$): δ 118.31.

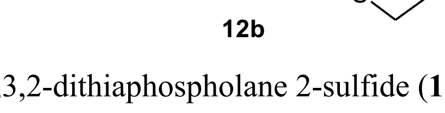

12b

2-(4-Phenoxyphenoxy)-1,3,2-dithiaphospholane 2-sulfide (**12b**): yield 48%.

^{1}H NMR (400 MHz, DMSO-*d*$_6$): δ 7.46–7.42 (m, 2H), 7.33–7.30 (m, 2H), 7.21–7.17

(m, J = 7.4 Hz, 1H), 7.09–7.04 (m, 4H), 3.99–3.72 (m, 4H).

^{13}C NMR (101 MHz, DMSO-d_6): δ 157.03 (s), 154.69 (s), 146.44 (d, J = 13.2 Hz), 130.58 (s), 124.16 (s), 124.16–123.70 (m), 119.98 (s), 119.10 (s), 42.72 (s).

^{31}P NMR (162 MHz, DMSO-d_6): δ 122.31 (s).

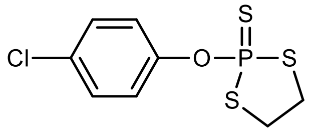

13b

2-(4-Chlorophenoxy)-1,3,2-dithiaphospholane 2-sulfide (**13b**): yield 52%.

^{1}H NMR (400 MHz, CD_3OD): δ 7.42 (d, J = 8.4 Hz, 2H), 7.30–7.28 (m, 2H), 3.89–3.71 (m, 4H).

^{13}C NMR (101 MHz, CD_3OD): δ 149.73 (s), 130.80 (s), δ 129.13 (d, J = 2.2 Hz), 123.36 (d, J = 5.0 Hz), 41.75 (s).

^{31}P NMR (162 MHz, CD_3OD): δ 122.02 (s).

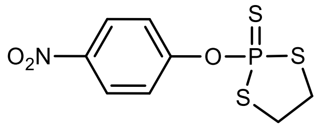

14b

The unsuccessful oxidation of **14a** to **14b** with elemental sulfur was probably due to the strong electron-withdrawing group -NO_2, while weak electron-withdrawing groups, such as, halogens, did not affect the precursor synthesis.

1, **2**, **5**–**13** were synthesized following **General procedure C**:

0.6 mL TBAF (1.0 M in THF) was added into a solution of **Xb** (0.40 mmol) in 5.0 mL THF. The mixture was stirred at RT for 2 min. After concentration in vacuo, the resulting crude residue was purified by column chromatography on silica gel to give the phosphorofluoridodithioate product **X**.[2]

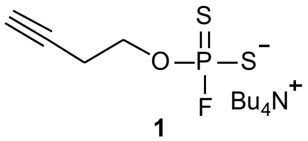

1

O-(But-3-yn-1-yl) phosphorofluoridodithioate (**1**): yield 90%.

^{1}H NMR (400 MHz, CDCl$_3$): δ 4.27–4.20 (m, 2H), 3.35 (t, *J* = 52.9 Hz, 8H), 1.99 (t, *J* = 9.7 Hz, 1H), 2.65–2.63 (m, 2H), 1.75–1.67 (m, 8H), 1.55–1.40 (m, 8H), 1.04 (t, *J* = 8.0 Hz, 12H).

^{13}C NMR (101 MHz, CDCl$_3$): δ 80.73 (s), 69.48 (s), 64.30 (d, *J* = 7.9 Hz), 59.03 (s), 24.16 (s), 20.60 (d, *J* = 8.3 Hz), 19.76 (s), 13.69 (s).

^{19}F NMR (376 MHz, CDCl$_3$): δ -7.28 (d, *J* = 1105.44 Hz).

^{31}P NMR (162 MHz, CDCl$_3$): δ 119.44 (d, *J* = 1103.22 Hz).

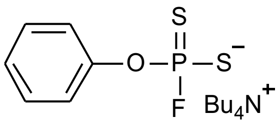

2

O-Phenyl phosphorofluoridodithioate (**2**): yield 93%.

^{1}H NMR (400 MHz, CD$_3$OD): δ 7.34–7.27 (m, 4H), 7.16 (t, *J* = 14.3 Hz, 1H), 3.26 (t, *J* = 17.7 Hz, 8H), 1.67–1.48 (m, 8H), 1.44–1.39 (m, 8H), 1.04 (t, *J* = 7.8 Hz, 12H).

^{13}C NMR (101 MHz, CD$_3$OD): δ 152.49 (d, *J* = 10.3 Hz), 128.69 (s), 123.76 (s), 121.55 (s), 58.34 (s), 23.59 (s), 19.37 (s), 12.72 (s).

^{19}F NMR (376 MHz, CD$_3$OD): δ -4.15 (d, *J* = 1101.68 Hz).

^{31}P NMR (162 MHz, CD$_3$OD): δ 114.83 (d, *J* = 1114.56 Hz).

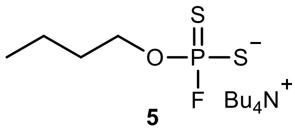

5

O-Butyl phosphorofluoridodithioate (**5**): yield 88%.

^{1}H NMR (400 MHz, CDCl$_3$): δ 4.16–4.11 (m, *J* = 16.5, 6.8 Hz, 2H), 3.40–3.36 (m, 8H), 2.20 (s, 10H), 1.74–1.70 (m, 10H), 1.50–1.40 (m, 10H), 1.05 (t, *J* = 7.3 Hz, 12H), 0.95 (t, *J* = 7.4 Hz, 3H).

^{13}C NMR (101 MHz, CDCl$_3$): δ 66.55 (d, *J* = 8.2 Hz), 58.85 (s), 32.52 (d, *J* = 8.3

Hz), 24.11 (s), 19.72 (s), 18.97 (s), 13.78 (d, J = 11.6 Hz).

^{19}F NMR (376 MHz, CDCl$_3$): δ -4.15 (d, J = 1097.92 Hz).

^{31}P NMR (162 MHz, CDCl$_3$): δ 123.19 (d, J = 1098.36 Hz).

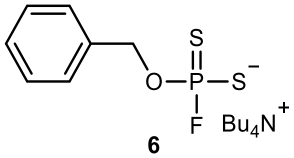

6

O-Benzyl phosphorofluoridodithioate (**6**): yield 89%.

^{1}H NMR (400 MHz, CD$_3$OD): δ 7.47–7.30 (m, 5H), 5.13 (d, J = 9.4 Hz, 2H), 3.29–3.25 (m, 8H), 1.79–1.65 (m, 8H), 1.50–1.41 (m, 8H), 1.05 (t, J = 7.3 Hz, 12H).

^{13}C NMR (101 MHz, CD$_3$OD): δ 137.75 (d, J = 9.6 Hz), 127.90 (s), 127.37 (d, J = 6.8 Hz), 67.95 (s), 58.18 (s), 23.47 (s), 19.32 (s), 12.59 (s).

^{19}F NMR (376 MHz, CD$_3$OD): δ -8.82 (d, J = 1116.72 Hz).

^{31}P NMR (162 MHz, CD$_3$OD): δ 120.64 (d, J = 1108.08 Hz).

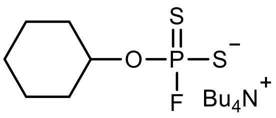

7

O-Cyclohexyl phosphorofluoridodithioate (**7**): yield 92%.

^{1}H NMR (400 MHz, CDCl$_3$): δ 4.62 (s, 1H), 3.43–3.30 (m, 8H), 2.05 (d, J = 11.6 Hz, 2H), 1.87–1.63 (m, 12H), 1.58–1.41 (m, 12H), 1.04 (t, J = 7.3 Hz, 12H).

^{13}C NMR (101 MHz, CDCl$_3$): δ 76.31 (d, J = 8.6 Hz), 59.04 (s), 33.84 (s), 25.49 (s), 24.19 (s), 19.74 (s), 13.66 (s).

^{19}F NMR (376 MHz, CDCl$_3$): δ -3.13 (d, J = 1097.92 Hz).

^{31}P NMR (162 MHz, CDCl$_3$): δ 117.18 (d, J = 1197.18 Hz).

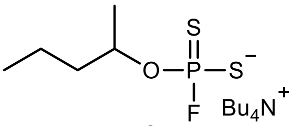

8

O-(Pentan-2-yl) phosphorofluoridodithioate (**8**): yield 92%.

^{1}H NMR (400 MHz, CDCl$_3$): δ 4.84–4.74 (m, 1H), 3.42–3.33 (m, 8H), 1.75–1.68 (m, 8H), 1.56–1.37 (m, 12H), 1.34 (d, *J* = 6.2 Hz, 3H), 1.04 (t, *J* = 7.3 Hz, 12H), 0.93 (t, *J* = 7.1 Hz, 3H).

^{13}C NMR (101 MHz, CDCl$_3$): δ 74.56 (s), 59.02 (s), 39.97 (s), 24.21 (s), 21.69 (s), 19.78 (s), 18.77 (s), 14.15 (s), 13.73 (s).

^{19}F NMR (376 MHz, CDCl$_3$): δ -1.13 (d, *J* = 1097.92 Hz).

^{31}P NMR (162 MHz, CDCl$_3$): δ 119.32 (d, *J* = 1099.98 Hz).

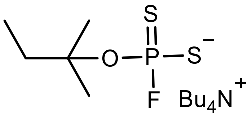

9

O-(*tert*-Pentyl) phosphorofluoridodithioatee (**9**): yield 80%.

^{1}H NMR (400 MHz, CDCl$_3$): δ 3.16–3.11 (m, 8H), 1.87–1.69 (m, 10H), 1.56–1.42 (m, 14H), 1.05–0.98 (m, 15H).

^{13}C NMR (101 MHz, CDCl$_3$): δ 81.72 (s), δ 52.37 (s), 35.82 (s), 27.37 (d, *J* = 13.1 Hz), 25.25 (s), 19.97 (s), 13.57 (s), 8.44 (s).

^{19}F NMR (376 MHz, CDCl$_3$): δ -10.70 (d, *J* = 1104.9 Hz).

^{31}P NMR (162 MHz, CDCl$_3$): δ 119.79 (d, *J* = 1104.3 Hz).

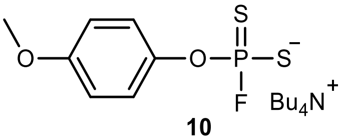

10

O-(*p*-Tolyl) phosphorofluoridodithioate (**10**): yield 81%.

^{1}H NMR (400 MHz, CD$_3$OD): δ 7.19 (d, *J* = 7.2 Hz, 2H), 6.88 (d, *J* = 9.0 Hz, 2H), 3.81 (s, 3H), 3.30–3.25 (m, 8H), 1.74–1.68 (m, 8H), 1.48–1.41 (m, 8H), 1.05 (t, *J* = 7.3 Hz, 12H).

^{13}C NMR (101 MHz, CD$_3$OD): δ 156.32 (s), 146.04 (d, *J* = 10.7 Hz), 122.29 (s), 113.60 (s), 58.19 (s), 54.63 (s), 23.47 (s), 19.32 (s), 12.58 (s).

^{19}F NMR (376 MHz, CD$_3$OD): δ -11.83 (d, *J* = 1105.44 Hz).

^{31}P NMR (162 MHz, CD$_3$OD): δ 118.36 (d, *J* = 1106.46 Hz).

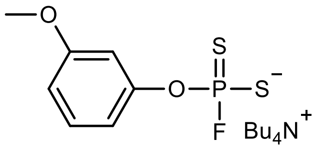

11

O-(*m*-Tolyl) phosphorofluoridodithioate (**11**): yield 91%.

^{1}H NMR (400 MHz, CD_3OD): δ 7.24–7.20 (m, 1H). 6.77 (s, 1H), 6.77 (d, *J* = 8 Hz, 1H), 6.74 (d, *J* = 12 Hz, 1H), 3.82 (s, 3H), 3.31–3.22 (m, 8H), 1.76–1.64 (m, 8H), 1.52–1.39 (m, 8H), 1.05 (t, *J* = 7.3 Hz, 12H).

^{13}C NMR (101 MHz, CD_3OD): δ 160.33 (s), 153.38 (s), 128.88 (s), 113.74 (d, *J* = 4.8 Hz), 109.45 (s), 107.60 (d, *J* = 4.8 Hz), 58.54–58.05 (m), 54.47 (s), 23.52 (s), 19.32 (s), 12.56 (s).

^{19}F NMR (376 MHz, CD_3OD): δ -10.10 (d, *J* = 1116.72 Hz).

^{31}P NMR (162 MHz, CD_3OD): δ 111.76 (d, *J* = 1117.80 Hz).

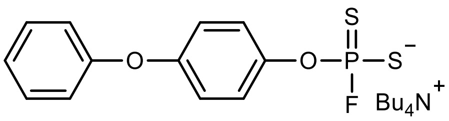

12

O-(4-Phenoxyphenyl) phosphorofluoridodithioate (**12**): yield 96%.

^{1}H NMR (400 MHz, CD_3OD): δ 7.30–7.26 (m, 2H), 7.19 (d, *J* = 7.2 Hz, 2H), 7.04–7.01 (m, 1H), 6.93–6.87 (m, 4H), 3.22–3.18 (m, 8H), 1.66–1.58 (m, 8H), 1.41–1.32 (m, 8H), 0.96 (t, *J* = 7.3 Hz, 12H).

^{13}C NMR (101 MHz, CD_3OD): δ 156.31 (s), 151.80 (s), 146.72 (s), 127.95 (s), 117.56 (s), 116.41 (s), 56.77 (s), 22.02 (s), 17.85 (s), 11.16 (s).

^{19}F NMR (376 MHz, CD_3OD): δ -8.78 (d, *J* = 1116.72 Hz).

^{31}P NMR (162 MHz, CD_3OD): δ 114.35 (d, *J* = 1119.42 Hz).

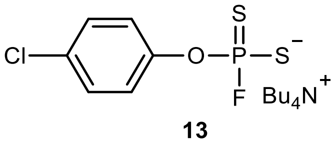

13

O-(4-Chlorophenyl) phosphorofluoridodithioate (**13**): yield 87%.

^{1}H NMR (400 MHz, CD_3OD): δ 7.17 (d, *J* = 8.9 Hz, 2H), 6.78 (d, *J* = 8.9 Hz, 2H), 3.30–3.26 (m, 8H), 1.74–1.67 (m, 8H), 1.53–1.40 (m, 8H), 1.06 (t, *J* = 7.4 Hz, 12H).

^{13}C NMR (101 MHz, CD_3OD): δ 129.75 (s), 128.04 (s), 117.08 (s), 115.54 (s), 58.27 (s), 23.46 (s), 19.31 (s), 13.15 (s).

^{19}F NMR (376 MHz, CD_3OD): δ -4.62 (d, *J* = 1099.6 Hz).

^{31}P NMR (162 MHz, CD_3OD): δ 117.25 (d, *J* = 1101.4 Hz).

2.2 *O*-(But-3-yn-1-yl) phosphorofluoridothioate (3)

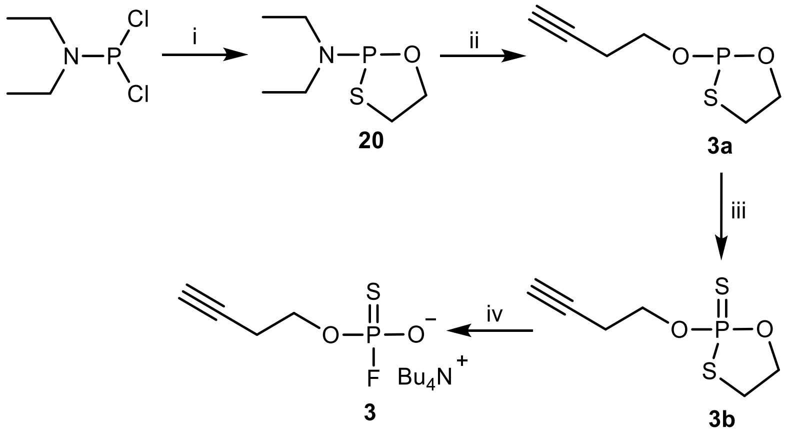

Scheme S2. Synthetic route of **3**. Reagents and conditions: (i) 2-mercaptoethan-1-ol, benzol and hexane, RT, 2 h; (ii) but-3-yn-1-ol, dichloromethane, RT, 3 h; (iii) S_8, dichloromethane, RT, overnight; (iv) TBAF, THF, RT, 2 min.

N,*N*-Diethyl-1,3,2-oxathiaphospholan-2-amine (**20**)

1,1-Dichloro-*N,N*-diethylphosphanamine (1.00 g, 5.74 mmol) was dissolved in 40 mL of anhydrous benzene and kept stirred in a reaction bottle. 2-Mercaptoethan-1-ol (0.45 g, 5.76 mmol) and triethylamine (1.20 g, 11.86 mmol) dissolved in 10 mL of anhydrous benzene was added slowly from a constant pressure dropping funnel to the reaction bottle at 0 °C. The reaction mixture was then stirred at 0 °C for 30 min and slowly warmed to RT over 2 h. The resultant white precipitate was isolated by filtration under an inert atmosphere, and the filtrate was concentrated and dried in

vacuo to get crude product as a yellow oil. **20** was used directly in the next procedure, because its instability in air do not allow further purification.[3]

2-(But-3-yn-1-yloxy)-1,3,2-oxathiaphospholane (**3a**)

20 was dissolved in 10 mL anhydrous CH_2Cl_2 and the solution was kept stirred. A solution of 5-(ethylthio)-1H-tetrazole (782 mg, 6.01 mmol) and but-3-yn-1-ol (0.40 g, 6.00 mmol) in anhydrous CH_2Cl_2 (40.0 mL) was slowly added. The reaction mixture was stirred at RT for 3 h and immediately used in the next step without further disposition.

^{31}P NMR (162 MHz, $CDCl_3$): δ 104.18 (s).

2-(But-3-yn-1-yloxy)-1,3,2-oxathiaphospholane 2-sulfide (**3b**)

3b was synthesized following **General procedure B**. Yield 53%.

^{1}H NMR (400 MHz, $CDCl_3$): δ 4.78–4.43 (m, 2H), 4.40–4.16 (m, 2H), 3.74–3.49 (m, 2H), 2.70 (td, *J* = 6.8, 2.6 Hz, 2H), 2.11 (s, 1H).

^{13}C NMR (101 MHz, $CDCl_3$): δ 79.45 (s), 70.50 (d, *J* = 17.3 Hz), 66.13 (s), 36.45 (s), 20.48 (s).

^{31}P NMR (162 MHz, $CDCl_3$): δ 104.08 (s).

O-(But-3-yn-1-yl) phosphorofluoridothioate (**3**)

0.6 mL TBAF (1.0 M in THF) was added into a solution of **3b** (0.08 g, 0.39 mmol) in 5.0 mL THF. The mixture was stirred at RT for 2 min. After concentration in vacuo, the resulting crude residue was purified by column chromatography on silica gel to afford **3**. Yield 89%.

^{1}H NMR (400 MHz, $CDCl_3$): δ 4.03–3.94 (m, 2H), 3.20–3.16 (m, 8H), 2.45 (t, *J* = 7.3 Hz, 2H), 1.88 (s, 1H), 1.57–1.51 (m, 8H), 1.36–1.31 (m, 8H), 0.89 (t, *J* = 7.2 Hz, 12H).

^{13}C NMR (101 MHz, $CDCl_3$): δ 80.63 (s), 69.49 (s), 64.03 (s), 58.60 (s), 23.86 (s), 20.58 (s), 19.55 (s), 13.53 (s).

^{19}F NMR (376 MHz, D_2O): δ -32.51 (d, *J* = 1045.28 Hz).

^{31}**P NMR (162 MHz, D$_2$O)**: δ 52.98 (d, J = 1048.14 Hz).

2.3 *O*-(4-(((2,5-Dioxopyrrolidin-1-yl)oxy)carbonyl)benzyl) phosphorofluoridodithioate (15)

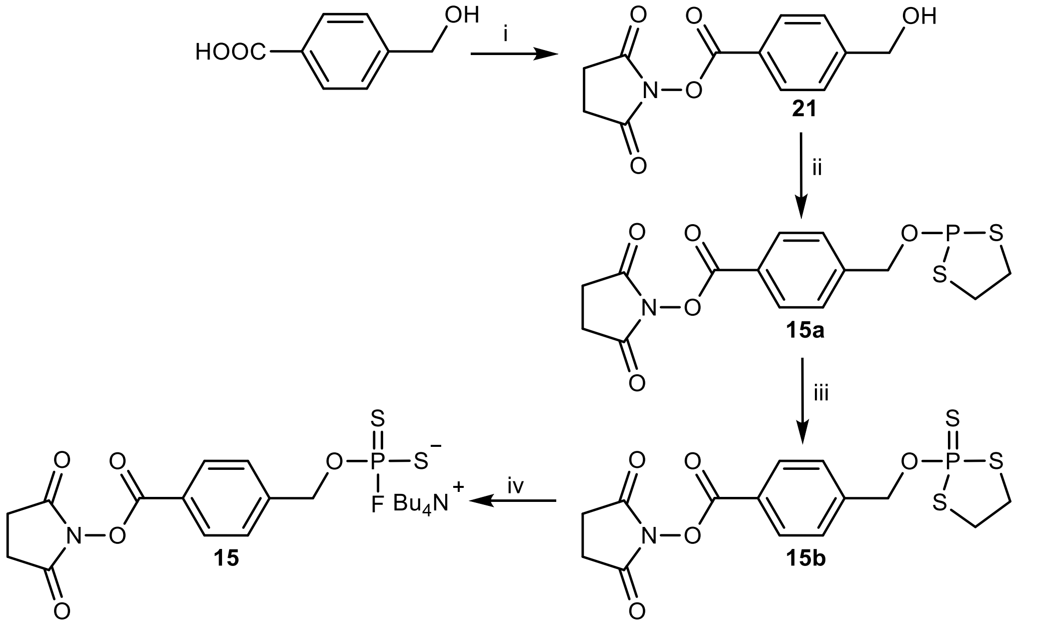

Scheme S3. Synthetic route of **15**. Reagents and conditions: (i) *N*-Hydroxysuccinimide, DCC, THF, RT, 24 h; (ii) dichloromethane, RT, 3 h; (iii) S_8, dichloromethane, RT, overnight; (iv) TBAF, THF, RT, 2 min.

2,5-Dioxopyrrolidin-1-yl 4-(hydroxymethyl)benzoate (**21**)

N-Hydroxysuccinimide (1.06 g, 9.21 mmol) was added into a 50.0 mL round bottom flask with a stir bar, and placed under N_2 atmosphere. 4-(Hydroxymethyl)benzoic acid (1.30 g, 8.54 mmol) was dissolved in 15 mL of anhydrous THF under N_2 atmosphere and added, with gentle stirring, to the flask charged with *N*-hydroxysuccinimide. The reaction round bottom flask was cooled to 0 °C in an ice bath, before a solution of dicyclohexylcarbodiimide (DCC) (3.59 g, 17.40 mmol) dissolved in 15.0 mL anhydrous THF was added drop-wise to the mixture with gentle stirring. Upon complete addition of DCC, the reaction flask was removed from the ice bath and the reaction was allowed to proceed for another 16 h. The reaction mixture was filtered to remove 1,3-dicyclohexylurea (DCU) precipitate, and the filtrate was dried. The

residue was suspended in 50.0 mL of diethyl ether, filtered, and washed with an additional 75.0 mL of diethyl ether (3 × 25 mL). The crude product was purified by column chromatography on silica gel to give a sticky product. The material was then dried, yielding **21** as a white powder.[4] Yield 71%.

^{1}H NMR (400 MHz, DMSO-*d_6*): δ 8.09 (d, *J* = 8.2 Hz, 2H), 7.62 (d, *J* = 8.2 Hz, 2H), 5.50 (t, *J* = 5.7 Hz, 1H), 4.67 (d, *J* = 5.7 Hz, 2H), 2.92 (s, 4H).

^{13}C NMR (101 MHz, DMSO-*d_6*): δ 170.80 (s), 162.17 (s), 151.44 (s), 130.39 (s), 127.32 (s), 123.16 (s), 62.74 (s), 26.00 (s).

2,5-Dioxopyrrolidin-1-yl 4-(((1,3,2-dithiaphospholan-2-yl)oxy)methyl)benzoate (**15a**)

15a was synthesized following **General procedure A**.

^{31}P NMR (162 MHz, $CDCl_3$): δ 148.68 (s).

2,5-Dioxopyrrolidin-1-yl 4-(((2-sulfido-1,3,2-dithiaphospholan-2-yl)oxy)methyl)benzoate (**15b**)

15b was synthesized following **General procedure B**. Yield 58%.

^{1}H NMR (400 MHz, $CDCl_3$): δ 8.19 (d, *J* = 8.1 Hz, 2H), 7.59 (d, *J* = 8.1 Hz, 2H), 5.29 (d, *J* = 11.5 Hz, 2H), 3.80–3.62 (m, 4H), 2.96 (s, 4H).

^{13}C NMR (101 MHz, $CDCl_3$): δ 169.17 (s), 161.50 (s), 142.91 (s), 130.98 (s), 128.02 (s), 125.09 (s), 68.39 (s), 41.58 (s), 25.69 (s).

^{31}P NMR (162 MHz, $CDCl_3$): δ 122.90 (s).

O-(4-(((2,5-Dioxopyrrolidin-1-yl)oxy)carbonyl)benzyl) phosphorofluoridodithioate (**15**)

15 was synthesized following **General procedure C**. Yield 78%.

^{1}H NMR (400 MHz, CD_3OD): δ 8.09 (d, *J* = 8.1 Hz, 2H), 7.70 (d, *J* = 8.1 Hz, 2H), 5.53 (s, 1H), 5.27 (d, *J* = 10.2 Hz, 1H), 3.29–3.25 (m, 8H), 2.95 (s, 4H), 1.64–1.59 (m, 8H), 1.47–1.40 (m, 8H), 1.05 (t, *J* = 7.3 Hz, 12H).

^{13}C NMR (101 MHz, CD_3OD): δ 168.25 (s), 143.20 (d, *J* = 9.4 Hz), 129.83 (s), 129.43 (d, *J* = 13.7 Hz), 127.14 (s), 126.91 (s), 67.13 (s), 58.20 (s), 26.07 (s), 23.47

(s), 19.33 (s), 12.61 (s).

^{19}F NMR (376 MHz, CD_3OD): δ -7.10 (d, *J* = 1106.3 Hz).

^{31}P NMR (162 MHz, CD_3OD): δ 115.28 (d, *J* = 1103.22 Hz).

2.4 *O*-(Cyclooct-4-en-1-yl) phosphorofluoridodithioate (16)

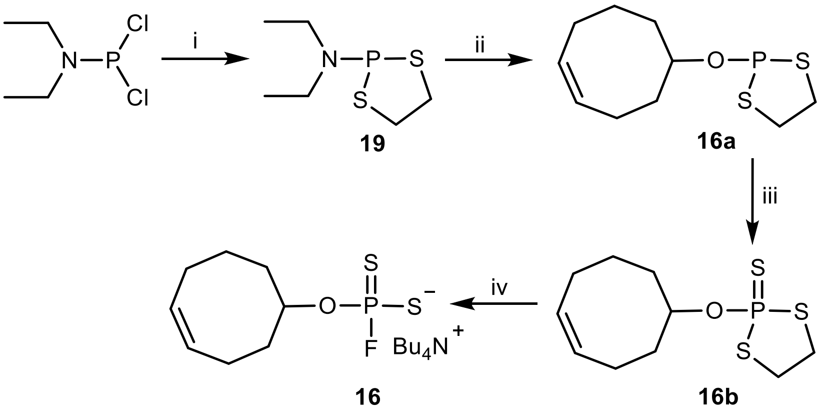

Scheme S4. Synthetic route of **16**. Reagents and conditions: (ii) ethane-1,2-dithiol, benzol and hexane, RT, 2 h; (ii) Cyclooct-4-enol, dichloromethane, RT, 3 h; (iii) S_8, dichloromethane, RT, overnight; (iv) TBAF, THF, RT, 2 min.

Cis-Cyclooctene is cheaper and easier to obtain, and we use it as a substitute for trans-cyclooctene in this methodology research.

2-(Cyclooct-4-en-1-yloxy)-1,3,2-dithiaphospholane (**16a**)

16a was synthesized following **General procedure** A.

^{31}P NMR (162 MHz, $CDCl_3$): δ 146.95 (s).

(Z)-2-(Cyclooct-4-en-1-yloxy)-1,3,2-dithiaphospholane 2-sulfide (**16b**)

16b was synthesized following **General procedure B**. Yield 33%.

^{1}H NMR (400 MHz, $CDCl_3$): δ 5.73–5.60 (m, 2H), 4.80–4.74 (m, 1H), 3.68–3.58 (m, 4H), 2.42–2.32(m, 1H), 2.17–1.99 (m, 6H), 1.89–1.57 (m, 3H).

^{13}C NMR (101 MHz, $CDCl_3$): δ 129.72 (s), 129.42 (s), 82.46 (s), 41.59 (d, *J* = 14.6 Hz, 2H), 35.49 (s), 34.53 (s), 25.52 (s), 24.52 (s), 22.33 (s).

^{31}P NMR (162 MHz, CDCl$_3$): δ 118.60 (s).

O-(Cyclooct-4-en-1-yl) phosphorofluoridodithioate (**16**)

16 was synthesized following **General procedure C**. Yield 78%.

^{1}H NMR (400 MHz, CDCl$_3$): δ 5.76–5.58 (m, 2H), 4.85–4.78 (m, 1H), 3.41–3.37 (m, 8H), 2.36–2.34 (m, 1H), 2.34–2.09 (m, 6H), 1.82–1.62 (m, 11H), 1.53–1.49 (m, 8H), 1.06 (*t*, J = 7.3 Hz, 12H).

^{13}C NMR (101 MHz, CDCl$_3$): δ 130.08 (s), 129.30 (s), 59.09 (s), 35.69 (s), 34.66 (s), 25.57 (s), 24.66 (s), 24.22 (s), 22.86 (s), 19.77 (s), 13.68 (s).

^{19}F NMR (376 MHz, CDCl$_3$): δ -4.32 (d, J = 1164.3 Hz).

^{31}P NMR (162 MHz, CDCl$_3$): δ 118.71 (d, J = 2704 Hz).

2.5 (*S*)-*O*-(4-(2-Amino-2-carboxyethyl)phenyl) phosphorofluoridodithioate (FTP-Tyr, 17)

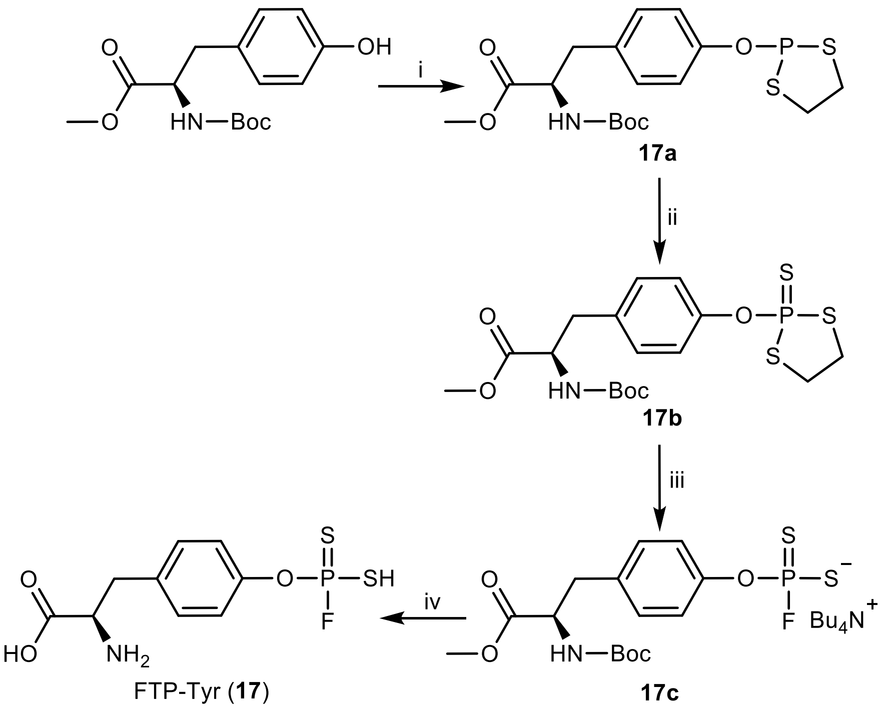

Scheme S5. Synthetic route of FTP-Tyr (**17**) and the corresponding precursor. Reagents and conditions: (i) *N*,*N*-diethyl-1,3,2-dithiaphospholan-2-amine (**19**), RT, 3

h; (ii) S_8, dichloromethane, RT, overnight; (iii) TBAF, THF, RT, 2 min; (iv) 1) methanol, NaOH, RT, 2 h, 2) methanol, HCl, RT, 1 h.

Methyl (*S*)-3-(4-((1,3,2-Dithiaphospholan-2-yl)oxy)phenyl)-2-((*tert*-butoxycarbonyl)amino)propanoate (**17a**)

17a was synthesized following **General procedure A**.

^{31}P NMR (162 MHz, $CDCl_3$): δ 156.12.

Methyl (*S*)-2-((*tert*-Butoxycarbonyl)amino)-3-(4-((2-sulfido-1,3,2-dithiaphospholan-2-yl)oxy)phenyl)propanoate (**17b**)

17b was synthesized following **General procedure B**. Yield 54%.

^{1}H NMR (400 MHz, CD_3OD): δ 7.28–7.22 (m, 4H), 4.46–4.29 (m, 1H), 3.90–3.60 (m, 7H), 3.17–3.12 (m, 1H), 3.01–2.93 (m, 1H), 1.42 (s, 9H).

^{13}C NMR (101 MHz, CD_3OD): δ 172.92 (s), 156.35 (s),149.60 (d, *J* = 13.1 Hz), 135.77 (s), 130.78 (s), 122.00 (s), 78.77 (s), 55.44 (s), 52.23 (s), 42.64 (s), 28.60 (s).

^{31}P NMR (162 MHz, $CDCl_3$): δ 121.04.

(*S*)-*O*-(4-(2-((*tert*-Butoxycarbonyl)amino)-3-methoxy-3-oxopropyl)phenyl) phosphorofluoridodithioate (**17c**)

17c was synthesized following **General procedure C**. Yield 47%.

^{1}H NMR (400 MHz, CD_3OD): δ 7.22–7.16 (m, 4H), 4.35–4.32 (m, 1H), 3.72 (s, 2H), 3.35 (s, 3H), 3.35–3.27 (m, 8H), 1.75–1.70 (m, 8H), 1.54–1.33 (m, 17H), 1.06 (t, *J* = 7.1 Hz, 12H).

^{13}C NMR (101 MHz, CD_3OD): δ 168.25 (s), 143.20 (d, *J* = 9.4 Hz), 129.83 (s), 129.43 (d, *J* = 13.7 Hz), 127.14 (s), 126.91 (s), 67.13 (s), 58.20 (s), 23.47 (s), 19.33 (s), 12.61 (s).

^{19}F NMR (376 MHz, $CDCl_3$): δ -3.03 (s), -5.87 (s).

^{31}P NMR (162 MHz, CD_3OD): δ 114.90 (d, *J* = 1112.94 Hz).

(*S*)-*O*-(4-(2-Amino-2-carboxyethyl)phenyl) phosphorofluoridodithioate (FTP-Tyr, **17**)

10 mL NaOH (2.0 M) was added into a solution of **17c** (65 mg, 1.0 mmol) in 30.0 mL MeOH for 2 h, the reaction mixture was neutralized by 1.0 M HCl and extracted with ethyl acetate, and then the organic phase was dried over anhydrous Na_2SO_4 and evaporated under vacuum. The residue was dissolved in MeOH (15.0 mL) and 5.0 M HCl (15.0 mL) or TFA was added and stirred at RT for 1 h. The product was recrystallized from diethyl ether. Yield 41%.

^{1}H NMR (400 MHz, D_2O): δ 7.47–7.37 (m, 4H), 4.03 (dd, *J* = 8.1, 5.1 Hz, 1H), 3.42–3.37 (m, 1H), 3.22–3.15 (m, 1H).

^{13}C NMR (101 MHz, D_2O): δ 175.88 (s), 150.72 (d, *J* = 17.3 Hz), 133.29 (s), 130.83 (s), 122.31 (s), 56.60 (s), 36.90 (s).

^{19}F NMR (376 MHz, $CDCl_3$): δ 2.08 (d, *J* = 832 Hz)

^{31}P NMR (162 MHz, D_2O): δ 116.71 (d, *J* = 1124.28 Hz).

2.6 *O*-(((2*R*,3*S*,4*R*,5*R*)-5-(6-Amino-9H-purin-9-yl)-3,4-dihydroxytetrahydrofuran-2-yl)methyl) phosphorofluoridodithioate (FTP-AMP, 18)

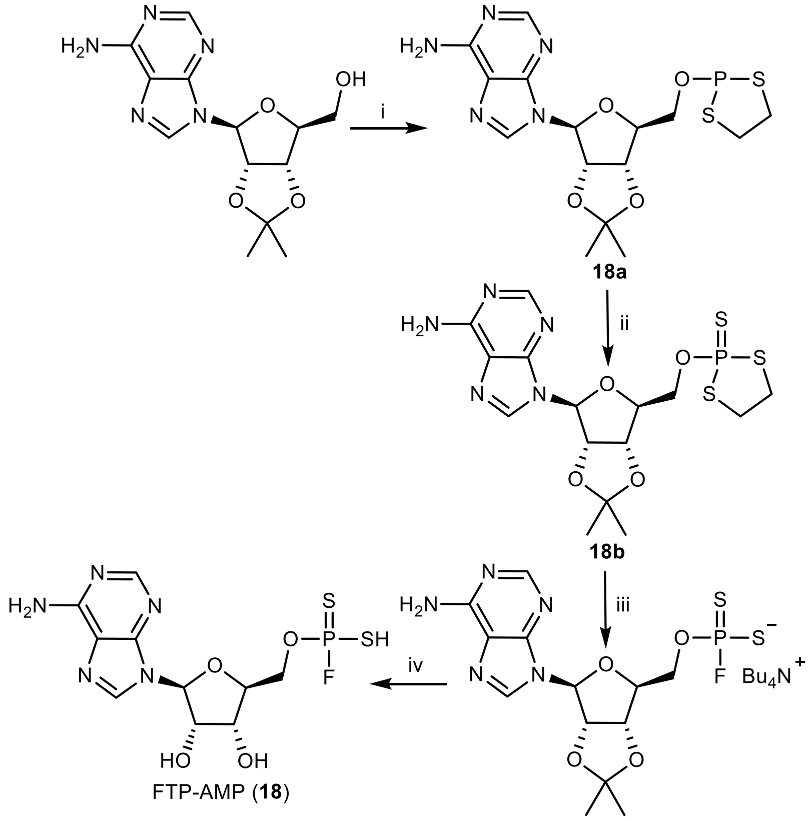

Scheme S6. Synthetic route of FTP-AMP (**18**) and the corresponding precursor. Reagents and conditions: (i) *N*,*N*-diethyl-1,3,2-dithiaphospholan-2-amine (**19**), dichloromethane, RT, 3 h; (ii) S_8, dichloromethane, RT, overnight; (iii) TBAF, THF, RT, 2 min; (iv) TFA, methanol, RT, 1 h.

9-((3a*R*,4*R*,6*R*,6a*R*)-6-(((1,3,2-Dithiaphospholan-2-yl)oxy)methyl)-2,2-dimethyltetrahydrofuro[3,4-*d*][1,3]dioxol-4-yl)-9*H*-purin-6-amine (**18a**)

18a was synthesized following **General procedure A**.

^{31}P NMR (162 MHz, CDCl$_3$): δ 148.54.

2-(((3a*R*,4*R*,6*R*,6a*R*)-6-(6-Amino-9*H*-purin-9-yl)-2,2-dimethyltetrahydrofuro[3,4-*d*][1,3]dioxol-4-yl)methoxy)-1,3,2-dithiaphospholane 2-sulfide (**18b**)

18b was synthesized following **General procedure B**. Yield 44%.

^{1}H NMR (400 MHz, CDCl$_3$): δ 8.37 (s, 1H), 8.07 (s, 1H), 6.66 (s, 2H), 6.20 (s, 1H), 5.48 (s, 1H), 5.10 (s, 1H), 4.60 (s, 1H), 4.31 (s, 2H), 3.59–3.45 (m, 4H), 1.62 (s, 3H), 1.41 (s, 3H).

^{13}C NMR (101 MHz, CDCl$_3$): δ 155.41 (s), 152.59 (s), 149.18 (s), 139.74 (s), 119.89 (s), 114.40 (s), 91.44 (s), 85.57 (d, J = 9.8 Hz), 84.44 (s), 81.66 (s), 67.12 (s), 41.67 (s), 27.10 (s), 25.39 (s), 14.97 (s).

^{31}P NMR (162 MHz, CDCl$_3$): δ 121.34.

O-(((3a*R*,4*R*,6*R*,6a*R*)-6-(6-Amino-9*H*-purin-9-yl)-2,2-dimethyltetrahydrofuro[3,4-*d*][1,3]dioxol-4-yl)methyl) phosphorofluoridodithioate (**18c**)

18c was synthesized following **General procedure C**. Yield 57%.

^{1}H NMR (400 MHz, DMSO-*d*$_6$): δ 7.45 (dd, J = 200.4, 29.1 Hz, 4H), 4.39–4.25 (m, 1H), 3.72 (s, 2H), 3.35 (s, 3H), 3.35–3.25 (m, 8H), 1.71 (dt, J = 16.2, 8.3 Hz, 8H), 1.54–1.33 (m, 17H), 1.06 (t, J = 7.1 Hz, 12H).

O-(((2*S*,3*R*,4*S*,5*S*)-5-(6-Amino-9*H*-purin-9-yl)-3,4-dihydroxytetrahydrofuran-2-yl)methyl) phosphorofluoridodithioate (FTP-AMP, **18**)

5 mL TFA was added into a solution of **18c** (77 mg, 0.12 mmol) in 5.0 mL MeOH and the mixture was stirred at RT for 1 h. The crude product was purified by column chromatography on silica gel to give **18**. Yield 90%. The product often contains trifluoroacetate and it is difficult to remove cleanly.

^{1}H NMR (400 MHz, DMSO-*d*$_6$): δ 8.71 (s, 1H), 8.30 (s, 1H), 7.93 (s, 2H), 5.99 (s, 1H), 4.68–4.56 (m, 1H), 4.28–4.02 (m, 5H), 3.45 (d, J = 14.4 Hz, 1H).

^{19}F NMR (376 MHz, DMSO-*d*$_6$): δ -6.77 (d, J = 1097.92 Hz).

^{31}P NMR (162 MHz, DMSO-*d*$_6$): δ 114.69 (d, J = 1106.46 Hz).

3 Reaction pathways and free-energy profiles

DFT calculations were performed to simulate the nucleophilic substitution mechanisms possibly operating in the ^{18}F-labeling processes and probe the stabilities of flourophosphoric mimics in aqueous solution. Detailed reaction mechanisms and free-energy profiles for both F^- and OH^- substitutions (i.e., hydrolysis) are depicted, with the use of precursors **1b** and **2b** as two models.

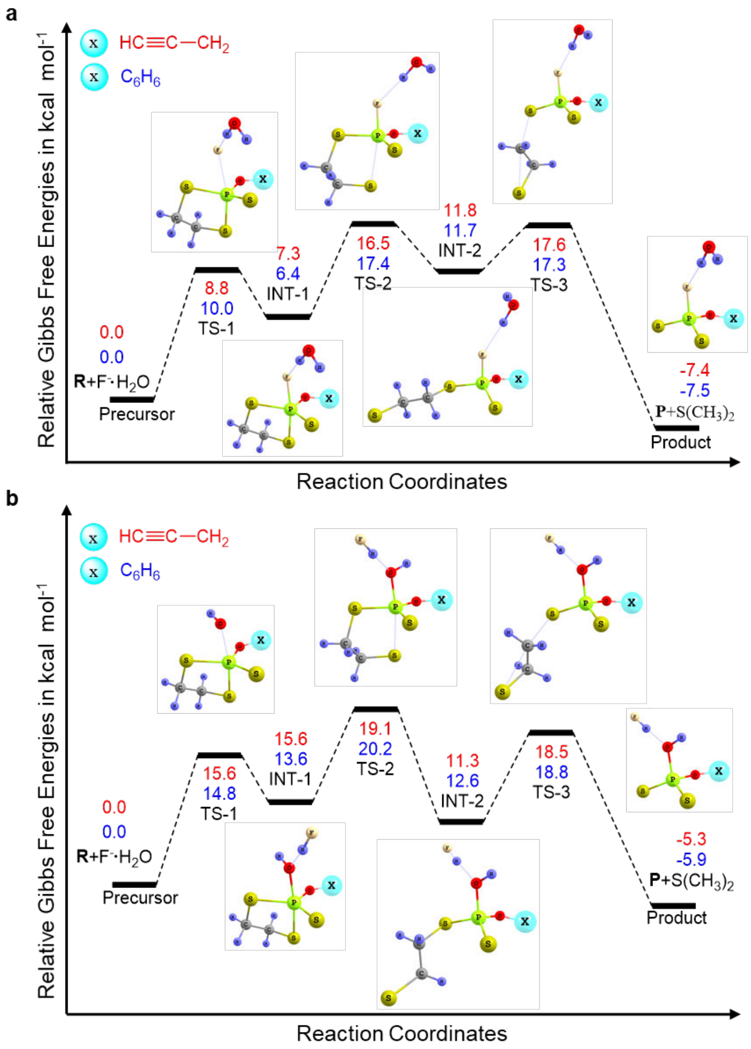

Figure S1. Reaction pathways and free-energy (kcal mol^{-1}) profiles. Geometries are optimized at B3LYP/6-31+G* level of theory, and single point calculations are performed at CAM-B3LYP/6-311++G** level of theory. (**a**) Reaction processes and free-energy (kcal mol^{-1}) profiles for F^- nucleophilic substitution on two

oxydithiaphospholane 2-sulfide precursors **1b** and **2b**. (**b**) Reaction pathways and free-energy (kcal mol^{-1}) profiles for the OH^- nucleophilic substitution on two oxydithiaphospholane 2-sulfide precursors **1b** and **2b**, whose energy barriers are higher than the F^- nucleophilic substitution.

Depending on the B3LYP/6-311+G* computational data, the three-step addition-elimination pathway was identified as the plausible reaction channel for both F^- and OH^- substitutions. The first step is the nucleophilic bonding of $[F\cdots H_2O]^-$ with the reactant phosphorus atom, passing over the transition state **TS-1** to produce the pentacoordinate intermediate **INT-1**. In this process, F^- substitution forms a P–F bond while the possibly competing hydrolysis forms a P–O bond. The second step is the cleavage of the *trans* P–S bond, which has the transition state **TS-2** and recovers the tetracoordinate phosphorus structure in **INT-2**. The final step is an intramolecular substitution to remove a molecule of $S(CH_3)_2$ *via* the transition state **TS-3**, which would yield the product **P** of F^- substitution or hydrolysis.

The corresponding hydrolysis processes on **1b** and **2b** need to surmount the rate-determining barrier heights of up to 19.1 and 20.2 kcal mol^{-1}, respectively. These results suggest that the F^- substitution processes are kinetically favorable while the hydrolysis processes are inaccessible at RT, excluding hydrolysis as a competitive reaction pathway kinetically.

Additionally, F^- substitution products should be more stable than hydrolysis products in aqueous solutions in terms of free-energy changes, because the former processes are 2–3 kcal mol^{-1} more exothermal in free-energy, though the latter processes are also thermodynamically spontaneous.

Overall, DFT calculations indicate that F^- substitution should be more competitive both kinetically and thermodynamically than its opponent, OH^- substitution. It is the theoretical basis of fluorination in wet solvents.

Computational details

All calculations were carried out under the Gaussian 09 computational programme.[5]

Geometries were optimized using the B3LYP method, with the 6-311+G* basis set used for all elements.[6] Solvation effect was treated by the default PCM method.[7] Frequency analyses were performed at the same level of theory. For Gibbs free-energy calculations, some modifications were made. The contributions of small vibrational frequencies (< 100 cm^{-1}) to entropies were corrected on the basis of Grimme's quasi-RRHO approach.[8] The default translational entropies obtained from the Gaussian 09 program are ideal-gas-phase translational entropies, which would exaggerate the entropy decrease of bimolecular reactions in solution.[9] In the present study, the calculated entropy changes were scaled by a factor of 0.5 to compute more accurate free-energy data.[10]

4 Fluorination kinetics

4.1 Predicted fluorination kinetics by DFT

The calculated free-energy profiles show that the F^- substitution processes on **1b** and **2b** have the rate-determining free-energy barriers of 19.8 and 20.4 kcal mol^{-1}, respectively, corresponding to the rate constants of 1.87×10^{-2} M^{-1} s^{-1} and 6.80×10^{-3} M^{-1} s^{-1} and half-life times of 0.90 and 2.45 min at RT, respectively.[11]

$$k = \frac{k_B T}{h}(C^{\theta})^{1-n}\exp(-\frac{\Delta G^{\neq}}{RT})$$

$$t_{1/2} = \frac{1}{c_{A,0} \times K}$$

4.2 Fast fluorination measured by ^{31}P NMR

The fluorination reaction was initially monitored by ^{31}P NMR spectroscopy that would distinguish the precursors and FTPs at the same time. Precursors **1b** and **2b** were used as substrates to explore the ^{19}F-fluorination efficiency. **1b** or **2b** (0.10 mmol) was dissolved in THF (0.4 mL) in NMR sample tubes. 0.1 mL TBAF (1.0 M in THF) was added to the NMR sample tube, and the kinetics was detected by NMR after being inverted and mixed. Surprisingly, the reaction was completed in less than 2 min. Therefore, we abandoned NMR to measure this too fast rate constant.

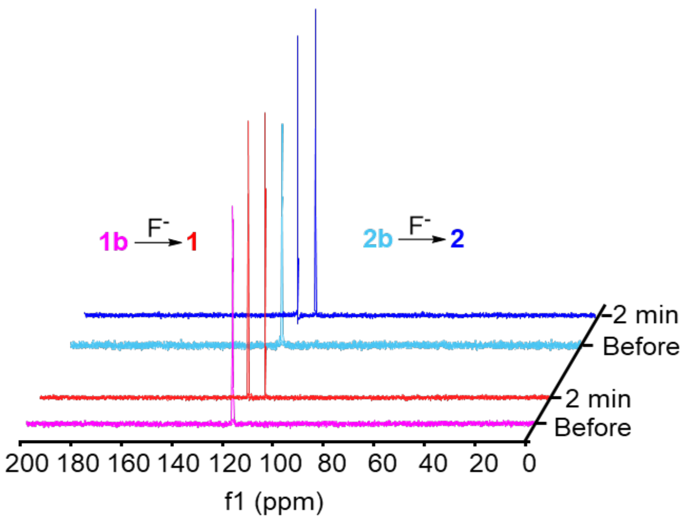

Figure S2. ^{31}P NMR spectra detection of fluorination process from precursors to fluorinated products.

4.3 Experimental fluorination rates

In order to determine the fluorination kinetics and activation energy (E_a) for ^{18}F-fluorination process, we decided to carry out a series of experiments at different temperatures. We found experimentally that the optimal temperature range to monitor the labeling reaction rate of [^{18}F]**1** and [^{18}F]**2** is between -55 °C and -25 °C, since the reaction is too fast at higher temperatures and too slow at lower temperatures to measure. Concentrations of precursors and in DMF solutions were kept constant at 3.82 × 10^{-3} M and 4.01 × 10^{-3} M, respectively. The RCCs under each individual temperature was monitored by radio-TLC. Dynamic RCCs were able to be monitored by TLC because we took 10 μL of the reaction mixture at the indicated time points, quenched it in 1.0 mL of water before TLC developing. Labeling efficiency graphs could be converted to line graphs of reaction time versus ln [1/(1-RCC)], whose slopes represent specific rate constants at specific temperatures. Pseudo-first order initial rate constants at different temperatures were calculated from the exponential fit equation (for ^{18}F-labeling reaction of [^{18}F]**1**: k'_{223} = 3.26 × 10^{-2} s^{-1}; k'_{228} = 9.80 × 10^{-2} s^{-1}; k'_{233} = 13.35 × 10^{-2} s^{-1}; k'_{243} = 23.38 × 10^{-2} s^{-1}; k'_{248} = 26.50 × 10^{-2} s^{-1}; ^{18}F-labeling reaction of [^{18}F]**2**: k'_{218} = 2.36 × 10^{-2} s^{-1}; k'_{223} = 3.44 × 10^{-2} s^{-1}; k'_{228} = 7.89 × 10^{-2} s^{-1};

k'_{240} = 19.14 × 10^{-2} s^{-1}; k'_{248} = 26.48 × 10^{-2} s^{-1}); and then k' was divided by the concentration of **1b** or **2b** to determine the actual second-order rate constants. For ^{18}F-labeling reaction of [^{18}F]**1**: k_{223} = 8.53 L mol^{-1} s^{-1}; k_{228} =25,66 L mol^{-1} s^{-1}; k_{233} = 34.93 L mol^{-1} s^{-1}; k_{243} = 61.20 L mol^{-1} s^{-1}; k_{248} = 69.38 L mol^{-1} s^{-1}; for ^{18}F-labeling reaction of [^{18}F]**2**: k_{218} = 5.91 L mol^{-1} s^{-1}; k_{223} = 8.60 L mol^{-1} s^{-1}; k_{228} = 19.72 L mol^{-1} s^{-1}; k_{240} = 47.85 L mol^{-1} s^{-1}; k_{248} = 66.20 L mol^{-1} s^{-1}. These rate constants were used to create an Arrhenius plot (ln k versus T^{-1}) to calculate the E_a, which was found to be 8.41 kcal mol^{-1} for ^{18}F-labeling reaction of [^{18}F]**1** and 8.98 kcal mol^{-1} for ^{18}F-labeling reaction of [^{18}F]**2**.

5 Radiosynthesis of ^{18}F-labeled FTPs

5.1 General manual ^{18}F-labeling procedures

Labeling procedure I: [^{18}F]F^{-} was azeotropically dried as previously described.[12] Briefly, [^{18}F]F^{-} was produced *via* the ^{18}O(p, n)^{18}F reaction and delivered as [^{18}F]F^{-} in [^{18}O]H_2O. [^{18}F]F^{-} (0.185–1.85 GBq) was separated from ^{18}O-enriched-water using QMA and subsequently released with by a solution of 8.0 mg kryptofix 222 (K_{222}) and 1.0 mg K_2CO_3 in 1.0 mL of acetonitrile/H_2O (4/1, v/v). The solution was azeotropically dried for three times (300 µL acetonitrile × 3) at 100 °C in a clean glass vial. 0.2 µmol precursor was dissolved in 100 µL acetonitrile (anhydrous or non-anhydrous) and added into the glass vial containing [^{18}F]F^{-}. The mixture was incubated at RT for 30 sec before quenching by 500 µL water. RCCs were analyzed by radio-HPLC or radio-TLC (n = 3).

Labeling procedure II (with wet [^{18}F]F^{-}): 0.2 µmol precursor was dissolved in 50 µL acetonitrile. Aqueous [^{18}F]F^{-} solution (diluted from target water, no K_{222} and K_2CO_3 added, 0.185–1.85 GBq) in appropriate volume was added into the solution. Then total reaction volume was adjusted to 100 µL with pure water and acetonitrile. The reaction vial was gently shaken at RT for 30 sec before quenching by adding 500 µL water. RCCs were analyzed by radio-HPLC or radio-TLC (n = 3).

5.2 Optimization of ^{18}F-labeling conditions

5.2.1 Effect of labeling time

^{18}F-labeling of [^{18}F]**1** and [^{18}F]**2** was performed following **Labeling procedure I** except that the reactions were quenched after different time duration.

Table S1. RCCs of [^{18}F]**1** and [^{18}F]**2** at different reaction time points.

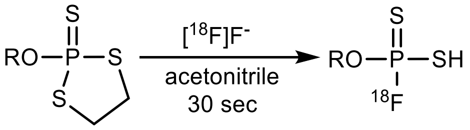

R = but-3-yn-1-yl [^{18}F]**1**
phenoxy [^{18}F]**2**

Time (sec)	RCC (%) of [^{18}F]**1** [a]	RCC (%) of [^{18}F]**2** [a]
10	93 ± 5	97 ± 2
30	94 ± 3	97 ± 1
60	94 ± 2	98 ± 3
120	94 ± 3	98 ± 1
300	97 ± 1	98 ± 2

[a] RCCs determined by radio-TLC analysis (n = 3).

5.2.2 Effect of precursor loads

The reaction was run under the same conditions as **Labeling procedure I** except that different precursor loads were performed.

Table S2. RCCs of [^{18}F]**1** and [^{18}F]**2** at different precursor loads.

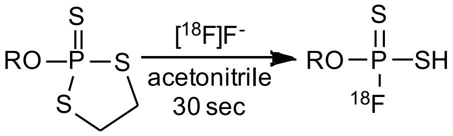

R = but-3-yn-1-yl [^{18}F]**1**
phenyl [^{18}F]**2**

Precursor load (mg)	Precursor load (μmol)	RCC (%) of [^{18}F]**1** [a]	Precursor load (μmol)	RCC (%) of [^{18}F]**2** [a]
0.01	0.04	52 ± 6	0.04	90 ± 7
0.05	0.22	95 ± 9	0.20	94 ± 3
0.1	0.45	98 ± 1	0.40	97 ± 5
0.5	2.23	97 ± 2	2.02	87 ± 6
1	4.46	84 ± 8	4.03	87 ± 8

[a] RCCs determined by radio-TLC analysis (n = 3).

5.2.3 Effect of temperatures and solvent water contents

Effect of temperatures and solvent water contents on ^{18}F-labeling RCCs have been examined. Reactions were run following **Labeling procedure II** except that different temperatures or solvent water ratio were set.

Table S3. RCCs of [^{18}F]**1** at different temperatures and solvent water ratios.

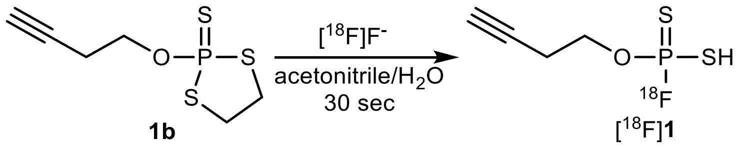

T (°C)	Solvent water ratio (%) [a]	RCC (%) of [^{18}F]**1** [b]
RT	0	97 ± 3
RT	10	48 ± 5
RT	20	12 ± 2
RT	25	2 ± 1
RT	34	2 ± 1
RT	50	0
50	0	98 ± 1
50	10	93 ± 4
50	20	9 ± 7
50	25	3 ± 1
50	34	1 ± 1
50	50	0
80	0	98 ± 2
80	10	96 ± 2
80	20	23 ± 3
80	25	6 ± 4
80	34	3 ± 2
80	50	2 ± 1

[a] The solution is a mixture of acetonitrile and water,100 μL. [b] RCCs determined by radio-TLC analysis.

Table S4. RCCs of [^{18}F]**2** at different temperatures and solvent water ratios.

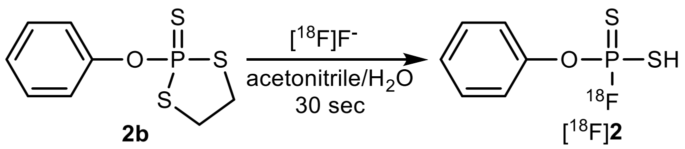

T (°C)	Solvent water ratio (%) [a]	RCC (%) of [^{18}F]**2** [b]
RT	0	97 ± 3
RT	10	10 ± 3
RT	20	0
RT	25	0
RT	34	0
RT	50	0
50	0	98 ± 1
50	10	20 ± 4
50	20	0
50	25	0
50	34	0
50	50	0
80	0	99 ± 2
80	10	40 ± 5
80	20	10 ± 4
80	25	0
80	34	0
80	50	0

[a] The solution is a mixture of acetonitrile and water, 100 μL. [b] RCCs determined by radio-TLC analysis.

5.2.4 Effect of solvents

The reaction was run under the same conditions as **Labeling procedure I** except those different solvents were applied.

Table S5. RCCs of [^{18}F]**1** and [^{18}F]**2** in different solvents.

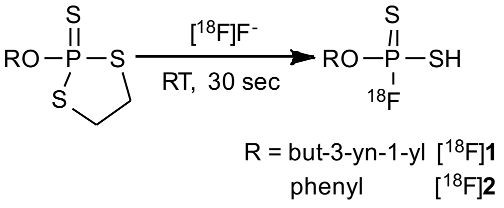

Solvent	RCC (%) of [^{18}F]**1** [a]	RCC (%) of [^{18}F]**2** [a]
DMF	97 ± 1	98 ± 3
DMSO	98 ± 1	96 ± 2
THF	96 ± 2	96 ± 3
CH3CN	98 ± 1	97 ± 1
MeOH	6 ± 2	0

[a] RCCs determined by radio-TLC analysis.

5.3 Identification of [^{18}F]1, [^{18}F]2, [^{18}F]5–[^{18}F]13, [^{18}F]16

[^{18}F]**1**, [^{18}F]**2**, [^{18}F]**5**–[^{18}F]**13**, and [^{18}F]**16** were labeled under the same conditions as **Labeling procedure I** or **Labeling procedure II**.

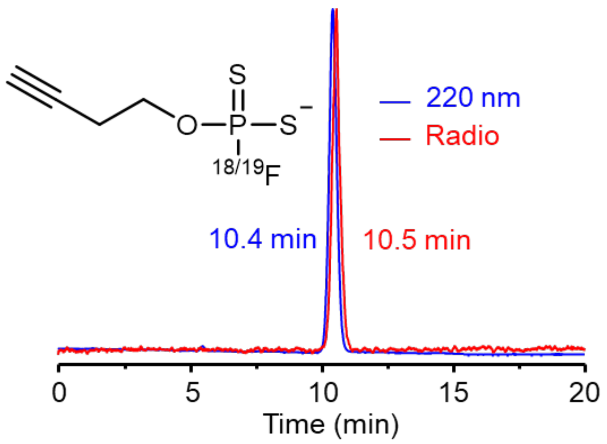

Figure S3. Radio-HPLC chromatogram of co-injection of **1** and [^{18}F]**1**. HPLC condition: Nacalai Tesque Cosmosil 5C18-MS-II column (4.4 µm, 4.6 mm × 250 mm, Japan). Phase A: PBS (0.01 mol L^{-1} pH = 7.4); phase B: HPLC grade acetonitrile; isocratic elution at 85% phase A and 15% phase B. Flow rate: 1.0 mL min^{-1}.

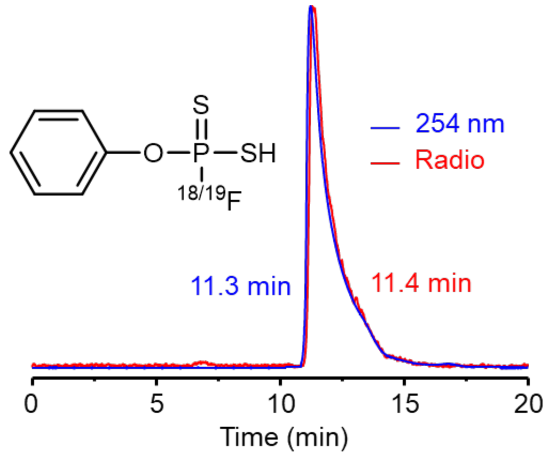

Figure S4. Radio-HPLC chromatogram of co-injection of **2** and [^{18}F]**2**. HPLC condition: Nacalai Tesque Cosmosil 5C18-MS-II column (4.4 μm, 4.6 mm × 250 mm, Japan). Phase A: PBS (0.01 mol L^{-1} pH = 7.4); phase B: HPLC grade acetonitrile; isocratic elution at 75% phase A and 25% phase B. Flow rate: 1.0 mL min^{-1}.

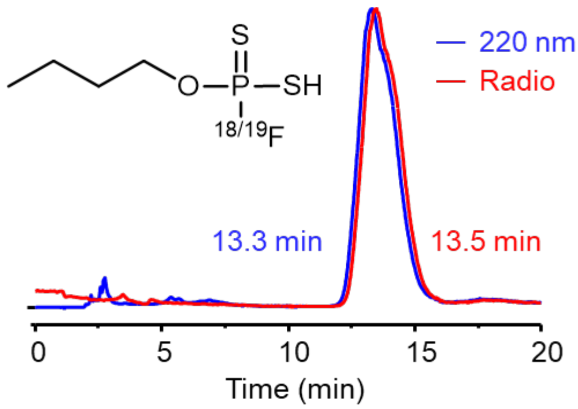

Figure S5. Radio-HPLC chromatogram of co-injection of **5** and [^{18}F]**5**. HPLC condition: Nacalai Tesque Cosmosil 5C18-MS-II column (4.4 μm, 4.6 mm × 250 mm, Japan). Phase A: PBS (0.01 mol L^{-1} pH = 7.4); phase B: HPLC grade acetonitrile; isocratic elution at 75% phase A and 25% phase B. Flow rate: 1.0 mL min^{-1}.

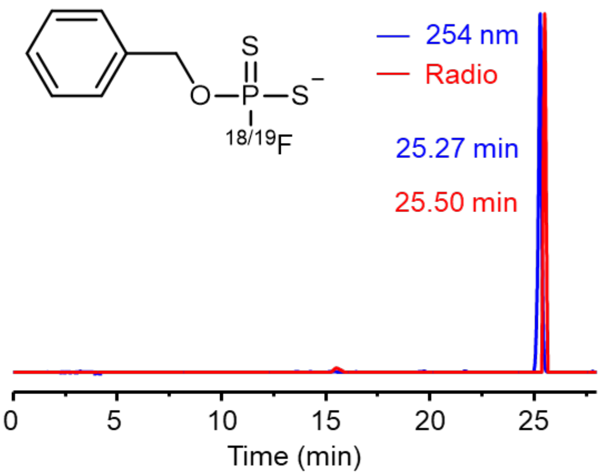

Figure S6. Radio-HPLC chromatogram of co-injection of **6** and [^{18}F]**6**. HPLC condition: Nacalai Tesque Cosmosil 5C18-MS-II column (4.4 μm, 4.6 mm × 250 mm, Japan). Phase A: PBS (0.01 mol L^{-1} pH = 7.4); phase B: HPLC grade acetonitrile; gradient elution: 0–10 min (10% acetonitrile to 15% acetonitrile) linear increase; 10–30 min (15% acetonitrile to 40% acetonitrile) linear increase. Flow rate: 1.0 mL min^{-1}.

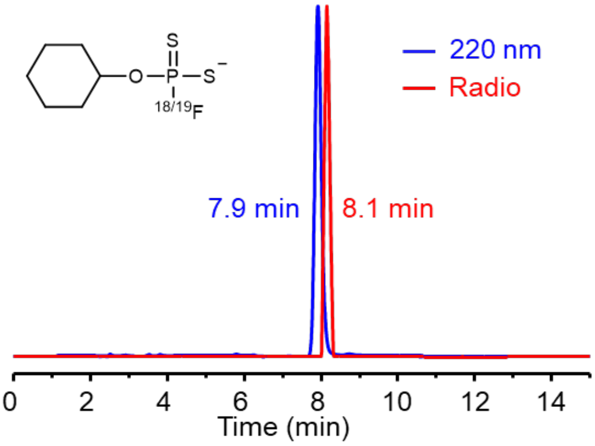

Figure S7. Radio-HPLC chromatogram of co-injection of **7** and [^{18}F]**7**. HPLC condition: Nacalai Tesque Cosmosil 5C18-MS-II column (4.4 μm, 4.6 mm × 250 mm, Japan). Phase A: PBS (0.01 mol L^{-1} pH = 7.4); phase B: HPLC grade acetonitrile; isocratic elution at 75% phase A and 25% phase B. Flow rate: 1.0 mL min^{-1}.

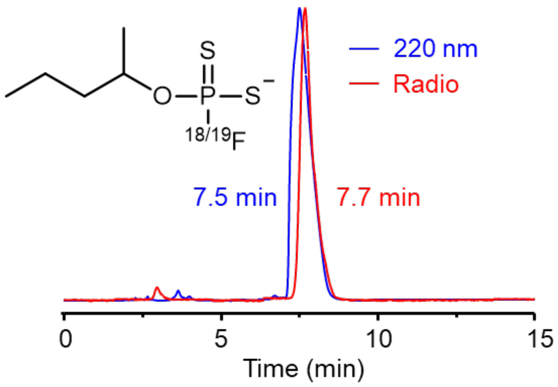

Figure S8. Radio-HPLC chromatogram of co-injection of **8** and [^{18}F]**8**. HPLC condition: Nacalai Tesque Cosmosil 5C18-MS-II column (4.4 μm, 4.6 mm × 250 mm, Japan). Phase A: PBS (0.01 mol L^{-1} pH = 7.4); phase B: HPLC grade acetonitrile; isocratic elution at 75% phase A and 25% phase B. Flow rate: 1.0 mL min^{-1}.

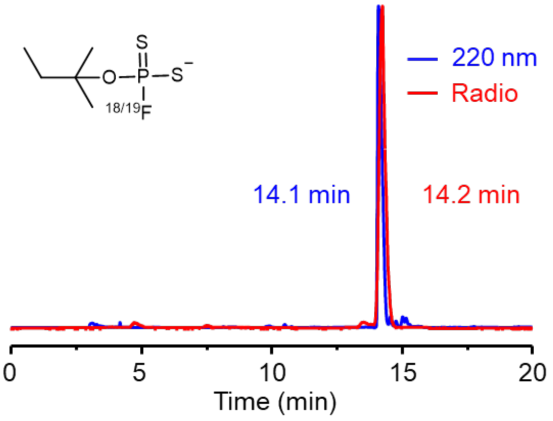

Figure S9. Radio-HPLC chromatogram of co-injection of **9** and [^{18}F]**9**. HPLC condition: Nacalai Tesque Cosmosil 5C18-MS-II column (4.4 μm, 4.6 mm × 250 mm, Japan). Phase A: PBS (0.01 mol L^{-1} pH = 7.4); phase B: HPLC grade acetonitrile; isocratic elution at 85% phase A and 15% phase B. Flow rate: 1.0 mL min^{-1}.

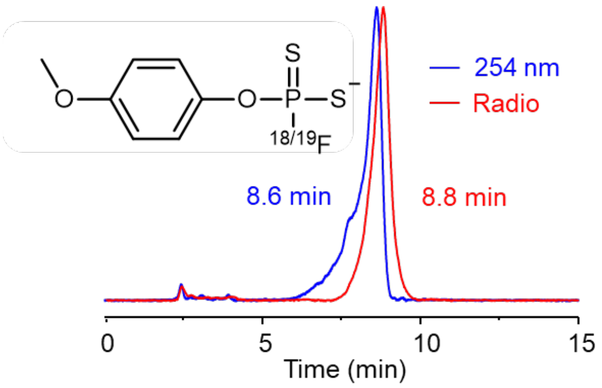

Figure S10. Radio-HPLC chromatogram of co-injection of **10** and [^{18}F]**10**. HPLC condition: Nacalai Tesque Cosmosil 5C18-MS-II column (4.4 µm, 4.6 mm × 250 mm, Japan). Phase A: PBS (0.01 mol L^{-1} pH = 7.4); phase B: HPLC grade acetonitrile; isocratic elution at 75% phase A and 25% phase B. Flow rate: 1.0 mL min^{-1}.

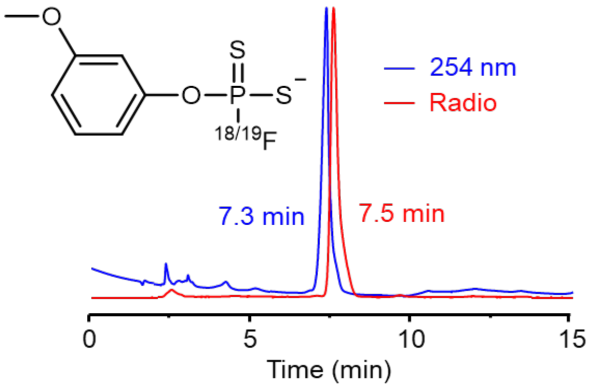

Figure S11. Radio-HPLC chromatogram of co-injection of **11** and [^{18}F]**11**. HPLC condition: Nacalai Tesque Cosmosil 5C18-MS-II column (4.4 µm, 4.6 mm × 250 mm, Japan). Phase A: PBS (0.01 mol L^{-1} pH = 7.4); phase B: HPLC grade acetonitrile; isocratic elution at 75% phase A and 25% phase B. Flow rate: 1.0 mL min^{-1}.

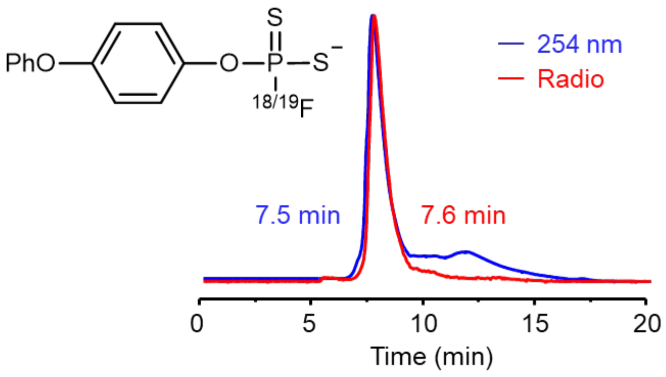

Figure S12. Radio-HPLC chromatogram of co-injection of **12** and [^{18}F]**12**. HPLC condition: Nacalai Tesque Cosmosil 5C18-MS-II column (4.4 µm, 4.6 mm × 250 mm, Japan). Phase A: PBS (0.01 mol L^{-1} pH = 7.4); phase B: HPLC grade acetonitrile; isocratic elution at 50% phase A and 50% phase B. Flow rate: 1.0 mL min^{-1}.

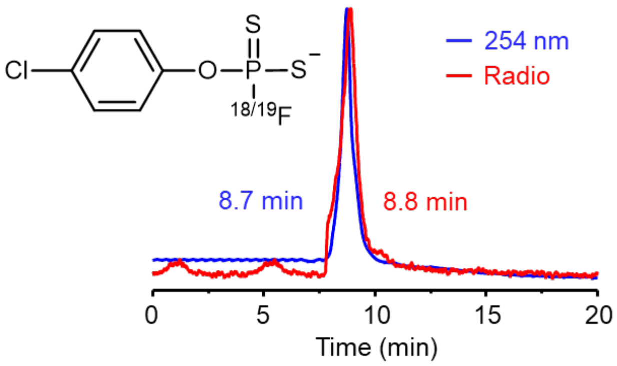

Figure S13. Radio-HPLC chromatogram of **13** and [^{18}F]**13**. HPLC condition: Nacalai Tesque Cosmosil 5C18-MS-II column (4.4 µm, 4.6 mm × 250 mm, Japan). Phase A: PBS (0.01 mol L^{-1} pH = 7.4); phase B: HPLC grade acetonitrile; isocratic elution at 75% phase A and 25% phase B. Flow rate: 1.0 mL min^{-1}.

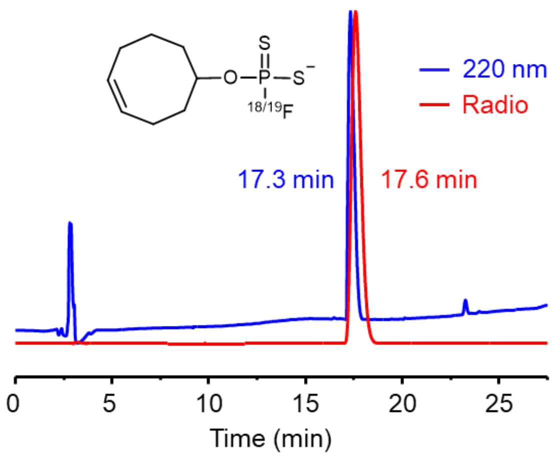

Figure S14. Radio-HPLC chromatogram of co-injection of **16** and [^{18}F]**16**. HPLC condition: Nacalai Tesque Cosmosil 5C18-MS-II column (4.4 µm, 4.6 mm × 250 mm, Japan). Phase A: PBS (0.01 mol L^{-1} pH = 7.4); phase B: HPLC grade acetonitrile; gradient elution: 0-30 min (10% acetonitrile to 95% acetonitrile) linear increase. Flow rate: 1.0 mL min^{-1}.

5.4 Radiosynthesis of ^{18}F-labeled *O*-(but-3-yn-1-yl) phosphorofluoridothioate ([^{18}F]3) as a radiosynthon

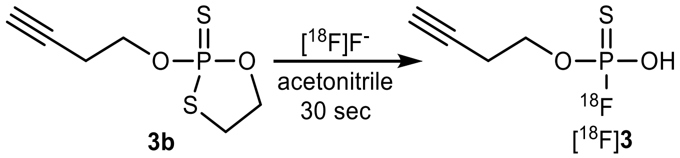

Scheme S7. Radiosynthetic route of [^{18}F]**3**.

The reaction was run under the same conditions as **Labeling procedure II**.

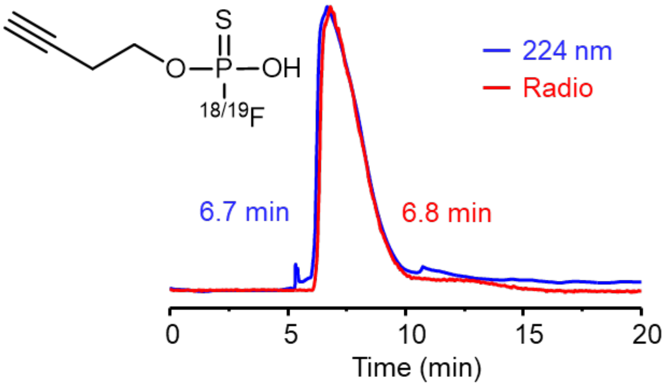

Figure S15. Radio-HPLC chromatogram of co-injection of **3** and [^{18}F]**3**. HPLC condition: Nacalai Tesque Cosmosil 5C18-MS-II column (4.4 μm, 4.6 mm × 250 mm, Japan). Phase A: PBS (0.01 mol L^{-1} pH = 7.4); phase B: HPLC grade acetonitrile; isocratic elution at 90% phase A and 10% phase B. Flow rate: 1.0 mL min^{-1}.

5.5 Radiosynthesis of *O*-(but-3-yn-1-yl) phosphorofluoridodithioate ([^{18}F]4)

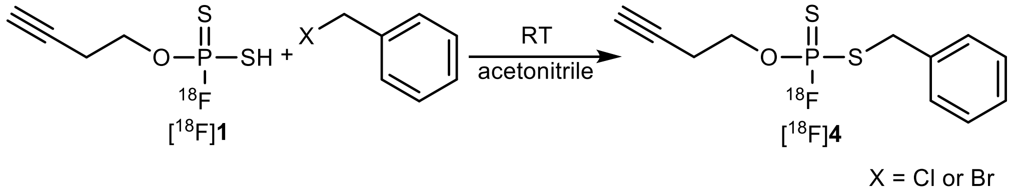

Scheme S8. Radiosynthetic route of [^{18}F]**4**.

^{18}F-labeled *S*-benzyl *O*-(but-3-yn-1-yl) phosphorofluoridodithioate ([^{18}F]**4**) was used as an example to study the stabilities when the coulombic repulsion was missing by blocking S^{-} and to investigate the conjugation efficiency between a ^{18}F-labeled FTP synthon and a halide.

A solution of benzyl halide (5 μmol) in acetonitrile (100 μL) was added dropwise into the solution of [^{18}F]**1**, and the mixture was stirred at RT for indicated time. The reaction mixture was quenched by water (500 μL). RCCs were determined by radio-TLC analysis of the crude product. The crude product was purified by radio-HPLC. Purified product was identified by LC-MS.

MS (ESI) calculated for $C_{11}H_{13}FOPS_2^+$ ($[M+H]^+$): requires 274.0, found 274.2.

Table S6. Total RCCs of [^{18}F]**4** at different reaction time points.

X	Time (min)	RCC (%) [a]
Cl	5	23 ± 2
Cl	15	24 ± 5
Cl	30	38 ± 8
Br	5	93 ± 4
Br	15	91 ± 8
Br	30	94 ± 5

[a] RCCs determined by radio-HPLC analysis of the crude product (n = 3).

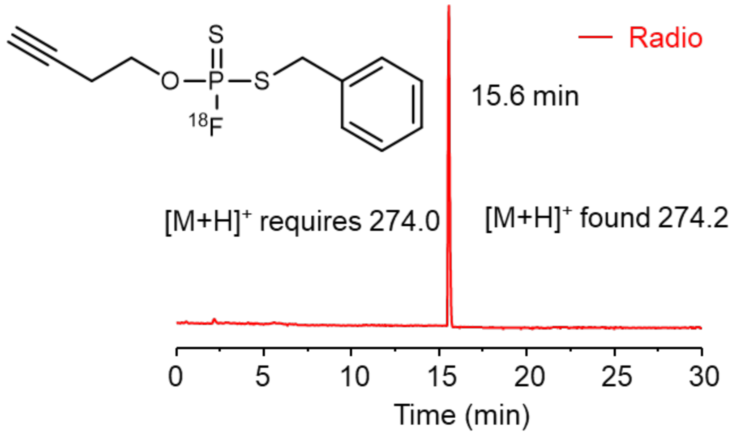

Figure S16. Radio-HPLC chromatogram of [^{18}F]**4**. HPLC condition: Nacalai Tesque Cosmosil 5C18-MS-II column (4.4 µm, 4.6 mm × 250 mm, Japan). Phase A: H_2O; phase B: HPLC grade acetonitrile; isocratic elution at 40% phase A and 60% phase B. Flow rate: 1.0 mL min^{-1}.

5.6 Radiosynthesis of ^{18}F-labeled FTPs as PET tracers

5.6.1 Radiosynthesis of [^{18}F]FTP-Tyr

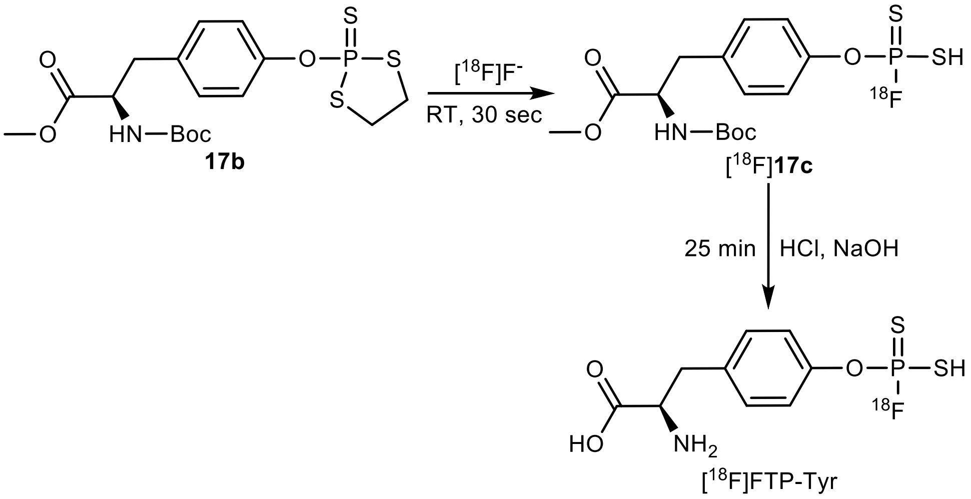

Scheme S9. Radiosynthetic route of [^{18}F]FTP-Tyr.

[^{18}F]**17c** was synthesized from **17b** following **labeling procedure I** as described in section **5.1**. [^{18}F]**17c** was then dissolved in 100 µL MeOH followed by adding 100 µL NaOH (2.0 M) for 15 min. The reaction mixture was acidified by 1.0 M HCl and was dried under a stream of nitrogen. The resulting residue was dissolved in acetonitrile (100 µL). 5.0 M HCl or TFA (100 µL) was added and shaken at RT for 10 min. The solution was then neutralized to pH 7 by adding NaOH (1.0 M) and diluted with PBS and acetonitrile before being analyzed and purified on HPLC. The collected product was then put on the rotary evaporator to remove excess methanol from the solution. [^{18}F]FTP-Tyr was dissolved in saline for PET imaging studies.

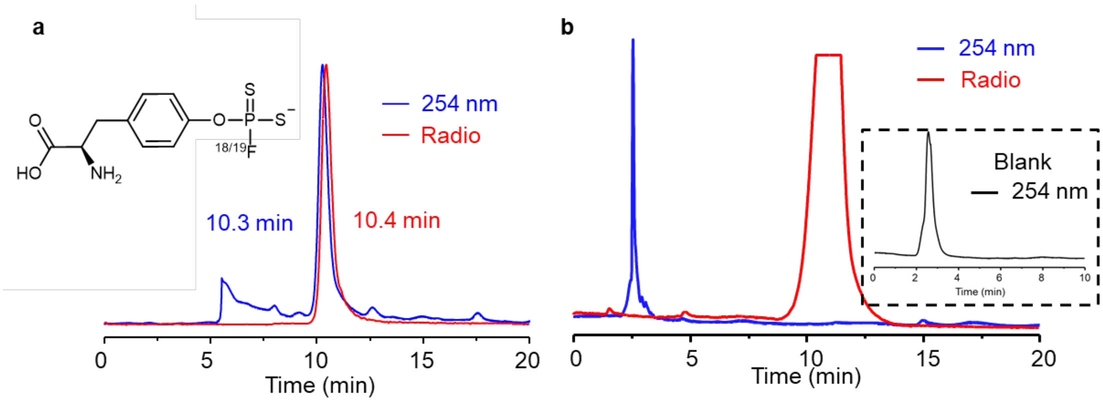

Figure S17. (**a**) Radio-HPLC chromatogram of co-injection of FTP-Tyr and [^{18}F]FTP-Tyr (**17** and [^{18}F]**17**). (**b**) HPLC chromatogram (UV and radio) of purified

[^{18}F]FTP-Tyr. HPLC condition: Nacalai Tesque Cosmosil 5C18-MS-II column (4.4 μm, 4.6 mm × 250 mm, Japan). Phase A: PBS (0.01 mol L^{-1} pH = 7.4); phase B: HPLC grade methanol; isocratic elution at 90% phase A and 10% phase B. Flow rate: 1.0 mL min^{-1}.

5.6.2 Radiosynthesis of [^{18}F]FTP-AMP

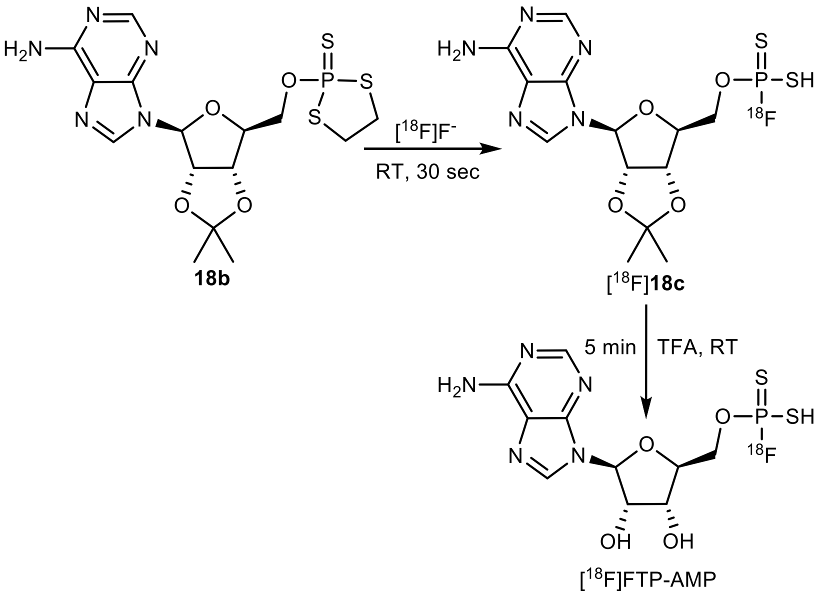

Scheme S10. Radiosynthetic route of [^{18}F]FTP-AMP.

[^{18}F]**18c** was synthesized from **18b** following **labeling procedure I** as described in section **5.1**. Then 100 μL TFA was added to [^{18}F]**18c** and stirred at RT for 5 min to deprotection. The solution was then neutralized to pH 7 by adding NaOH (1.0 M) and diluted with PBS and acetonitrile before being analyzed and purified on radio-HPLC. The collected product was then put on the rotary evaporator to remove excess acetonitrile from the solution. [^{18}F]FTP-AMP was dissolved in saline for PET imaging studies.

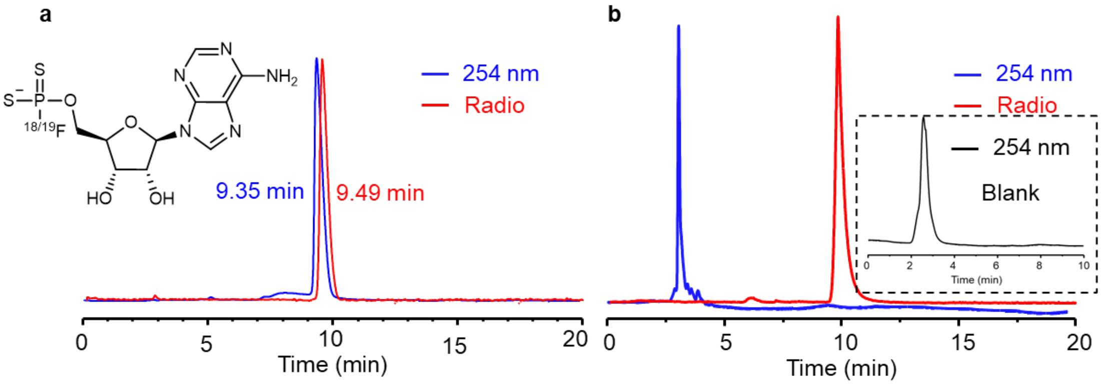

Figure S18. (**a**) Radio-HPLC chromatogram of co-injection of FTP-AMP and [^{18}F] FTP-AMP (**18** and [^{18}F]**18**). (**b**) HPLC chromatogram (UV and radio) of purified [^{18}F]FTP-AMP. HPLC condition: Nacalai Tesque Cosmosil 5C18-MS-II column (4.4 μm, 4.6 mm × 250 mm, Japan). Phase A: PBS (0.01 mol L^{-1} pH = 7.4); phase B: HPLC grade acetonitrile; isocratic elution at 75% phase A and 25% phase B. Flow rate: 1.0 mL min^{-1}.

5.7 Radiosynthesis of ^{18}F-labeled biomolecules *via* ^{18}F-labeled FTP synthons

5.7.1 Radiosynthesis of [^{18}F]FTP-NHS ([^{18}F]15)

Radiosynthesis procedure for [^{18}F]**15** was descried as an example:

1) 0.1 mg precursor **15b** (0.2 μmol) was dissolved in dichloromethane (40 μL) and loaded onto a cotton ball (about 0.03 cm^3). Then the dichloromethane was allowed to volatilize in a fume hood, and the small cotton carrying precursor **15b** was fitted into a pipette tip (for manual labeling, any tube-like part for automatic modules).

2) No-carrier-added [^{18}F]$F^–$ was produced *via* the $^{18}O(p, n)^{18}F$ reaction and delivered as [^{18}F]$F^–$ in [^{18}O]H_2O. [^{18}F]$F^–$ (3 μL, 0.30–0.37 GBq) was added to 100 μL acetonitrile with tetrabutylammonium hydroxide (TBAOH, 0.52 mg/100 μL), which was used as the eluent later.

3) The pipette tip carrying precursor **15b** was eluted by this mixed solution into a polypropylene tube and subsequently dried under a stream of nitrogen to afford [^{18}F]**15** as a radiosynthon.

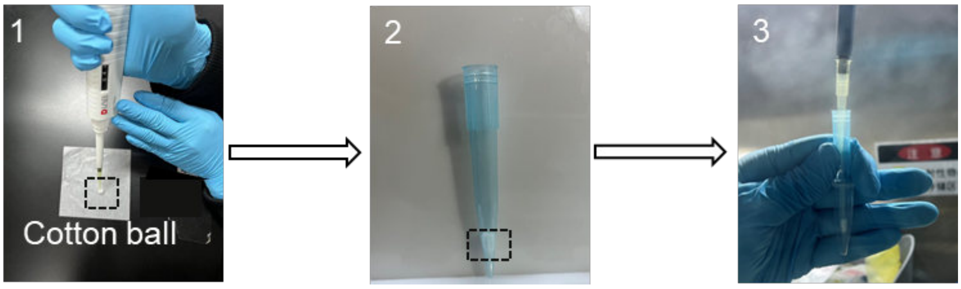

Figure S19. The manual ^{18}F-labeling procedure of [^{18}F]FTP radiosynthon. 1) 0.1 mg precursor **15b** was dissolved in 40 μL dichloromethane and loaded onto a cotton ball (about 0.03 cm^3). 2) The dried small cotton carrying **15b** was fitted into a pipette tip. 3) The precursor cotton was eluted with 100 μL [^{18}F]F$^-$/TBAOH solution.

5.7.2 Radiosynthesis of [^{18}F]FTP-c(RGDyK)

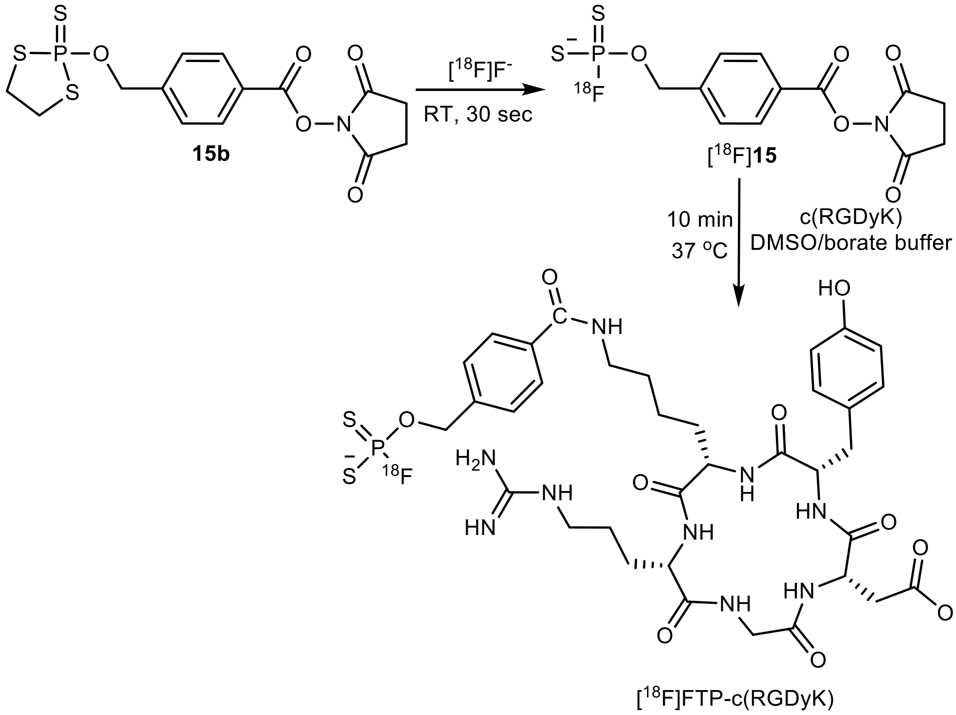

Scheme S11. Radiosynthetic route of [^{18}F]FTP-c(RGDyK).

1.0 mg (1.6 μmol) c(RGDyK) was dissolved in a mixture of 10 μL DMSO and 100 μL of borate buffer (pH = 8.0). This c(RGDyK) solution was then added into the vial containing [^{18}F]**15**. After 10 min reaction at 37 °C, the reaction mixture was diluted with 10.0 mL of H_2O and loaded onto a light C18 cartridge. The cartridge was flushed twice with 10.0 mL of pure water to remove the unreacted [^{18}F]F$^-$ and 1.0 mL of

ethanol to get crude product. The crude product was further purified by radio-HPLC. Purify condition: Waters XBridgeC-18 column (5 µm, 10 mm × 250 mm, USA). Phase A: PBS (0.02 mol L^{-1} pH = 7.4); phase B: HPLC grade acetonitrile; isocratic elution at 90% phase A and 10% phase B. Flow rate: 3.0 mL min^{-1}. The HPLC fraction was dried under a stream of nitrogen at RT. [^{18}F]FTP-c(RGDyK) was then dissolved in saline for injection.

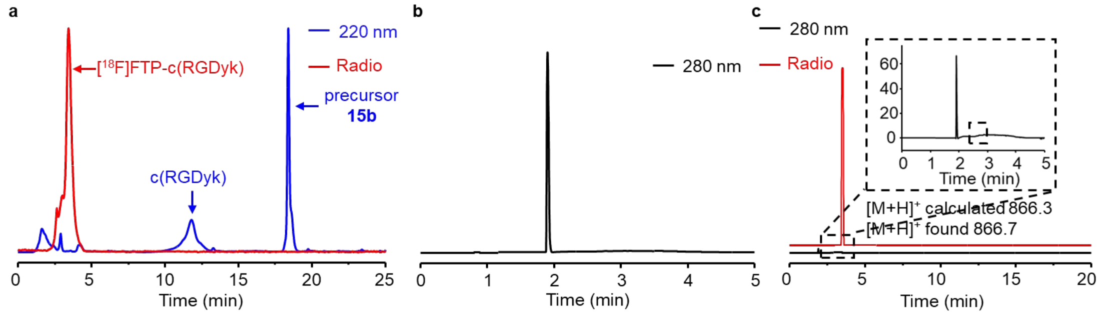

Figure S20. Analytical radio-HPLC chromatogram of [^{18}F]FTP-c(RGDyK). (**a**) Radio-HPLC chromatogram of co-injection of ^{18}F-labeling reaction mixture, c(RGDyK) and **15b**, to illustrate the adequate intervals among the retention times among [^{18}F]FTP-c(RGDyK), c(RGDyK) and **15b**. (**b**) Radio-HPLC chromatogram of a blank injection. (**c**) Radio-HPLC chromatogram of purified [^{18}F]FTP-c(RGDyK), with MS identification. HPLC condition for (**a**): Nacalai Tesque Cosmosil 5C18-MS-II column (4.4 µm, 4.6 mm × 250 mm, Japan). Phase A: PBS (0.02 mol L^{-1} pH = 7.4); phase B: HPLC grade acetonitrile; 0 to 5 min: isocratic elution at 10% phase B, 5 to 30 min: 10% to 95% phase B. Flow rate: 1.0 mL min^{-1}. HPLC condition for (**b**) and (**c**): Nacalai Tesque Cosmosil 5C18-MS-II column (4.4 µm, 4.6 mm × 250 mm, Japan). Phase A: PBS (0.02 mol L^{-1} pH = 7.4); phase B: HPLC grade acetonitrile; isocratic elution at 85% phase A and 15% phase B. Flow rate: 1.0 mL min^{-1}.

5.7.3 Radiosynthesis of [^{18}F]FTP-HSA

1.0 mg of HSA in 0.1 M borate buffer (pH = 8.0, 2 mg mL^{-1}, 100 µL) was added to a glass vial containing dried [^{18}F]**15** and the mixture was incubated for 10 min at 40 °C. [^{18}F]FTP-HSA was purified by a PD-10 column (GE Healthcare Bio-Science AB)

using PBS (pH = 7.4) as the eluent. A size exclusion chromatography (SEC) column was applied to analyze its RCP.

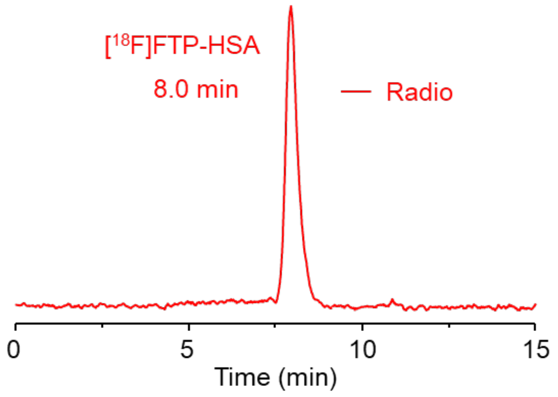

Figure S21. Radio-HPLC chromatogram of purified [^{18}F]FTP-HSA. HPLC condition: Xtimate SEC-300 column (Welch, China); isocratic elution with 100% PBS (0.01 mol L^{-1}, pH = 7.4); flow rate: 1.0 mL min^{-1}.

5.7.4 Radiosynthesis of [^{18}F]FTP-5F7

0.1 mg of 5F7 was dissolved in 100 μL of borate buffer (pH = 8.0) and added to [^{18}F]**15**. The reaction mixture was incubated for 10 min at RT. The product was purified and then analyzed by radio-HPLC equipped with an Xtimate SEC-300 column (Welch, China).

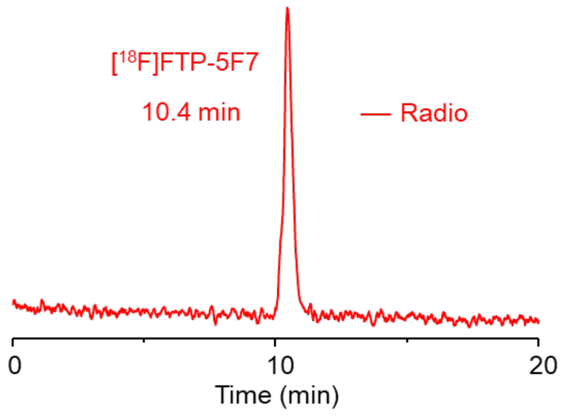

Figure S22. Radio-HPLC chromatogram of purified [^{18}F]FTP-5F7. HPLC condition: Xtimate SEC-300 column (Welch, China); isocratic elution with 100% PBS (0.01 mol L^{-1}, pH = 7.4); flow rate: 1.0 mL min^{-1}.

5.7.5 Radiosynthesis of [^{18}F]FTP-Pds-PEG

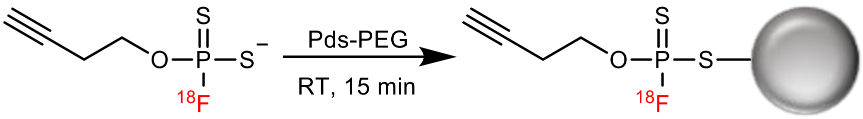

Scheme S12. Radiosynthetic route of [^{18}F]FTP-Pds-PEG.

^{18}F-Labeled FTPs were prepared following the **Labeling procedure I**. Pd nanosheets with an average size of 40 nm (200 μg), as an example of metal nanoparticle, dissolved in thiol-polyethylene glycol (mPEG-SH) solution (2 mg in 100 μL water) to obtain PEGylated Pd nanosheets. Then, [^{18}F]**1** (taking [^{18}F]**1** as an example) was added to Pd-PEG and stirred for 15 min at RT to get [^{18}F]FTP-Pds-PEG. RCCs was determined by radio-TLC analysis. [^{18}F]**1** labeled Pd nanosheets with a high efficiency (about 93%). Then the mixture was subject to ultrafiltration (13000 rpm for 10 min, repeated 3 times) to remove unlabeled [^{18}F]**1** and [^{18}F]F$^-$. RCPs was determined by radio-TLC analysis.

The labeling efficiency of radionuclides can be calculated by radio-TLC. Polyamide film/saline system was used to identify [^{18}F]F$^-$, [^{18}F]**1** and [^{18}F]FTP-Pds-PEG. [^{18}F]FTP-Pds-PEG stayed at the point of origin (Rf: 0–0.1).

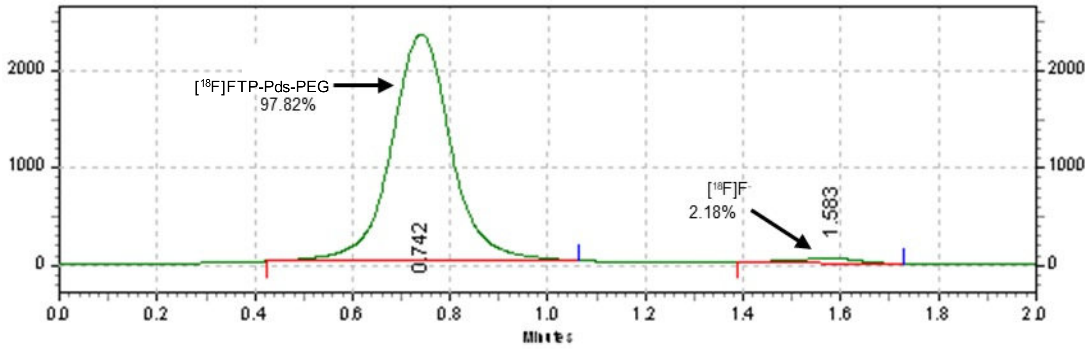

Figure S23. Radio-TLC analysis of RCC of [^{18}F]FTP-Pds-PEG, RCC = 97.8%. Radio-TLC analyses were achieved on polyamide film. Developing solvent of radio-TLC was saline.

6 Automated radiosynthesis and quality control of [^{18}F]FTP-c(RGDyK)

The automated synthesis of [^{18}F]FTP-c(RGDyK) was performed on an AllinOne module (Trasis, Ans, Belgium) following this stepwise procedure.

1) No-carrier-added [^{18}F]F^{-} was produced *via* the ^{18}O(p, n)^{18}F nuclear reaction by irradiation of [^{18}O]H_2O. The aqueous [^{18}F]F^{-} was directly recovered from the cyclotron target onto the automated synthesis module. 1−1.5 mL of the [^{18}F]F^{-} solution was then transferred into a vial to mix with 1 mL TBAOH (5.2 mg mL^{-1}). The reactor was heated up to 125 °C, and the solvent was evaporated under a stream of nitrogen for 4.5 min.

2) After cooling to room temperature, the pressurized precursor **15b** (0.5 mg in 0.5 mL of CH_3CN) was then transferred to the reactor, then the mixture was incubated at room temperature for 1 min. The reactor was heated up to 125 °C, and the solvent was evaporated under a stream of nitrogen for 2.5 min. After the solvent was removed, c(RGDyK) [5 mg c(RGDyK) in 0.5 mL DMSO/PBS = 5/95 (v/v)] was transferred into the reaction vessel which was then kept temperature at 25−37 °C for 15 min.

3) The reaction mixture was diluted with PBS (total volume = 10 mL) and passed through a C18 cartridge. The cartridge was flushed twice with 10 mL of PBS to the waste. [^{18}F]FTP-c(RGDyK) was eluted from the cartridge by 1 mL of ethanol.

4) The crude product was diluted with PBS (5 mL), and was injected to HPLC for purification using PBS/CH_3OH (90/10, v/v) as an eluent. [^{18}F]FTP-c(RGDyK) was collected (retention time = 3.1−5.1 min) directly onto the manifold.

5) The collected compound was further diluted with water (total volume = 40 mL) and passed through a C18 cartridge for reformulation in two steps. [^{18}F]FTP-c(RGDyK) was eluted from the cartridge by 1 mL of ethanol/water (80/20, v/v) eluent into the final formulation flask. The injectable bulk formulation is constituted of 10 mL of water, 2.3% ethanol, and the [^{18}F]FTP-c(RGDyK).

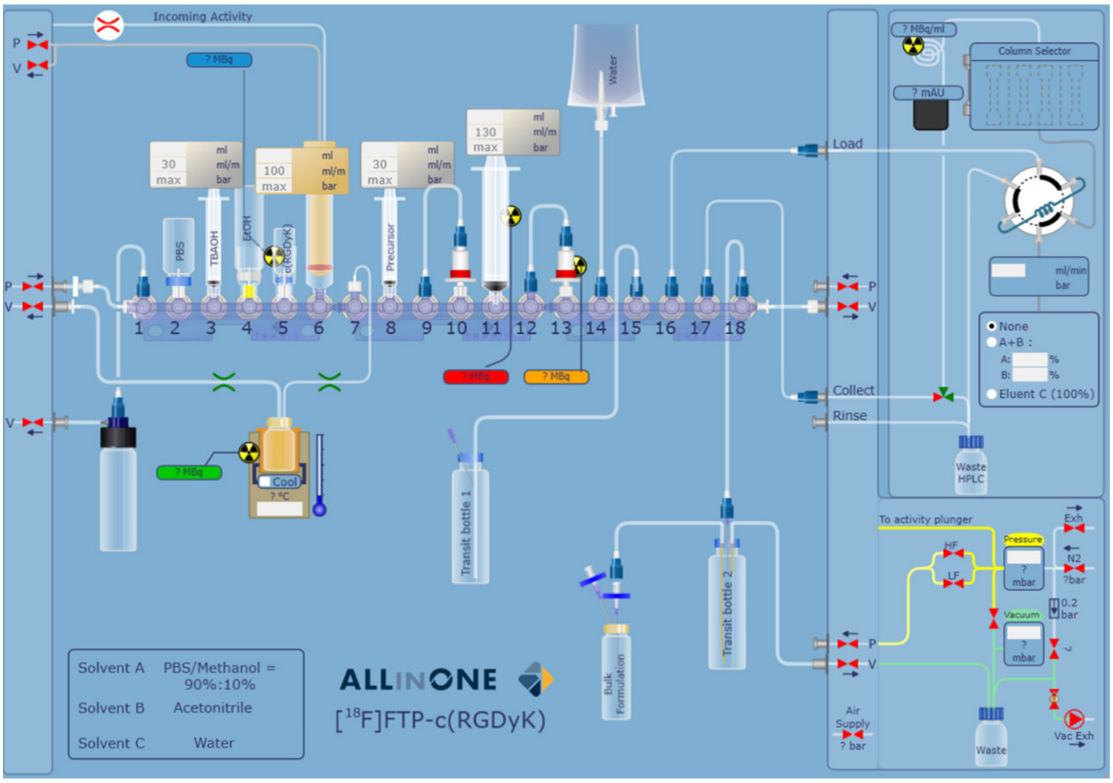

Figure S24. Graphical representation of the cassette designed for the automated synthesis of [^{18}F]FTP-c(RGDyK) on a Trasis AllinOne synthesizer.

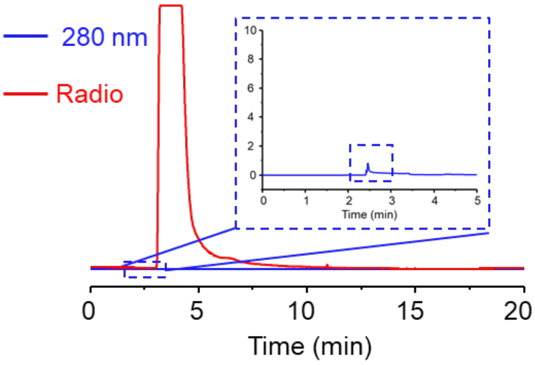

Figure S25. Radio-HPLC chromatogram of [^{18}F]FTP-c(RGDyK). Waters XBridgeC-18 (5 µm, 10 mm × 250 mm, USA). Phase A: PBS (0.02 mol L^{-1} pH = 7.4); phase B: HPLC grade acetonitrile; isocratic elution at 90% phase A and 10% phase B. Flow rate: 3.0 mL min^{-1}.

Radiochemical identity of [^{18}F]FTP-c(RGDyK)

The radiochemical identity of [^{18}F]FTP-c(RGDyK) was determined by HPLC. HPLC condition: Nacalai Tesque Cosmosil 5C18-MS-II column (4.4 µm, 4.6 mm × 250 mm,

Japan). Phase A: PBS; phase B: HPLC grade acetonitrile; 0 to 5 min: isocratic elution at 10% phase B, 5 to 30 min: 10% to 95% phase B. Flow rate: 1 mL min^{-1}, UV = 280 nm. [^{18}F]FTP-c(RGDyK) was determined by Mass spectrometry (**Figure S20c**).

RCP of [^{18}F]FTP-c(RGDyK) injection

Using the same HPLC system described for the radiochemical identity test, an appropriate volume of [^{18}F]FTP-c(RGDyK) was injected at a quantity injected that avoids uncorrected dead-time loss (for main peak) in the radioactive detection system. The RCP of [^{18}F]FTP-c(RGDyK) was determined by dividing the radioactivity associated with the [^{18}F]FTP-c(RGDyK) peak by total activity assayed in the chromatogram multiplied by 100. The product met this acceptance specification and the radiochemical purity was greater than 95% (**Figure S20c**).

Residual solvent analysis of [^{18}F]FTP-c(RGDyK) injection

Analyses of residual solvent levels in [^{18}F]FTP-c(RGDyK) were conducted using an Agilent 5979I gas chromatograph. The product met this acceptance specification and the acetonitrile level was less than 10 ppm.

pH of [^{18}F]FTP-c(RGDyK) injection

A drop of the [^{18}F]FTP-c(RGDyK) final product matrix was applied to pH indicator paper (Newstar pH-Indicator strip, pH 5.5–9.0). The strip color was matched to an indicator chart. The product met this acceptance specification and the pH was between 7.0 and 7.5.

7 Molar activity calculation

Standard UV absorption curves [Y axis = UV area, X axis = mass (μg)] were created from the HPLC traces of corresponding standard solutions. The UV area overlapping with radio peak was then recorded. The standard curve was used to calculate the mass and mole number. Each radiolabeled product was purified and collected by HPLC. Dividing the product activity by the mole number gives its molar activity (the measured radioactivity per mole of compound, A_m) in GBq μmol^{-1}.

7.1 Calculation of A_m of [^{18}F]1

Solutions of **1** at graded concentrations (10, 20, 40, 200, 400 ng/15 μL) were prepared and analyzed by an analytical HPLC. The wavelength was set at 224 nm. The UV absorption peak areas at different concentrations of **1** were measured and the relationship between the concentration of the substance and the area of the absorption peak was obtained by linear analysis. The mass-UV absorption standard curve of **1** is given below.

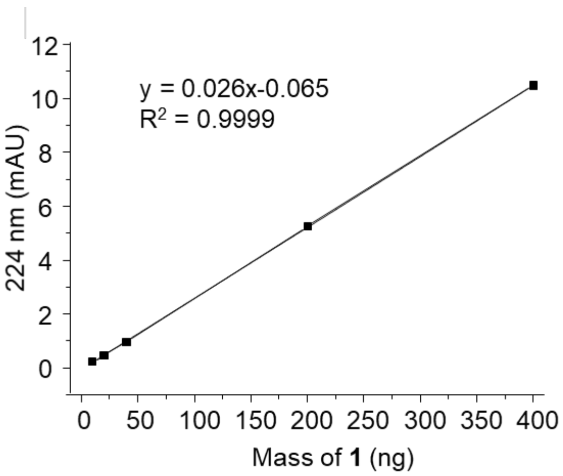

Figure S26. A linear standard curve was generated by correlating the HPLC signal (224 nm, mAU) and the injected mass of **1** (ng). The HPLC UV signal was obtained by injecting 10, 20, 40, 200, 400 ng of **1**.

HPLC condition: phase A: PBS (0.01 mol L^{-1}, pH = 7.4); phase B: HPLC grade acetonitrile; isocratic elution at 70% phase A and 30% phase B; flow rate: 1.0 mL min^{-1}.

The calculation of A_m of [^{18}F]**1** by manual radiosynthesis:

For example: 74 MBq radioactivity was collected from HPLC. Hence, A_m of [^{18}F]**1** = activity of [^{18}F]**1**/molar amount of **1** = 74 MBq/1.81 nmol = 40.70 GBq μmol^{-1}.

7.2 Calculation of A_m of [^{18}F]2

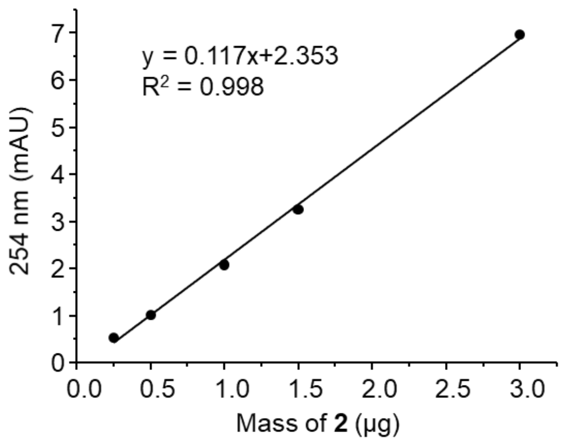

Figure S27. A linear standard curve was generated by correlating the HPLC signal (254 nm, mAU) and the injected the injected mass of **2** (μg). The HPLC signal was obtained by injecting 0.25, 0.5, 1, 1.5, 3 μg of **2**.

HPLC condition: phase A: PBS (0.01 mol L^{-1}, pH = 7.4); phase B: HPLC grade acetonitrile; isocratic elution at 80% phase A and 20% phase B; flow rate: 1.0 mL min^{-1}.

For example, A_m of [^{18}F]**2** = activity of [^{18}F]**2**/molar amount of **2** = 77.71 MBq/2.22 nmol = 35.15 GBq μmol^{-1}.

7.3 Calculation of A_m of [^{18}F]15 ([^{18}F]FTP-NHS)

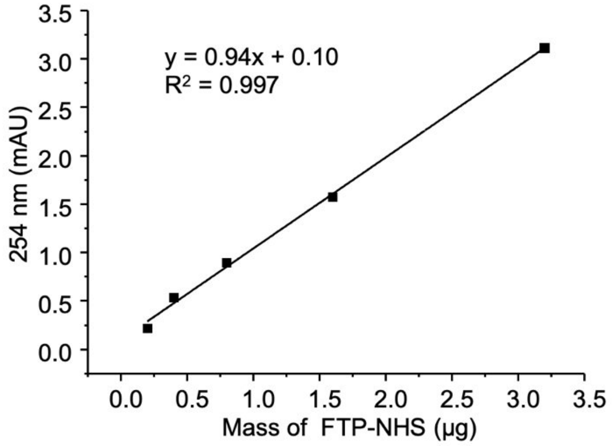

Figure S28. A linear standard curve was generated by correlating the HPLC signal (254 nm, mAU) and the injected mass of FTP-NHS (μg). The HPLC signal was obtained by injecting 0.2, 0.4, 0.8, 1.6, 3.2 μg of FTP-NHS.

HPLC condition: phase A: PBS (0.02 mol L^{-1}, pH = 7.4); phase B: HPLC grade methanol; isocratic elution at 85% phase A and 15% phase B; flow rate: 1.0 mL min^{-1}.

For example, A_m of [^{18}F]FTP-NHS = activity of [^{18}F]FTP-NHS/molar amount limit of FTP-NHS = 64.1 MBq/0.5 nmol = 128.2 GBq μmol^{-1}.

7.4 Calculation of A_m of [^{18}F]FTP-Tyr

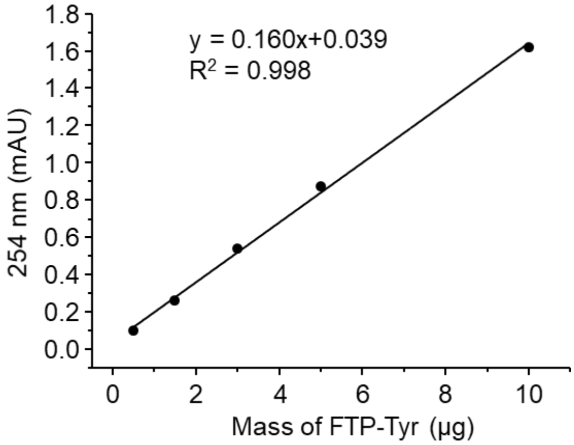

Figure S29. A linear standard curve was generated by correlating the HPLC signal (254 nm, mAU) and the injected mass of FTP-Tyr (μg). The HPLC signal was

obtained by injecting 0.5, 1.5, 3, 5, 10 μg of FTP-Tyr.

HPLC condition: phase A: PBS (0.01 mol L^{-1}, pH = 7.4); phase B: HPLC grade methanol; isocratic elution at 90% phase A and 10% phase B; flow rate: 1.0 mL min^{-1}.

Because the UV absorption peak with the [^{18}F]FTP-Tyr is too small to detect, the HPLC UV detection limit of [^{19}F]FTP-Tyr is used as the mass of FTP-Tyr.

For example, A_m of [^{18}F]FTP-Tyr > activity of [^{18}F]FTP-Tyr/molar amount limit of FTP-Tyr = 66.61 MBq/17 nmol = 3.91 GBq μmol^{-1}. Thus, A_m of [^{18}F]FTP-Tyr > 3.91 GBq μmol^{-1}.

7.5 Calculation of A_m of [^{18}F]FTP-AMP

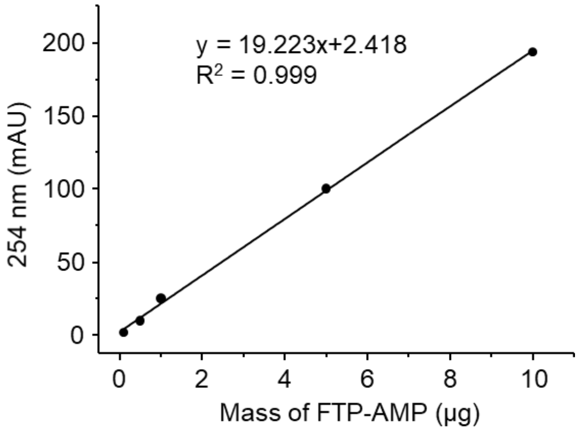

Figure S30. A linear standard curve was generated by correlating the HPLC signal (254 nm, mAU) and the injected mass of FTP-AMP. The HPLC signal was obtained by injecting 0.1, 0.5, 1, 5, 10 μg of FTP-AMP.

HPLC condition: solvent A: PBS (0.01 mol L^{-1} pH = 7.4); solvent B: HPLC grade acetonitrile; isocratic elution at 75% solvent A and 25% solvent B; flow rate: 1.0 mL min^{-1}.

For example, A_m of [^{18}F]FTP-Tyr = activity of [^{18}F]FTP-AMP/molar amount of [^{18}F]FTP-AMP = 15.95 MBq/3.2 nmol = 4.98 GBq μmol^{-1}.

7.6 Calculation of A_m of [^{18}F]FTP-c(RGDyK)

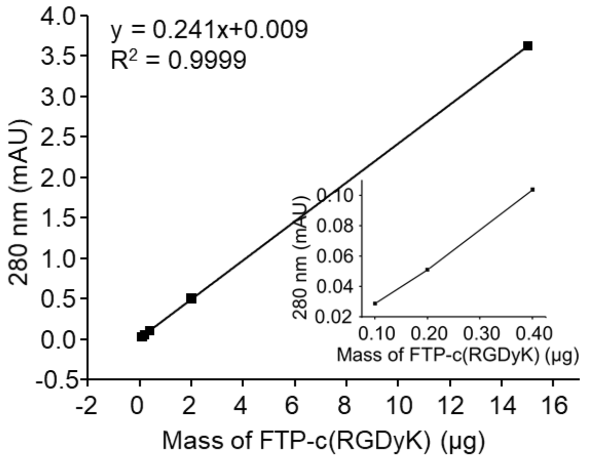

Figure S31. A linear standard curve was generated by correlating the HPLC signal (280 nm, mAU) and the injected mass of FTP-c(RGDyK). The HPLC signal was obtained by injecting 0.1, 0.2, 0.4, 2, 15 μg of FTP-c(RGDyK).

HPLC condition: solvent A: H_2O; solvent B: HPLC grade acetonitrile; isocratic elution at 90% solvent A and 10% solvent B; flow rate: 1.0 mL min^{-1}.

When [^{18}F]FTP-c(RGDyK) was manually synthesized, A_m of [^{18}F]FTP-c(RGDyK) = activity of [^{18}F]FTP-c(RGDyK)/molar amount of c(RGDyK) = 10.7 MBq/0.1 nmol = 107 GBq μmol^{-1}.

When [^{18}F]FTP-c(RGDyK) was synthesized by automated radiosynthesis module, for example, A_m of [^{18}F]FTP-c(RGDyK) = activity of [^{18}F]FTP-c(RGDyK)/molar amount of c(RGDyK) = 310 MBq/0.6 nmol = 517 GBq μmol^{-1}.

7.7 Calculation of A_m of [^{18}F]FTP-HSA

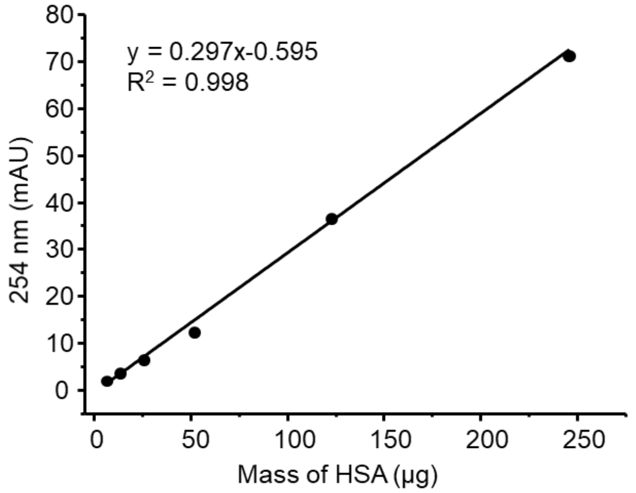

Figure S32. A linear standard curve was generated by correlating the HPLC signal (254 nm, mAU) and the injected mass of HSA. The HPLC signal was obtained by injecting 6.5, 13.5, 26, 52, 123, 246 μg of HSA.

HPLC condition: eluent: PBS (0.01 mol L^{-1}, pH = 7.4); flow rate: 1.0 mL min^{-1}.

For example, [^{18}F]FTP-HSA was manually synthesized. A_m of [^{18}F]FTP-c(RGDyK) = activity of [^{18}F]FTP-c(RGDyK)/molar amount of c(RGDyK) = 46.97 MBq/1.2 nmol = 39.14 GBq μmol^{-1}.

8 Lipophilicity (Log *D*)

The distribution coefficient (Log *D*) of ^{18}F-labeled FTPs were measured in a pre-saturated 1-octanol/PBS (pH = 7.4) system. The Log *D* was measured following the method: ^{18}F-labeled FTPs (1.11 MBq) dissolved in 100 μL H_2O and diluted with 0.9 mL PBS (0.05 mol L^{-1}, pH = 7.4) and 1 mL 1-octanol. After shaking for 3 min, the mixture was centrifuged at 6000 rpm for 5 min. The counts of 100 μL organic layer and 100 μL inorganic layer were determined by a gamma counter, respectively. The following equation was used to calculate log *D* = (activity in octanol phase-background activity)/(activity in aqueous phase-background activity). All the experiments were performed with triplicate samples and reported as mean ± SD.

$$\text{Log } D = \lg \frac{\text{Counts/mL in octanol}}{\text{Counts/mL in PBS}}$$

Table S7. Log *D* of various FTPs.

Compound	Log *D*
[^{18}F]**1**	-1.09 ± 0.05
[^{18}F]**3**	-1.77 ± 0.02
[^{18}F]FTP-Tyr	-0.63 ± 0.06
[^{18}F]FTP-AMP	-0.02 ± 0.01

Compared with [^{18}F]**3**, [^{18}F]**1** has stronger liposolubility, which is conducive to improving bioavailability. Therefore, we mainly design, synthesize and evaluate phosphorodithioates probes as a phosphate mimics.

9 Stabilities of precursors and ^{18}F-labeled FTPs

9.1 Stabilities of precursors at different pHs

1.0 mg of precursors **1b** or **2b** (chemical purity > 95%) was dissolved in 50 μL acetonitrile, then total volume was adjusted to 500 μL by different pH solution from 1 to 13. The solutions were incubated at 37 °C for 2 h, and the chemical purities were assayed by HPLC.

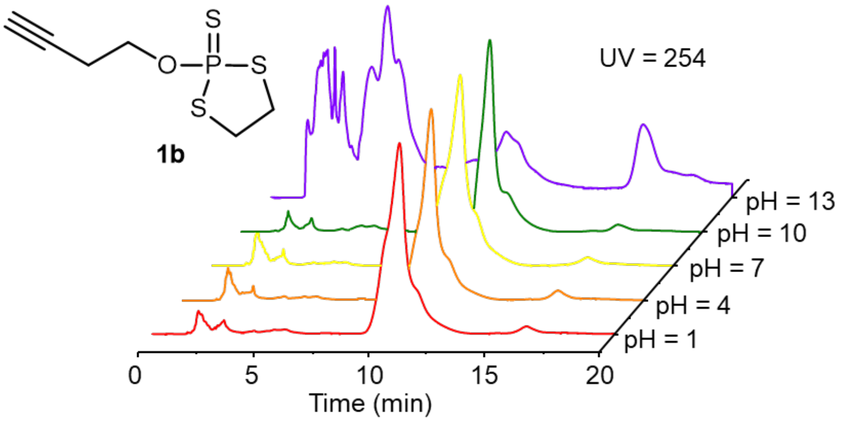

Figure S33. HPLC analysis of stabilities of precursor **1b** in different pH solutions.

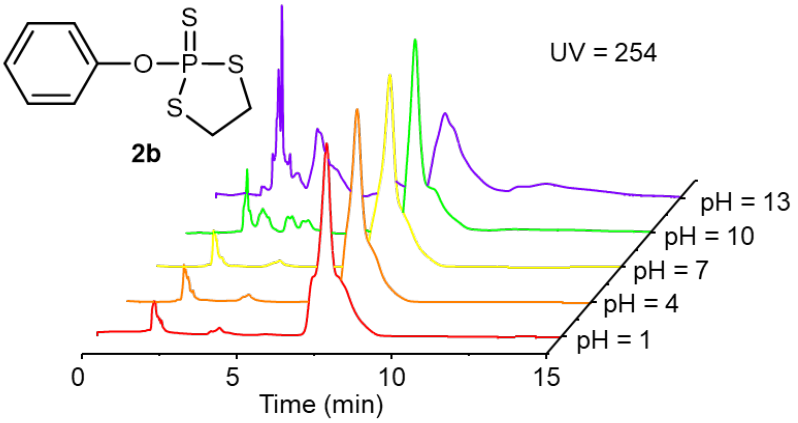

Figure S34. HPLC analysis of stabilities of precursor **2b** in different pH solutions.

9.2 Stabilities of ^{18}F-labeled FTPs at different pHs

Purified [^{18}F]**1** or [^{18}F]**2** (1-3 MBq, ~10 μL) was added into 100 μL solution at different pH from 1 to 13. The mixture was incubated at 37 °C after 2 h, and RCPs was assayed by radio-HPLC.

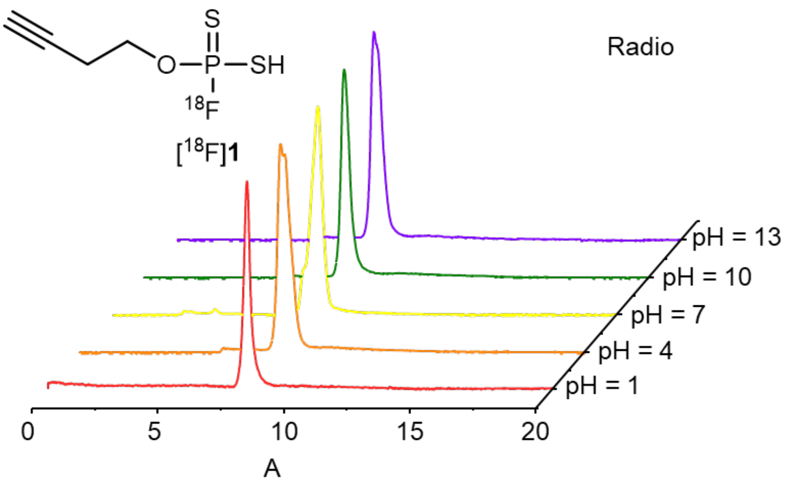

Figure S35. HPLC analysis of stabilities of [^{18}F]**1** at different pH solutions.

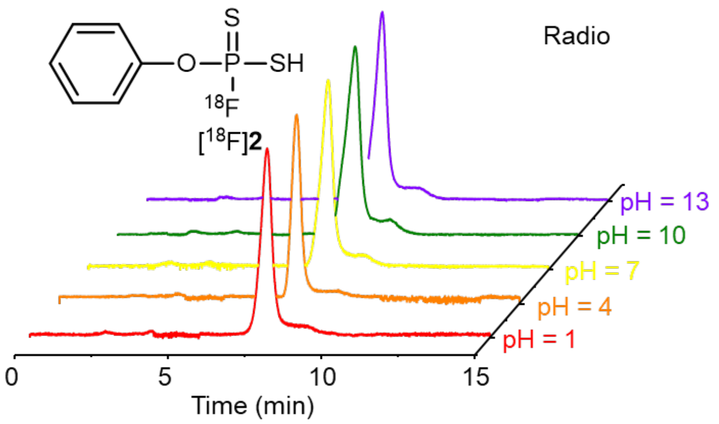

Figure S36. HPLC analysis of stabilities of [^{18}F]**2** at different pH solutions.

9.3 Stabilities of ^{18}F-labeled FTPs in PBS and serum

Purified [^{18}F]**1**, [^{18}F]**2**, [^{18}F]**3**, [^{18}F]**17** or [^{18}F]**18** (0.1–0.3 MBq, 10 μL) was added into 90 μL PBS, respectively. The mixtures were incubated at 37 °C after 0.5, 1, 2 h, then RCPs was assayed by radio-HPLC.

The stabilities in serum were determined in the same way with 90 μL mouse serum added instead. The mixtures were incubated at 37 °C after 0.5, 1, 2 h. After incubation, 100 μL acetonitrile was added to the mixture, and centrifuged at 16, 543 × g for 8 min. The supernatant was filtered by a 0.22 um Millipore filter. RCPs of the filtrate was analyzed by radio-HPLC.

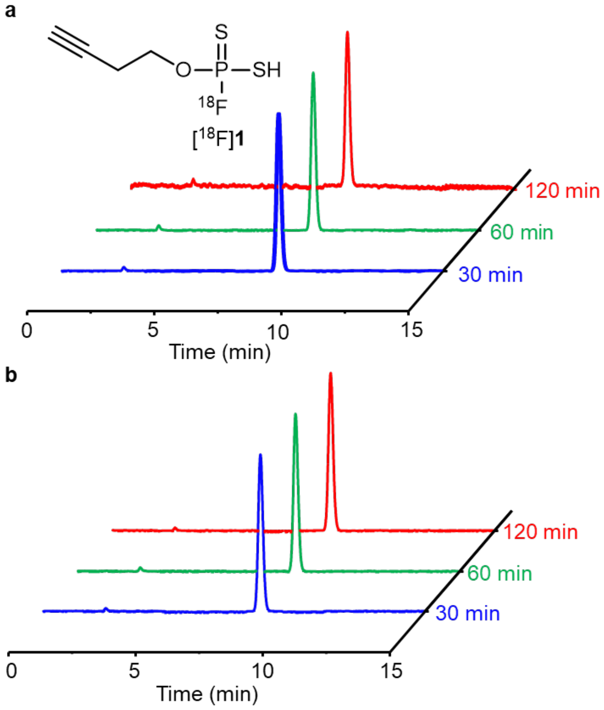

Figure S37. Stabilities of [^{18}F]**1** in PBS (**a**) and serum (**b**).

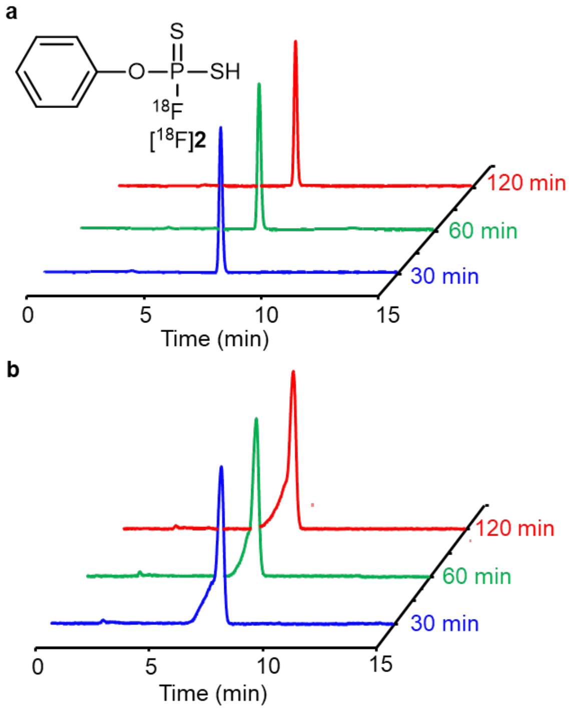

Figure S38. Stabilities of [^{18}F]**2** in PBS (**a**) and serum (**b**).

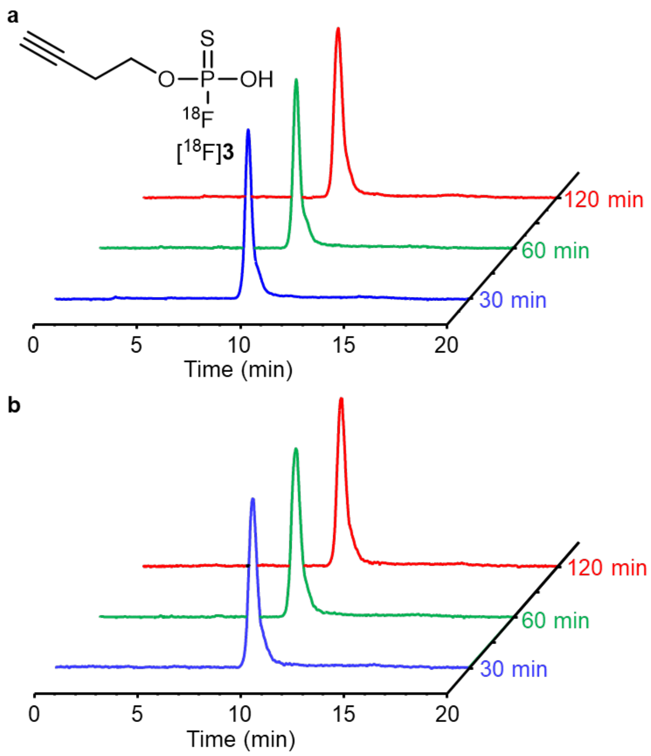

Figure S39. Stabilities of [^{18}F]**3** in PBS (**a**) and serum (**b**).

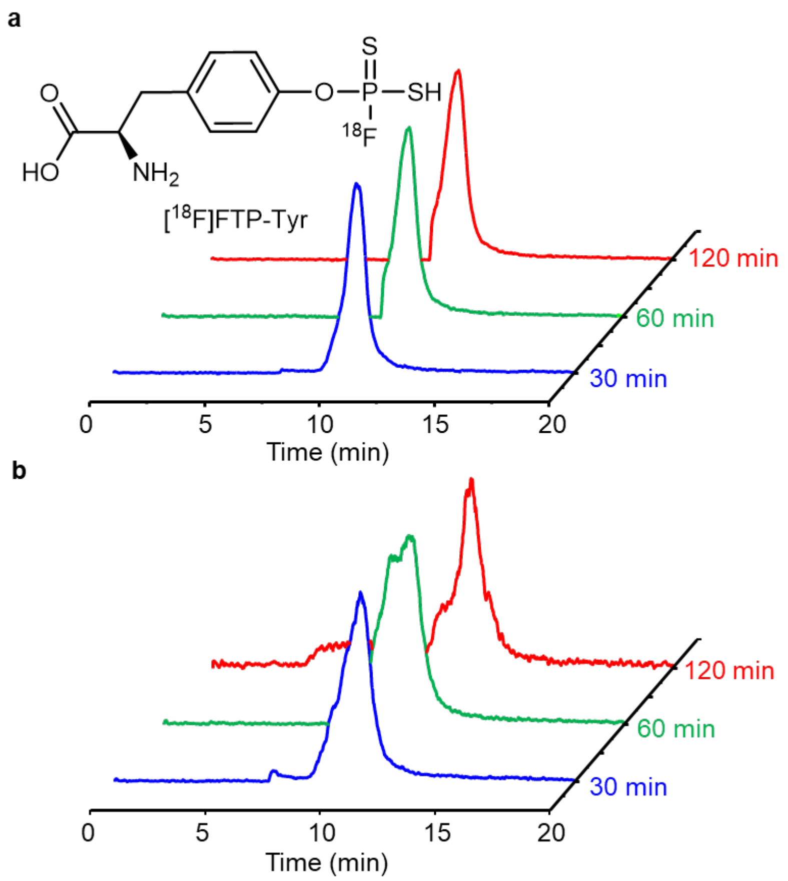

Figure S40 Stabilities of [^{18}F]FTP-Tyr in PBS (**a**) and serum (**b**).

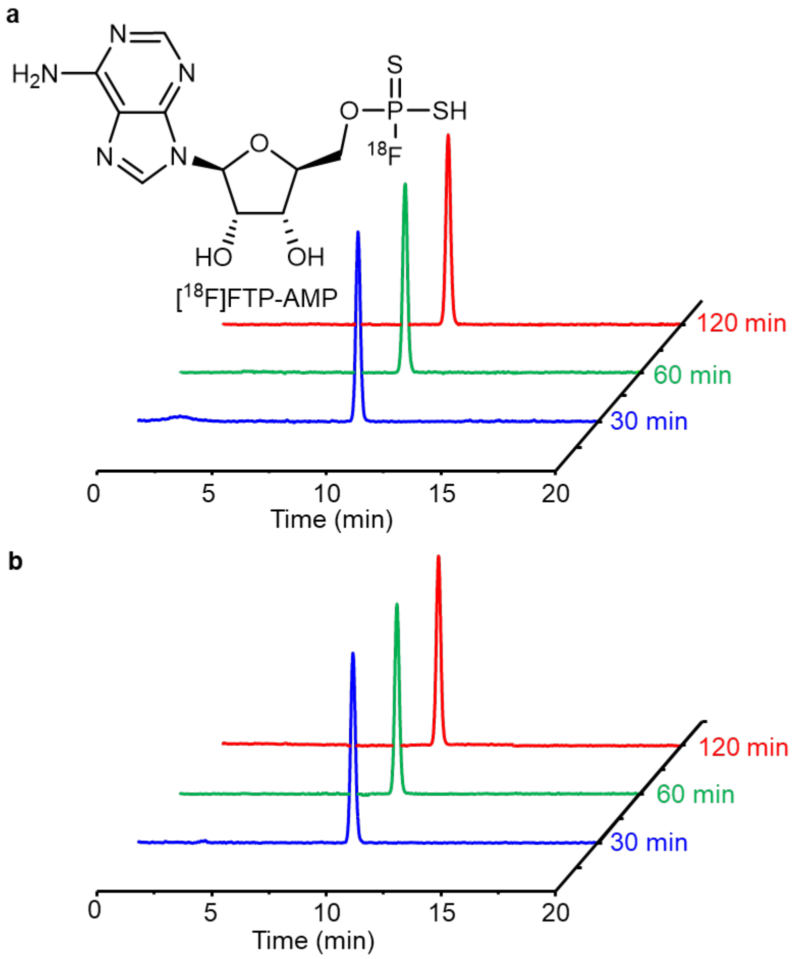

Figure S41. Stabilities of [^{18}F]FTP-AMP in PBS (**a**) and serum (**b**).

9.4 Storage stability of [^{18}F]FTP-c(RGDyK) in saline

10 μL purified [^{18}F]FTP-c(RGDyK) (0.1–0.3 MBq, RCP > 99%) was added into 90 μL saline (pH = 7). The solution was incubated at 37 °C. At 3, 6 h, the RCPs were assayed by radio-HPLC. [^{18}F]FTP-c(RGDyK) shows high storage stability in 6 h (about three half-lives) in saline.

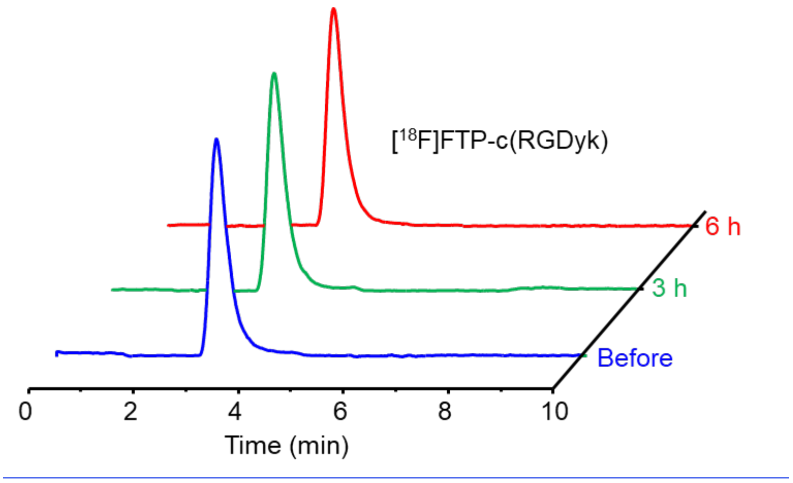

Figure S42. Storage stability of [^{18}F]FTP-c(RGDyK) in saline.

9.5 The stabilities of [^{18}F]FTP-Pds-PEG *in vitro*

Purified [^{18}F]FTP-Pds-PEG (1–3 MBq, 100 µL) about 10 µL was added into 90 µL PBS and serum respectively. The mixture was incubated at 37 °C after 30, 60, 90, 120, 240 min. Stabilities of [^{18}F]FTP-Pds-PEG were investigated by radio-TLC. Polyamide film/saline system was used to identify [^{18}F]F$^-$ and [^{18}F]FTP-Pds-PEG. [^{18}F]FTP-Pds-PEG stayed at the point of origin (Rf: 0–0.1).

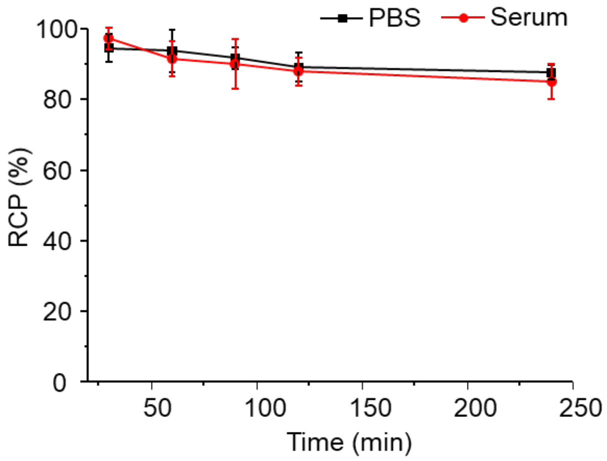

Figure S43. Stabilities of [^{18}F]FTP-Pds-PEG in PBS and serum.

9.6 Enzymatic stabilities of ^{18}F-labeled FTPs *in vitro*

[^{18}F]FTP-Tyr (0.1–0.3 MBq, 10 μL) was dissolved in 50 μL reaction buffer (10.0 mM Tris-HCl (pH = 8.0)), 5.0 mM $MgCl_2$, 0.1 M KCl， 0.02% Triton X-100, 1 mM 2-mercaptoethanol, 0.1mg mL^{-1} BSA). 5 μL beyoAP alkaline phosphatase was added to the solution, then incubated at 37 °C after 30, 60, 120 min. 50 μL acetonitrile was added to the mixture, and centrifuged at 16, 543 × g for 8 min. RCPs of the filtrate was analyzed by radio-HPLC.

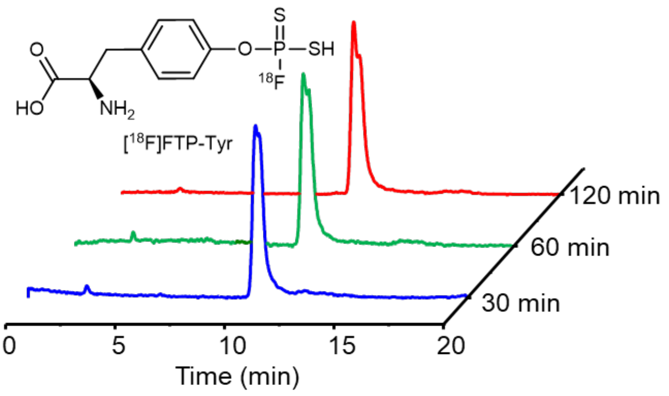

Figure S44. Enzymatic stabilities of [^{18}F]FTP-Tyr.

9.7 *In vivo* metabolic stabilities of ^{18}F-labeled FTPs

In vivo metabolic stabilities of ^{18}F-labeled FTPs in normal ICR mice were examined. Normal ICR mice were intravenously injected with [^{18}F]**1**, [^{18}F]**2**, or [^{18}F]**3** (18.50 MBq in 100 μL saline), respectively. Each mouse was sacrificed at indicated time with blood and urine collected. Each blood sample was immediately centrifuged at 10000 rpm for 5 min to obtain the serum. The serum was then treated with acetonitrile and centrifuged at 10000 rpm for 8 min to provide the supernatant. The supernatant was collected and passed through a 0.22 μm Millipore filter. Each urine sample was diluted with 100 μL water and passed through a 0.22 μm millipore filter. RCPs of ^{18}F-labeled FTPs in all samples were analyzed by radio-HPLC.

[^{18}F]**1** and [^{18}F]**2** have better metabolic stability than [^{18}F]**3** *in vivo*, and 80% of them

remain in the form of the original drug after 60 minutes inject. [^{18}F]**1** and [^{18}F]**2** almost disappeared in urine, and two new peaks appeared in the HPLC analysis after 5 min injection. The radiometabolite remained unknown but differed from [^{18}F]F^{-}, whose retention time was approximately 2.2 min. It can be clearly seen that, compared with [^{18}F]**3**, [^{18}F]**1** has a longer circulation and better metabolic stability *in vivo*, so phosphorodithioates are more suitable for use as a PET probe.

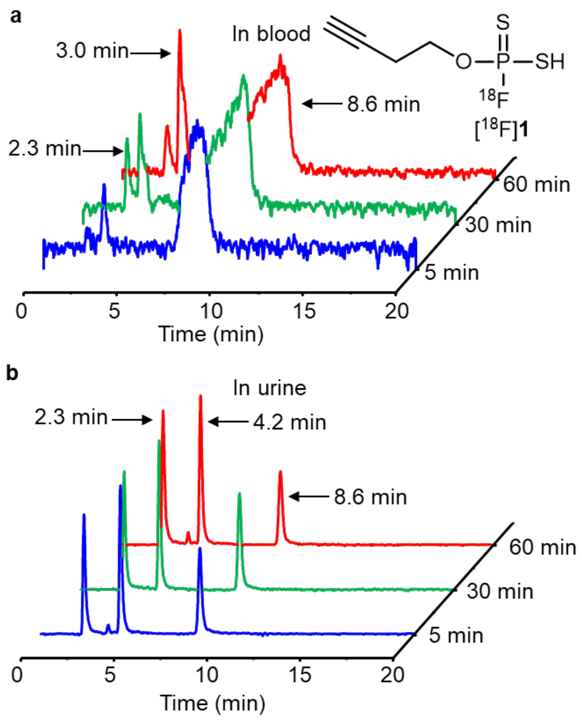

Figure S45. Metabolite profile of [^{18}F]**1** in blood (**a**)/urine (**b**).

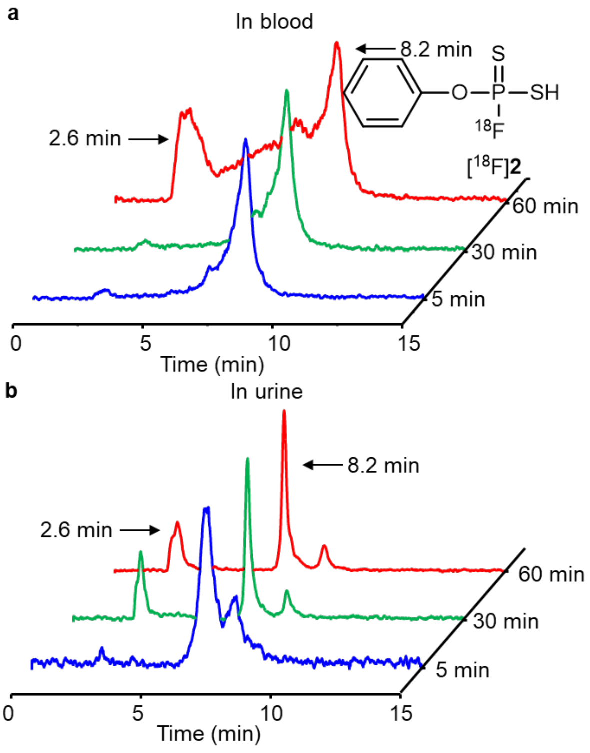

Figure S46. Metabolite profile of [^{18}F]**2** in blood (**a**)/urine (**b**).

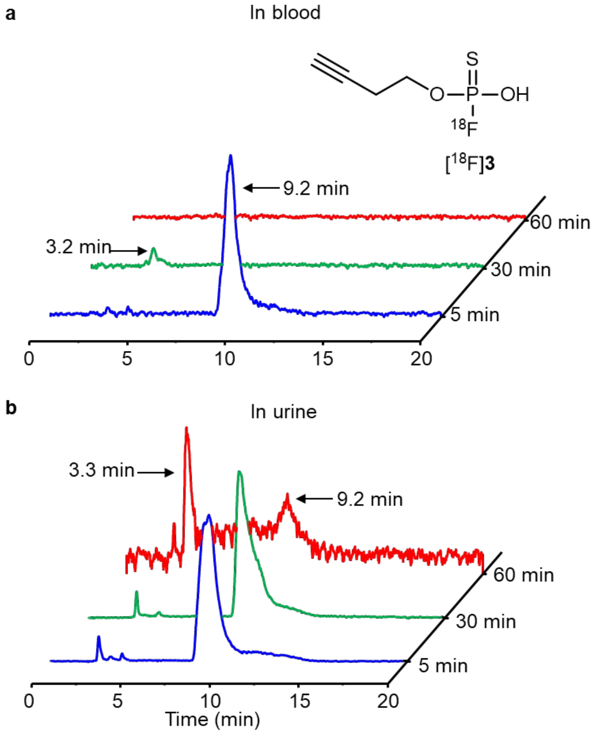

Figure S47. Metabolite profile of [^{18}F]**3** in blood (**a**)/urine (**b**).

9.8 *In vivo* stabilities of ^{18}F-labeled FTPs during dynamic PET imaging

General procedure:

Normal mice (n = 3) anesthetization was conducted with 2–4% isoflurane on room air/oxygen (80/20) and a tail vein cannula was placed for dosing of depurated ^{18}F-labeled FTPs (1–3 MBq, 100 μL). Core body temperature of the mice was maintained at 37 °C and respiration rate was monitored and kept at 40–60 beat per minute (BPM) for the duration of the imaging protocol. 60 min dynamic whole-body microPET imaging were performed by a Siemens Inveon microPET-CT (Siemens, Germany).

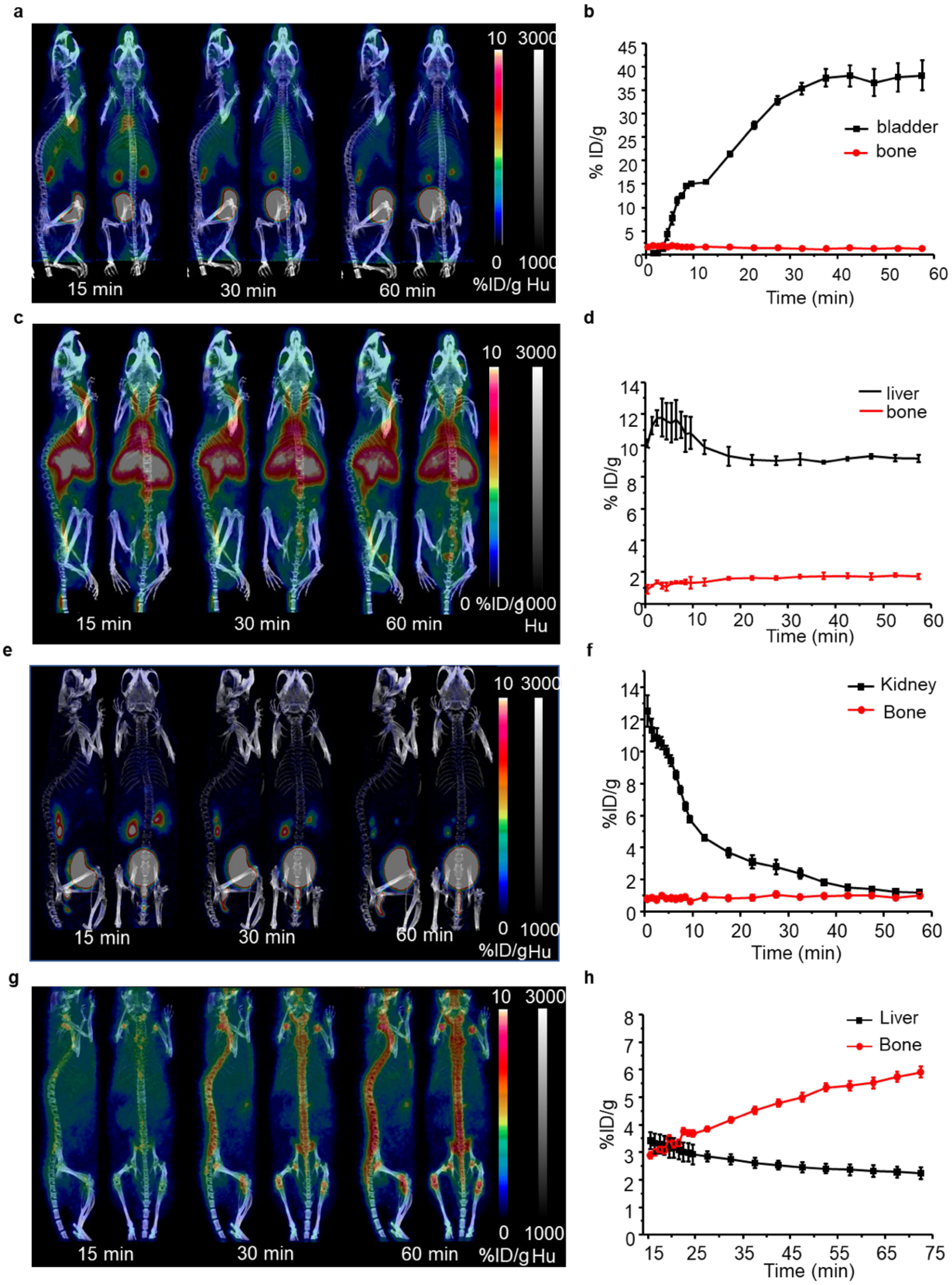

Figure S48. *In vivo* stabilities of FTPs during dynamic PET imaging. Dynamic microPET/CT images of [^{18}F]**1** (**a**), [^{18}F]**2** (**c**), [^{18}F]**3** (**e**) and [^{18}F]**4** (**g**) over 60 min after injection. Time-activity curves of [^{18}F]**1** (**b**), [^{18}F]**2** (**d**), [^{18}F]**3** (**f**) and [^{18}F]**4** (**h**) in selected organs.

It was found that the *in vivo* stability of phosphate mimic could be significantly

improved after oxidized, and that anions also contribute to its stability.

When the anion was occupied, [^{18}F]**4** was slowly defluorinated in the body, but this defluorination seemed to be acceptable, with only 3% increase in bone uptake within 1 h.

10 Biodistribution studies

The *in vivo* biodistribution of four ^{18}F-labeled FTPs ([^{18}F]**1**, [^{18}F]**2**, [^{18}F]**3** and [^{18}F]FTP-NHS/[^{18}F]**15**) were studied in BALB/c male mice (average weight 20 ± 2.0 g) by injecting a solution (in saline, pH = 7) of each compound (100 μL, 10 μCi/mouse, 0.1–0.2 nmol) into the tail vein of the animals. At 120 min post injection, the animals were sacrificed and the main organs were removed, weighed, and then counted by a γ-counter. In reference to a standard of the injected solution, results were expressed as the percentage of the injected dose per gram of wet tissue or organ (%ID g^{-1}).

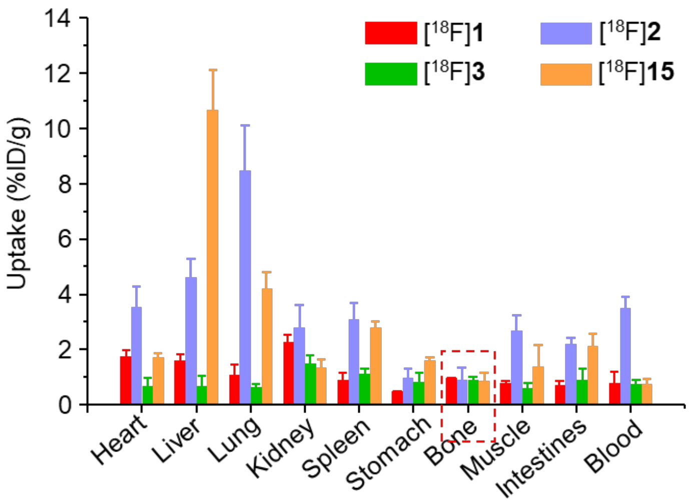

Figure S49. Comparative biodistribution studies at 120 min post injection of [^{18}F]**1**, [^{18}F]**2**, [^{18}F]**3** and [^{18}F]FTP-NHS ([^{18}F]**15**).

11 Similarity between the FTPs and phosphates

11.1 Delocalized molecular orbital

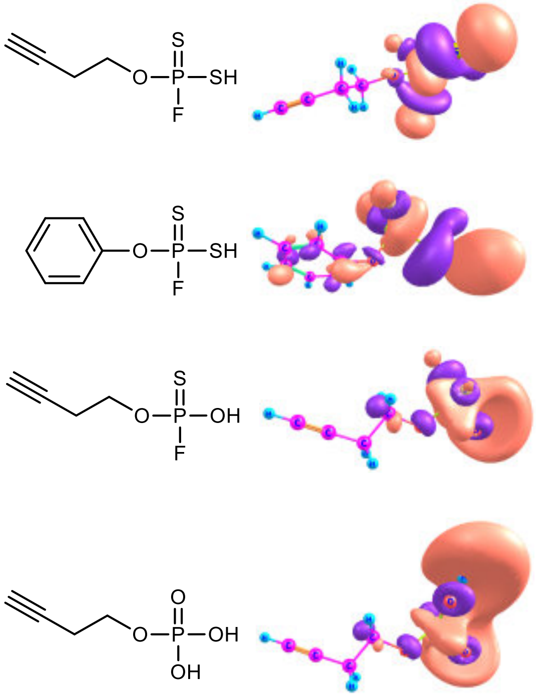

1
LUMO
E = -1.40 eV

2
LUMO
E = -1.45 eV

3
LUMO
E = -0.62 eV

Phosphate derivative
LUMO
E = -0.67 eV

Figure S50. Delocalized molecular orbitals of three FTP derivatives and a phosphate derivative.

11.2 Molecular electrostatic potential

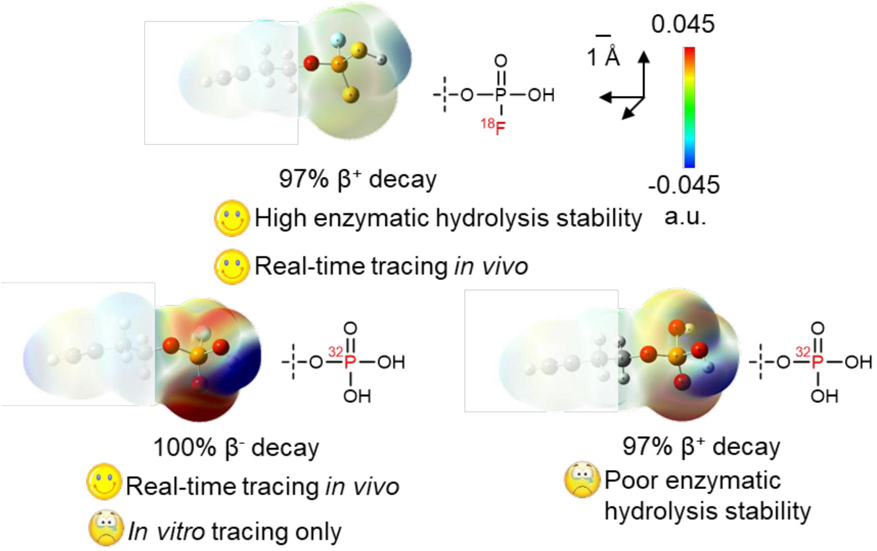

Figure S51. Molecular electrostatic potential (MEP) maps of FTPs and the original phosphates, which are performed at the B3LYP/6-31G (*d, p*) level in the water phase (Blue indicates the positive charge, and red indicates negative charge).

11.3 Molecular docking

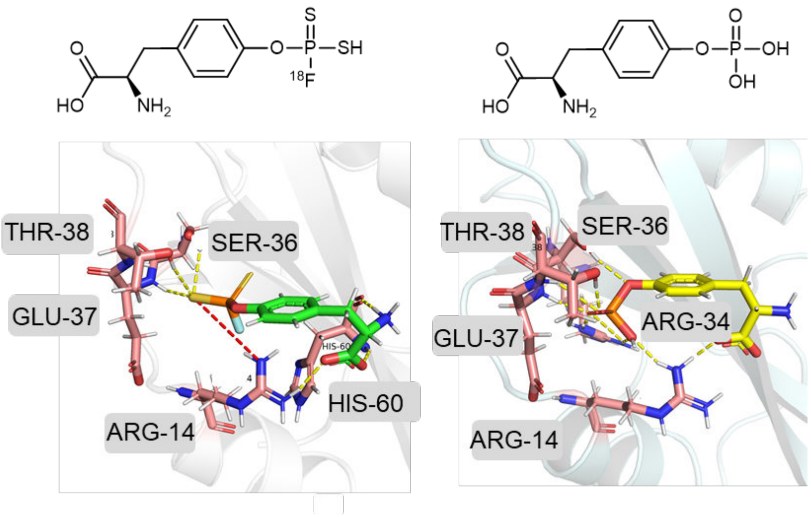

Figure S52. Schematic representation of the binding between Tyr/FTP-Tyr and SH2 site. SH2 domain (grey) is in solid ribbon representation. Amino acid residues (ARG-14, SER-36, GLU-37, THR-37, ARG-34, HIS-60 in the binding site are in stick representation.

11.4 Time-dependent cell uptake assay

All cell lines were kindly provided by the Cell Bank of the Chinese Academy of Sciences in Shanghai. Cells were cultured in Dulbecco's modified Eagle's medium (DMEM, GIBCO) supplemented with 10% fetal bovine serum (FBS, GIBCO), 1% 100 μg mL^{-1} streptomycin and 100 IU mL^{-1} penicillin (MRC) at 37 °C in the humidified circumstance containing 5% CO_2. When cells reached 90% confluence in the logarithmic growth phase, they were harvested or passaged.

B16 cells at a density of 1 × 10^5 cells/well were seeded into 24-well plates and incubated at 37 °C for overnight. After washing with warm binding buffer (PBS, pH 7.4, plus 0.2% BSA), the cells were incubated with [^{18}F]FTP-Tyr (100 μL, 1.11 MBq/10 mL) for 5, 15, 30, 45, 60, 120 min at 37 °C. After incubation, cells were washed with ice-cold binding buffer for three times, and lysed by 0.5 mL of 0.5 M NaOH, collected into test tubes and measured by a γ-counter.

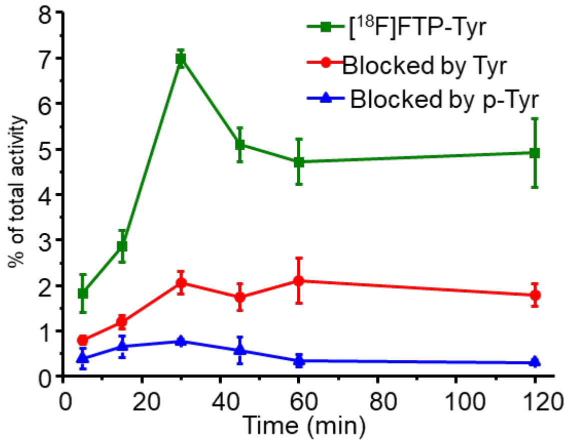

Figure S53. Time-dependent cell uptake assays performed using [^{18}F]FTP-Tyr in B16 cells.

11.5 Cell proliferation assay

HepG2 cells were cultivated in a Petrie dish, and medium with 10% Fetal Bovine Serum and 100 U mL^{-1} Penicillin-Streptomycin and maintained in a humidified 37 °C

incubator with 5 % CO_2. Subsequently, trypsin with EDTA were used to detach cells detach from the plates. Cells were counted and seeded equally into each well of 24-well plates. Each well contains around 900-1000 cells were then incubated in 0, 0.1×, 0.5×, 1.0×, 2.0× and 5.0× P-Tyr and FTP-Tyr (1× = 1.67 mM) with medium for 1, 2, 3, 4, 5 d at 37 °C. After incubated, the 3-(4,5-dimethyl-2-thiazolyl)-2,5-diphenyl-2-H-tetrazolium bromide (MTT) solution (5 mg mL^{-1}, 50 μL) was added to the wells for 4 h at 37 °C. Then the medium was aspirated and added 150 μL DMSO to well for 10 min. 100 μL of mixed solution from each well was taken and measured absorbance at 490 nm.[13]

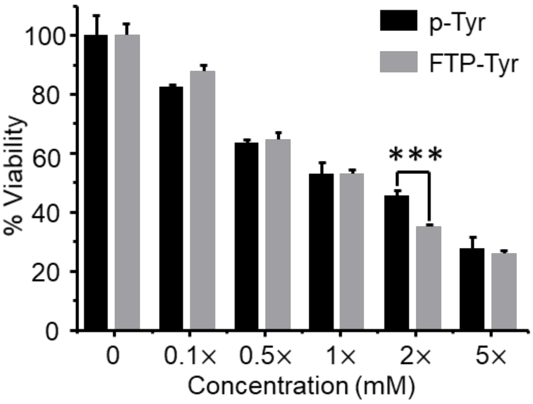

Figure S54. Relative $HepG_2$ cell viability in the presence of p-Tyr and FTP-Tyr (n = 3).

11.6 Western Blot (WB)

To determine whether incubation with p-Tyr could modulate insulin induced IR tyrosine phosphorylation, $HepG_2$ cells were grown to 6 well plate. The cells were washed and incubated overnight with culture medium with 10% FBS. Cells were then incubated in 0, 0.5×, and 5.0× p-Tyr or FTP-Tyr containing medium (1× = 1.67 mM) for 24 h at 37 °C. At the end of the p-Tyr and FTP-Tyr incubation, cells were further incubated with 1 U mL^{-1} of insulin for 5 min. At the end of the insulin incubation, the culture medium was removed and the cells were washed with sterile Hank's balanced salt solution and subsequently lysed with 1 mL of Laemmli's buffer. The samples were briefly sonicated and stored at -20 °C prior to determination of insulin induced

IR tyrosine phosphorylation by Western blotting using anti-phosphotyrosine antibody. The IR levels were determined using an anti-IR monoclonal mouse.

After SDS-PAGE, proteins were transferred onto methanol-preactivated Immobilon-P PVDF membranes (pore size 0.45 µm; Millipore) using a semi-dry transfer cell (Bio-Rad). After transfer, the membranes were blocked for 1 h with TBST wash buffer (20 mM Tris, pH 7.5, 150 mM NaCl, 0.1% Tween-20) containing 5% BSA (Fisher or Thermo Scientific). Membrane was then incubated with mouse anti-phosphotyrosine antibody [PY20] in 5% BSA-TBST. The membrane was washed with TBST three times for 5 min (3 × 5 min) followed by 2 h incubation with anti-mouse IgGκ BP-HRP in 5% BSA-TBST, washed 3 × 5 min with TBST and then visualized using SuperSignal West Pico PLUS chemiluminescent substrate (ThermoFisher Scientific) and recorded on a chemiluminescent western blot imaging system.[14]

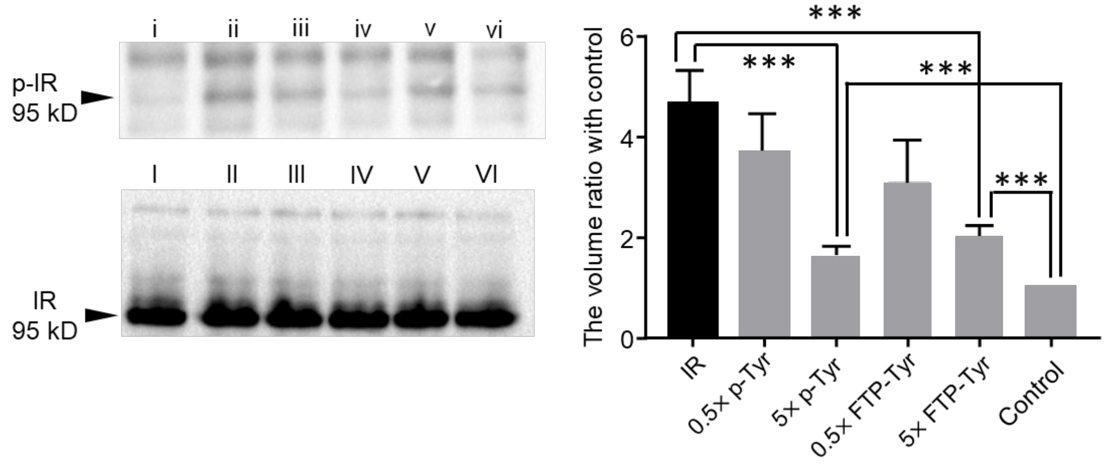

Figure S55. Inhibition of phosphorylation of insulin receptor (IR) with FTP-Tyr and p-Tyr in $HepG_2$ cells. Lane i, $HepG_2$ cells; ii, $HepG_2$ cells treated with insulin (1 unit mL^{-1}, 5 min); iii, p-Tyr (0.5 ×, 24 h) treated cells incubated with insulin; iv, p-Tyr (5 ×, 24 h) treated cells incubated with insulin; v, FTP-Tyr (0.5 ×, 24 h) treated cells incubated with insulin; vi, p-Tyr (5 ×, 24 h) treated cells incubated with insulin. Semi-quantitative analysis was performed (1 × = 1.67 mM).

12 Animal models

All animal experiments were conducted according to the Guide for the Care and Use of Laboratory Animals, after approval from the Xiamen University animal ethics committee.

12.1 MDA-MB-453 cells xenograft animal model

Human breast cancer cell line MDA-MB-453 were obtained from were obtained from China Infrastructure of Cell Line Resources. The cells were cultured in Leibovitz L15 medium supplemented with 10% (v/v) heat-inactivated fetal bovine serum (GIBCO) and penicillin/streptomycin (concentration of 100 U mL^{-1} for each). Cells were grown as a monolayer at 37 °C in a humidified atmosphere containing 5% CO_2.

5 to 7-week-old female Balb/C nude mice were injected subcutaneously into the right front flanks with MDA-MB-453 cells (1×10^7 in 200 μL of phosphate-buffered saline). The animals were used for *in vivo* studies when the tumor size reached 100-150 mm^3 (2-3 week after inoculation).

12.2 B16 cells xenograft animal model

Melanoma cell line B16 were obtained from were obtained from China Infrastructure of Cell Line Resources. The cells were cultured in DMEM medium supplemented with 10% (v/v) heat-inactivated fetal bovine serum (GIBCO) and penicillin/streptomycin (concentration of 100 U mL^{-1} for each). Cells were grown as a monolayer at 37 °C in a humidified atmosphere containing 5% CO_2.

5 to 7-week-old female nude mice were injected subcutaneously into the right front flanks with B16 cells (1×10^7 in 200 μL of phosphate-buffered saline). The animals were used for *in vivo* studies when the tumor size reached 100–150 mm^3 (2–3 week after inoculation).

12.3 4T1 cells xenograft animal model

Human breast cancer cell line 4T1 were obtained from China Infrastructure of Cell Line Resources. The cells were cultured in DMEM medium supplemented with 10% (v/v) heat-inactivated fetal bovine serum (GIBCO) and penicillin/streptomycin (concentration of 100 U mL^{-1} for each). Cells were grown as a monolayer at 37 °C in a humidified atmosphere containing 5% CO_2.

5 to 7-week-old female ICR mice were injected subcutaneously into the right front flanks with 4T1 cells (2×10^5 in 200 μL of phosphate-buffered saline). The animals were used for *in vivo* studies when the tumor size reached 100−150 mm^3 (2−3 week after inoculation).

13 MicroPET/CT imaging

13.1 MicroPET/CT imaging with [^{18}F]FTP-c(RGDyK)

The 4T1 tumor bearing mice received an intravenous injection of [^{18}F]FTP-c(RGDyK) (4.11 MBq). At 15 min post injection, the animals were anesthetized under 2% isoflurane and subjected to microPET/CT scans. Uptakes (%ID g^{-1}) of major organs were calculated and reported as mean ± SD. Reconstruction of microPET images was performed with 3D OPMAP algorithm.

13.2 MicroPET/CT imaging with [^{18}F]FTP-HSA

Healthy mice received intravenous injection of [^{18}F]FTP-HSA (3.70 MBq). Anesthetization was conducted with 2.5% isoflurane during preparation and 1.5% during scans. 60 min dynamic microPET/CT scans were acquired by an Inveon microPET/CT (Siemens, Germany). PET images reconstruction was performed with 3D OPMAP algorithm. Uptakes (%ID g^{-1}) of major organs were calculated and reported as mean ± SD.

13.3 MicroPET/CT imaging with [^{18}F]FTP-5F7

The MDA-MB-453 tumor bearing mice received an intravenous injection of [^{18}F]FTP-5F7 (4.11 MBq). At 60 min post injection, the animals were anesthetized under 2% isoflurane and subjected to microPET/CT scans. Uptakes (%ID g^{-1}) of major organs were calculated and reported as mean ± SD. Reconstruction of PET images was performed with 3D OPMAP algorithm.

13.4 MicroPET/CT imaging with [^{18}F]FTP-Tyr

The B16 tumor bearing mice received an intravenous injection of [^{18}F]FTP-Tyr (5.5 MBq). Anesthetization was conducted with 2.5% isoflurane during preparation and 1.5% during scans. 60 min dynamic microPET/CT scans were acquired by an Inveon microPET/CT (Siemens, Germany). Reconstruction of PET images was performed with 3D OPMAP algorithm. Uptakes (%ID g^{-1}) of major organs were calculated and reported as mean ± SD.

13.5 MicroPET/CT imaging with [^{18}F]FTP-Pds-PEG

The 4T1 tumor bearing mice received an intravenous injection of [^{18}F]FTP-Pds-PEG (4.11 MBq). At 1 h, 2 h and 4 h post-injection, the animals were anesthetized under 2% isoflurane and subjected to microPET/CT scans. Uptakes (%ID g^{-1}) of major organs were calculated and reported as mean ± SD. Reconstruction of PET images was performed with 3D OPMAP algorithm.

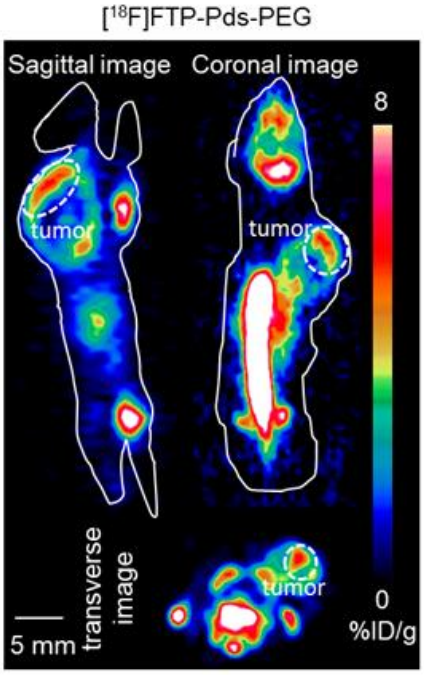

Figure S56. A PET image from a 2 h microPET/CT scans with [^{18}F]FTP-Pds-PEG in 4T1 tumor bearing mice. Tumor was indicated by the white ring.

14 Radio-TLC and RCC data for ^{18}F-labeled products in Figure 3

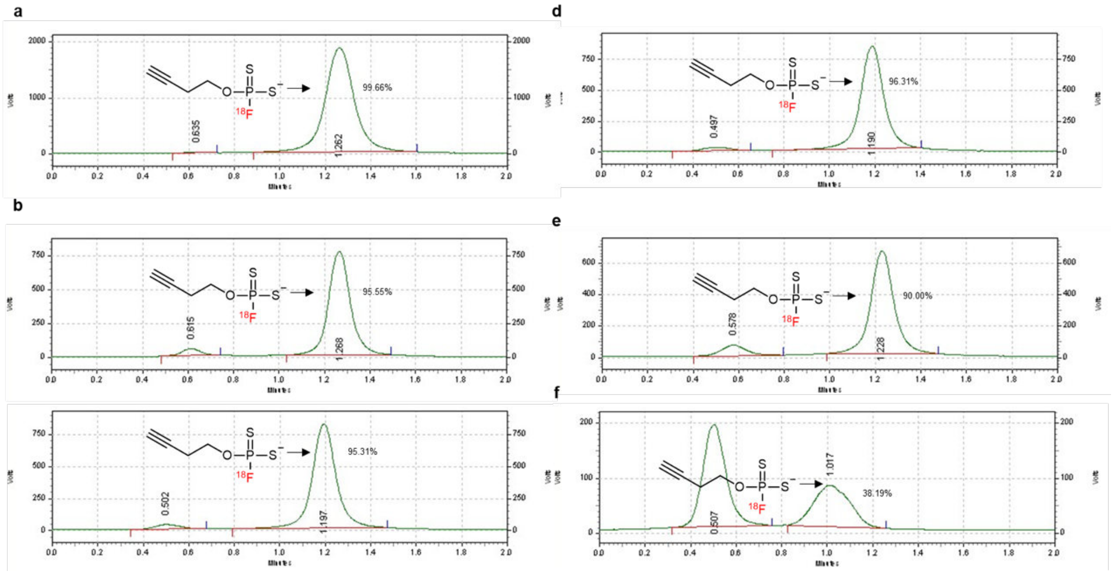

Figure S57. Radio-TLC analysis of RCCs of [^{18}F]**1**. (**a**) 0.1 mg precursor, [^{18}F]KF/K_{222} (1–5 mCi), 100 μL anhydrous acetonitrile, 80 °C, 30 sec. (**b**) 0.1 mg precursor, [^{18}F]KF/K_{222} (1–5 mCi), 100 μL anhydrous acetonitrile, RT, 30 sec. (**c**) 0.1 mg precursor, [^{18}F]F^- in cyclotron target water (1 μL, 0.3–0.5 mCi), 100 μL acetonitrile, RT, 30 sec. (**d**) 0.1 mg precursor, [^{18}F]F^- in cyclotron target water (3 μL, 1–3 mCi), 100 μL acetonitrile, RT, 30 sec. (**e**) 0.1 mg precursor, [^{18}F]F^- in cyclotron target water (5 μL, 1–5 mCi), 100 μL acetonitrile, RT, 30 sec. (**f**) 0.1 mg precursor, [^{18}F]F^- in cyclotron target water (10 μL, 1–5 mCi) and acetonitrile 90 μL, RT, 30 sec. Radio-TLC analyses were achieved on aluminum sheets coated with silica gel GF254 (2.5 cm × 5cm, Energy chemical). Developing solvent of radio-TLC was methanol/dichloromethane (1/1).

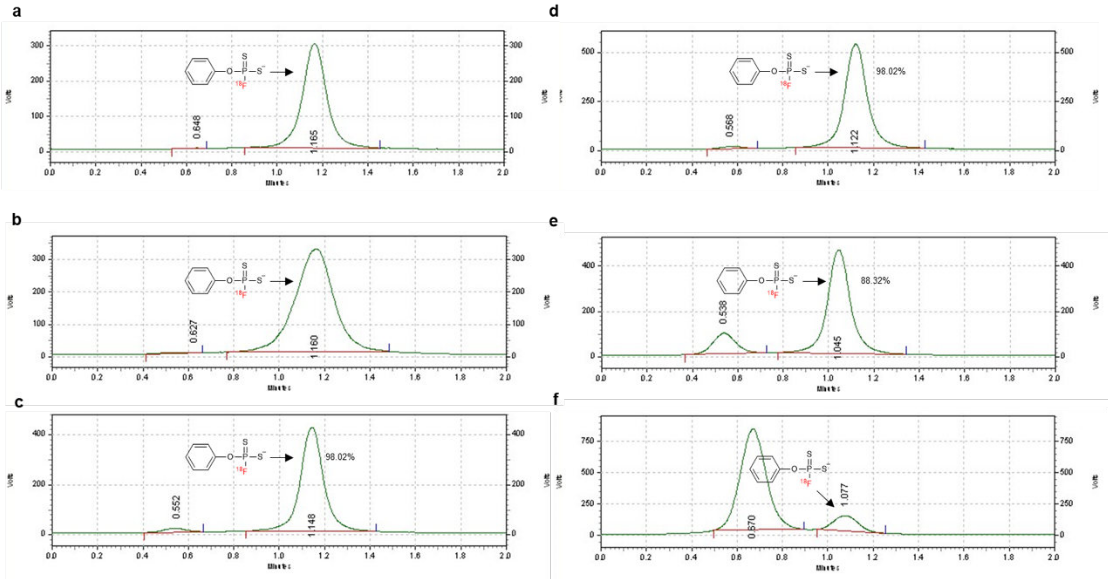

Figure S58. Radio-TLC analysis of RCCs of [^{18}F]**2**. (**a**) 0.1 mg precursor, [^{18}F]KF/K_{222} (1–5 mCi), 100 μL anhydrous acetonitrile, 80 °C, 30 sec. (**b**) 0.1 mg precursor, [^{18}F]KF/K_{222} (1–5 mCi), 100 μL anhydrous acetonitrile, RT, 30 sec. (**c**) 0.1 mg precursor, [^{18}F]F^{-} in cyclotron target water (1 μL, 0.3–0.5 mCi), 100 μL acetonitrile, RT, 30 sec. (**d**) 0.1 mg precursor, [^{18}F]F^{-} in cyclotron target water (3 μL, 1–3 mCi), 100 μL acetonitrile, RT, 30 sec. (**e**) 0.1 mg precursor, [^{18}F]F^{-} in cyclotron target water (5 μL, 1–5 mCi), 100 μL acetonitrile, RT, 30 sec. (**f**) 0.1 mg precursor, [^{18}F]F^{-} in cyclotron target water (10 μL, 1–5 mCi) and acetonitrile 90 μL, RT, 30 sec. Radio-TLC analyses were achieved on aluminum sheets coated with silica gel GF254 (2.5 cm × 5 cm, Energy chemical). Developing solvent of radio-TLC was methanol/dichloromethane (1/1).

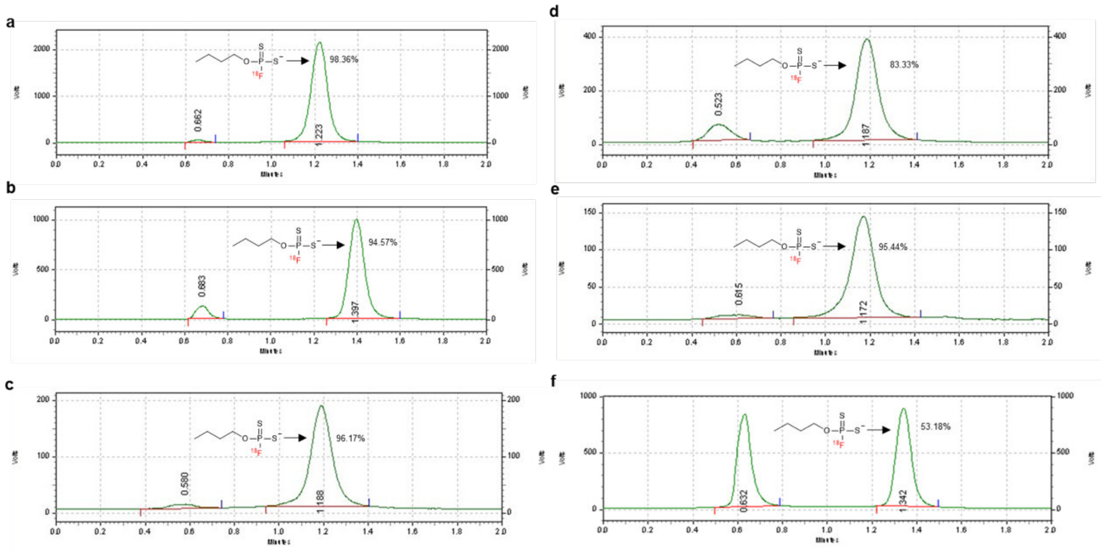

Figure S59. Radio-TLC analysis of RCCs of [^{18}F]**5**. (**a**) 0.1 mg precursor, [^{18}F]KF/K_{222} (1–5 mCi), 100 μL anhydrous acetonitrile, 80 °C, 30 sec. (**b**) 0.1 mg precursor, [^{18}F]KF/K_{222} (1–5 mCi), 100 μL anhydrous acetonitrile, RT, 30 sec. (**c**) 0.1 mg precursor, [^{18}F]F^- in cyclotron target water (1 μL, 0.3–0.5 mCi), 100 μL acetonitrile, RT, 30 sec. (**d**) 0.1 mg precursor, [^{18}F]F^- in cyclotron target water (3 μL, 1–3 mCi), 100 μL acetonitrile, RT, 30 sec. (**e**) 0.1 mg precursor, [^{18}F]F^- in cyclotron target water (5 μL, 1–5 mCi), 100 μL acetonitrile, RT, 30 sec. (**f**) 0.1 mg precursor, [^{18}F]F^- in cyclotron target water (10 μL, 1–5 mCi) and acetonitrile 90 μL, RT, 30 sec. Radio-TLC analyses were achieved on aluminum sheets coated with silica gel GF254 (2.5 cm × 5 cm, Energy chemical). Developing solvent of radio-TLC was methanol.

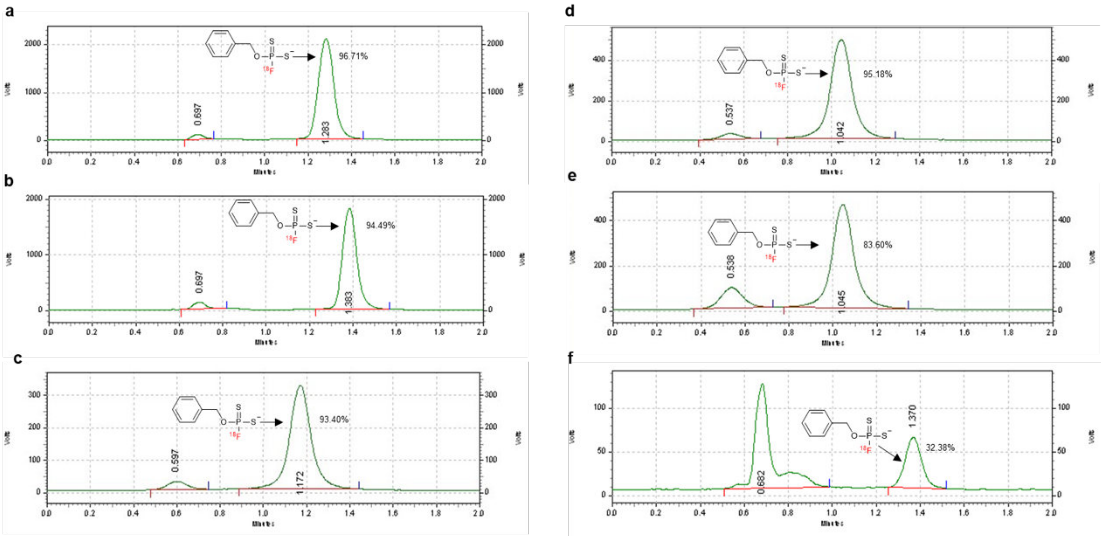

Figure S60. Radio-TLC analysis of RCCs of [^{18}F]**6**. (**a**) 0.1 mg precursor, [^{18}F]KF/K_{222} (1–5 mCi), 100 µL anhydrous acetonitrile, 80 °C, 30 sec. (**b**) 0.1 mg precursor, [^{18}F]KF/K_{222} (1–5 mCi), 100 µL anhydrous acetonitrile, RT, 30 sec. (**c**) 0.1 mg precursor, [^{18}F]$F^{–}$ in cyclotron target water (1 µL, 0.3–0.5 mCi), 100 µL acetonitrile, RT, 30 sec. (**d**) 0.1 mg precursor, [^{18}F]$F^{–}$ in cyclotron target water (3 µL, 1–3 mCi), 100 µL acetonitrile, RT, 30 sec. (**e**) 0.1 mg precursor, [^{18}F]$F^{–}$ in cyclotron target water (5 µL, 1–5 mCi), 100 µL acetonitrile, RT, 30 sec. (**f**) 0.1 mg precursor, [^{18}F]$F^{–}$ in cyclotron target water (10 µL, 1–5 mCi) and acetonitrile 90 µL, RT, 30 sec. Radio-TLC analyses were achieved on aluminum sheets coated with silica gel GF254 (2.5 cm × 5 cm, Energy chemical). Developing solvent of radio-TLC was methanol/dichloromethane (1/1).

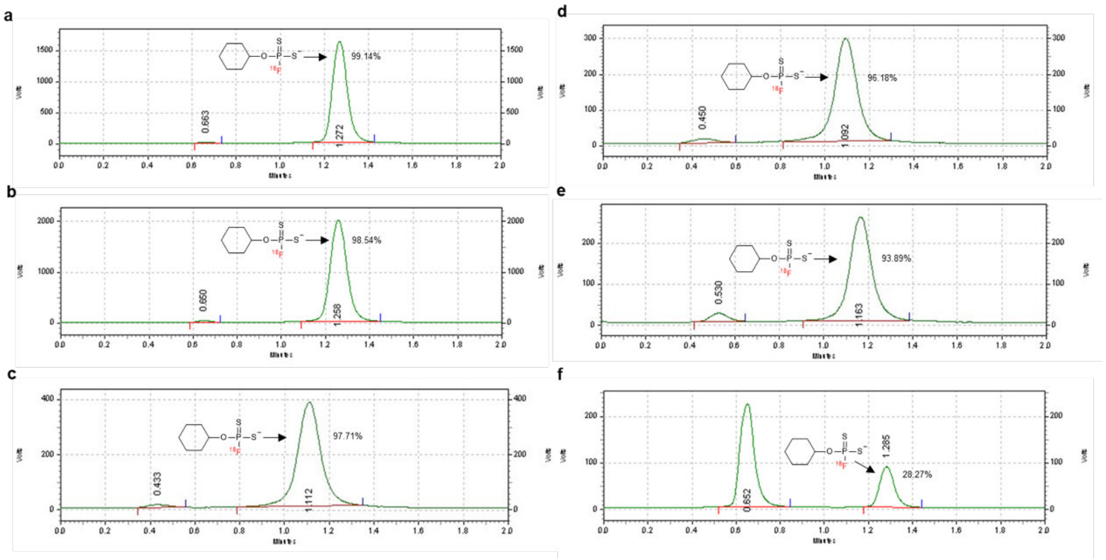

Figure S61. Radio-TLC analysis of RCCs of [^{18}F]**7**. (**a**) 0.1 mg precursor, [^{18}F]KF/K_{222} (1–5 mCi), 100 μL anhydrous acetonitrile, 80 °C, 30 sec. (**b**) 0.1 mg precursor, [^{18}F]KF/K_{222} (1–5 mCi), 100 μL anhydrous acetonitrile, RT, 30 sec. (**c**) 0.1 mg precursor, [^{18}F]F^{-} in cyclotron target water (1 μL, 0.3–0.5 mCi), 100 μL acetonitrile, RT, 30 sec. (**d**) 0.1 mg precursor, [^{18}F]F^{-} in cyclotron target water (3 μL, 1–3 mCi), 100 μL acetonitrile, RT, 30 sec. (**e**) 0.1 mg precursor, [^{18}F]F^{-} in cyclotron target water (5 μL, 1–5 mCi), 100 μL acetonitrile, RT, 30 sec. (**f**) 0.1 mg precursor, [^{18}F]F^{-} in cyclotron target water (10 μL, 1–5 mCi) and acetonitrile 90 μL, RT, 30 sec. Radio-TLC analyses were achieved on aluminum sheets coated with silica gel GF254 (2.5 cm × 5 cm, Energy chemical). Developing solvent of radio-TLC was methanol.

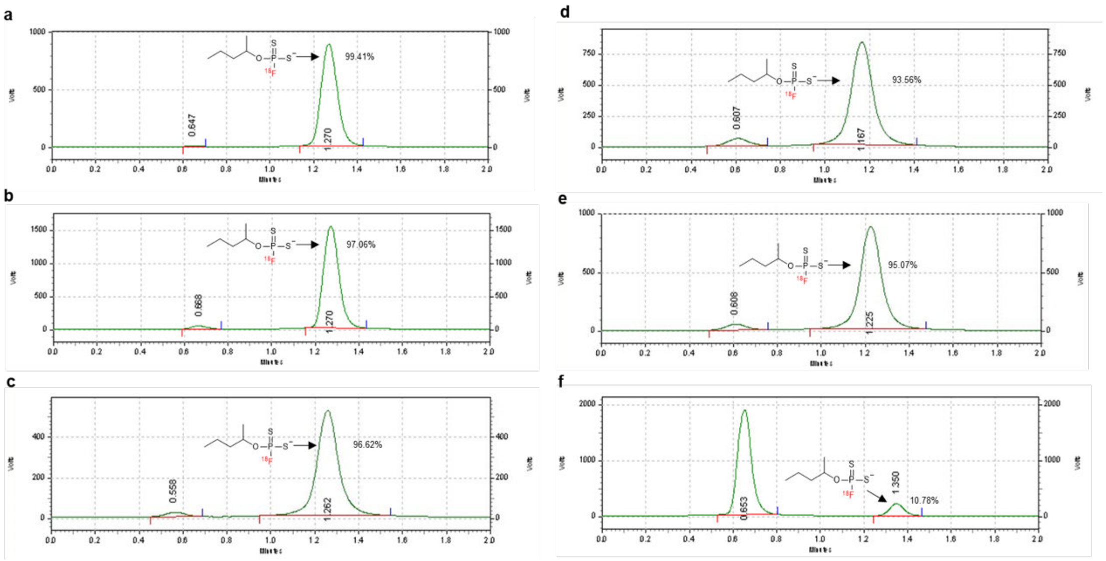

Figure S62. Radio-TLC analysis of RCCs of [^{18}F]**8**. (**a**) 0.1 mg precursor, [^{18}F]KF/K_{222} (1–5 mCi), 100 μL anhydrous acetonitrile, 80 °C, 30 sec. (**b**) 0.1 mg precursor, [^{18}F]KF/K_{222} (1–5 mCi), 100 μL anhydrous acetonitrile, RT, 30 sec. (**c**) 0.1 mg precursor, [^{18}F]F^{-} in cyclotron target water (1 μL, 0.3–0.5 mCi), 100 μL acetonitrile, RT, 30 sec. (**d**) 0.1 mg precursor, [^{18}F]F^{-} in cyclotron target water (3 μL, 1–3 mCi), 100 μL acetonitrile, RT, 30 sec. (**e**) 0.1 mg precursor, [^{18}F]F^{-} in cyclotron target water (5 μL, 1–5 mCi), 100 μL acetonitrile, RT, 30 sec. (**f**) 0.1 mg precursor, [^{18}F]F^{-} in cyclotron target water (10 μL, 1–5 mCi) and acetonitrile 90 μL, RT, 30 sec. Radio-TLC analyses were achieved on aluminum sheets coated with silica gel GF254 (2.5 cm × 5 cm, Energy chemical). Developing solvent of radio-TLC was methanol.

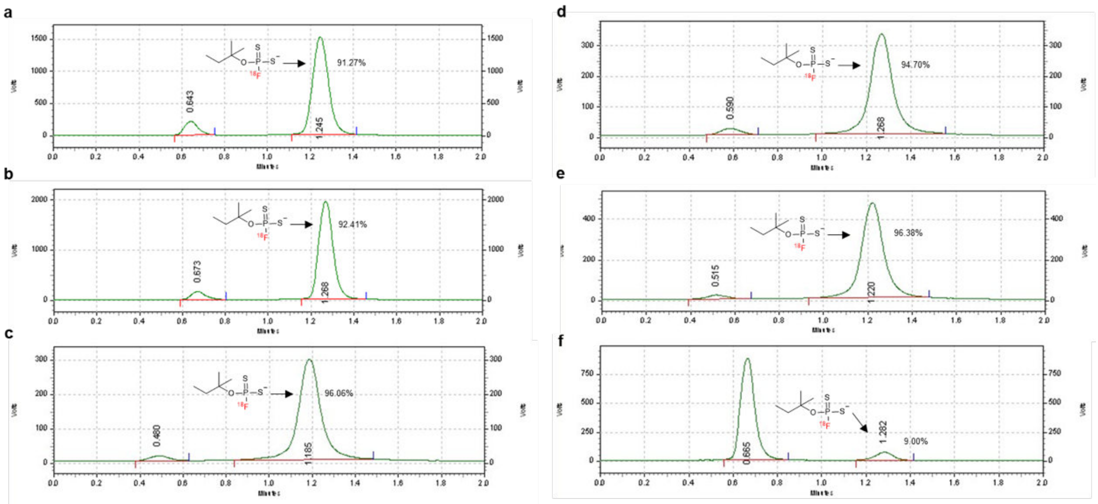

Figure S63. Radio-TLC analysis of RCCs of [^{18}F]**9**. (**a**) 0.1 mg precursor, [^{18}F]KF/K_{222} (1–5 mCi), 100 µL anhydrous acetonitrile, 80 ºC, 30 sec. (**b**) 0.1 mg precursor, [^{18}F]KF/K_{222} (1–5 mCi), 100 µL anhydrous acetonitrile, RT, 30 sec. (**c**) 0.1 mg precursor, [^{18}F]F^{-} in cyclotron target water (1 µL, 0.3–0.5 mCi), 100 µL acetonitrile, RT, 30 sec. (**d**) 0.1 mg precursor, [^{18}F]F^{-} in cyclotron target water (3 µL, 1–3 mCi), 100 µL acetonitrile, RT, 30 sec. (**e**) 0.1 mg precursor, [^{18}F]F^{-} in cyclotron target water (5 µL, 1–5 mCi), 100 µL acetonitrile, RT, 30 sec. (**f**) 0.1 mg precursor, [^{18}F]F^{-} in cyclotron target water (10 µL, 1–5 mCi) and acetonitrile 90 µL, RT, 30 sec. Radio-TLC analyses were achieved on aluminum sheets coated with silica gel GF254 (2.5 cm × 5 cm, Energy chemical). Developing solvent of radio-TLC was methanol.

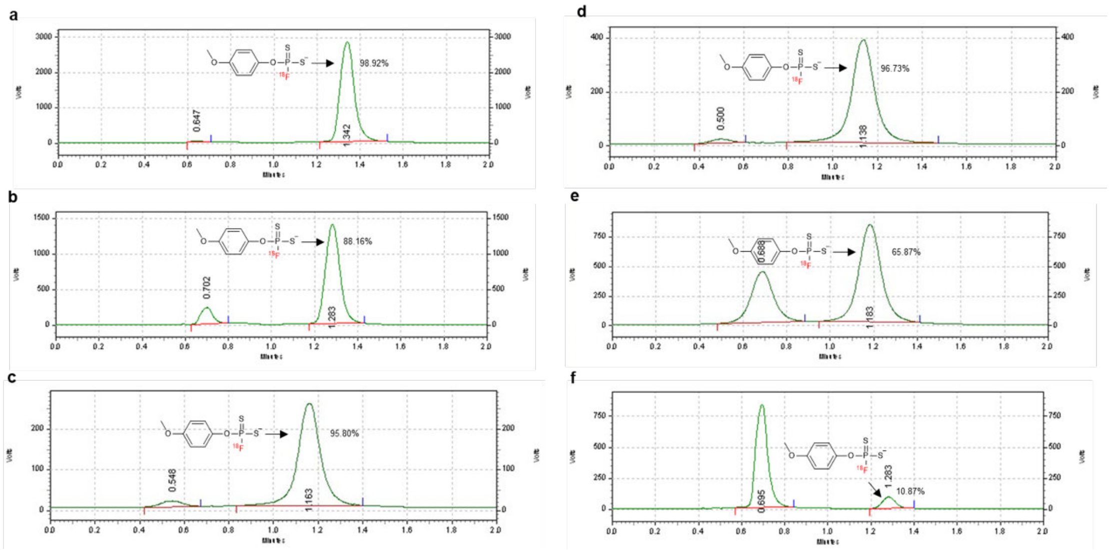

Figure S64. Radio-TLC analysis of RCCs of [^{18}F]**10**. (**a**) 0.1 mg precursor, [^{18}F]KF/K$_{222}$ (1–5 mCi), 100 μL anhydrous acetonitrile, 80 °C, 30 sec. (**b**) 0.1 mg precursor, [^{18}F]KF/K$_{222}$ (1–5 mCi), 100 μL anhydrous acetonitrile, RT, 30 sec. (**c**) 0.1 mg precursor, [^{18}F]F$^{–}$ in cyclotron target water (1 μL, 0.3–0.5 mCi), 100 μL acetonitrile, RT, 30 sec. (**d**) 0.1 mg precursor, [^{18}F]F$^{–}$ in cyclotron target water (3 μL, 1–3 mCi), 100 μL acetonitrile, RT, 30 sec. (**e**) 0.1 mg precursor, [^{18}F]F$^{–}$ in cyclotron target water (5 μL, 1–5 mCi), 100 μL acetonitrile, RT, 30 sec. (**f**) 0.1 mg precursor, [^{18}F]F$^{–}$ in cyclotron target water (10 μL, 1–5 mCi) and acetonitrile 90 μL, RT, 30 sec. Radio-TLC analyses were achieved on aluminum sheets coated with silica gel GF254 (2.5 cm × 5 cm, Energy chemical). Developing solvent of radio-TLC was methanol.

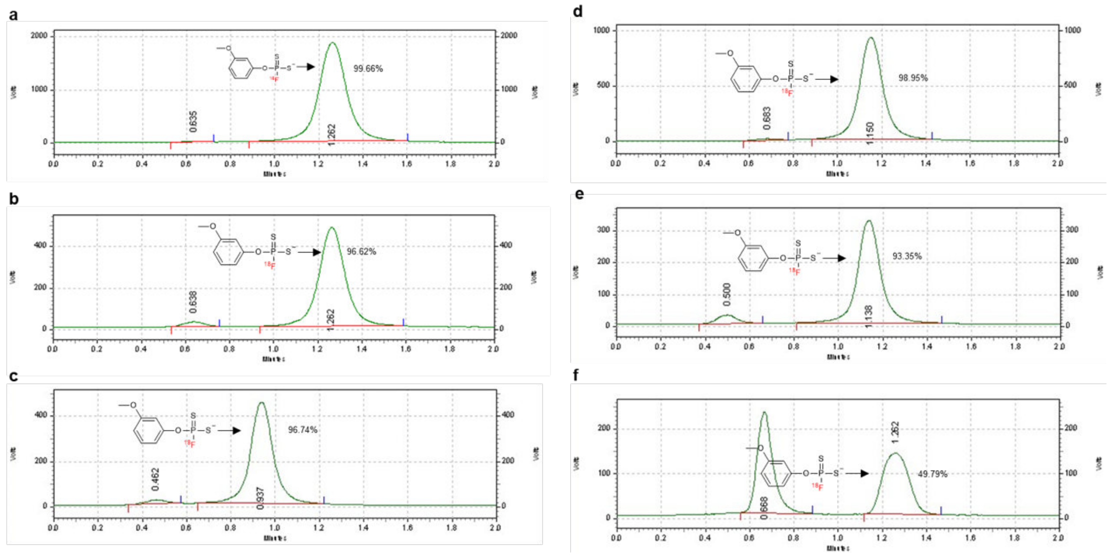

Figure S65. Radio-TLC analysis of RCCs of [^{18}F]**11**. (**a**) 0.1 mg precursor, [^{18}F]KF/K_{222} (1–5 mCi), 100 µL anhydrous acetonitrile, 80 °C, 30 sec. (**b**) 0.1 mg precursor, [^{18}F]KF/K_{222} (1–5 mCi), 100 µL anhydrous acetonitrile, RT, 30 sec. (**c**) 0.1 mg precursor, [^{18}F]F^{-} in cyclotron target water (1 µL, 0.3–0.5 mCi), 100 µL acetonitrile, RT, 30 sec. (**d**) 0.1 mg precursor, [^{18}F]F^{-} in cyclotron target water (3 µL, 1–3 mCi), 100 µL acetonitrile, RT, 30 sec. (**e**) 0.1 mg precursor, [^{18}F]F^{-} in cyclotron target water (5 µL, 1–5 mCi), 100 µL acetonitrile, RT, 30 sec. (**f**) 0.1 mg precursor, [^{18}F]F^{-} in cyclotron target water (10 µL, 1-5 mCi) and acetonitrile 90 µL, RT, 30 sec. Radio-TLC analyses were achieved on aluminum sheets coated with silica gel GF254 (2.5 cm × 5 cm, Energy chemical). Developing solvent of radio-TLC was methanol.

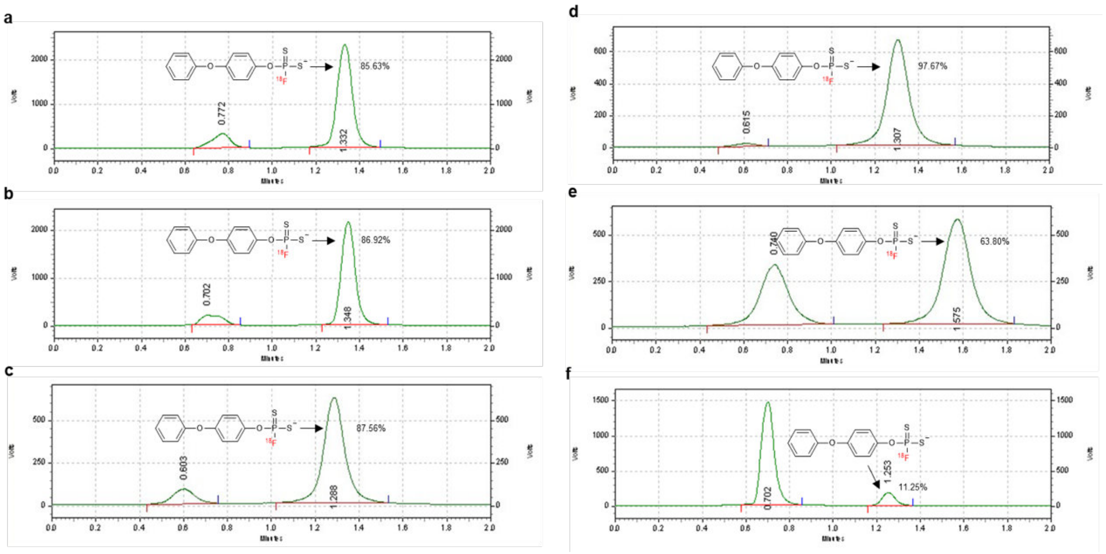

Figure S66. Radio-TLC analysis of RCCs of [^{18}F]**12**. (**a**) 0.1 mg precursor, [^{18}F]KF/K_{222} (1–5 mCi), 100 μL anhydrous acetonitrile, 80 °C, 30 sec. (**b**) 0.1 mg precursor, [^{18}F]KF/K_{222} (1–5 mCi), 100 μL anhydrous acetonitrile, RT, 30 sec. (**c**) 0.1 mg precursor, [^{18}F]F^- in cyclotron target water (1 μL, 0.3–0.5 mCi), 100 μL acetonitrile, RT, 30 sec. (**d**) 0.1 mg precursor, [^{18}F]F^- in cyclotron target water (3 μL, 1–3 mCi), 100 μL acetonitrile, RT, 30 sec. (**e**) 0.1 mg precursor, [^{18}F]F^- in cyclotron target water (5 μL, 1–5 mCi), 100 μL acetonitrile, RT, 30 sec. (**f**) 0.1 mg precursor, [^{18}F]F^- in cyclotron target water (10 μL, 1–5 mCi) and acetonitrile 90 μL, RT, 30 sec. Radio-TLC analyses were achieved on aluminum sheets coated with silica gel GF254 (2.5 cm × 5 cm, Energy chemical). Developing solvent of radio-TLC was methanol.

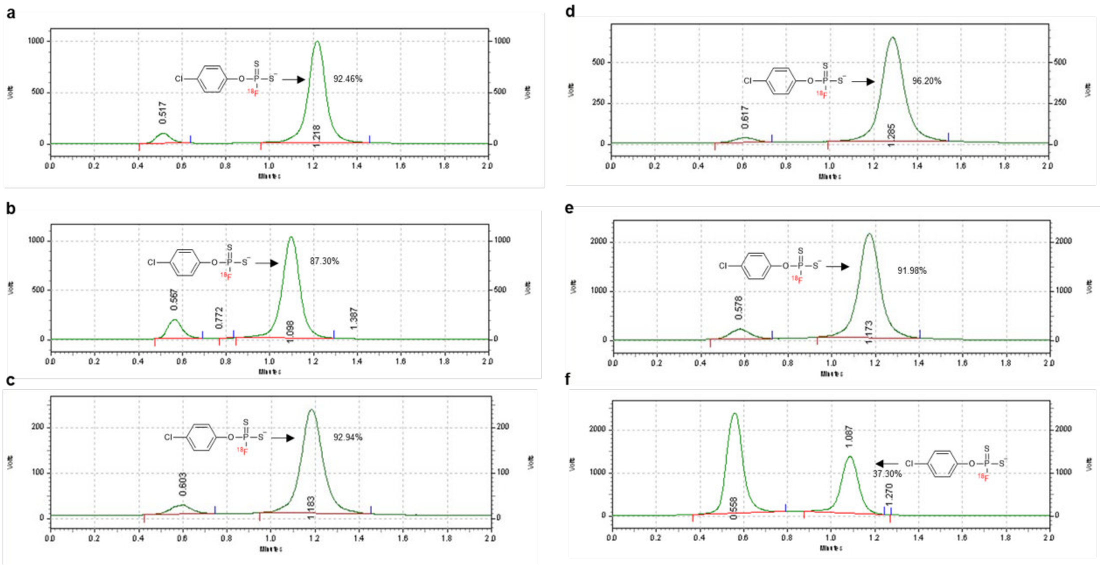

Figure S67. Radio-TLC analysis of RCCs of [^{18}F]**13**. (**a**) 0.1 mg precursor, [^{18}F]KF/K$_{222}$ (1–5 mCi), 100 µL anhydrous acetonitrile, 80 °C, 30 sec. (**b**) 0.1 mg precursor, [^{18}F]KF/K$_{222}$ (1–5 mCi), 100 µL anhydrous acetonitrile, RT, 30 sec. (**c**) 0.1 mg precursor, [^{18}F]F^{-} in cyclotron target water (1 µL, 0.3–0.5 mCi), 100 µL acetonitrile, RT, 30 sec. (**d**) 0.1 mg precursor, [^{18}F]F^{-} in cyclotron target water (3 µL, 1–3 mCi), 100 µL acetonitrile, RT, 30 sec. (**e**) 0.1 mg precursor, [^{18}F]F^{-} in cyclotron target water (5 µL, 1–5 mCi), 100 µL acetonitrile, RT, 30 sec. (**f**) 0.1 mg precursor, [^{18}F]F^{-} in cyclotron target water (10 µL, 1–5 mCi) and acetonitrile 90 µL, RT, 30 sec. Radio-TLC analyses were achieved on aluminum sheets coated with silica gel GF254 (2.5 cm × 5 cm, Energy chemical). Developing solvent of radio-TLC was methanol.

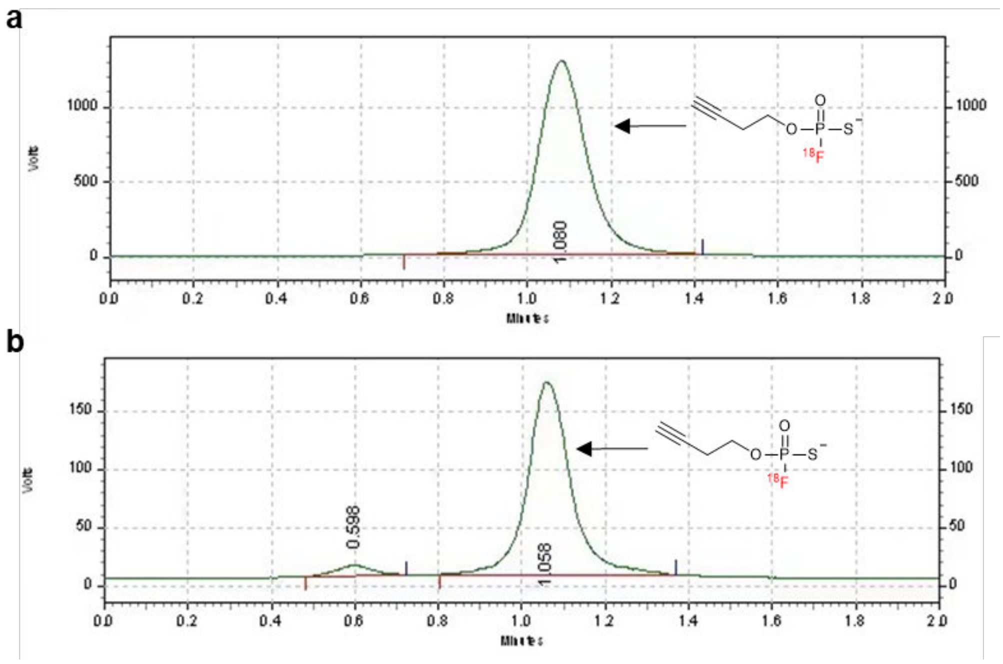

Figure S68. Radio-TLC analysis of RCCs of [^{18}F]**3**. (**a**) 0.1 mg precursor, [^{18}F]KF/K_{222} (1–5 mCi), 100 µL anhydrous acetonitrile, RT, 30 sec. (**b**) 0.1 mg precursor, [^{18}F]F^{-} in cyclotron target water (3 µL, 1–3 mCi), tetrabutylammonium hydroxide (0.52 mg), 100 µL acetonitrile, RT, 30 sec. Radio-TLC analyses were achieved on aluminum sheets coated with silica gel GF254 (2.5 cm × 5 cm, Energy chemical). Developing solvent was methanol.

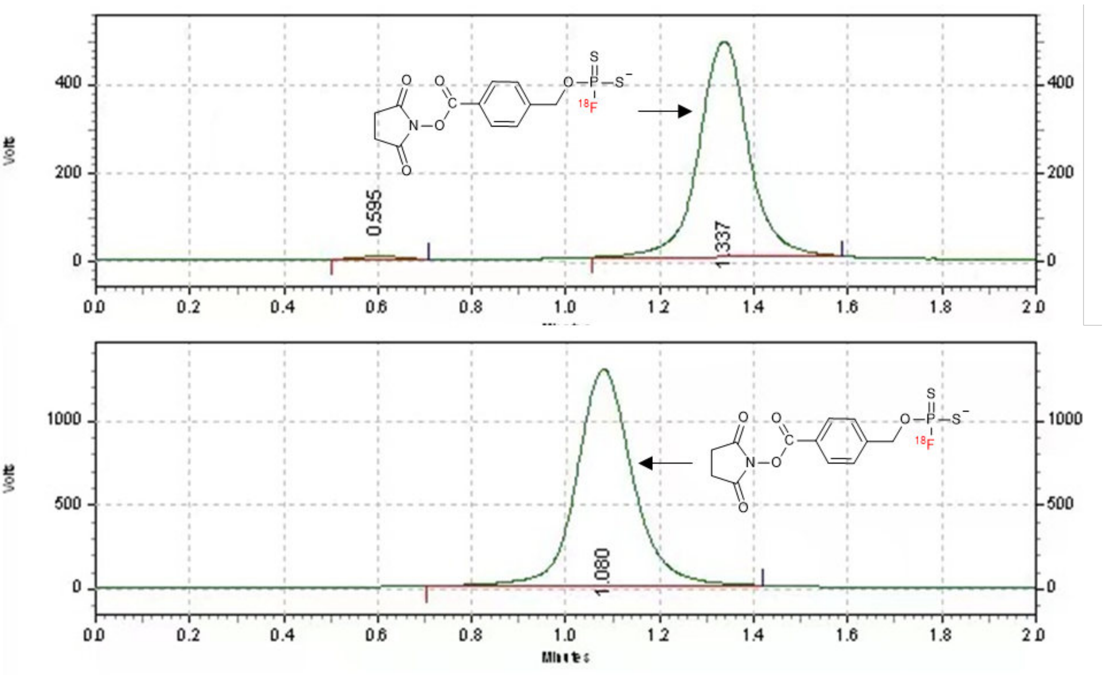

Figure S69. Radio-TLC analysis of RCCs of [^{18}F]**15**. (**a**) 0.1 mg precursor, [^{18}F]KF/K_{222} (1–5 mCi), 100 μL anhydrous acetonitrile, RT, 30 sec. (**b**) 0.1 mg precursor, [^{18}F]F^{-} in cyclotron target water (3 μL, 1–3 mCi), tetrabutylammonium hydroxide (0.52 mg), 100 μL acetonitrile, RT, 30 sec. Radio-TLC analyses were achieved on aluminum sheets coated with silica gel GF254 (2.5 cm × 5 cm, Energy chemical). Developing solvent was methanol.

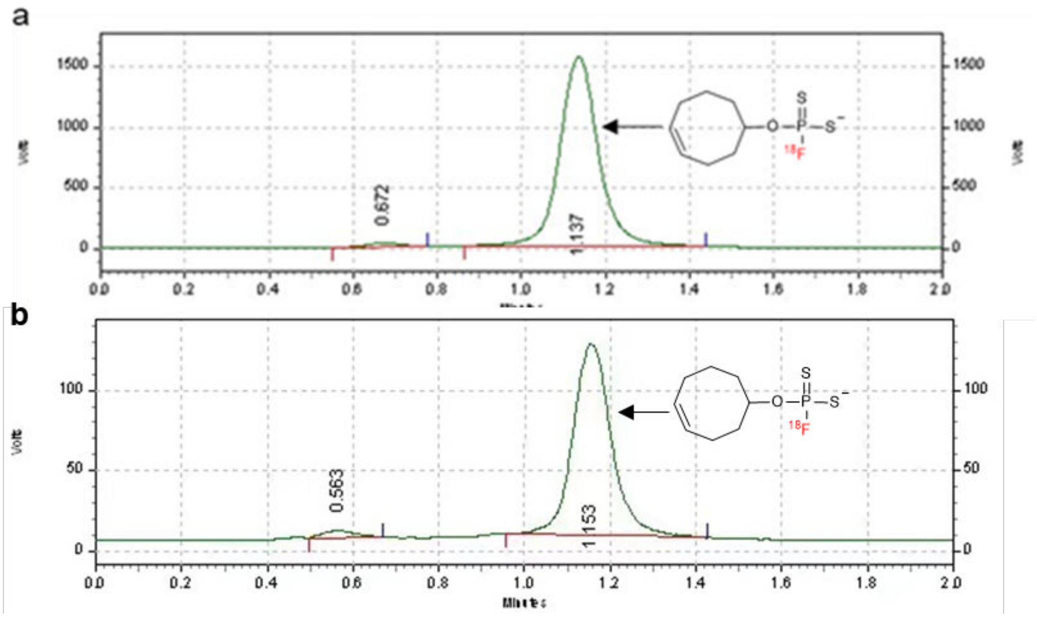

Figure S70. Radio-TLC analysis of RCCs of [^{18}F]**16**. (**a**) 0.1 mg precursor, [^{18}F]KF/K_{222} (1–5 mCi), 100 μL anhydrous acetonitrile, RT, 30 sec. (**b**) 0.1 mg precursor, [^{18}F]F^{-} in cyclotron target water (3 μL, 1–3 mCi), tetrabutylammonium hydroxide (0.52 mg), 100 μL acetonitrile, RT, 30 sec. Radio-TLC analyses were achieved on aluminum sheets coated with silica gel GF254 (2.5 cm × 5 cm, Energy chemical). Developing solvent was methanol.

Supplementary references

1 Olesiak, M. and Okruszek, A. Studies of asymmetric induction in the synthesis of dinucleoside phosphorothioates from 2-oxo-1,3,2-dithiaphospholane nucleoside derivatives. *Phosphorus Sulfur* **184**, 1548-1560, (2009).

2 Murai, T. et al. Fluorinative hydrolysis of phosphorothioic acid esters with a binaphthyl group through axis-to-center chirality transfer leading to the formation of P-chiral phosphorothioic monofluoridic acid salts. *Chem. Commun.* **50**, 12473-12475 (2014).

3 Rytczak, P., Koziołkiewicz, M., and Okruszek, A. The chemical synthesis of phosphorothioate and phosphorodithioate analogues of lysophosphatidic acid (LPA) and cyclic phosphatidic acid (CPA). *New J. Chem.* **34**, 1008-1017 (2010).

4 Vogel, K. et al. DNA-Modification of eukaryotic cells. *Small* **9**, 255-262 (2013).

5 Frisch M. J. et al. Gaussian 09, revision A.02, Gaussian, Inc., Wallingford, CT, (2009).

6 Zhurko, G. A., Aleksandriiskii, V. V., Burmistrov, V. A. Conformational state of benzilidene aniline derivatives from ab initio calculation and NMR spectroscopy data. *J. Struct. Chem.*2006, **47**, 622-628 (2006).

7 Scalmani, G., Frisch, M. J. Continuous surface charge polarizable continuum models of solvation. *J. Chem. Phys.* **132**, 114110-114124 (2010).

8 Grimme, S. Supramolecular Binding Thermodynamics by Dispersion-Corrected Density Functional Theory. *Chem. Eur. J.* **18**, 9955-9964 (2012) .

9 Mammen, M., Shakhnovich, E. I., Deutch, J. M., Whitesides, G. M. Estimating the Entropic Cost of Self-Assembly of Multiparticle Hydrogen-Bonded Aggregates Based on the Cyanuric Acid•Melamine Lattice. *J. Org. Chem.* **63**, 3821-3830 (1998).

10 Huang, F, et al. The catalytic role of *N*-heterocyclic carbene in a metal-free conversion of carbon dioxide into methanol: a computational mechanism study.

J. Am. Chem. Soc. **132**, 12388-12396 (2010).

11 Wang, C. et al. Direct ^{18}F-Labeling of biomolecules *via* spontaneous site-Specific nucleophilic substitution by F^{-} on phosphonate prostheses. *Org. Lett.* **4**, 4261-4266 (2021).

12 Wängler, C. et al. One-step ^{18}F-labeling of peptides for positron emission tomography imaging using the SiFA methodology. *Nat. Protoc.* **7**, 1946-1955 (2012).

13 Kraskouskaya, D., Duodu, E., Arpin, C. C., Gunning, P. T. Progress towards the development of SH2 domain inhibitors. *Chem. Soc. Rev.* **42**, 3337-3370 (2013).

14 Cao, Y. et al. ABHD10 is an S-depalmitoylase affecting redox homeostasis through peroxiredoxin-5. *Nat. Chem. Biol.* **15**, 1232-1240 (2019).

Appendix-Spectral data for new compounds

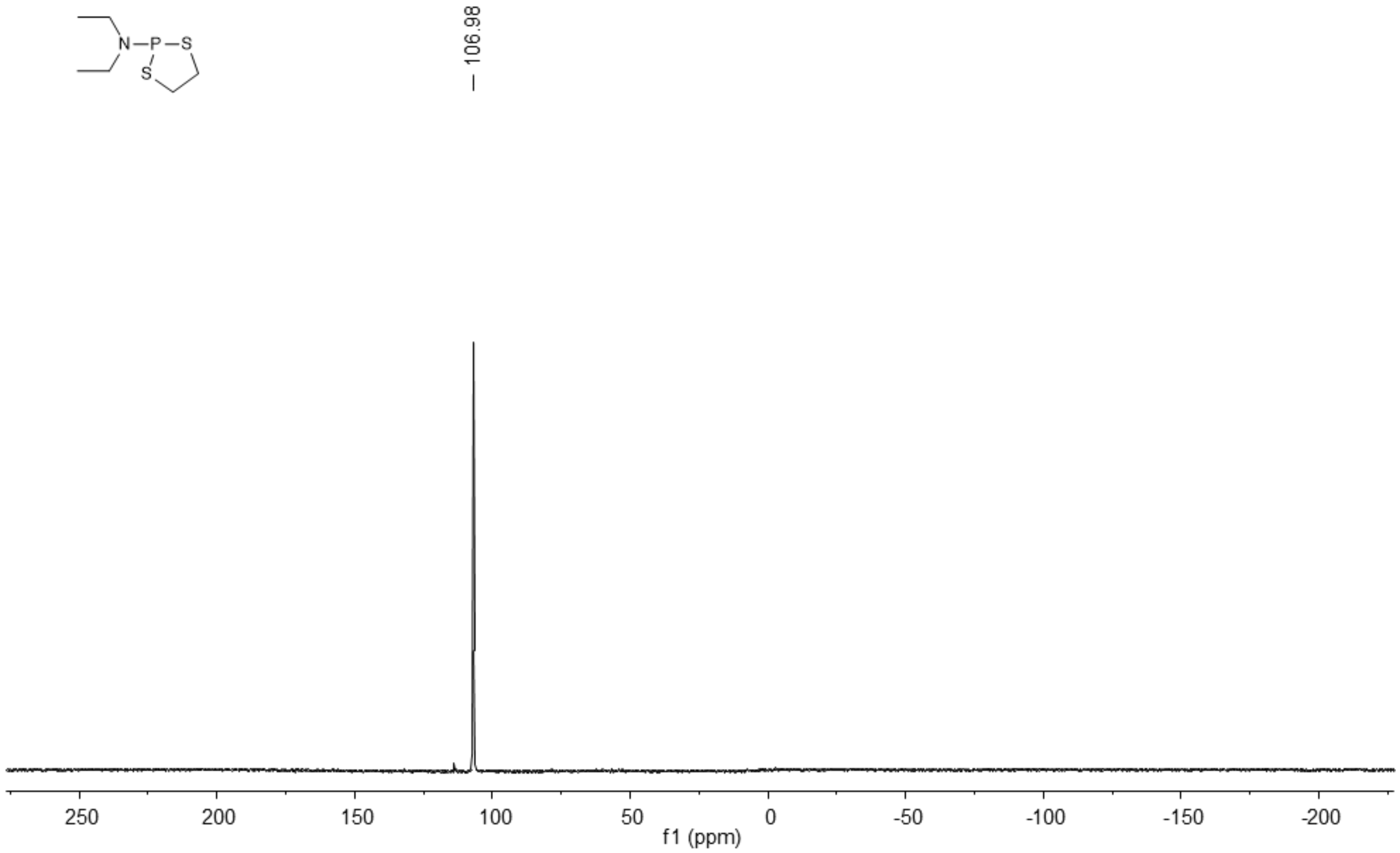

Figure A1. ^{31}P NMR (162 MHz, $CDCl_3$) spectrum of **19**.

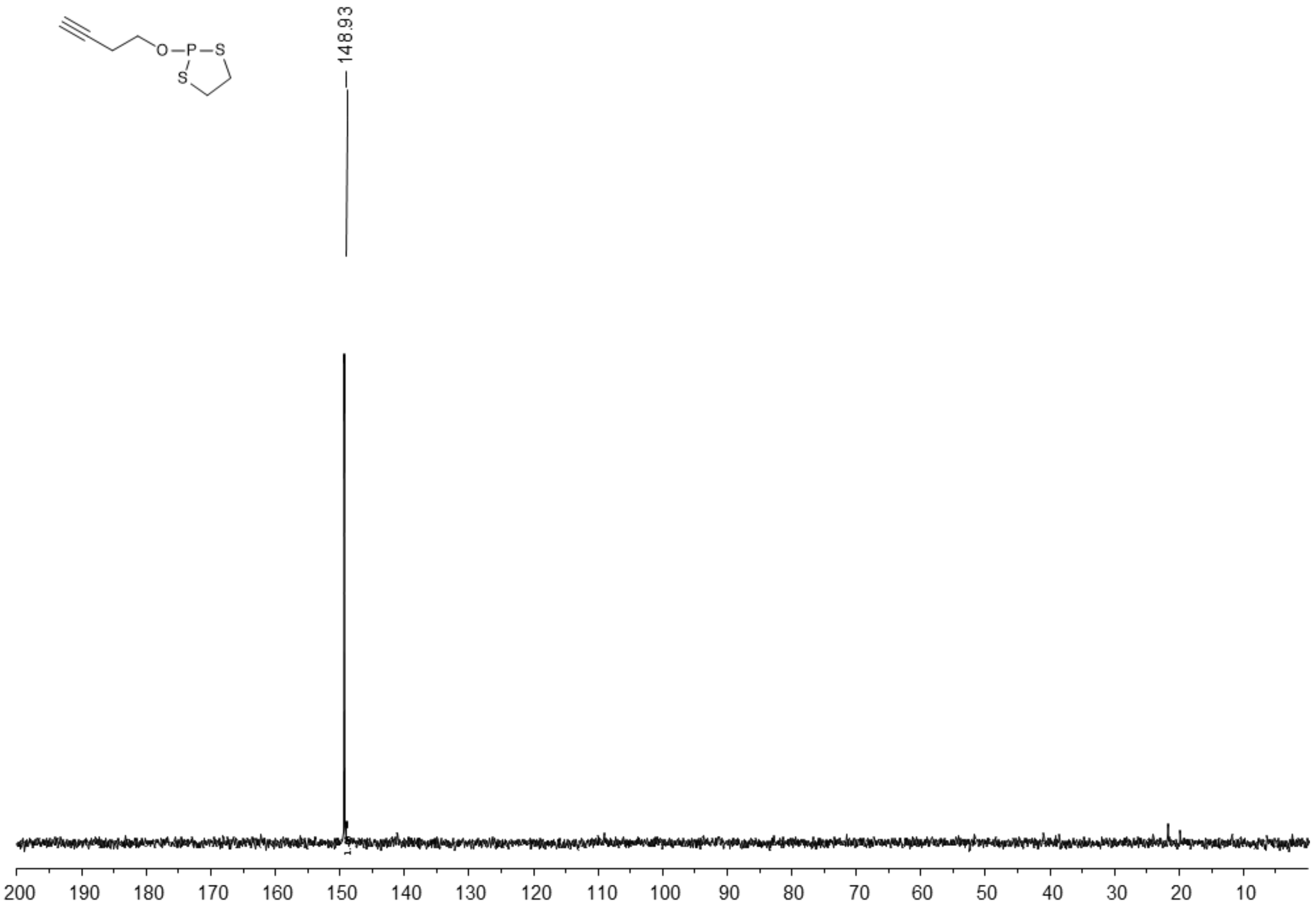

Figure A2. **^{31}P NMR (162 MHz, $CDCl_3$)** spectrum of **1a**.

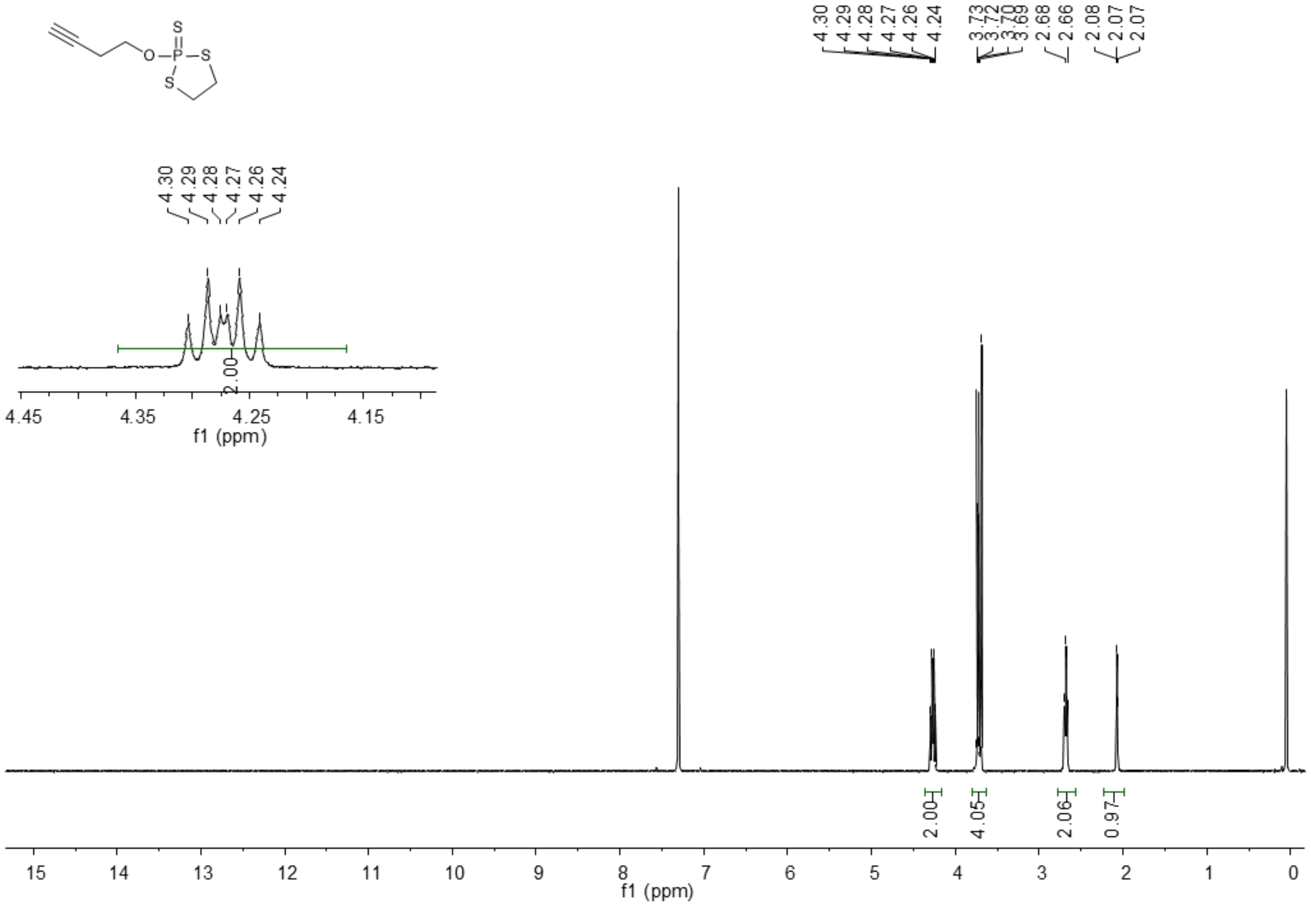

Figure A3. **^{1}H NMR (400 MHz, $CDCl_3$)** spectrum of **1b**.

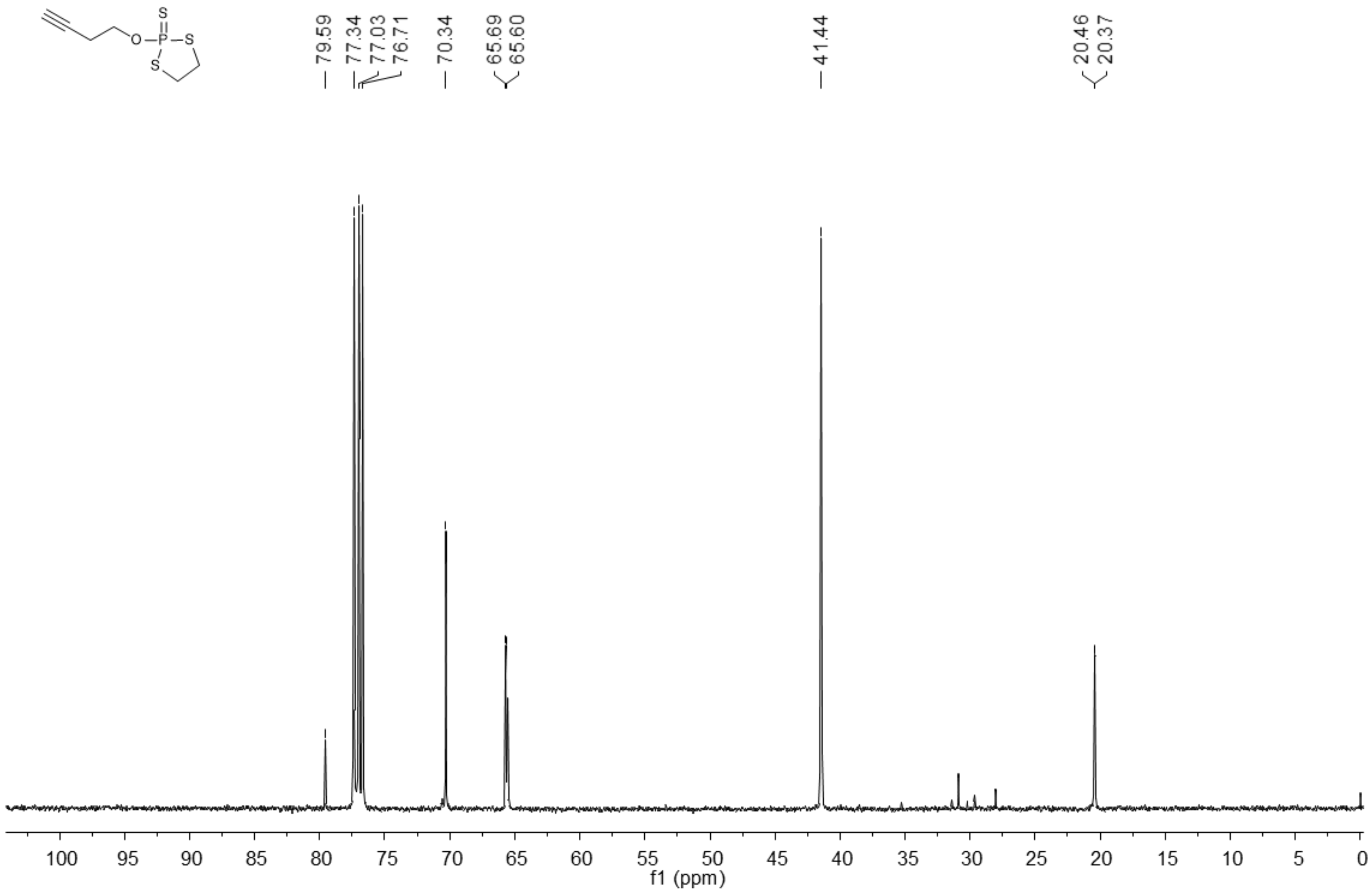

Figure A4. **^{13}C NMR (101 MHz, $CDCl_3$)** spectrum of **1b**.

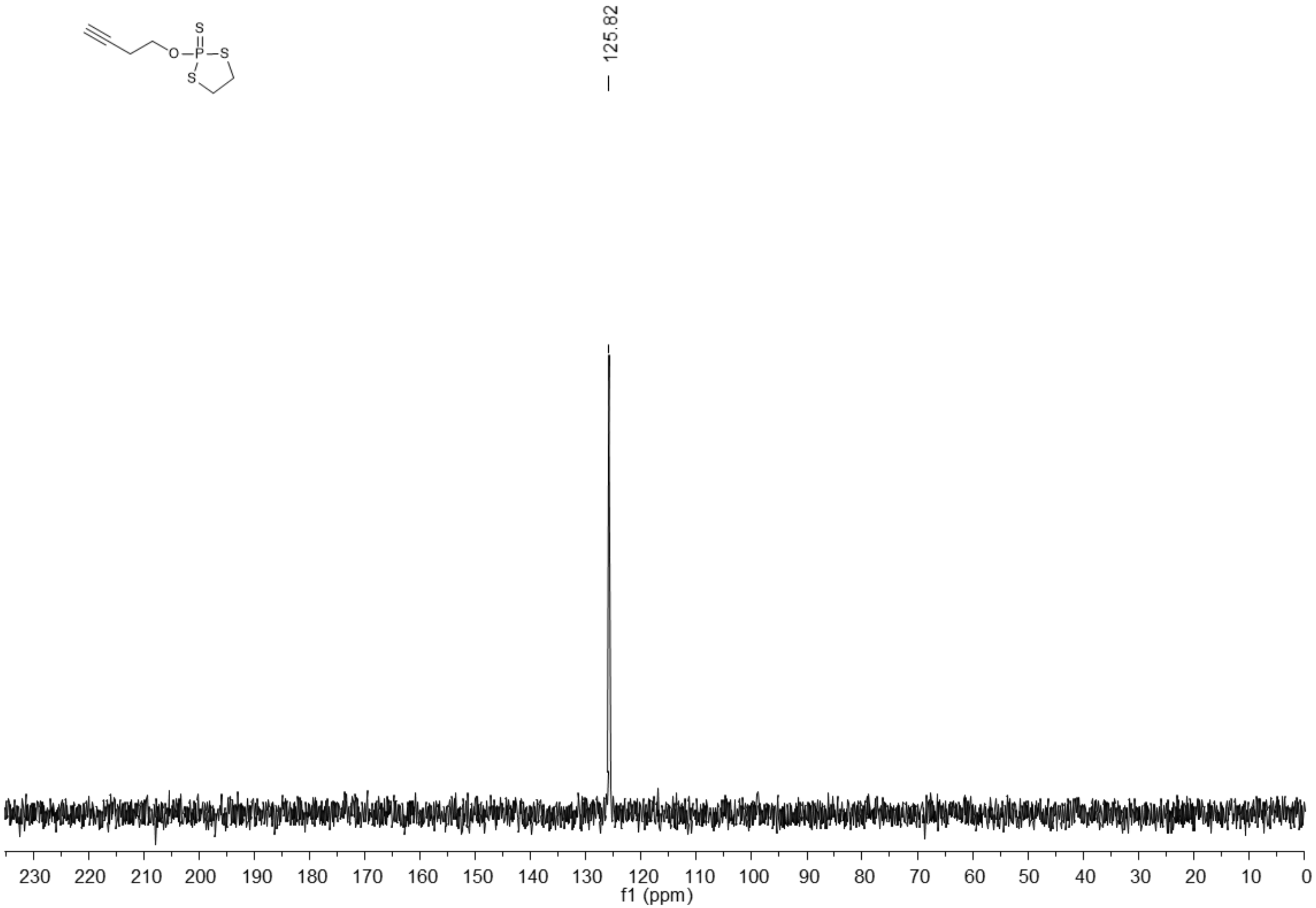

Figure A5. ^{31}**P NMR (162 MHz, CDCl$_3$)** spectrum of **1b**.

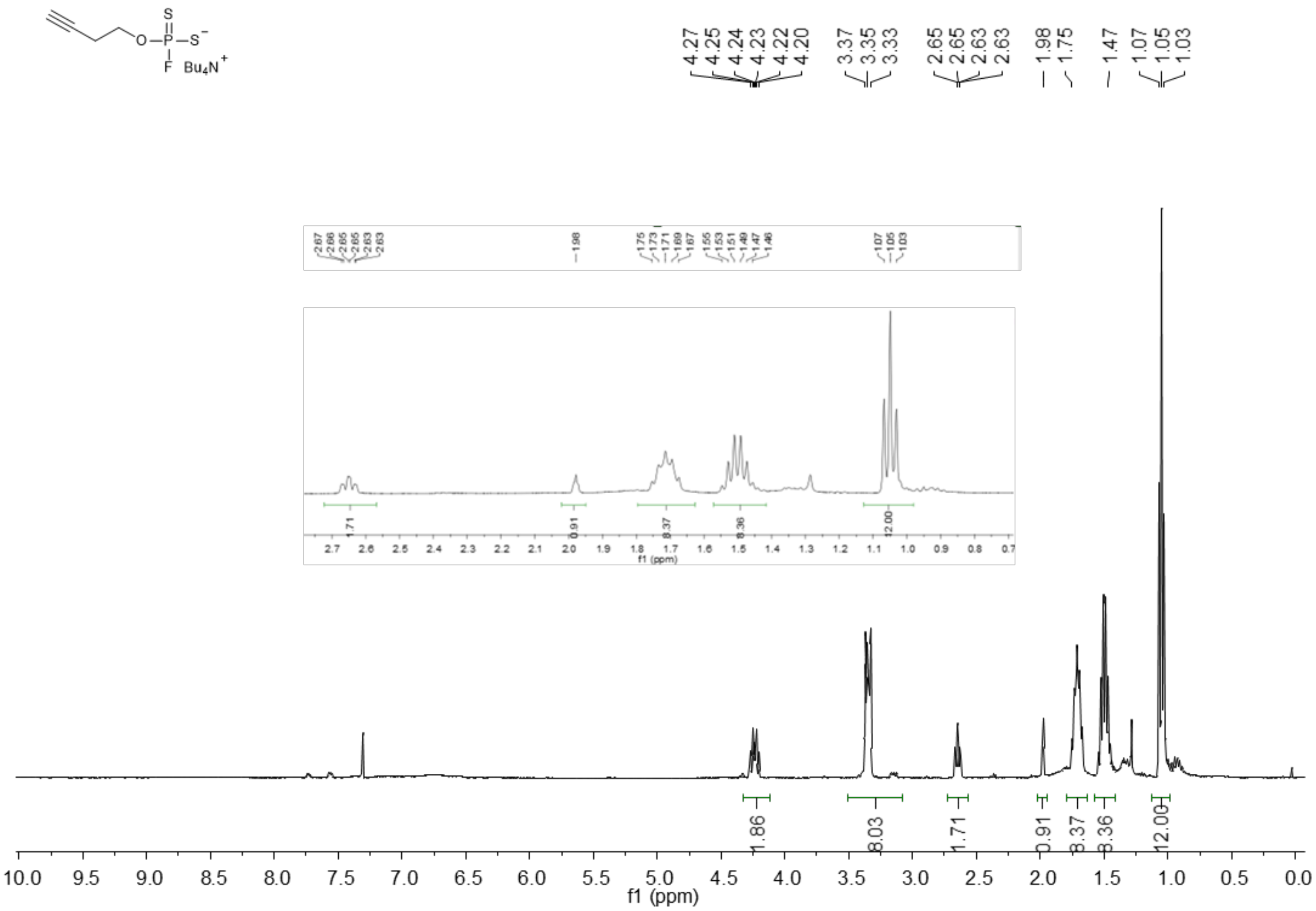

Figure A6. **^{1}H NMR (400 MHz, $CDCl_3$)** spectrum of **1**.

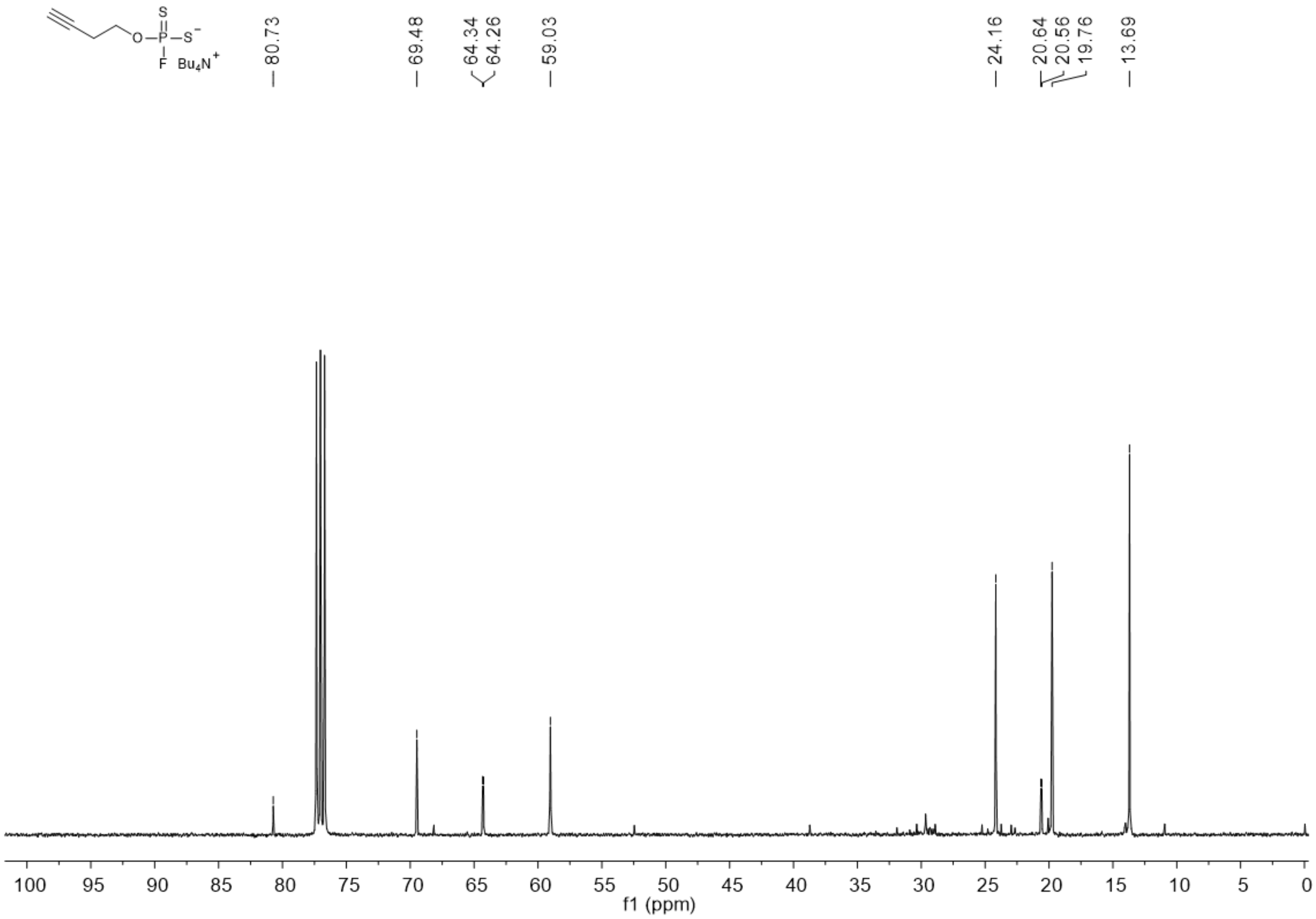

Figure A7. **^{13}C NMR (101 MHz, $CDCl_3$)** spectrum of **1**.

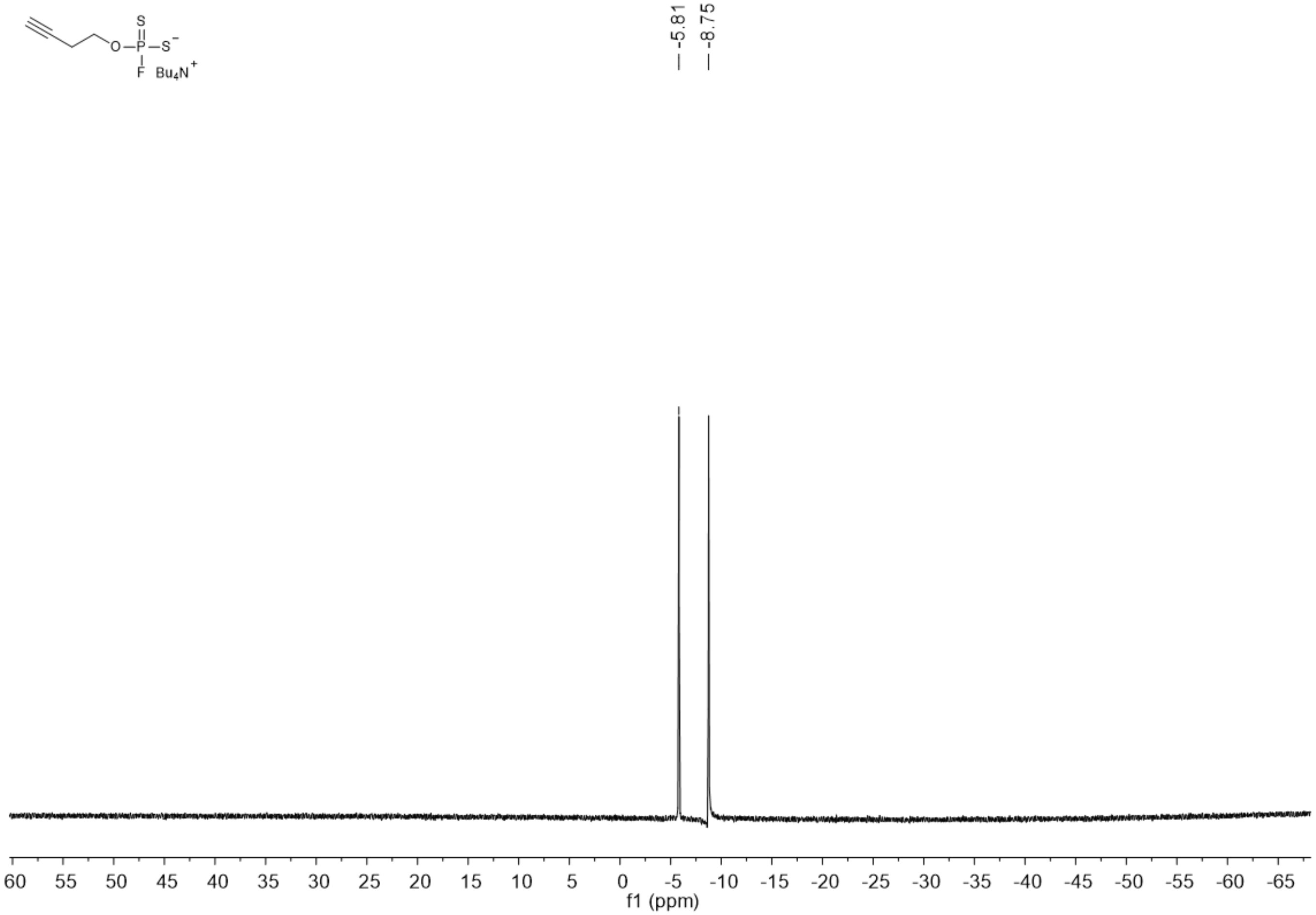

Figure A8. **^{19}F NMR (376 MHz, $CDCl_3$)** spectrum of **1**.

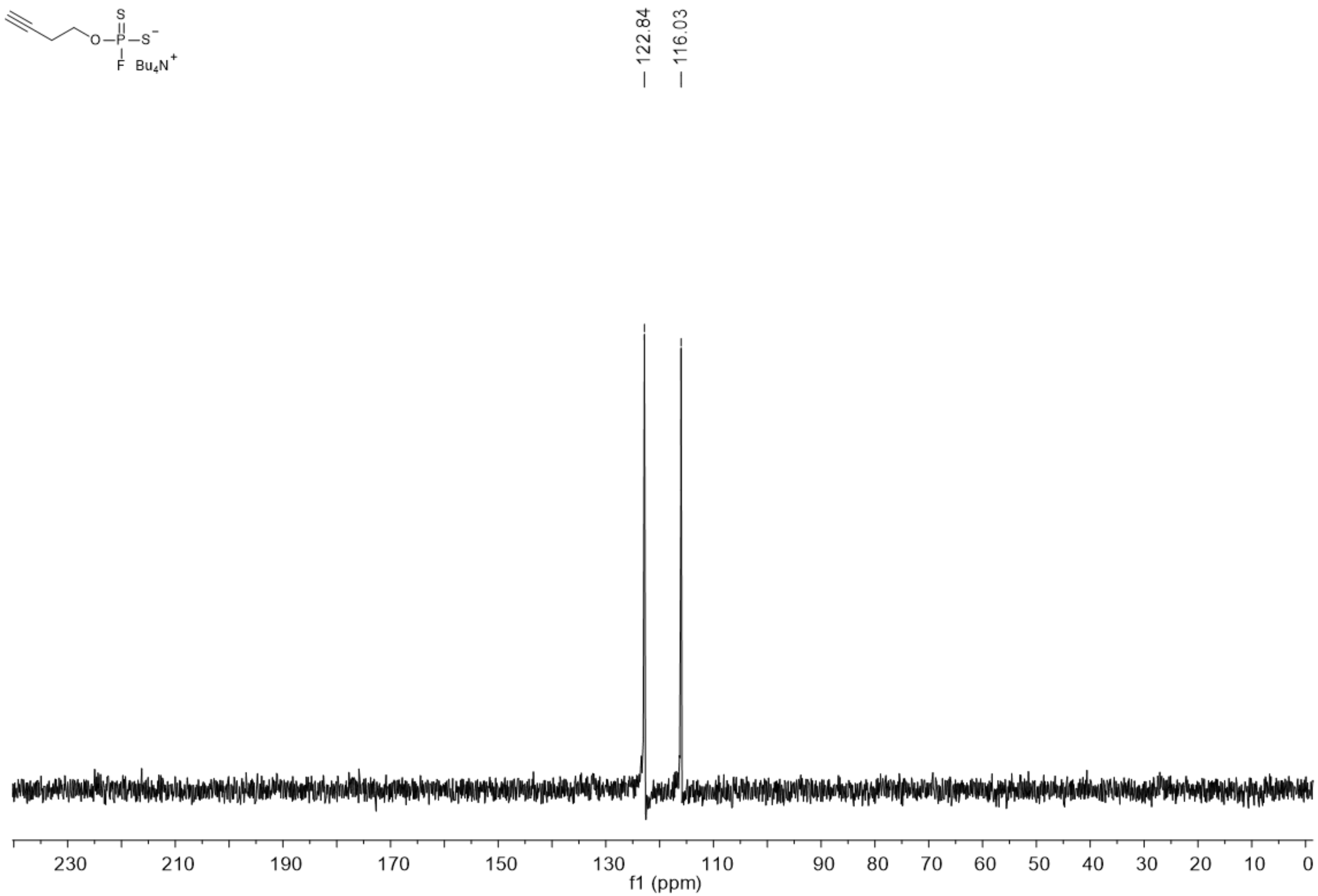

Figure A9. **^{31}P NMR (162 MHz, $CDCl_3$)** spectrum of **1**.

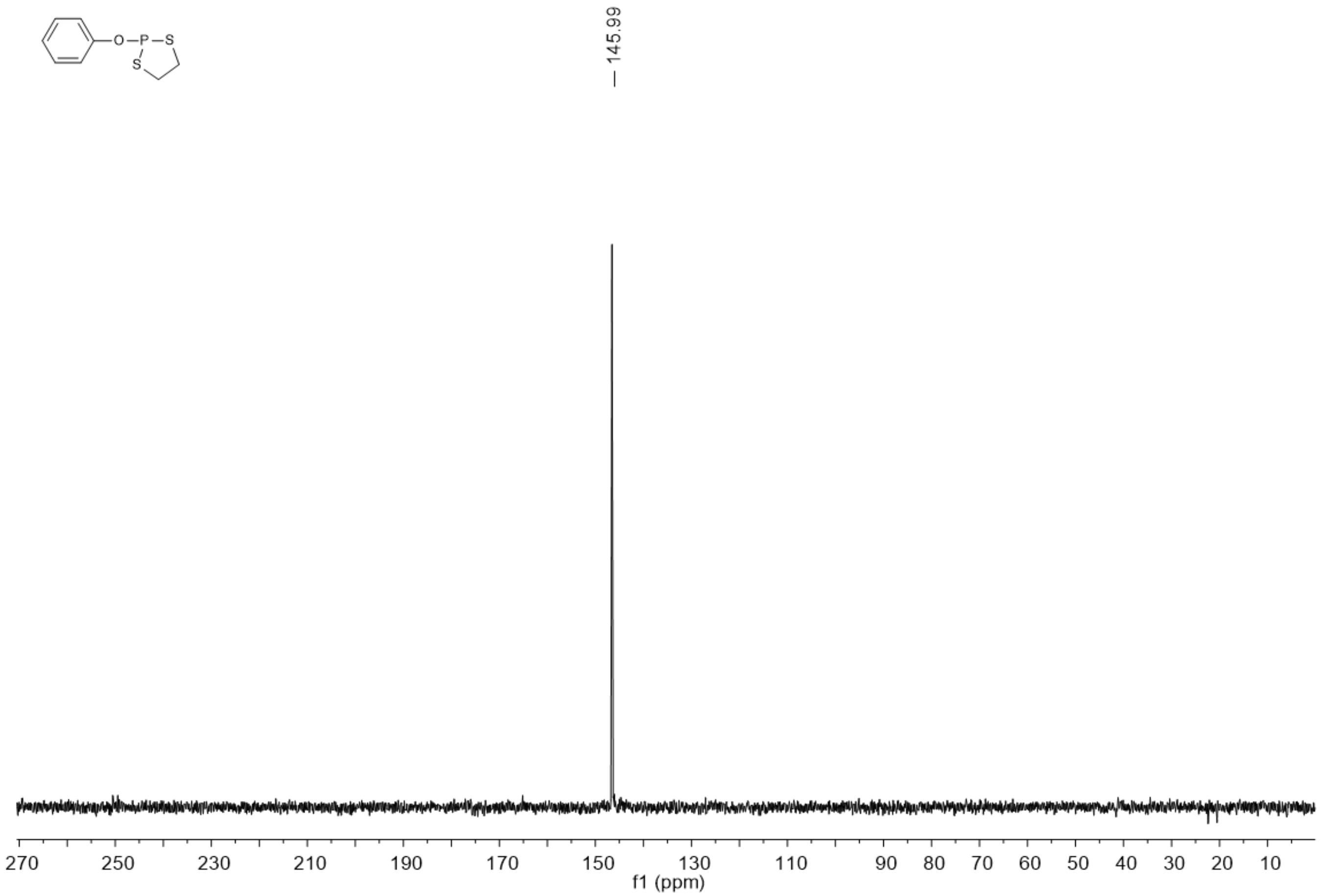

Figure A10. **^{31}P NMR (162 MHz, $CDCl_3$)** spectrum of **2a**.

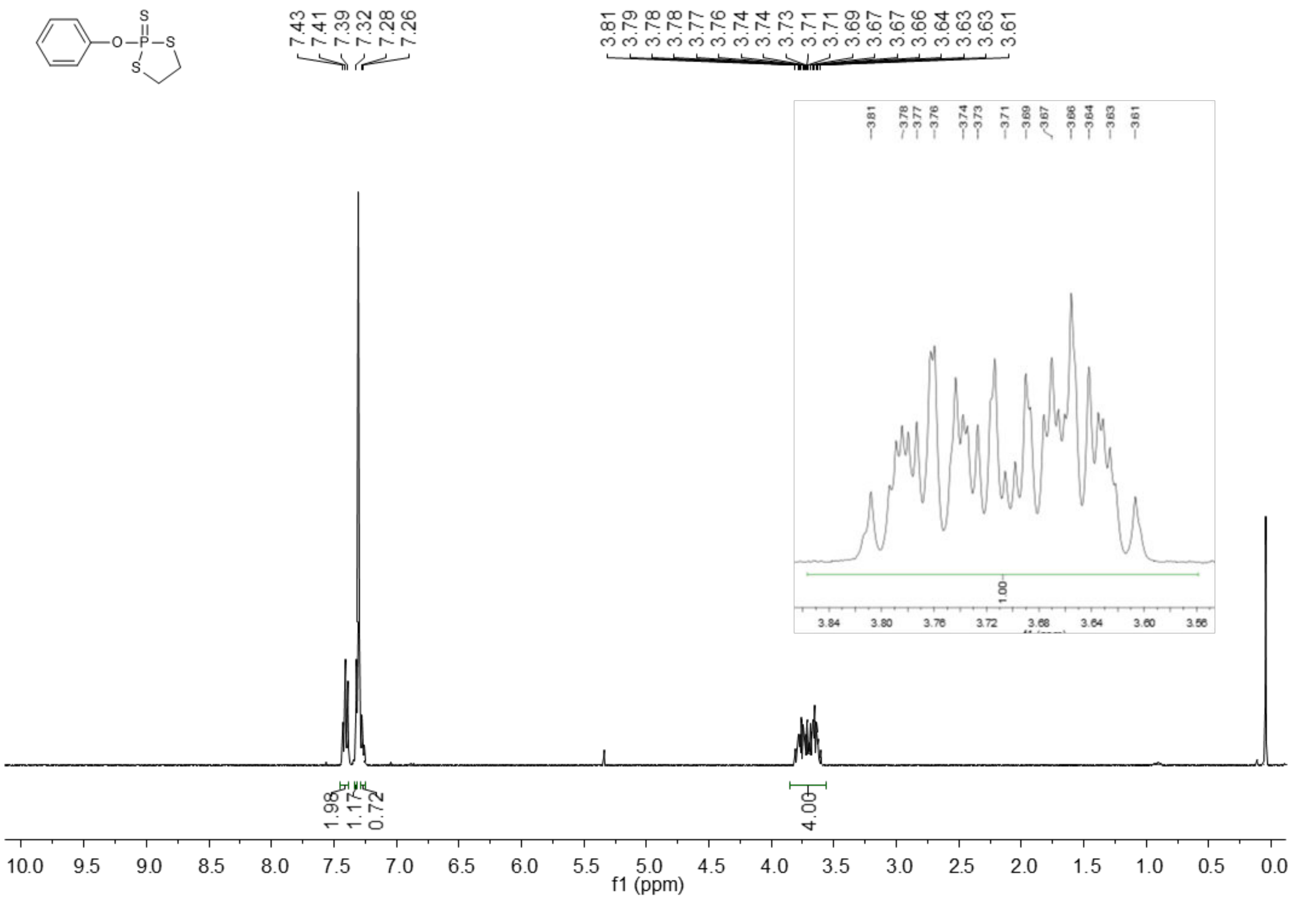

Figure A11. **^{1}H NMR (400 MHz, CDCl$_3$)** spectrum of **2b**.

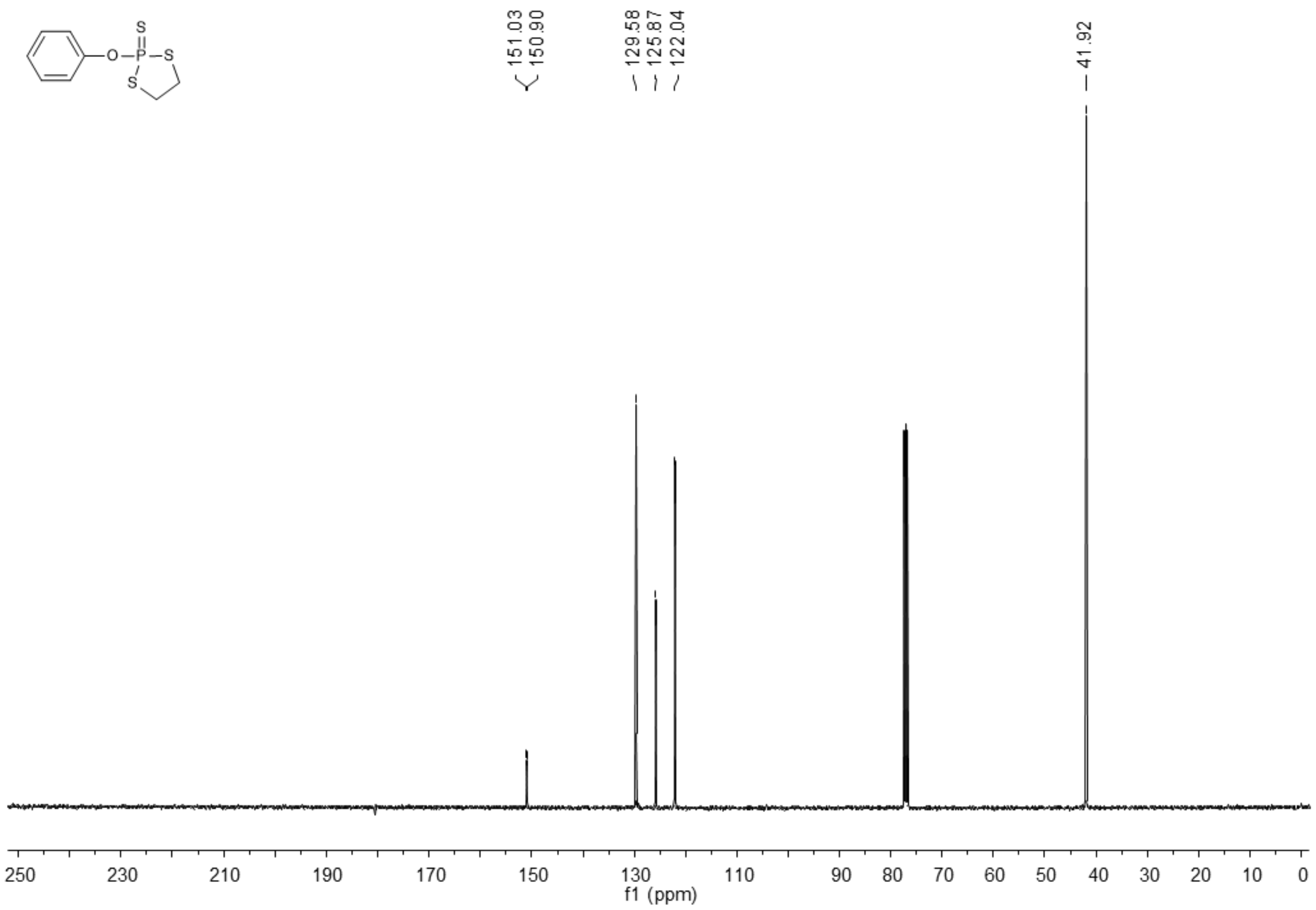

Figure A12. **^{13}C NMR (101 MHz, $CDCl_3$)** spectrum of **2b**.

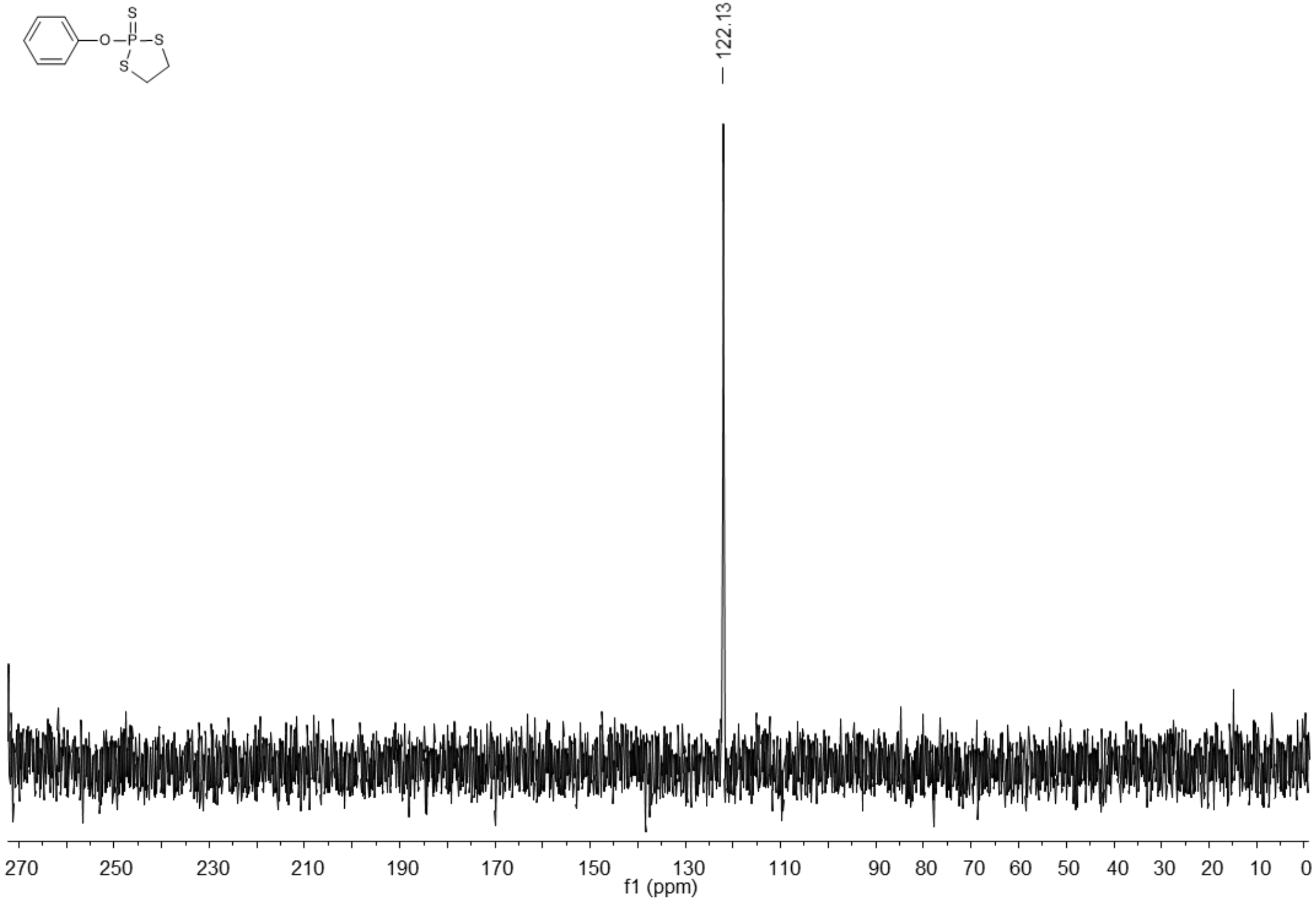

Figure A13. **^{31}P NMR (162 MHz, $CDCl_3$)** spectrum of **2b**.

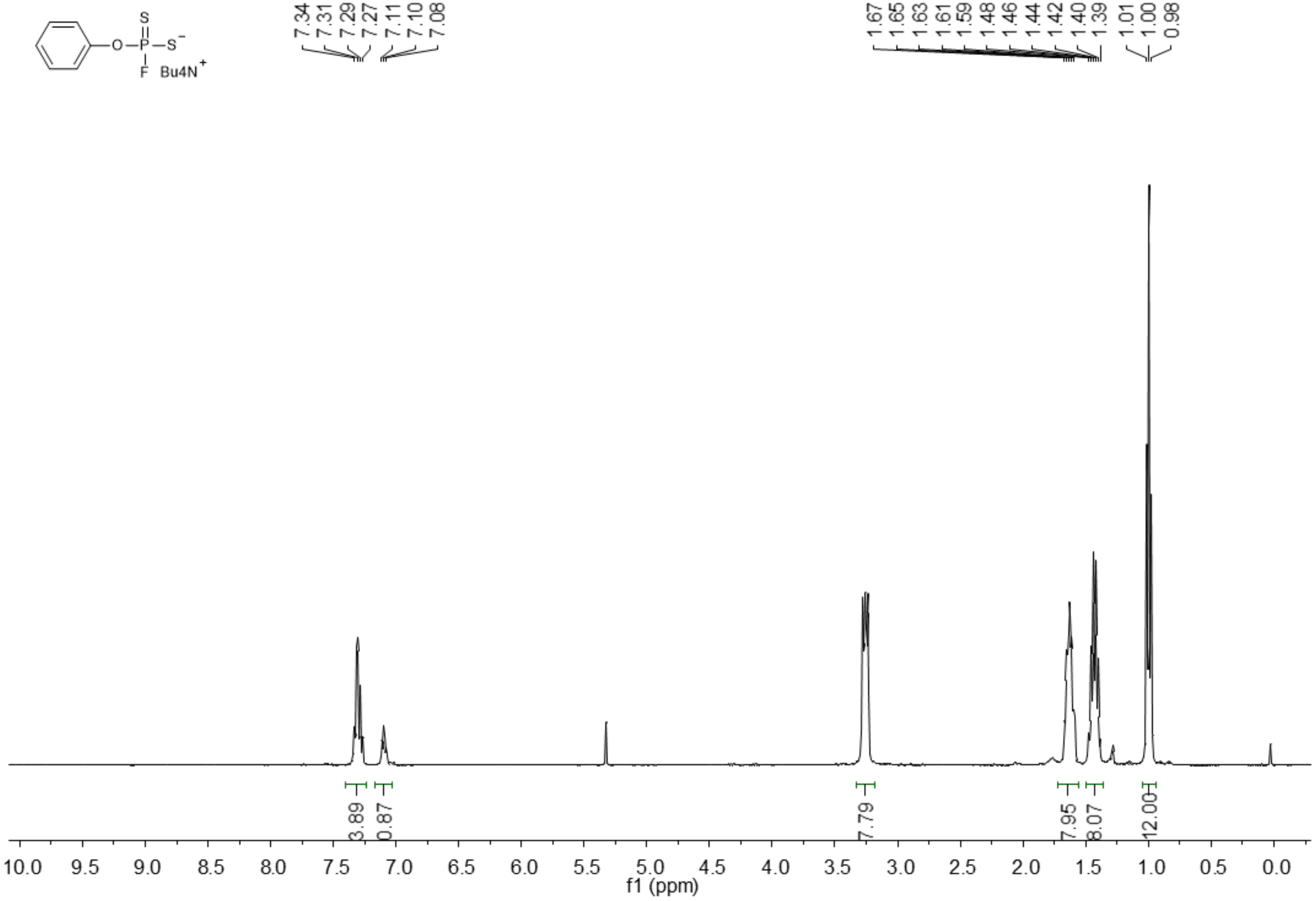

Figure A14. ^{1}**H NMR (400 MHz, CD_3OD)** spectrum of **2**.

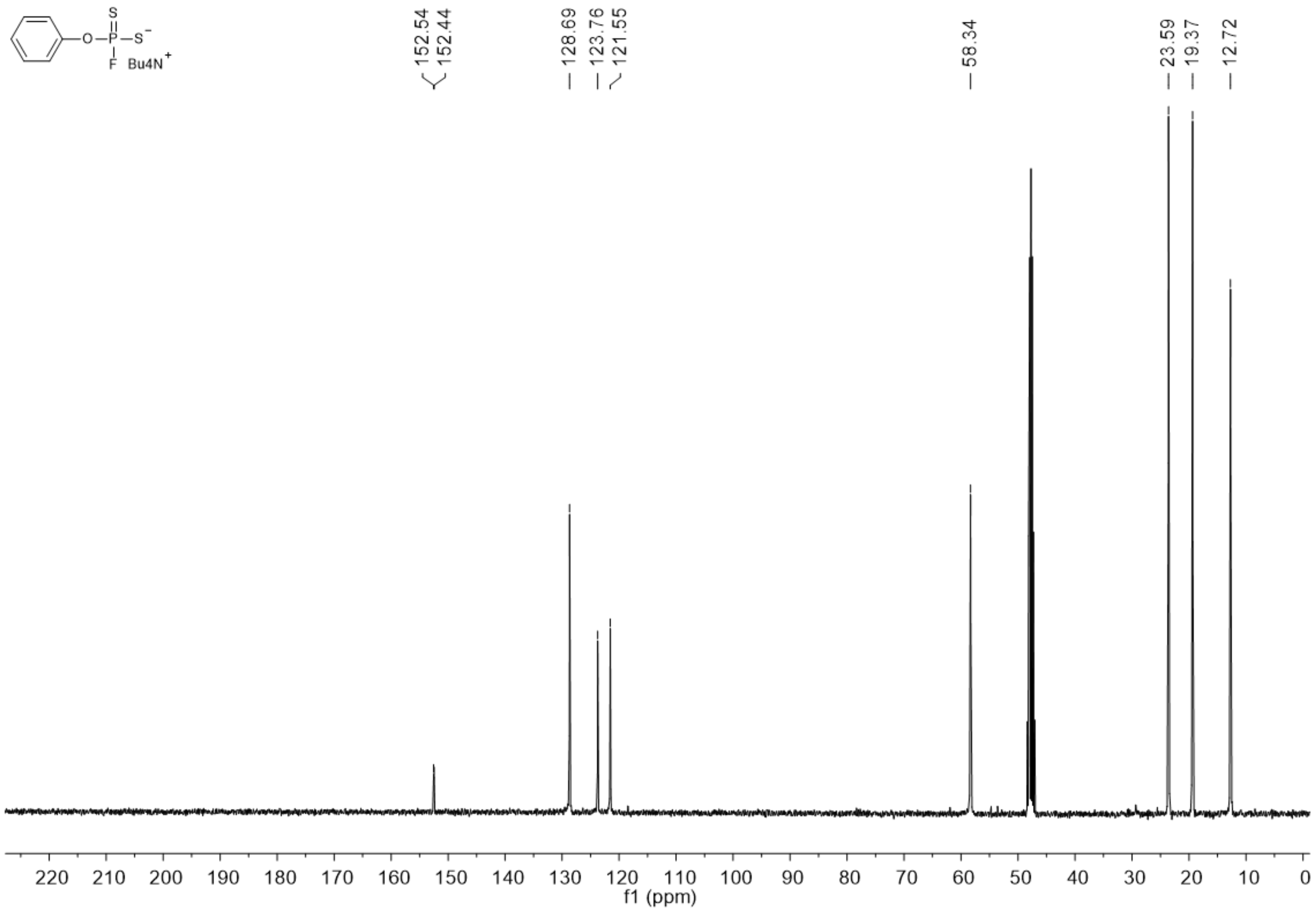

Figure A15. **^{11}C NMR (101 MHz, CD_3OD)** spectrum of **2**.

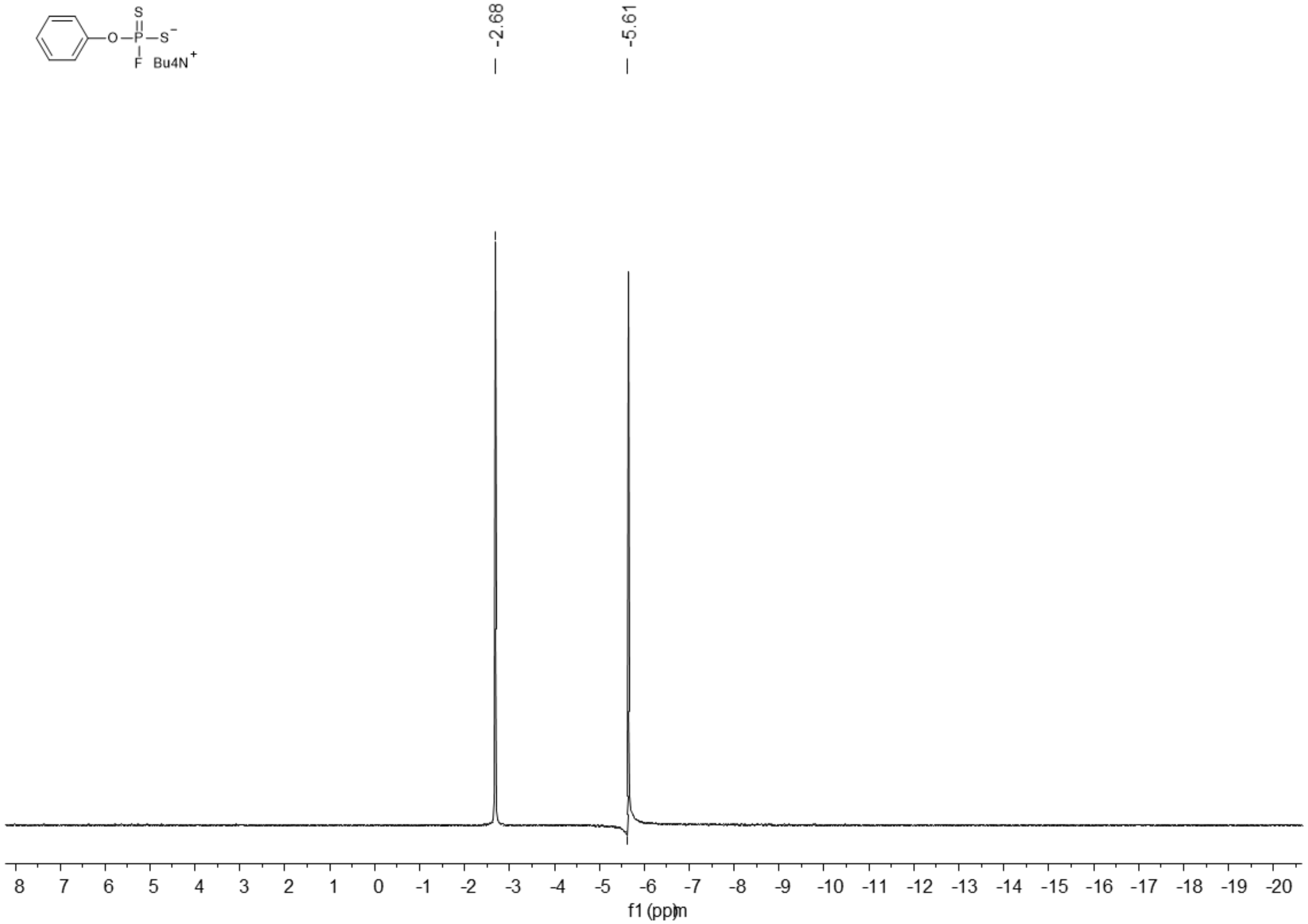

Figure A16. **^{19}F NMR (376 MHz, CD_3OD)** spectrum of **2**.

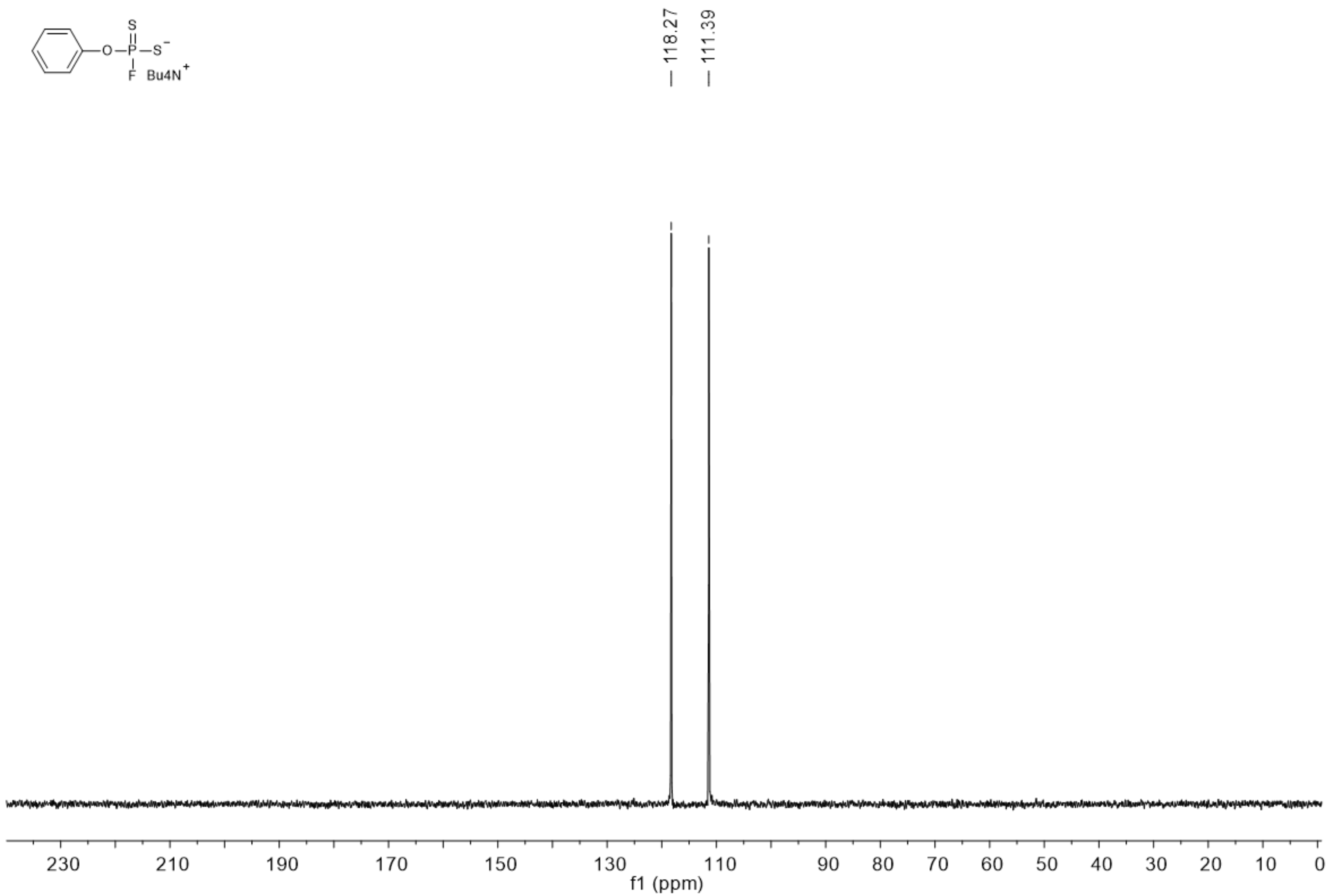

Figure A17. ^{31}**P NMR (162 MHz, CD$_3$OD)** spectrum of **2**.

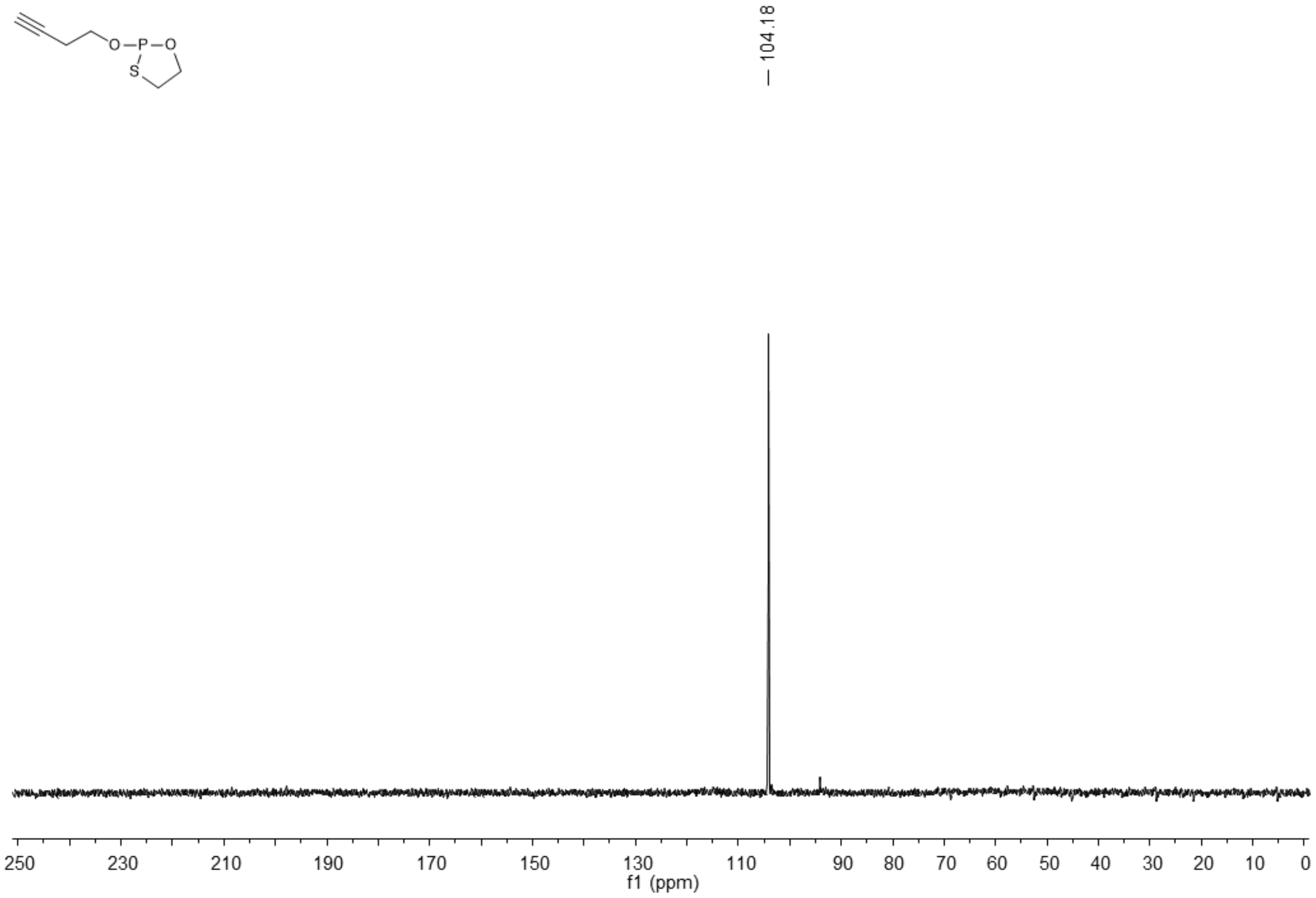

Figure A18. **^{31}P NMR (162 MHz, $CDCl_3$)** spectrum of **3a**.

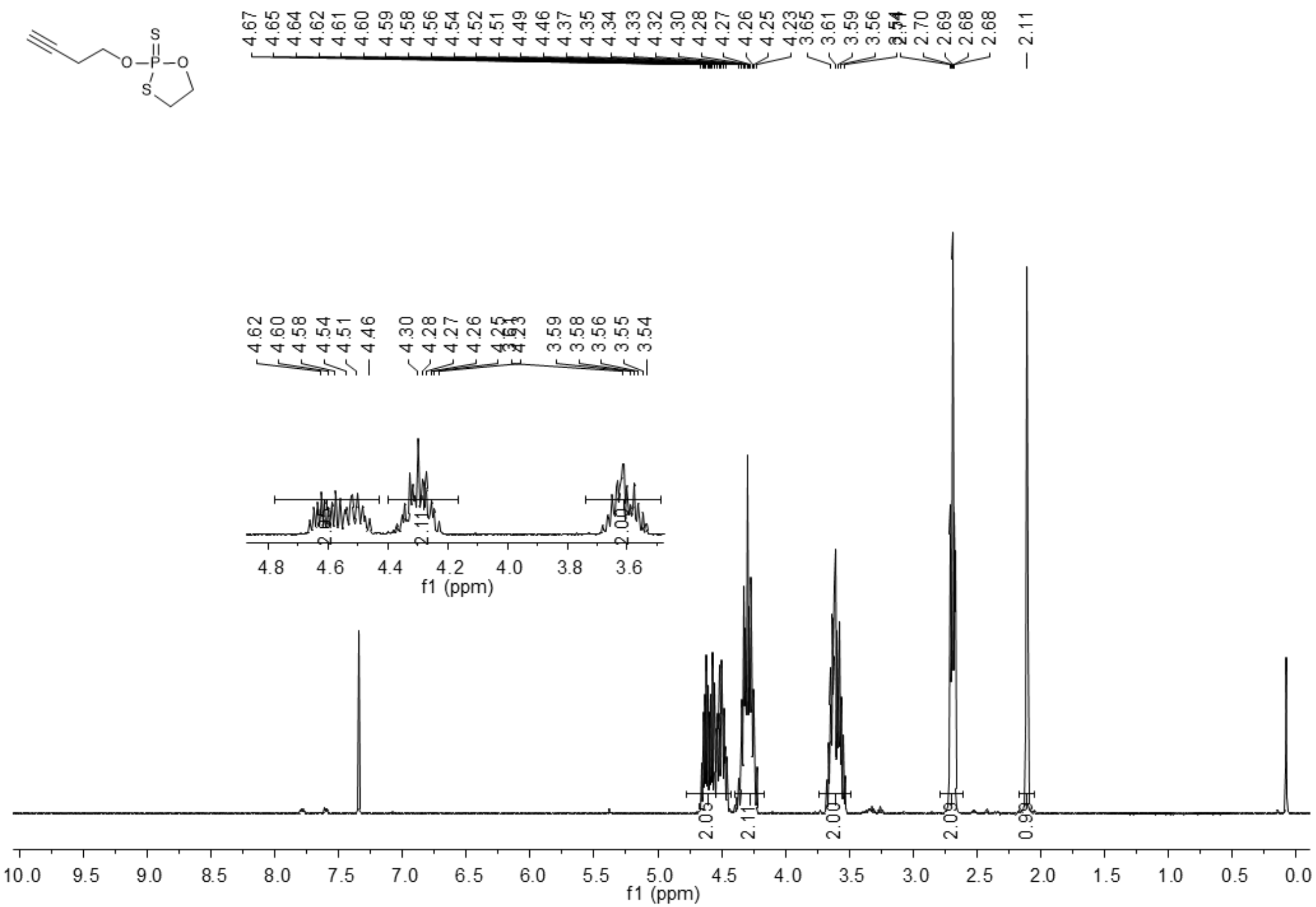

Figure A19. **^{1}H NMR (400 MHz, CDCl$_3$)** spectrum of **3b**.

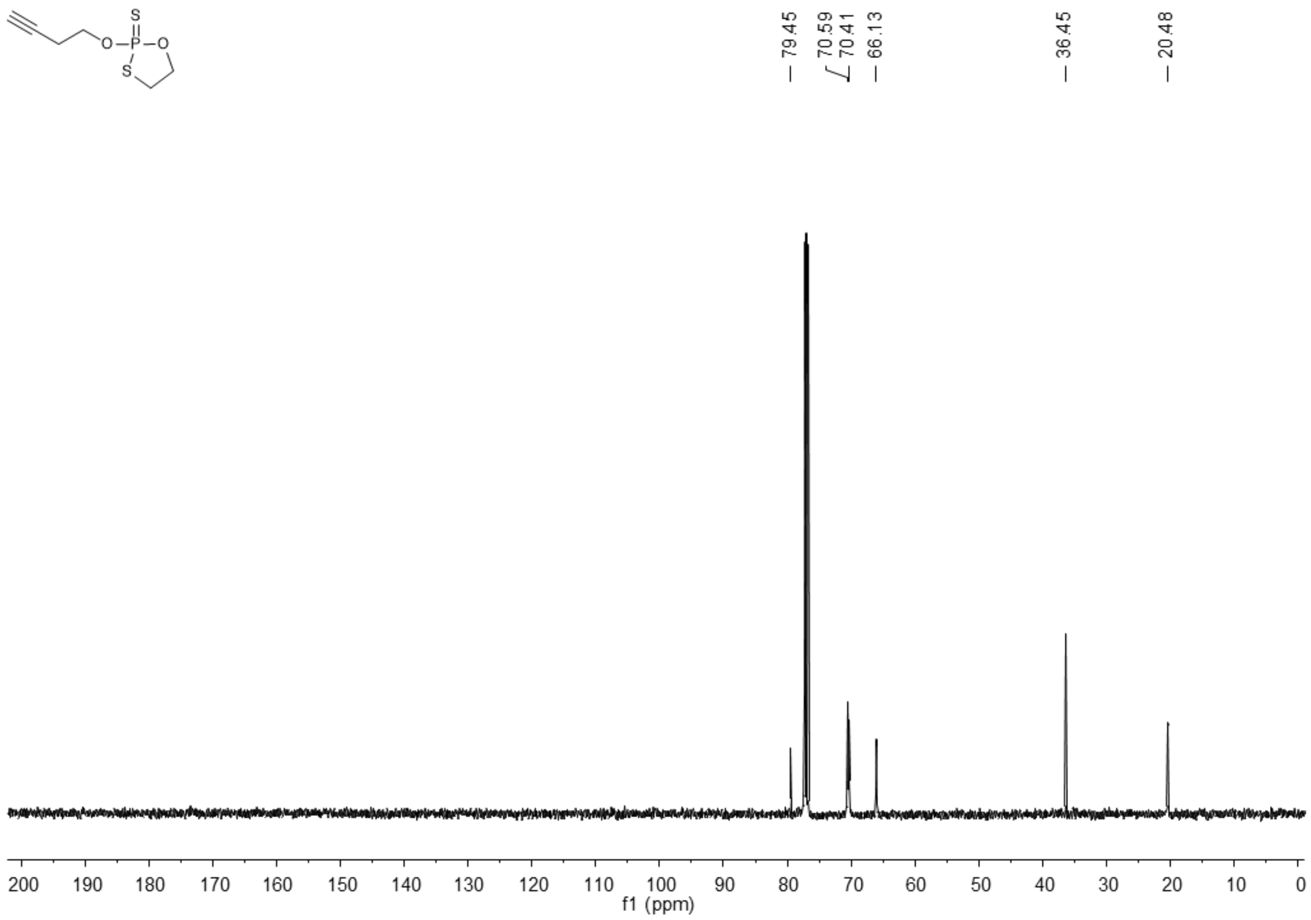

Figure A20. **^{13}C NMR (101 MHz, $CDCl_3$)** spectrum of **3b**.

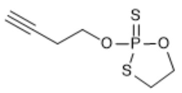

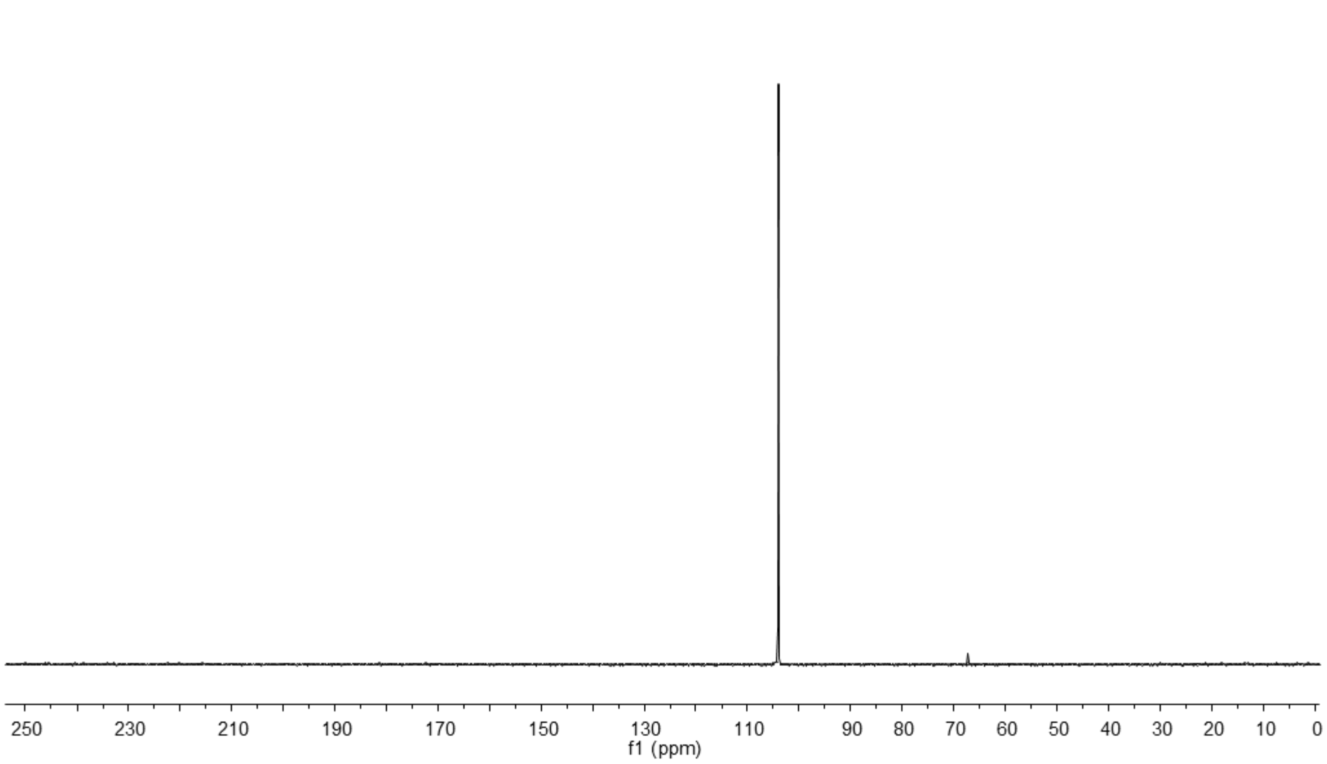

Figure A21. **^{31}P NMR (162 MHz, $CDCl_3$)** spectrum of **3b**.

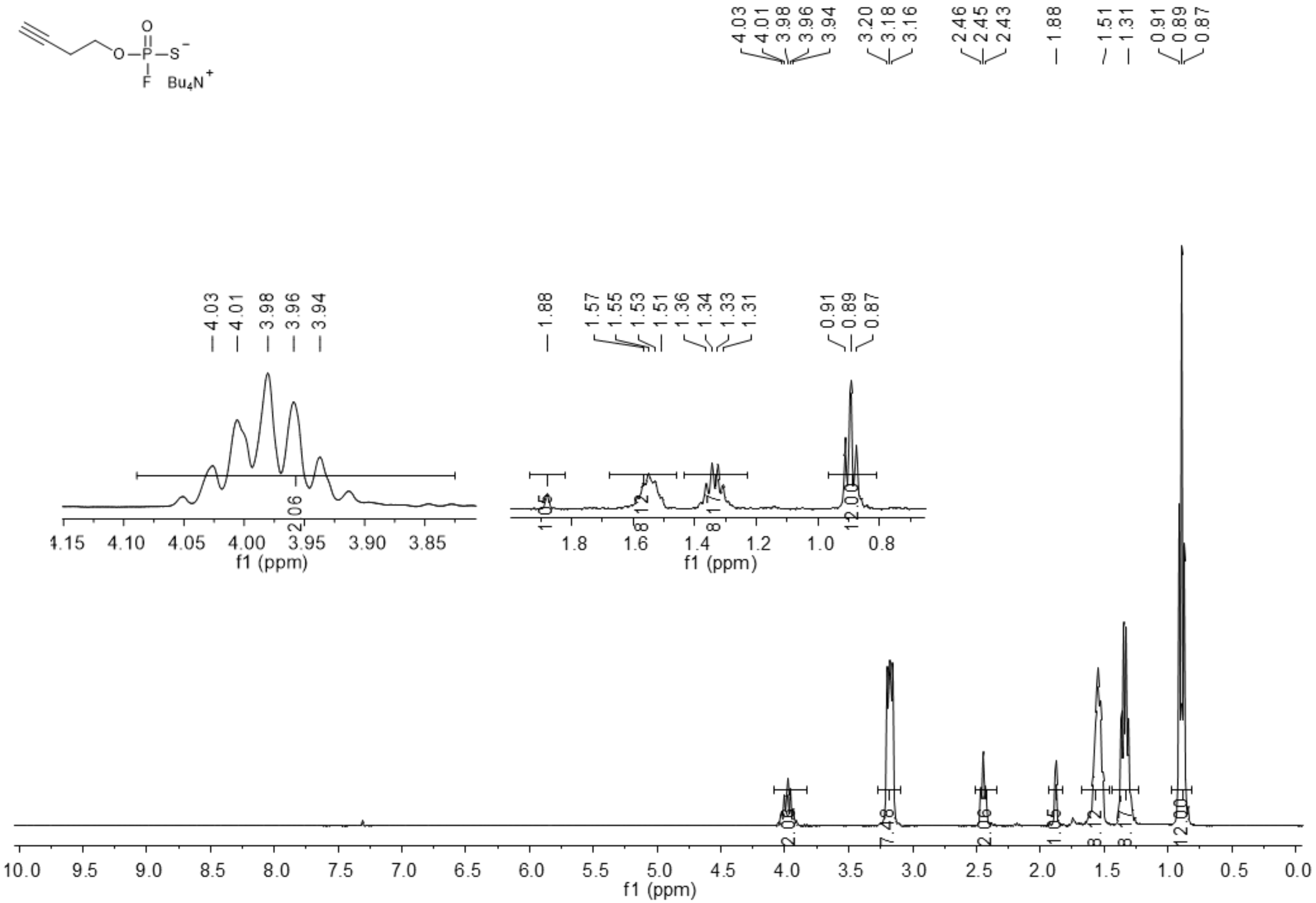

Figure A22. **^{1}H NMR (376 MHz, CDCl$_3$)** spectrum of **3**.

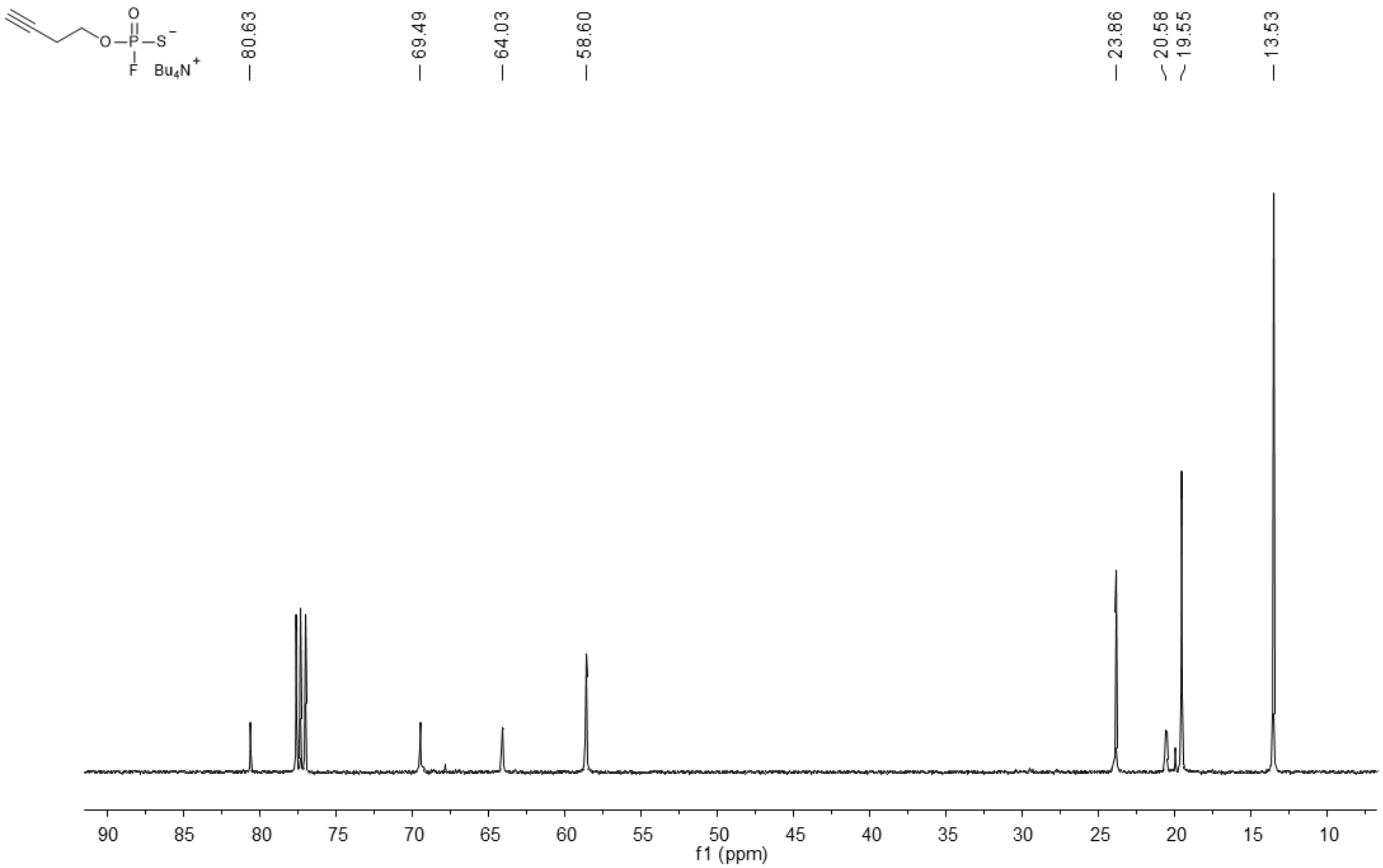

Figure A23. **^{13}C NMR (101 MHz, $CDCl_3$)** spectrum of **3**.

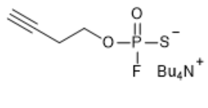

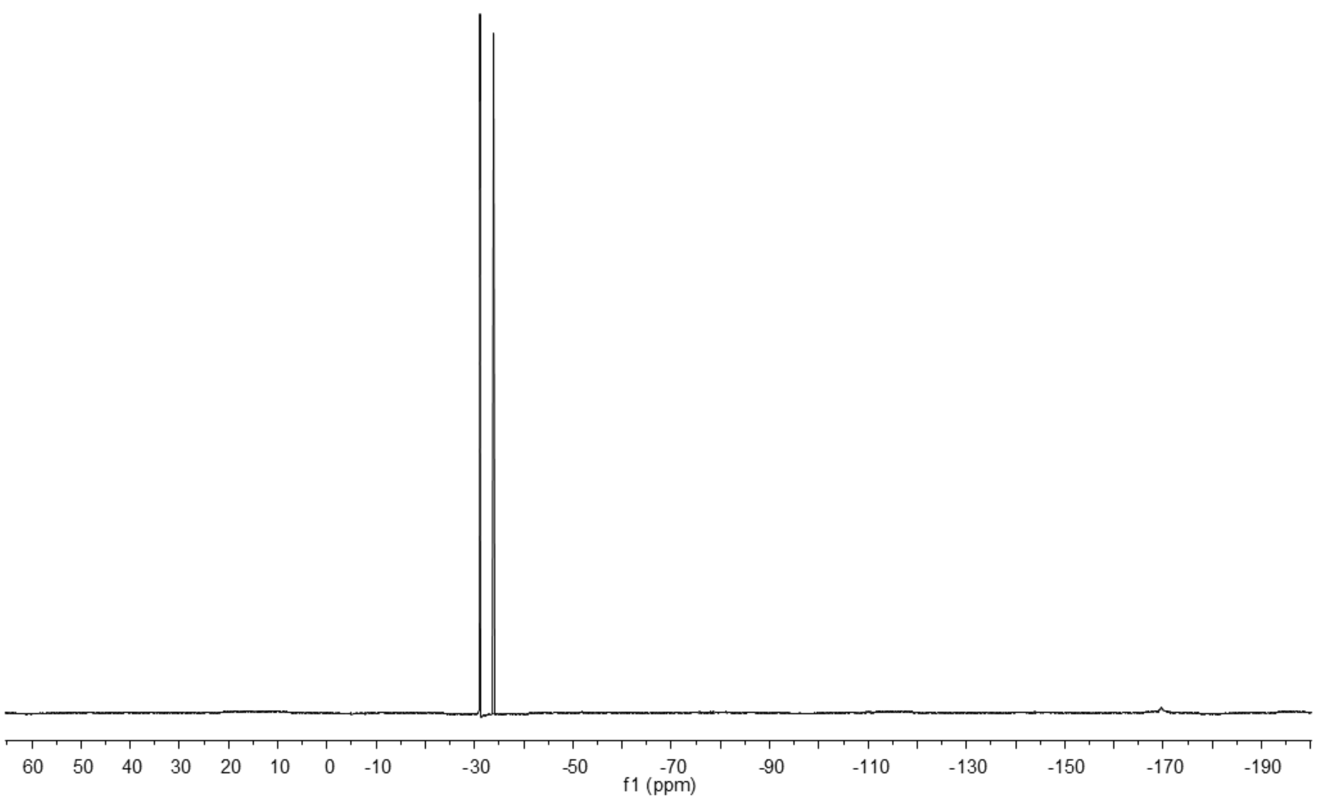

Figure A24. **^{19}F NMR (376 MHz, $CDCl_3$)** spectrum of **3**.

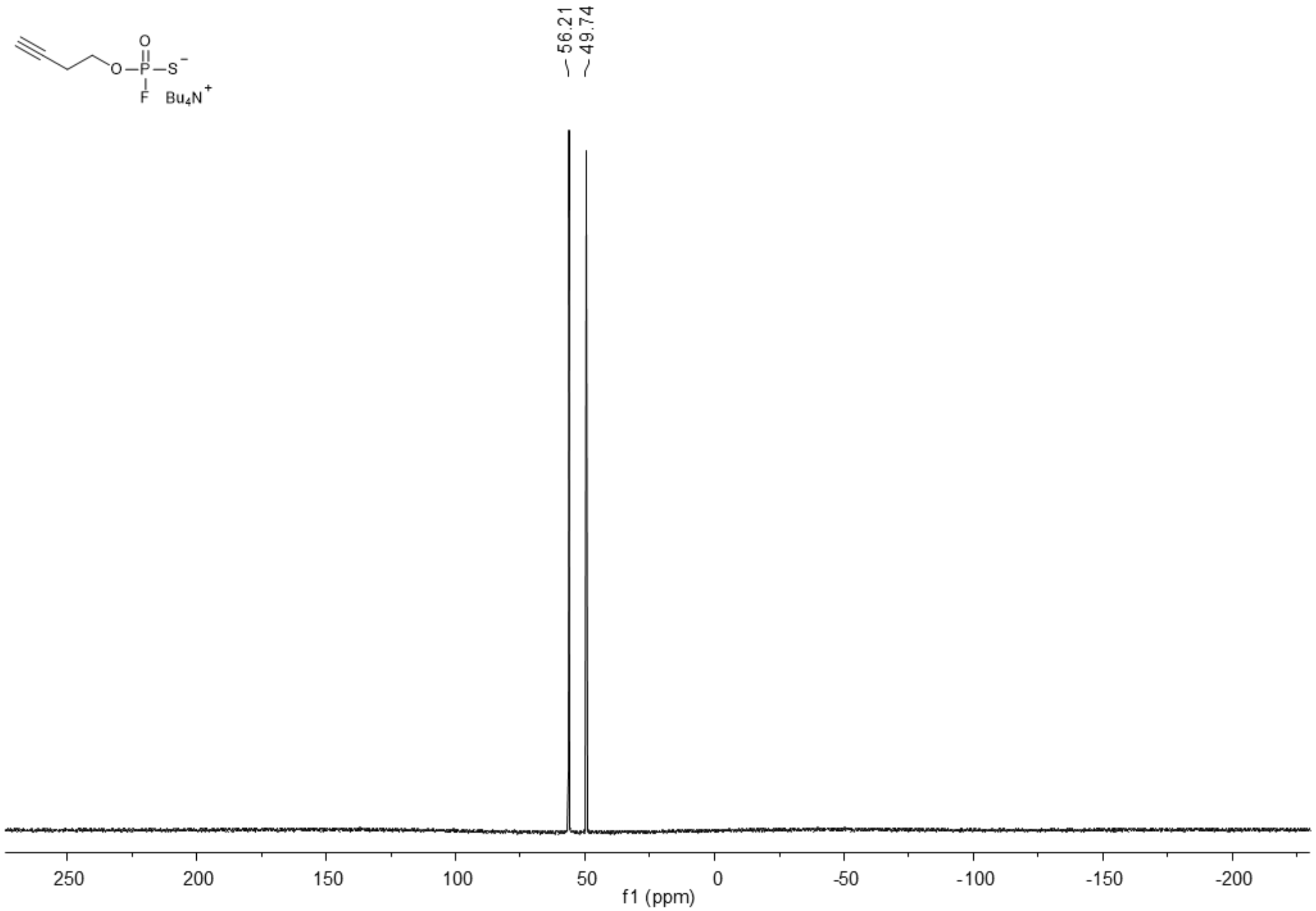

Figure A25. **^{31}P NMR (162 MHz, $CDCl_3$)** spectrum of **3**.

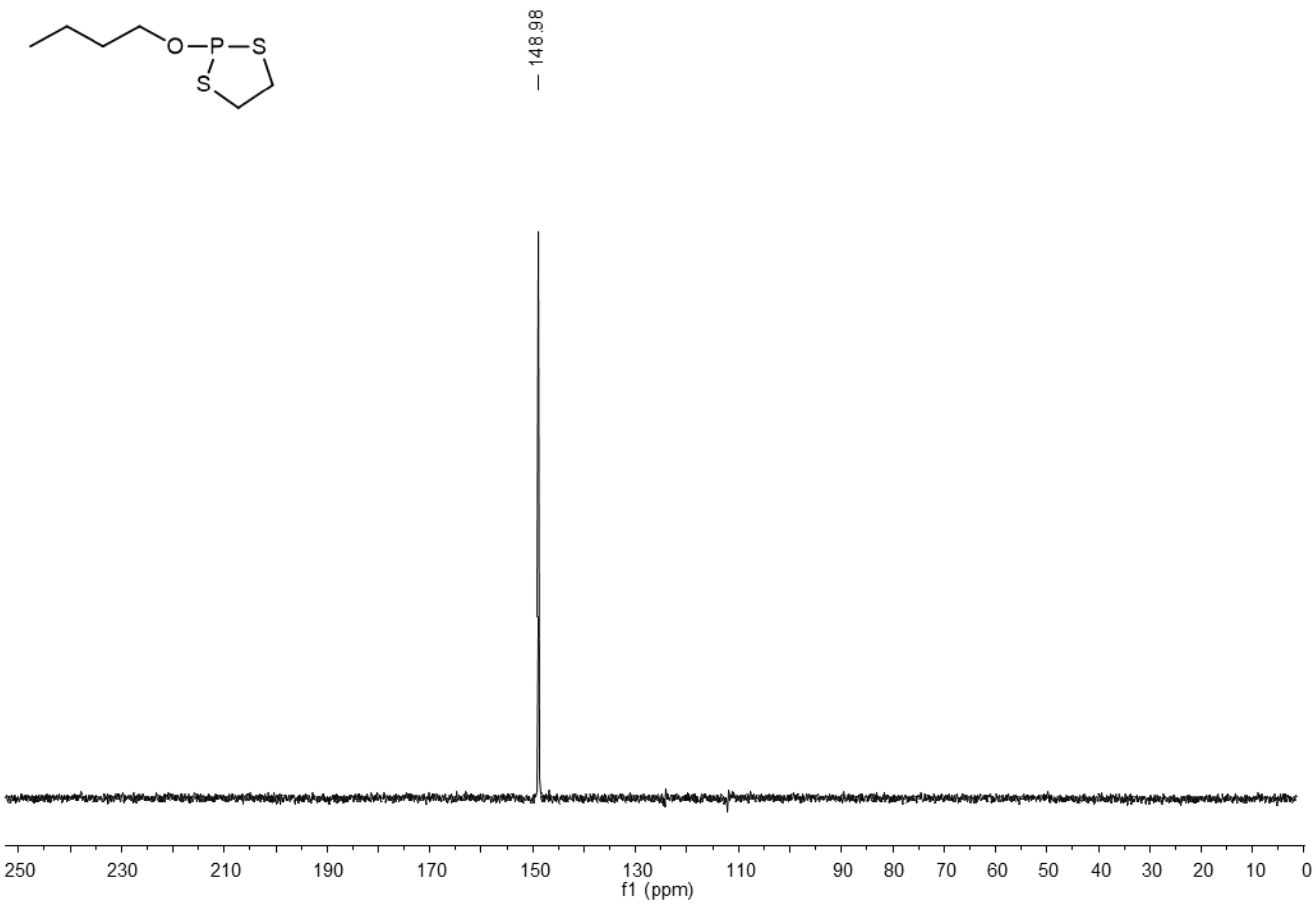

Figure A26. **^{31}P NMR (162 MHz, $CDCl_3$)** spectrum of **5a**.

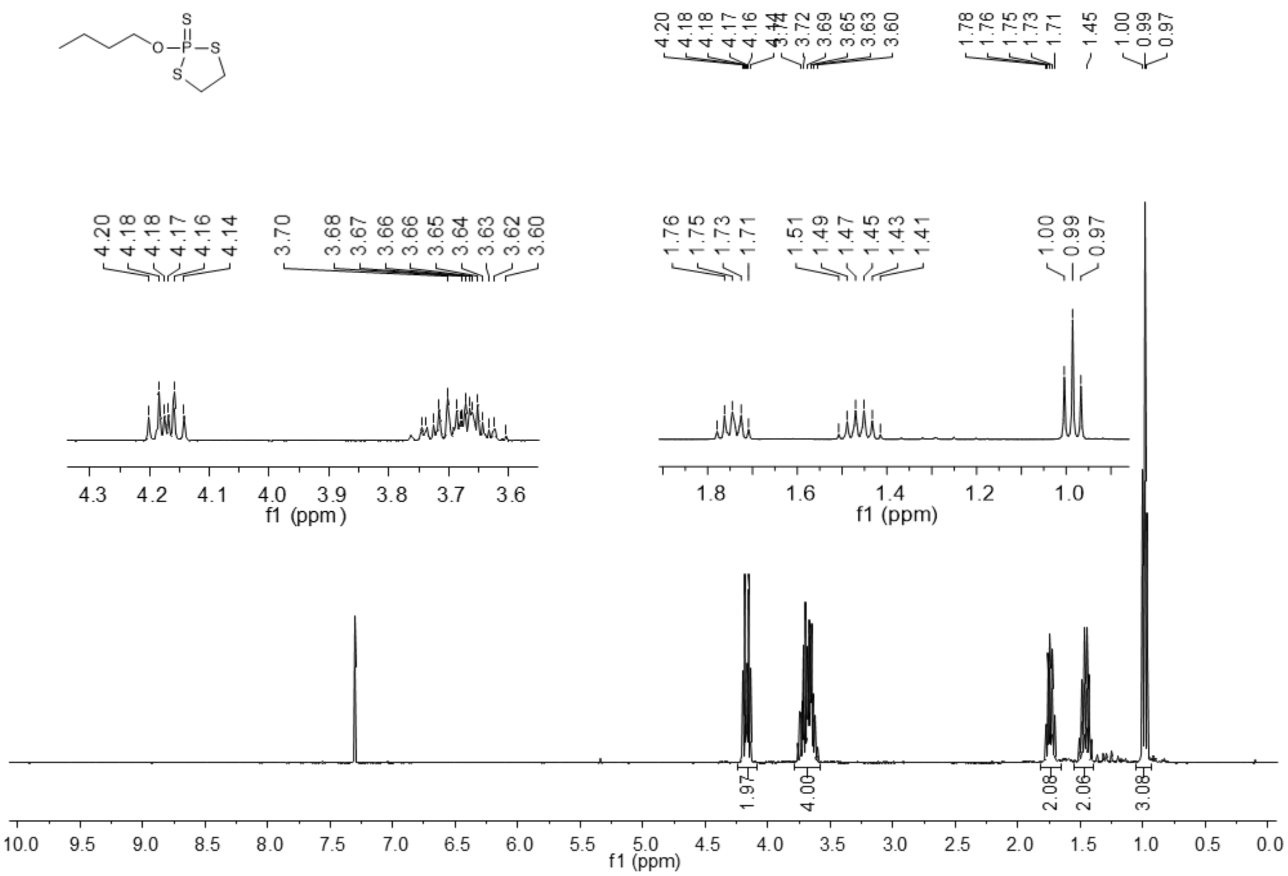

Figure A27. **^{1}H NMR (400 MHz, $CDCl_3$)** spectrum of **5b**.

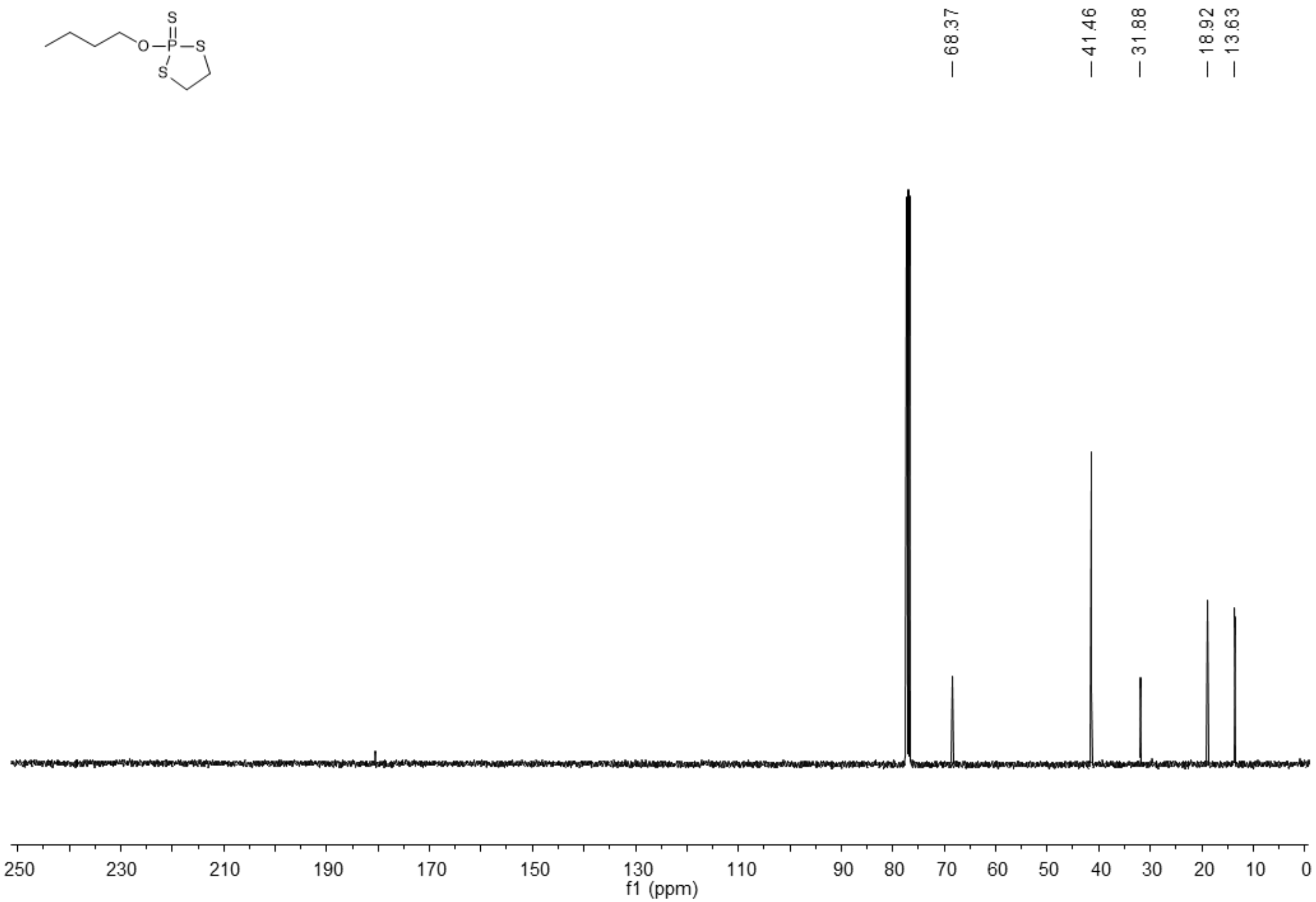

Figure A28. **^{13}C NMR (101 MHz, $CDCl_3$)** spectrum of **5b**.

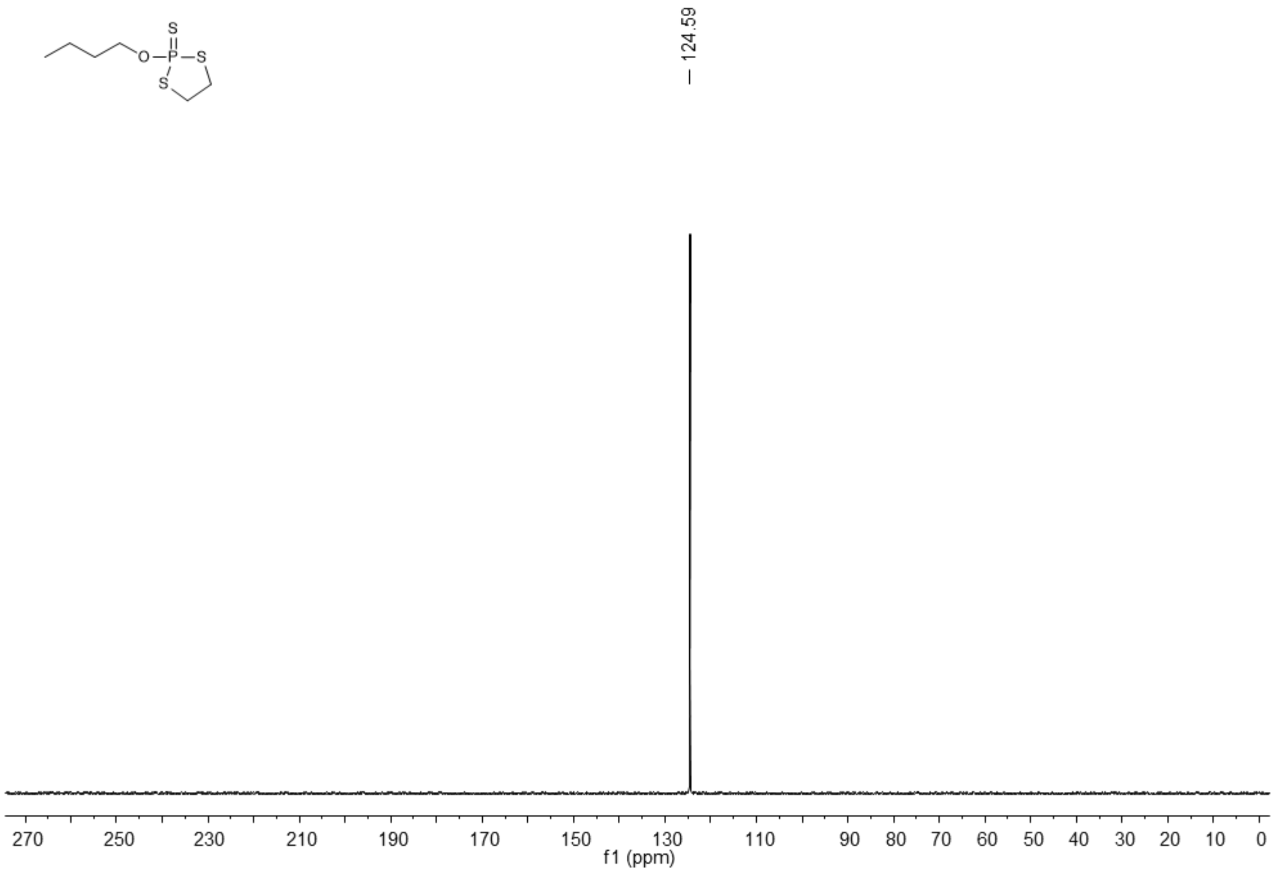

Figure A29. **^{31}P NMR (162 MHz, $CDCl_3$)** spectrum of **5b**.

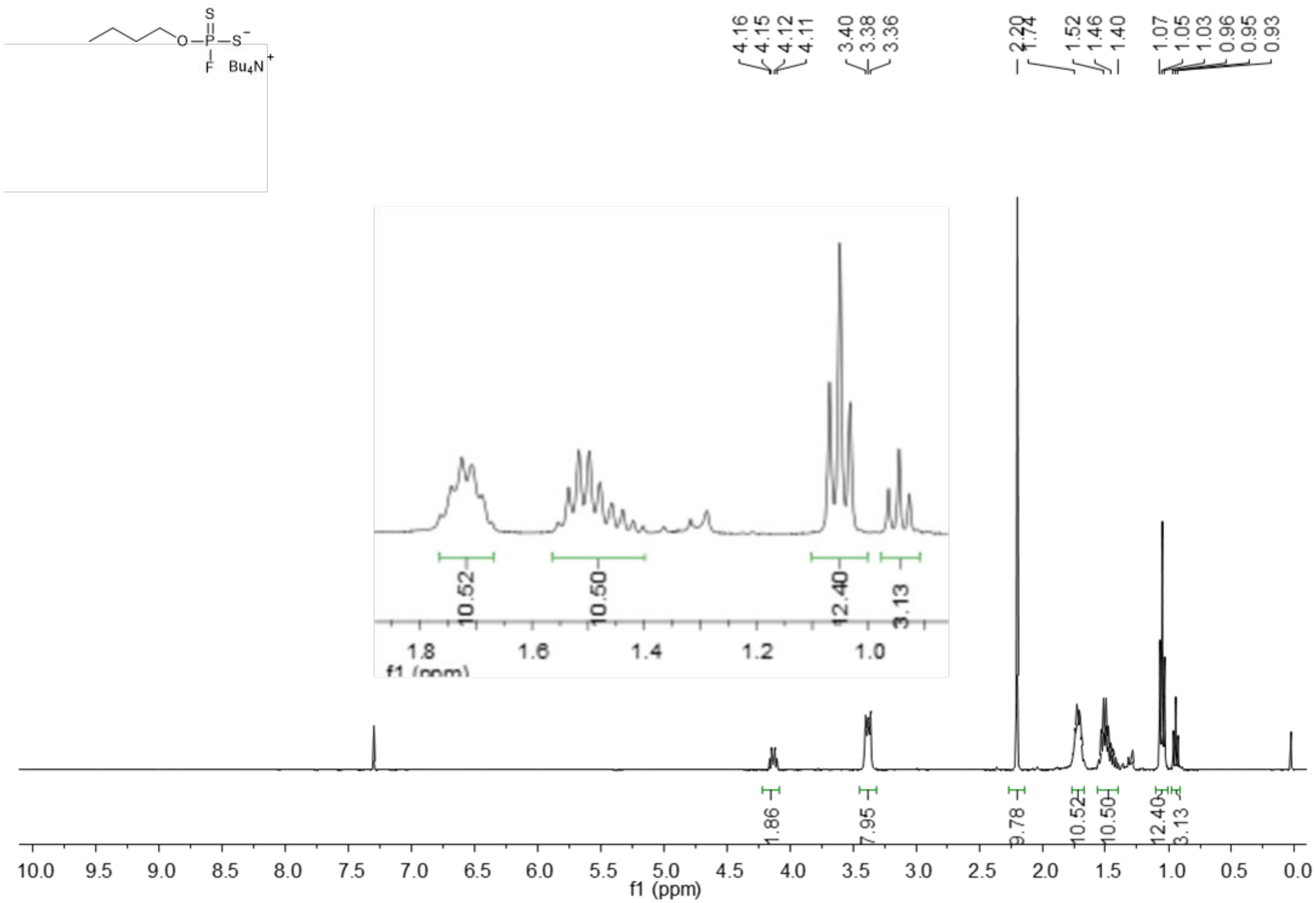

Figure A30. **^{1}H NMR (400 MHz, CDCl$_3$)** spectrum of **5**.

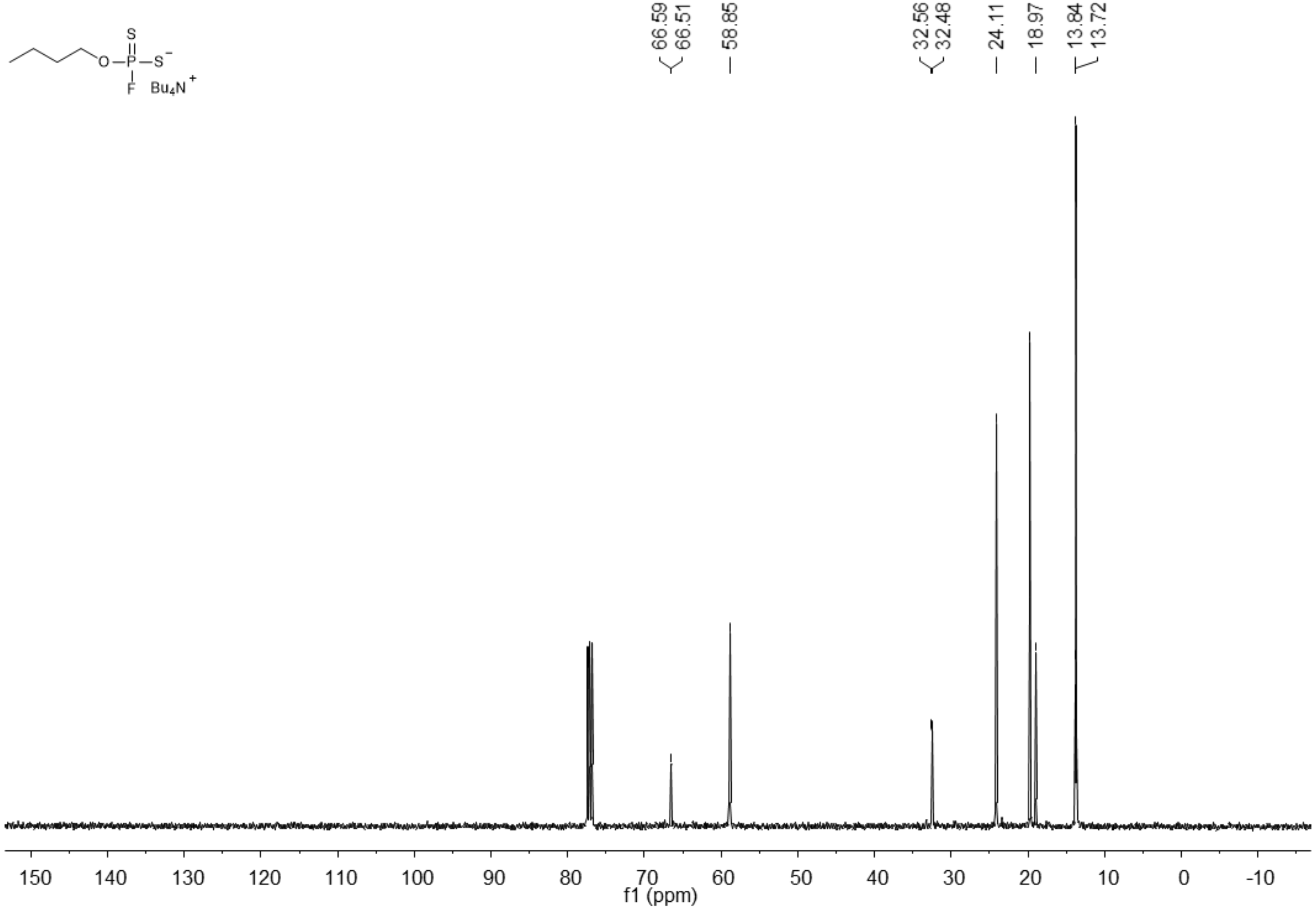

Figure A31. **^{13}C NMR (101 MHz, $CDCl_3$)** spectrum of **5**.

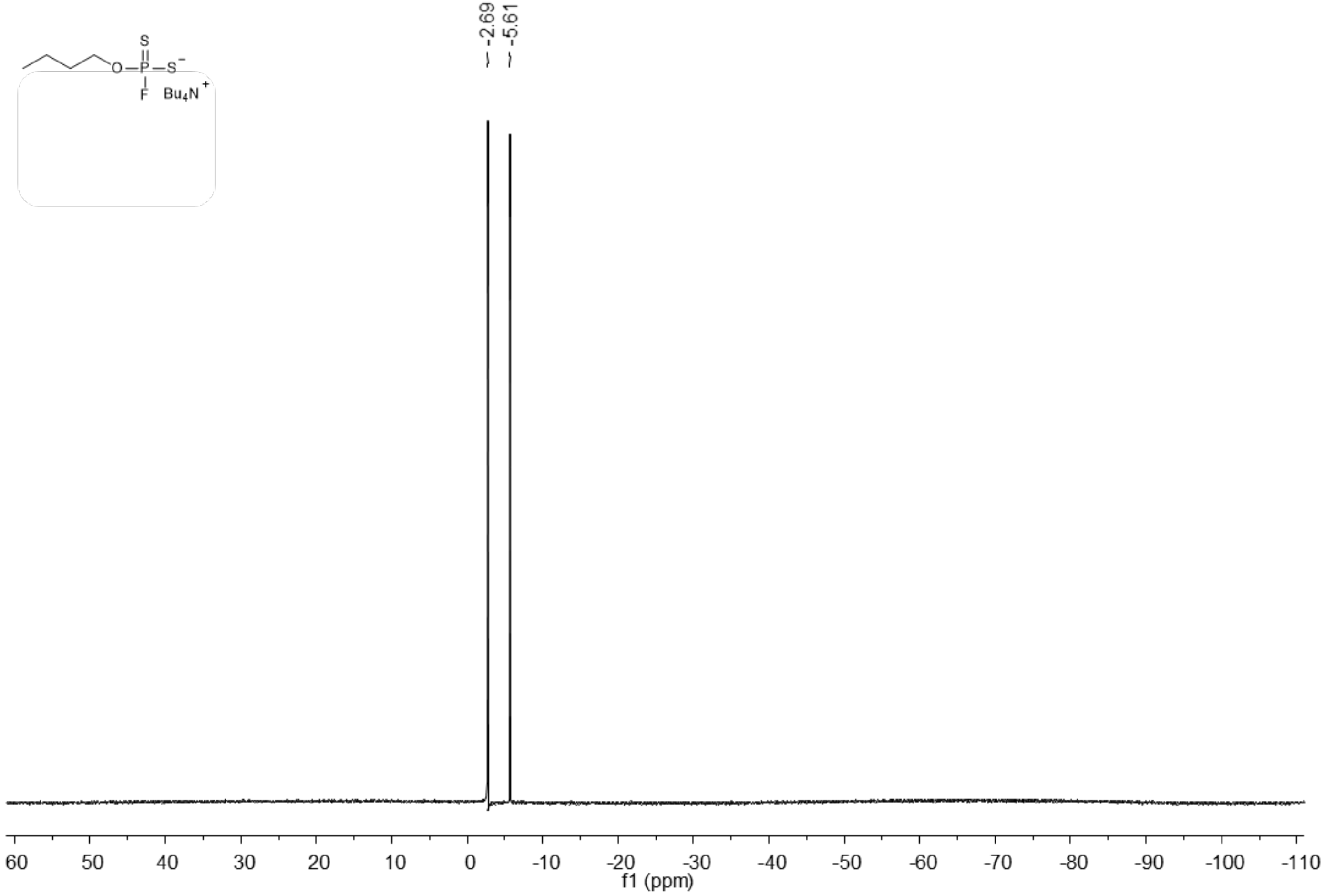

Figure A32. **^{19}F NMR (376 MHz, $CDCl_3$)** spectrum of **5**.

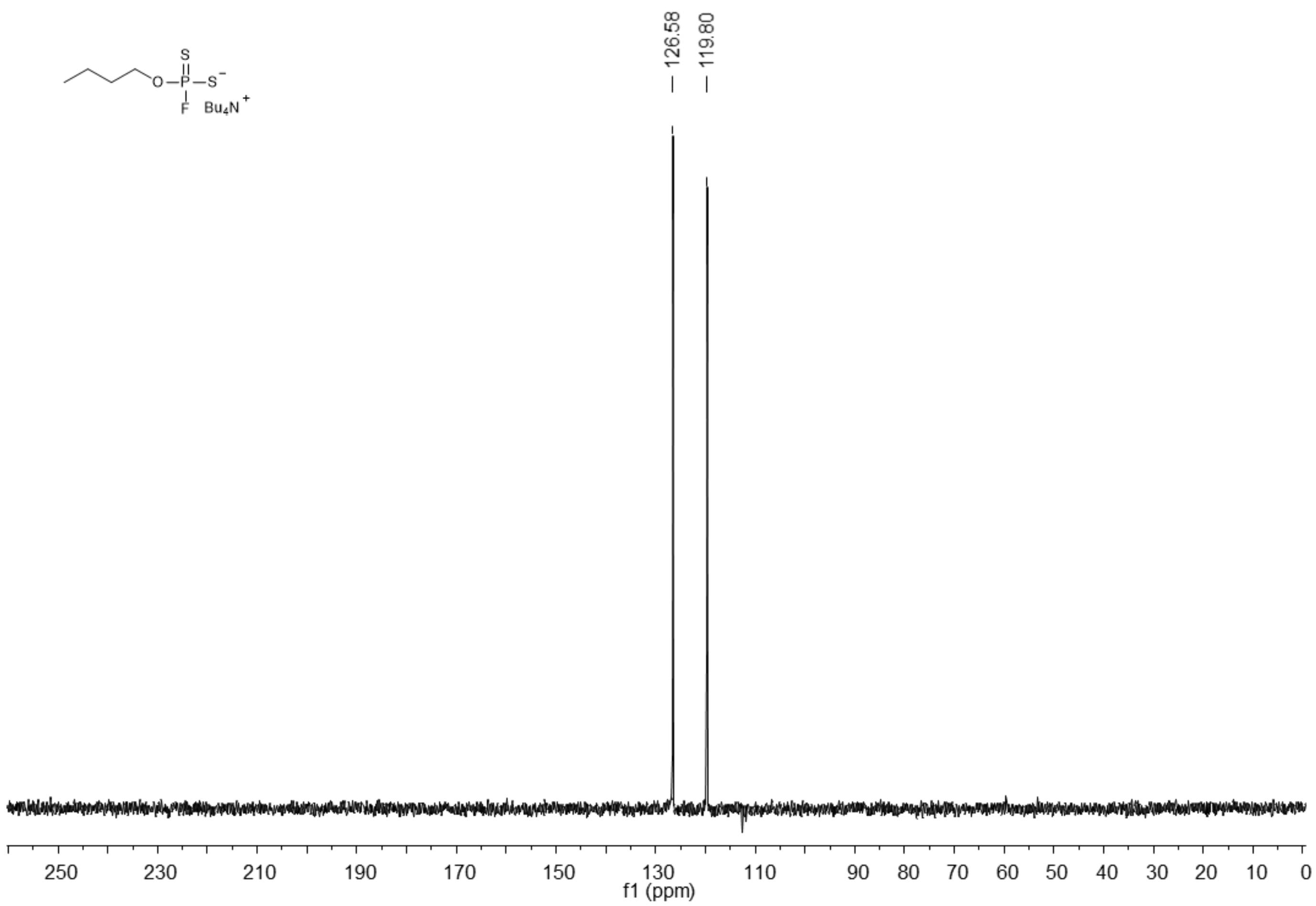

Figure A33. **^{31}P NMR (162 MHz, $CDCl_3$)** spectrum of **5**.

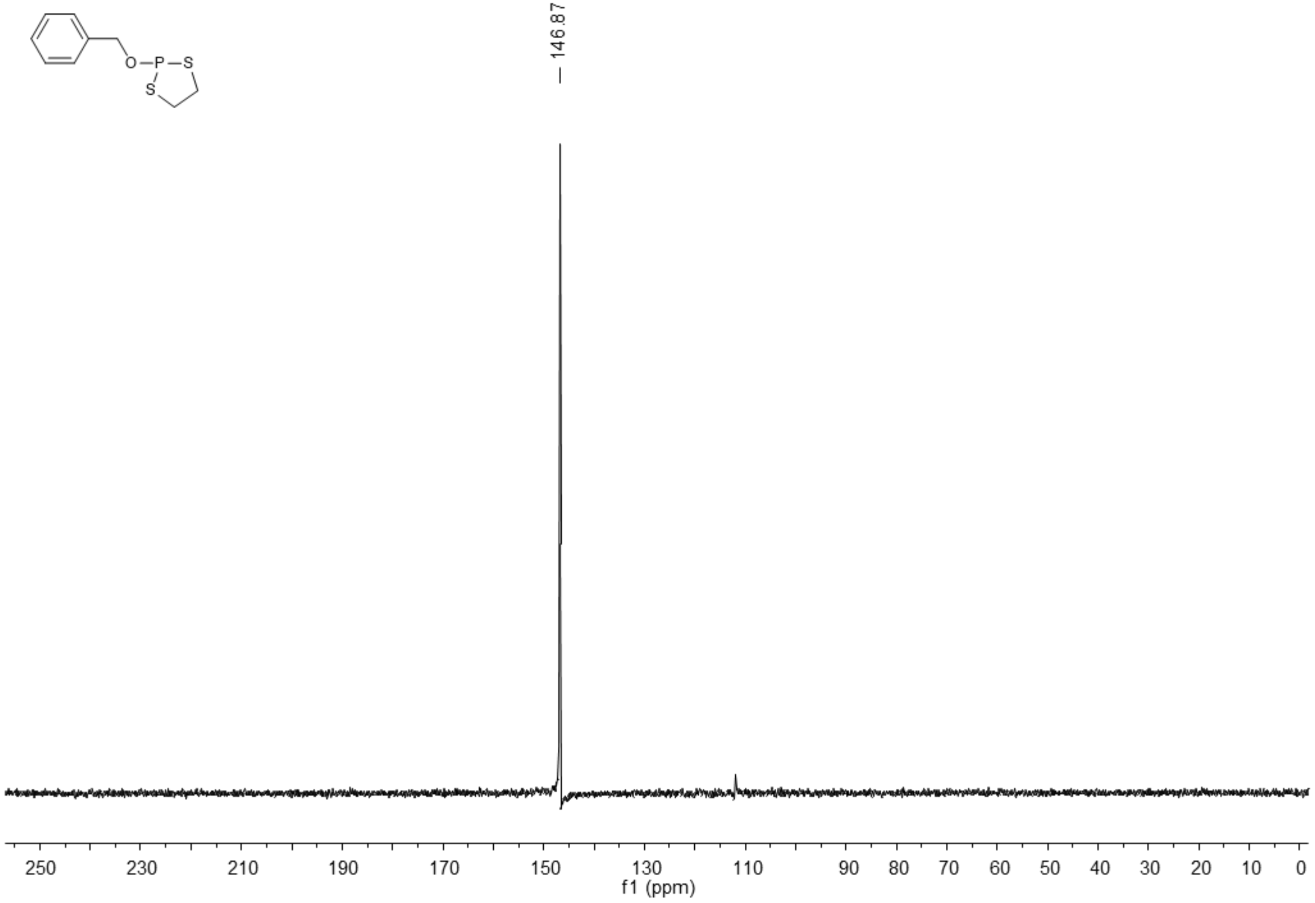

Figure A34. **^{31}P NMR (162 MHz, $CDCl_3$)** spectrum of **6a**.

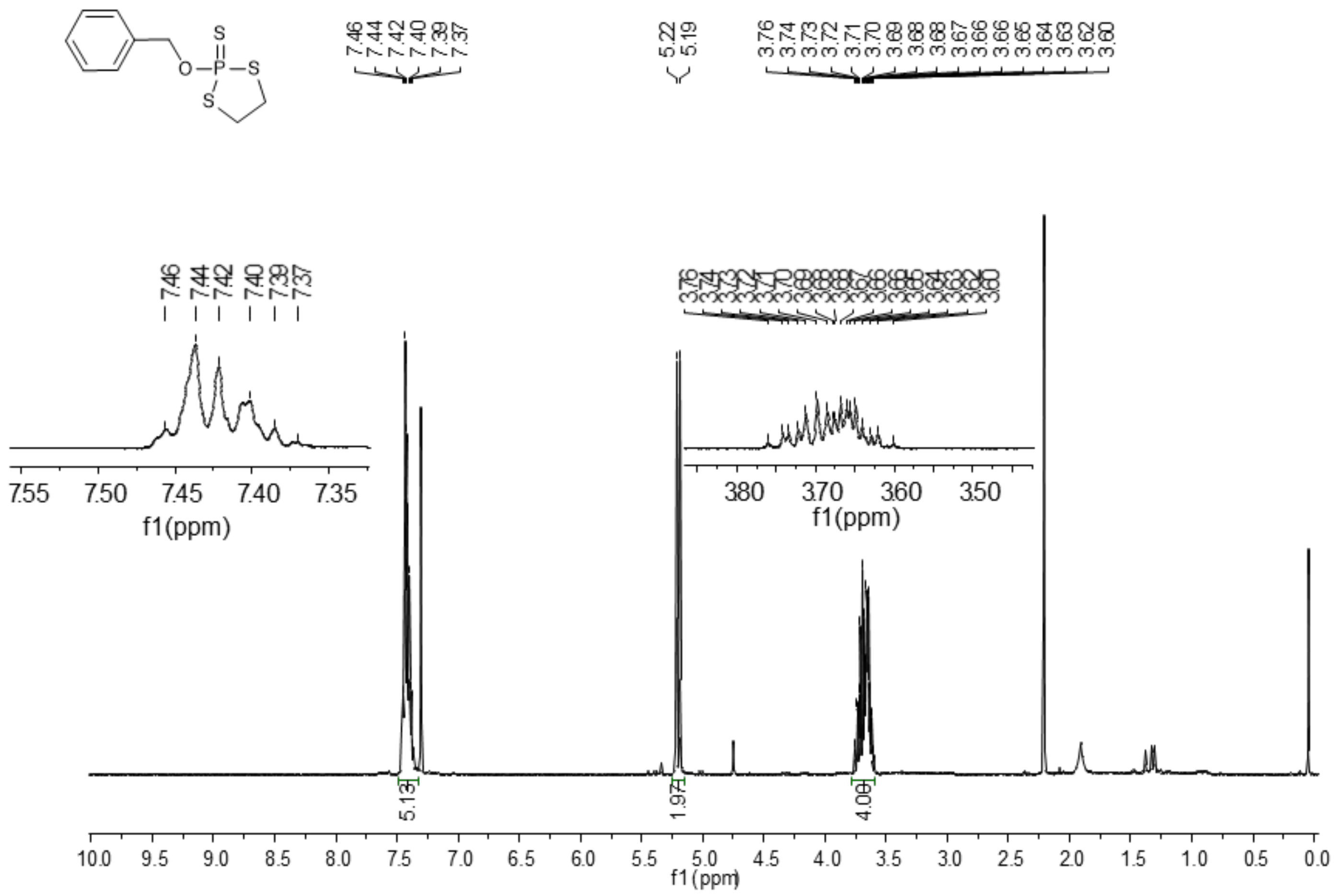

Figure A35. **^{1}H NMR (400 MHz, $CDCl_3$)** spectrum of **6b**.

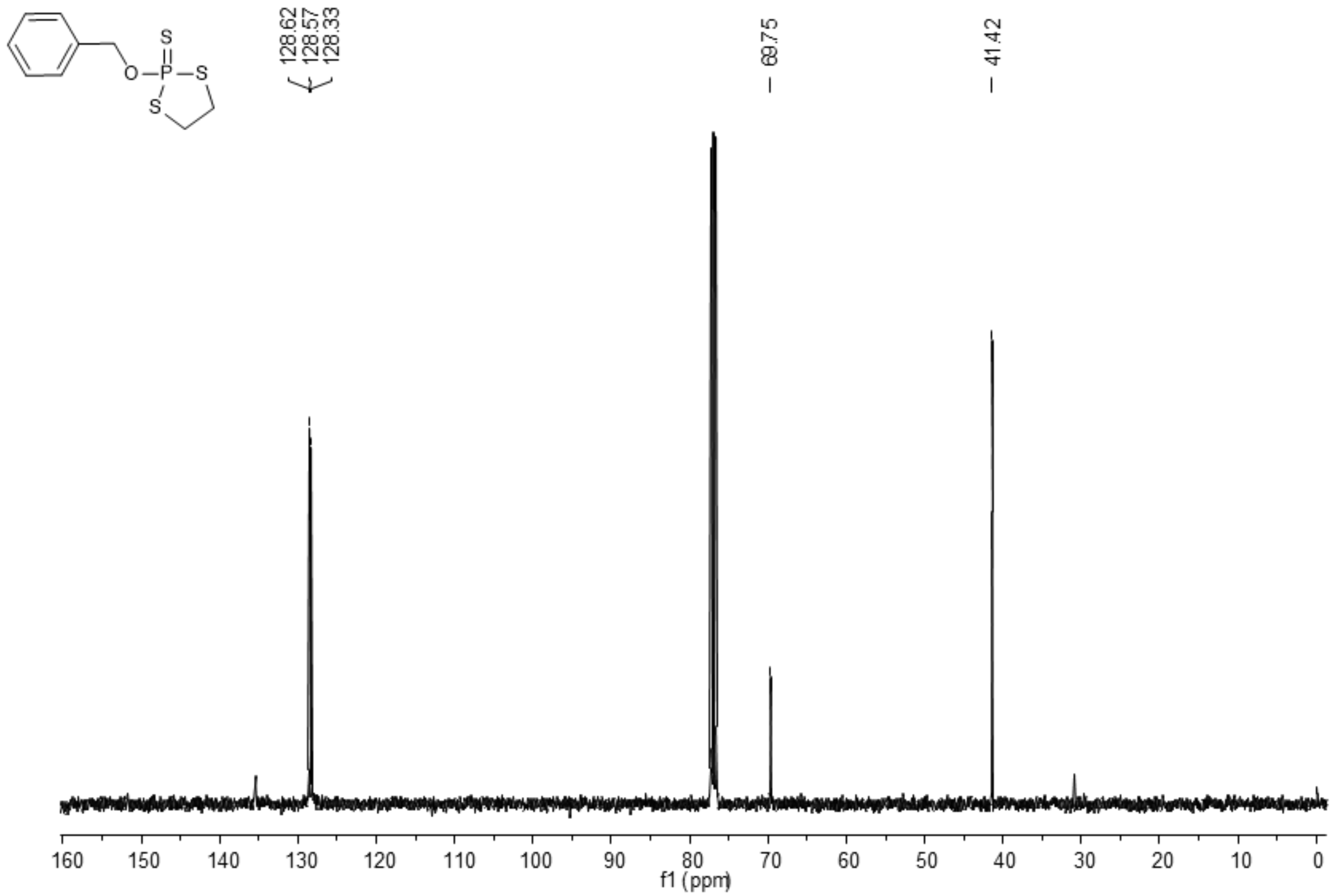

Figure A36. ^{13}**C NMR (101 MHz, CDCl$_3$)** spectrum of **6b**.

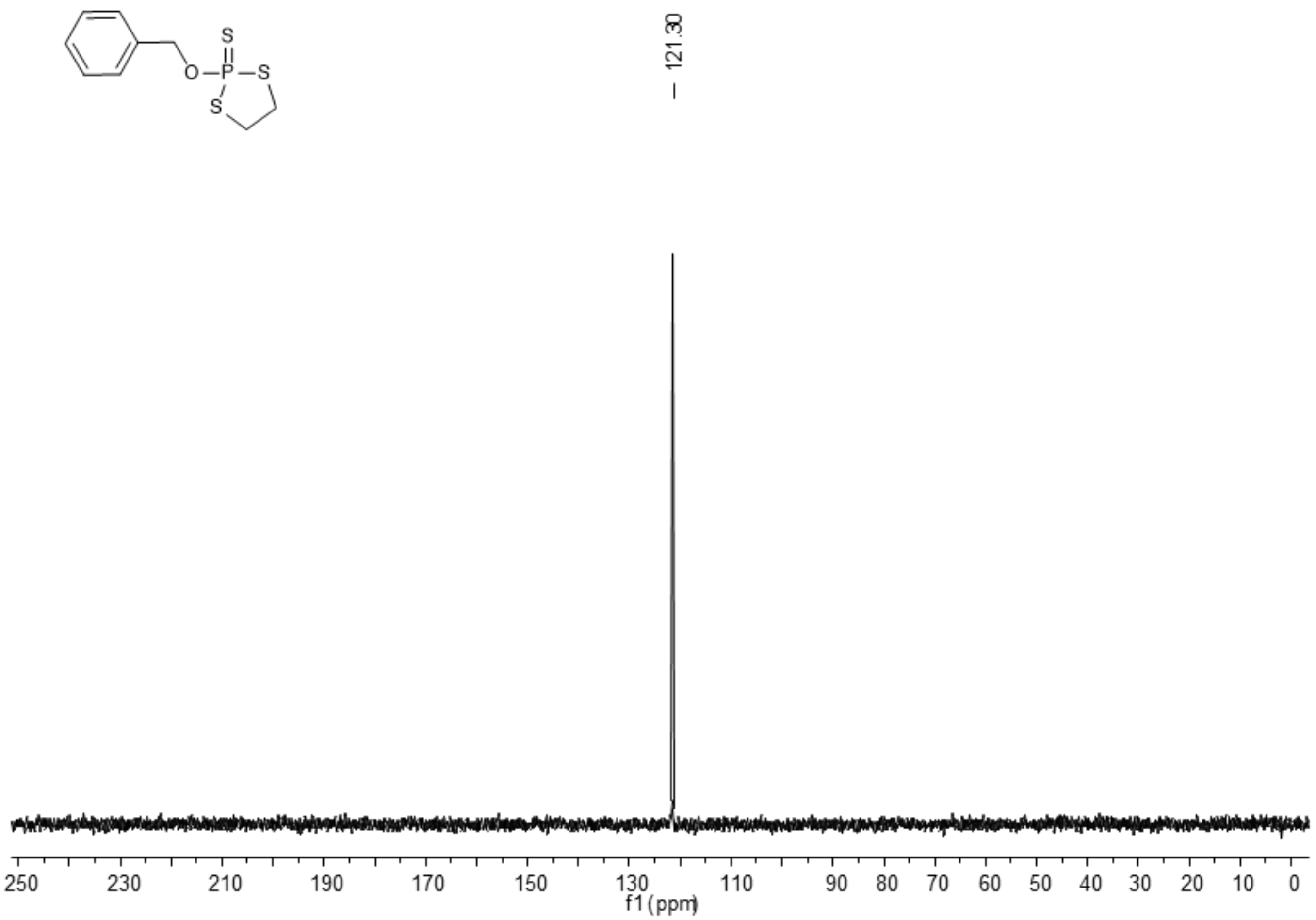

Figure A37. **^{31}P NMR (162 MHz, $CDCl_3$)** spectrum of **6b**.

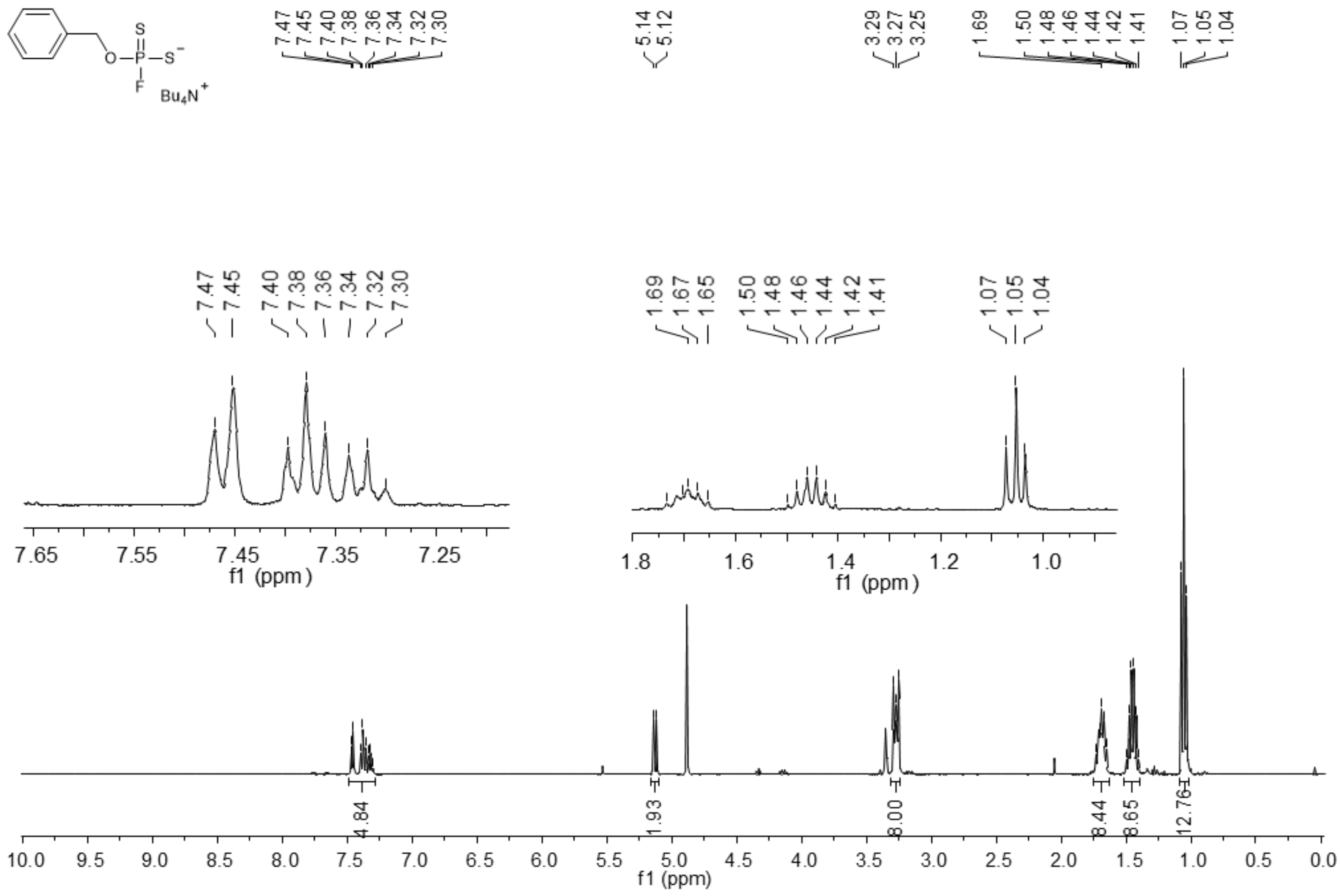

Figure A38. **^{1}H NMR (400 MHz, CD_3OD)** spectrum of **6**.

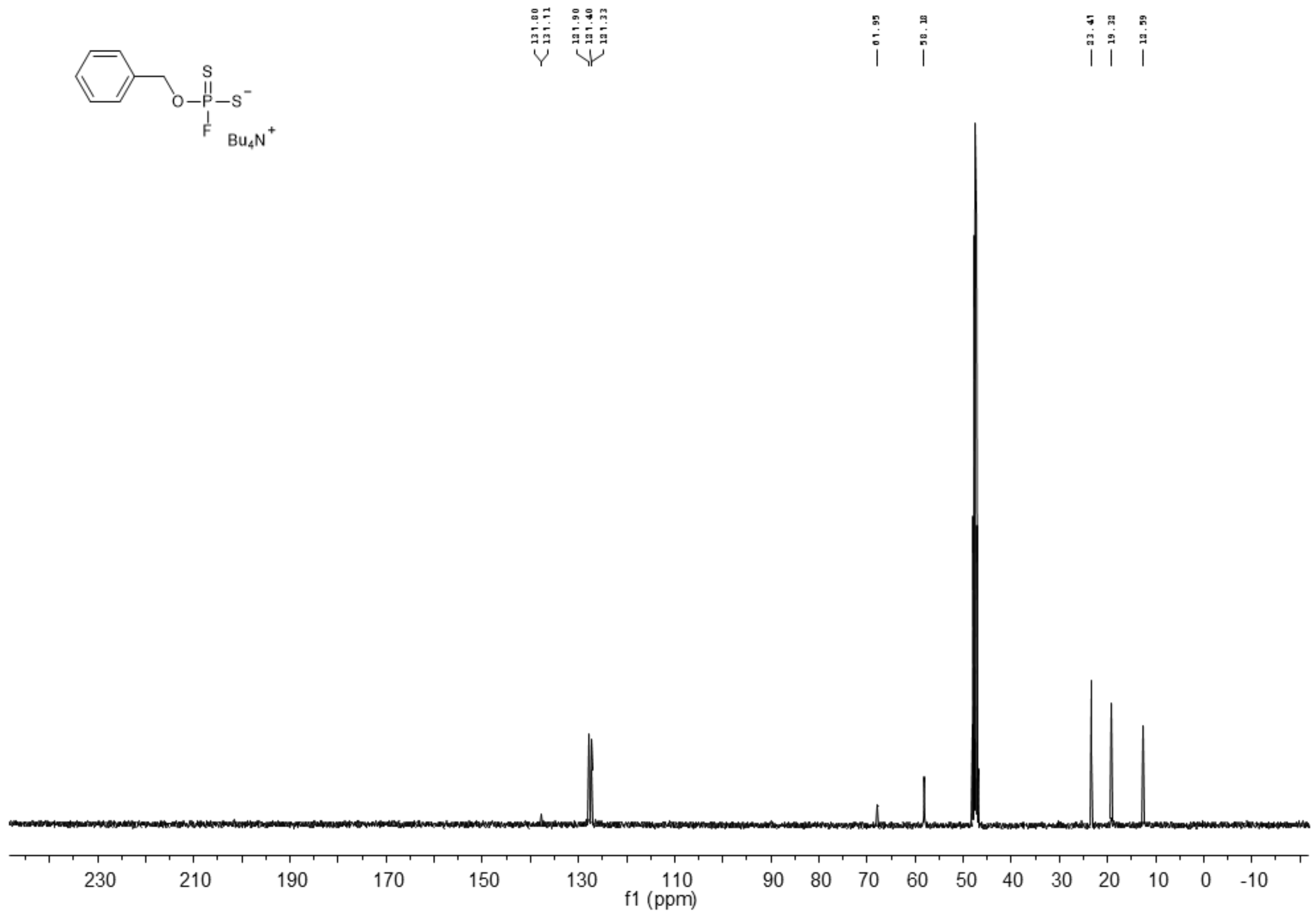

Figure A39. **^{13}C NMR (101 MHz, CD_3OD)** spectrum of **6**.

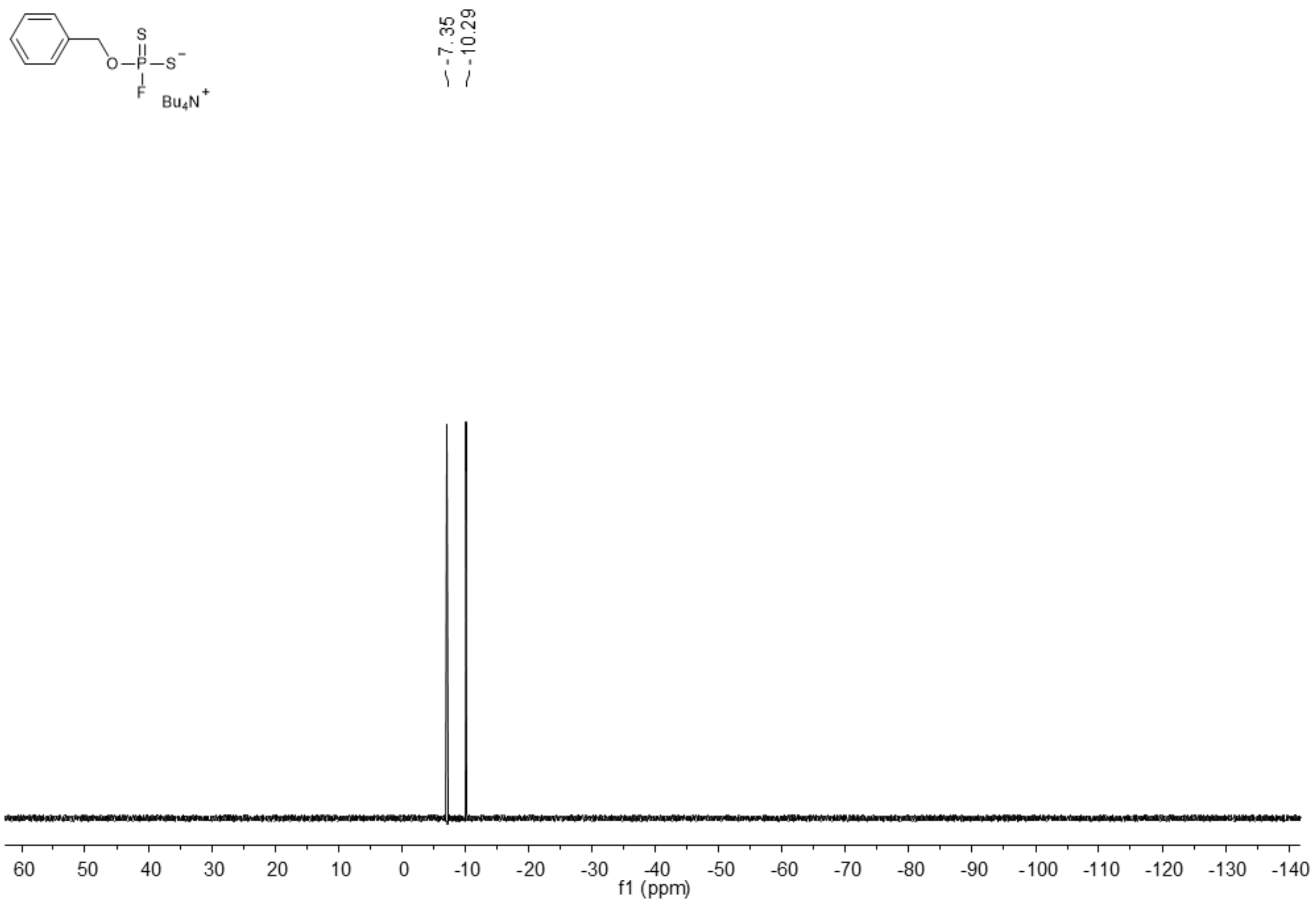

Figure A40. **^{19}F NMR (376 MHz, CD_3OD)** spectrum of **6**.

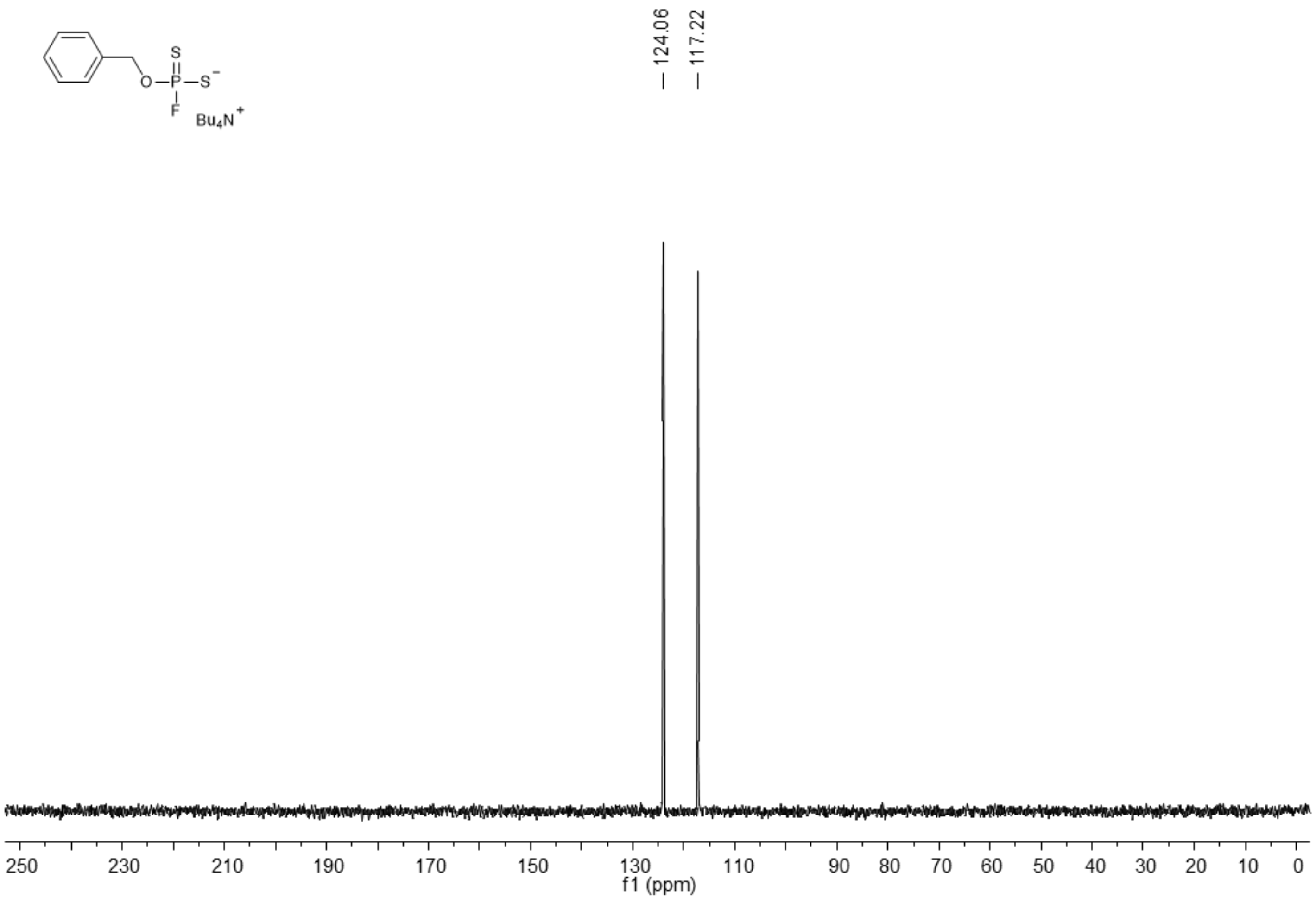

Figure A41. **^{31}P NMR (162 MHz, CD$_3$OD)** spectrum of **6**.

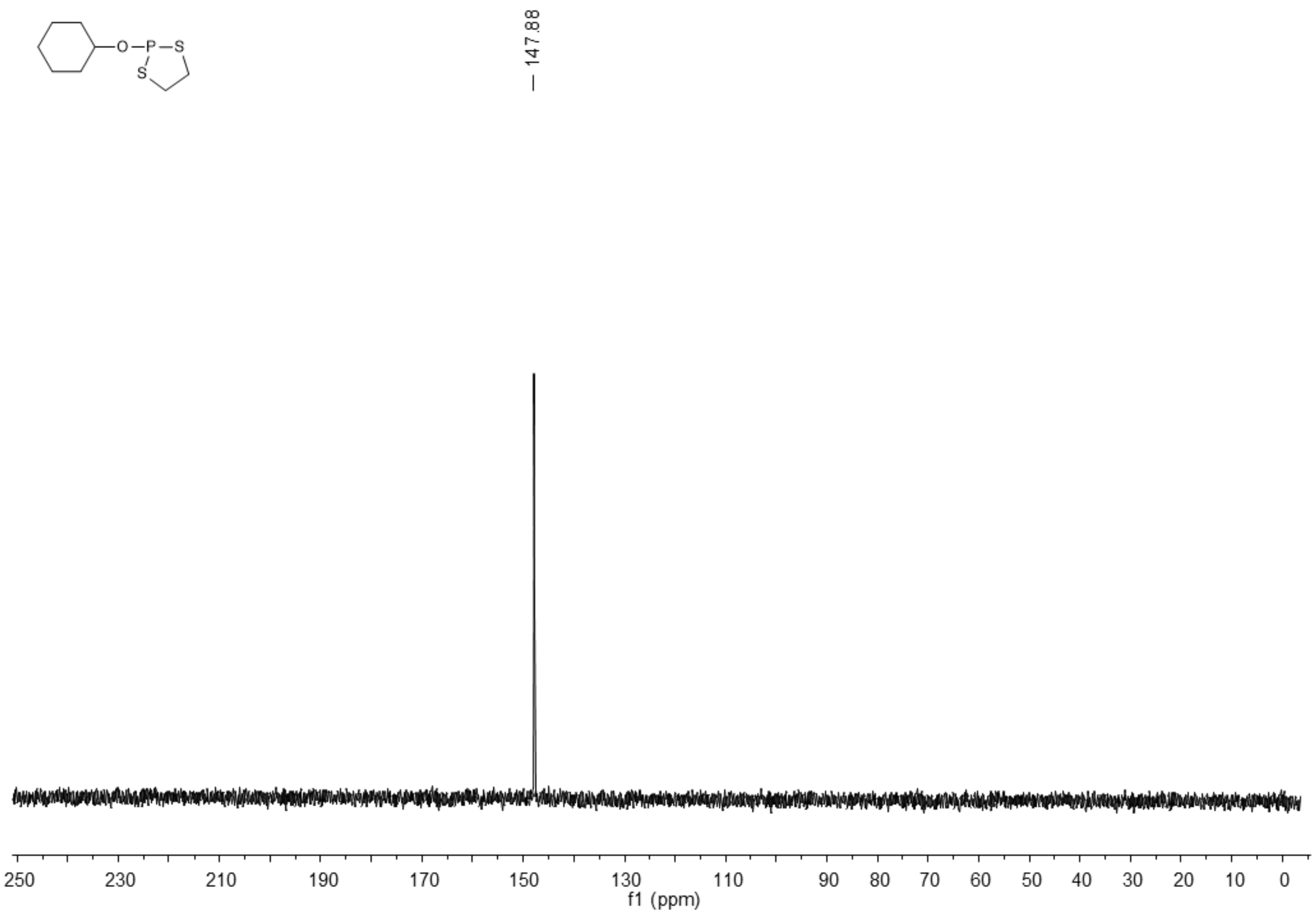

Figure A42. ^{31}**P NMR (162 MHz, $CDCl_3$)** spectrum of **7a**.

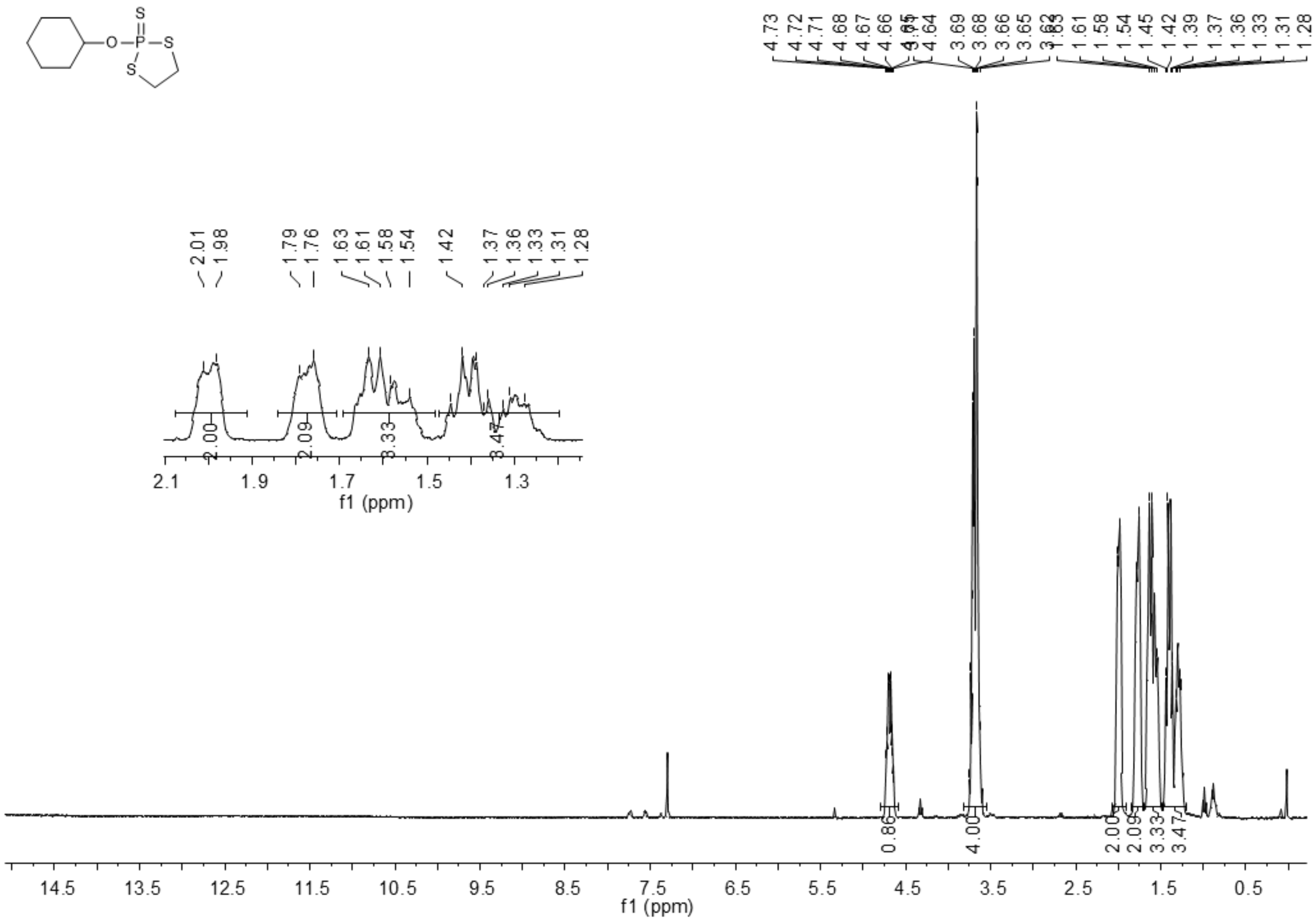

Figure A43. **^{1}H NMR (162 MHz, CDCl$_3$)** spectrum of **7b**.

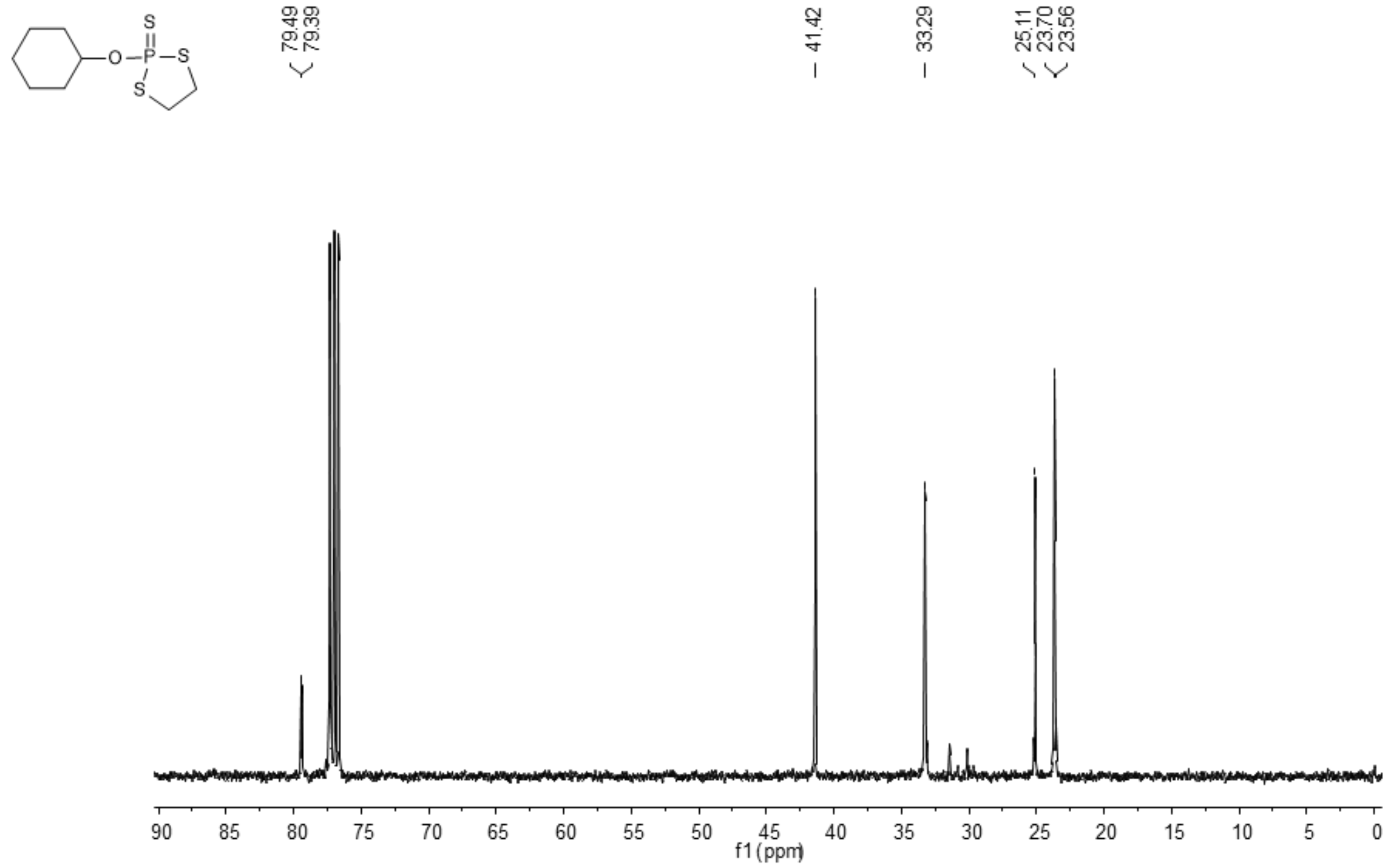

Figure A44. **^{13}C NMR (101 MHz, $CDCl_3$)** spectrum of **7b**.

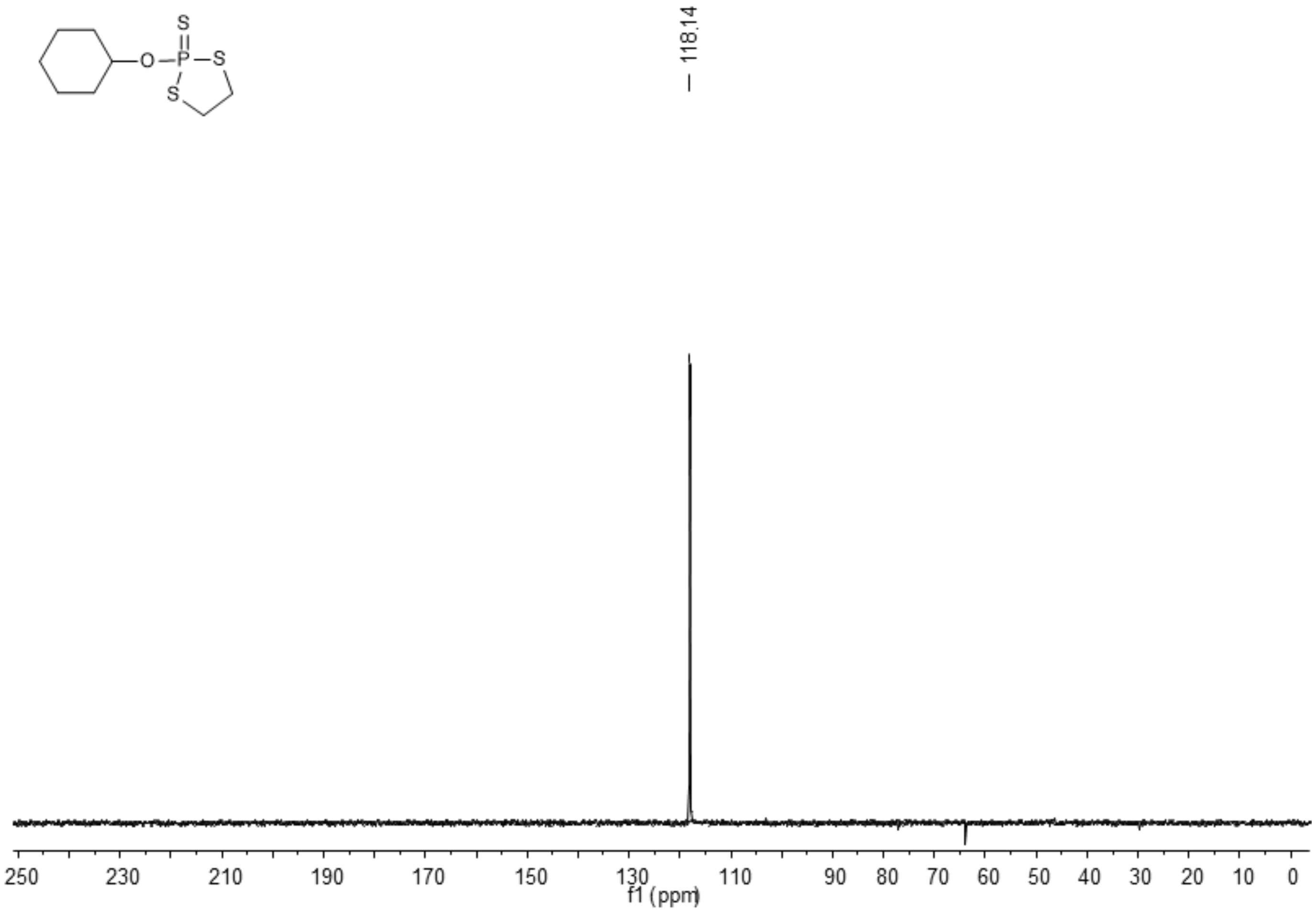

Figure A45. **^{31}P NMR (162 MHz, CDCl$_3$)** spectrum of **7b**.

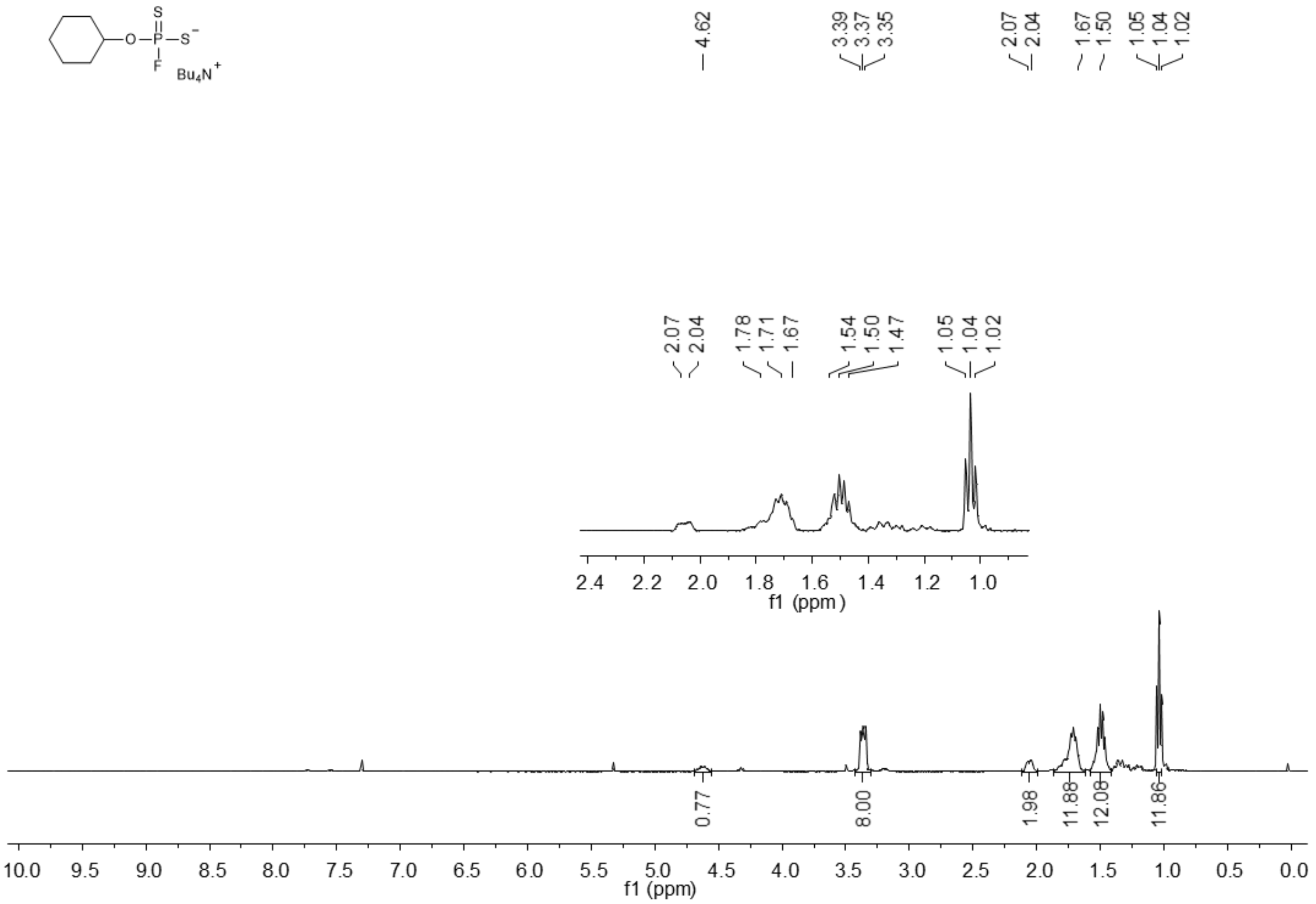

Figure A46. **^{1}H NMR (400 MHz, $CDCl_3$)** spectrum of **7**.

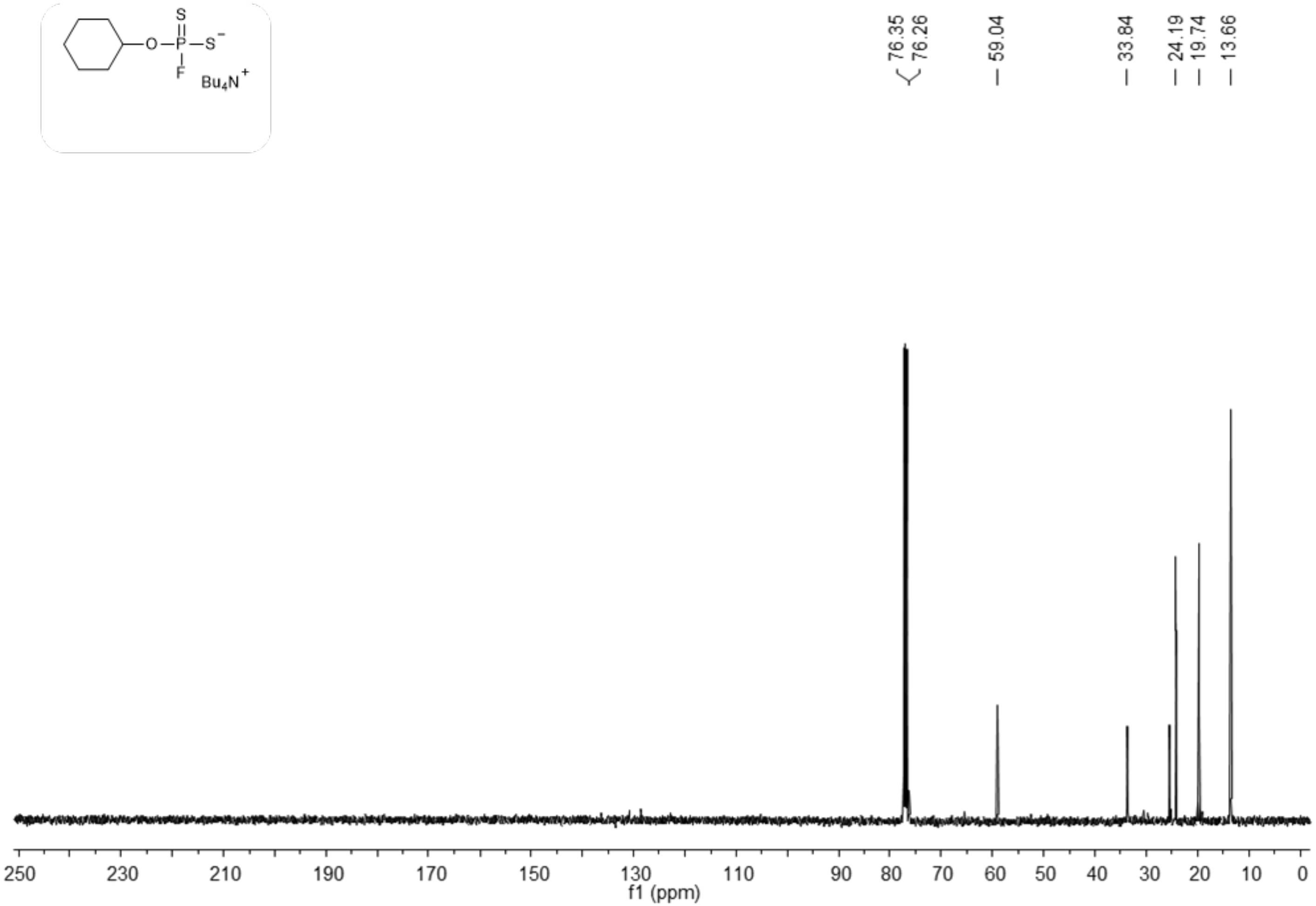

Figure A47. **^{13}C NMR (101 MHz, CDCl$_3$)** spectrum of **7**.

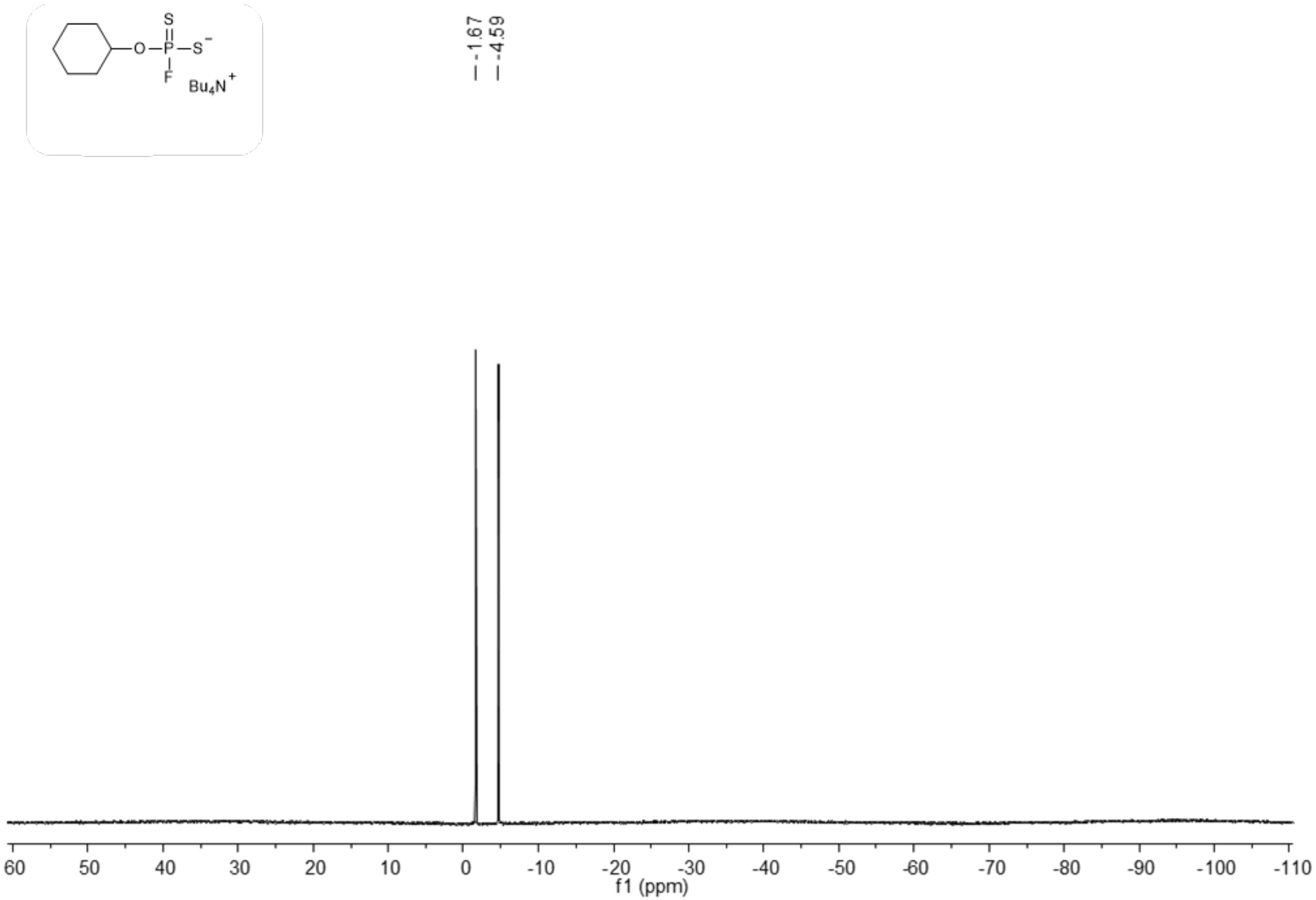

Figure A28. **^{19}F NMR (376 MHz, $CDCl_3$)** spectrum of **7**.

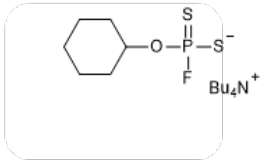

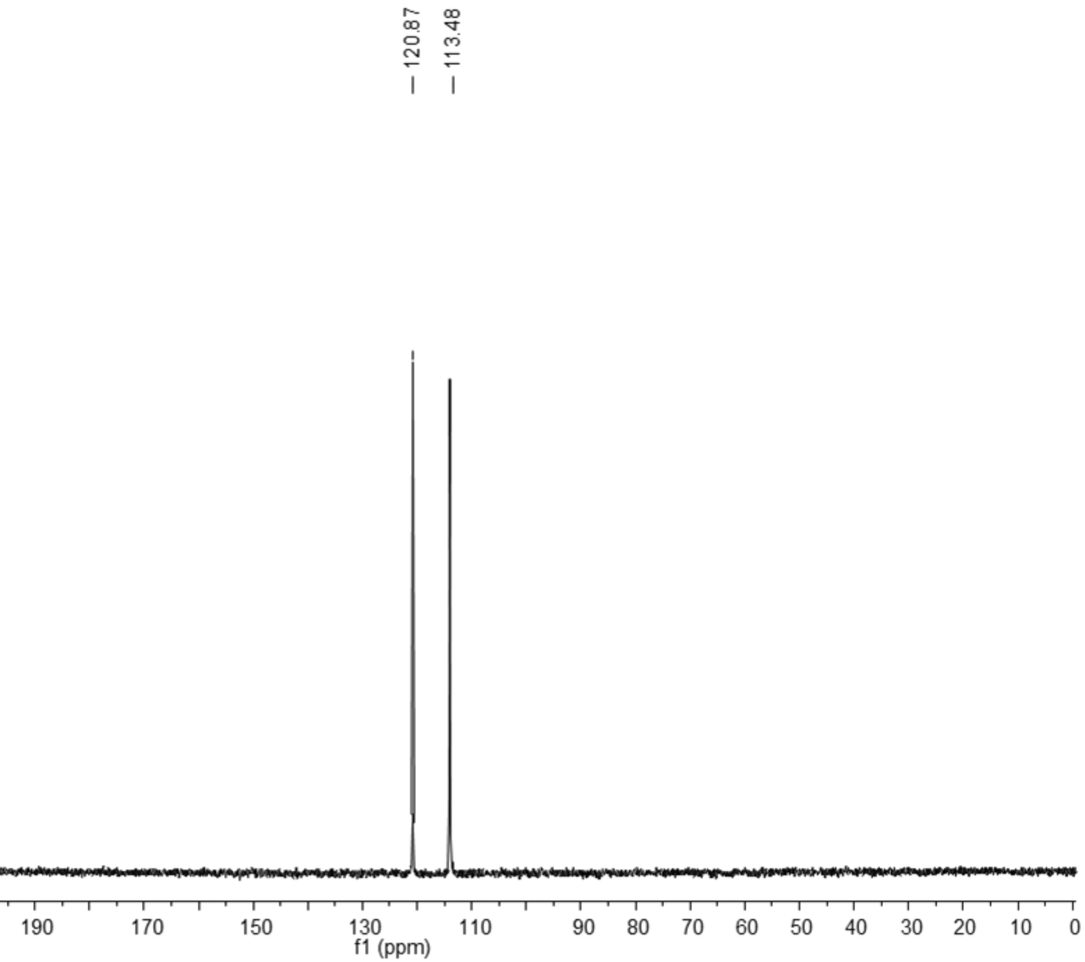

Figure A49. **^{31}P NMR (162 MHz, $CDCl_3$)** spectrum of **7**.

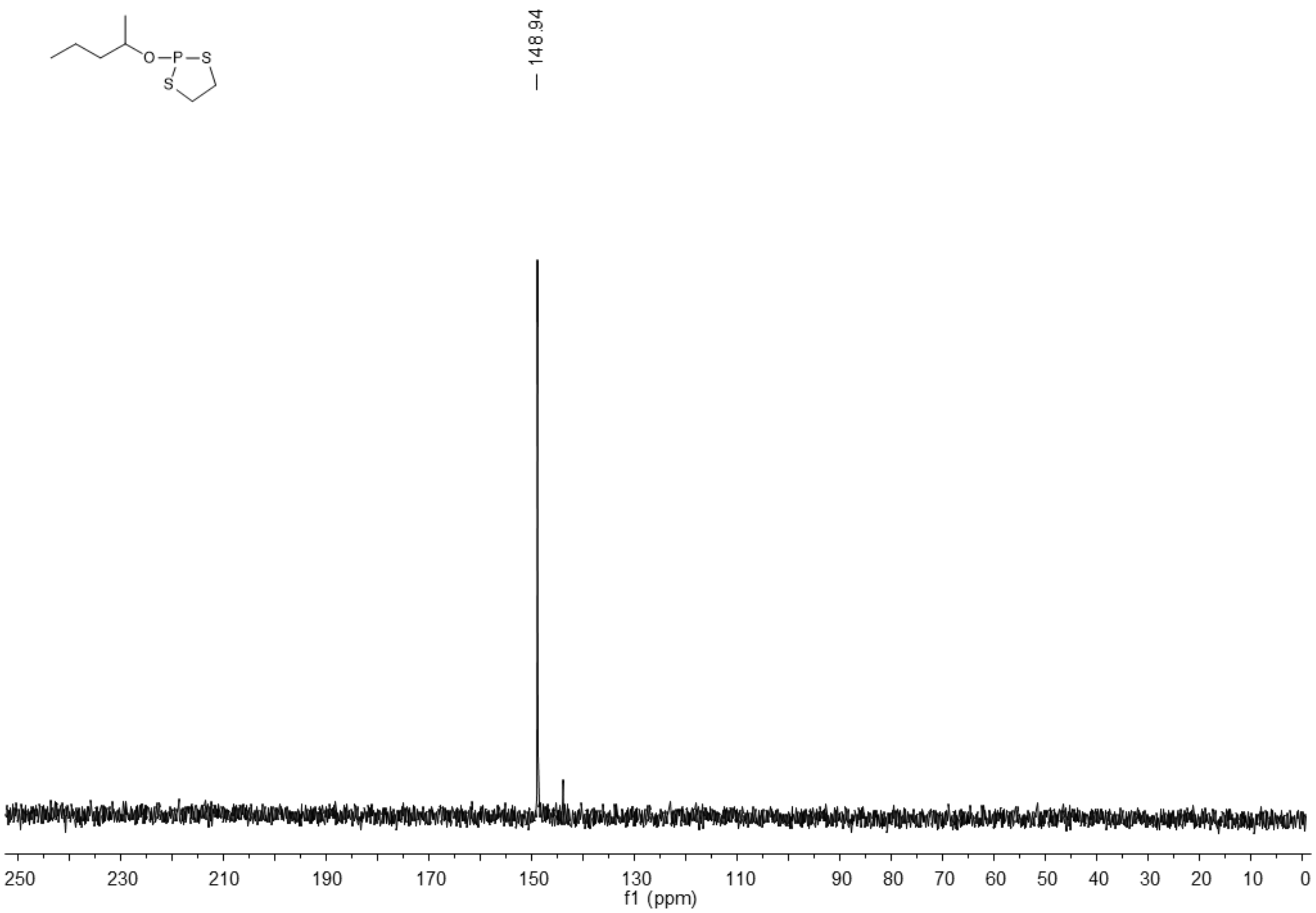

Figure A50. **^{31}P NMR (162 MHz, $CDCl_3$)** spectrum of **8a**.

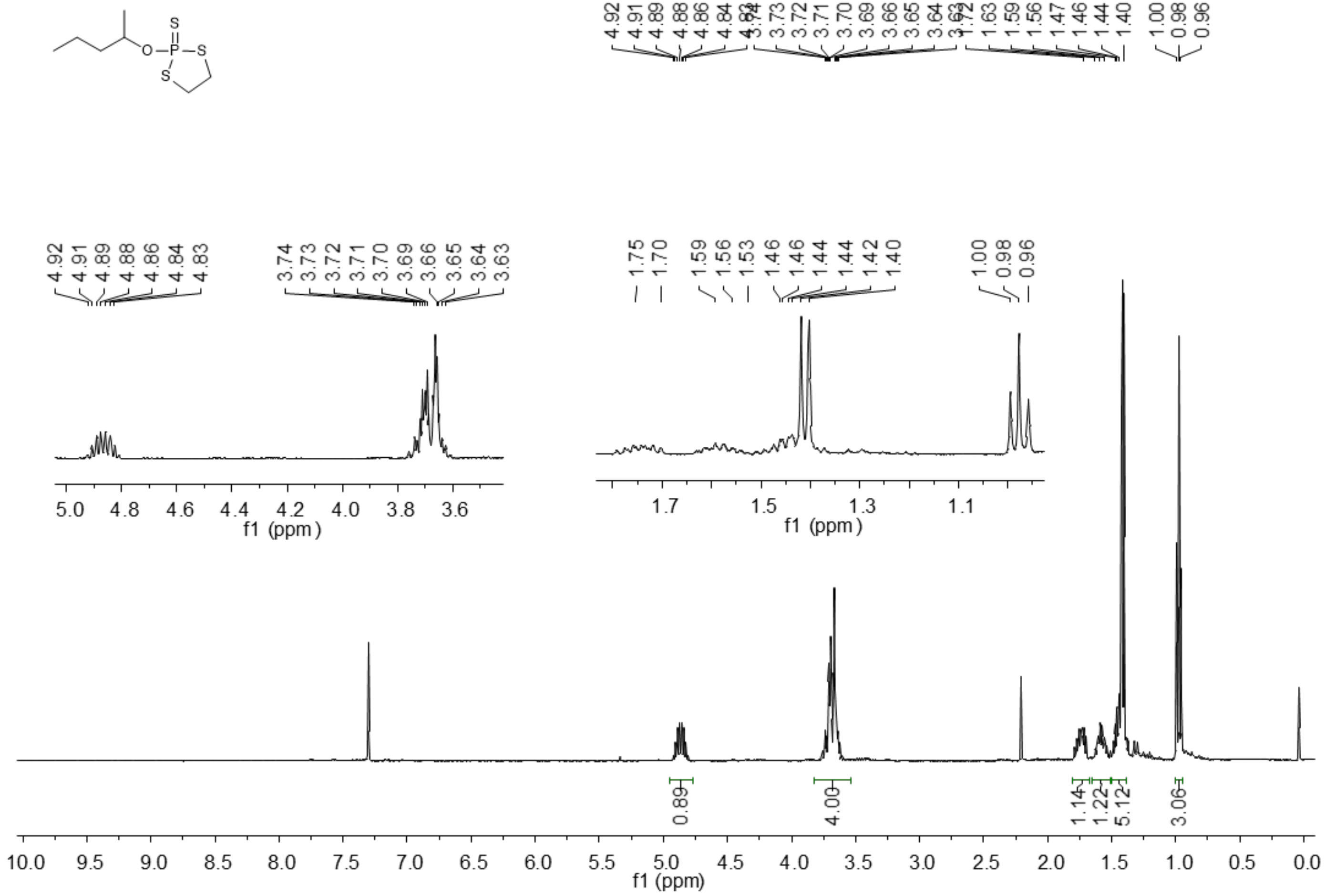

Figure A51. **^{1}H NMR (400 MHz, $CDCl_3$)** spectrum of **8b**.

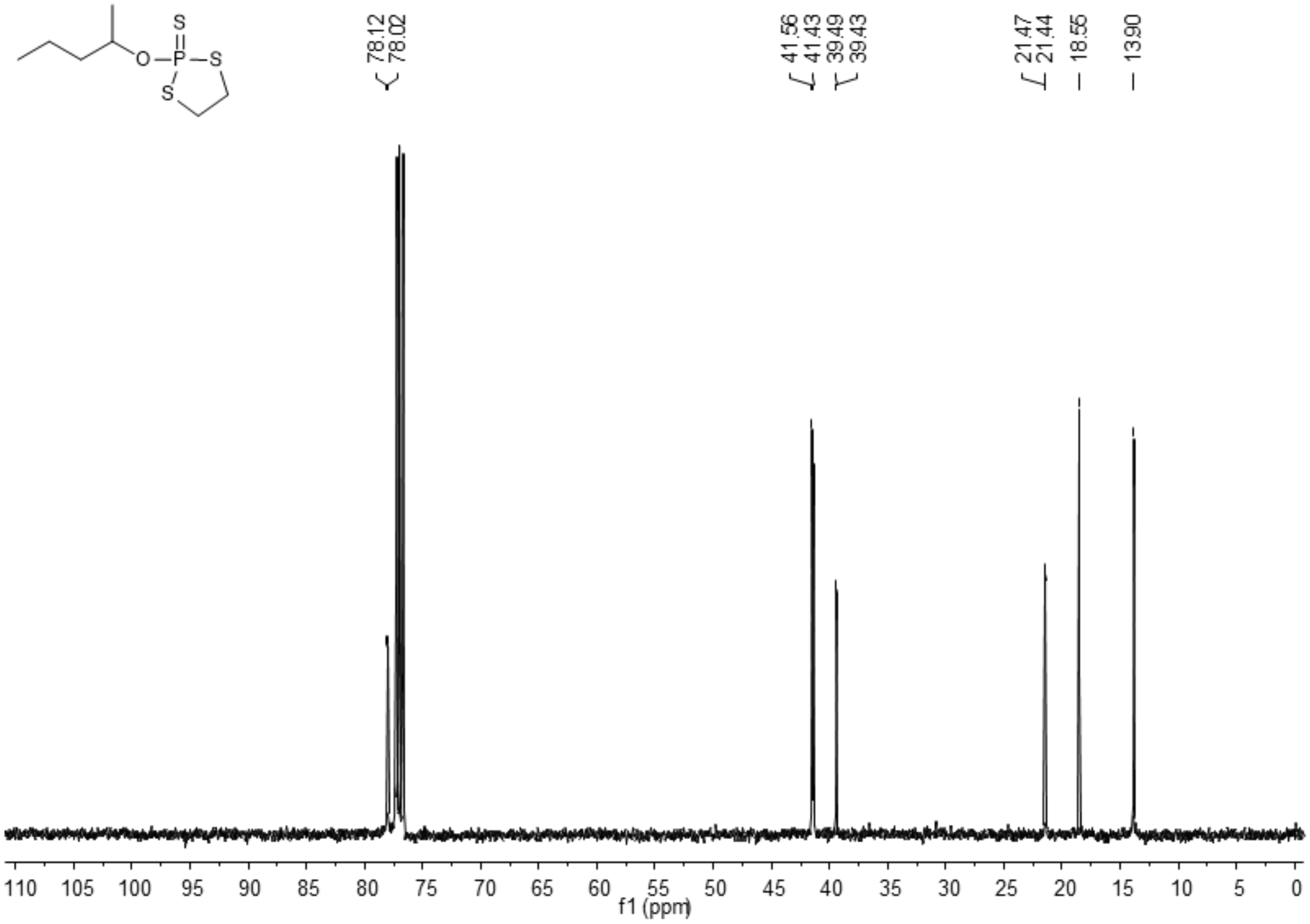

Figure A52. **^{13}C NMR (101 MHz, CDCl$_3$)** spectrum of **8b**.

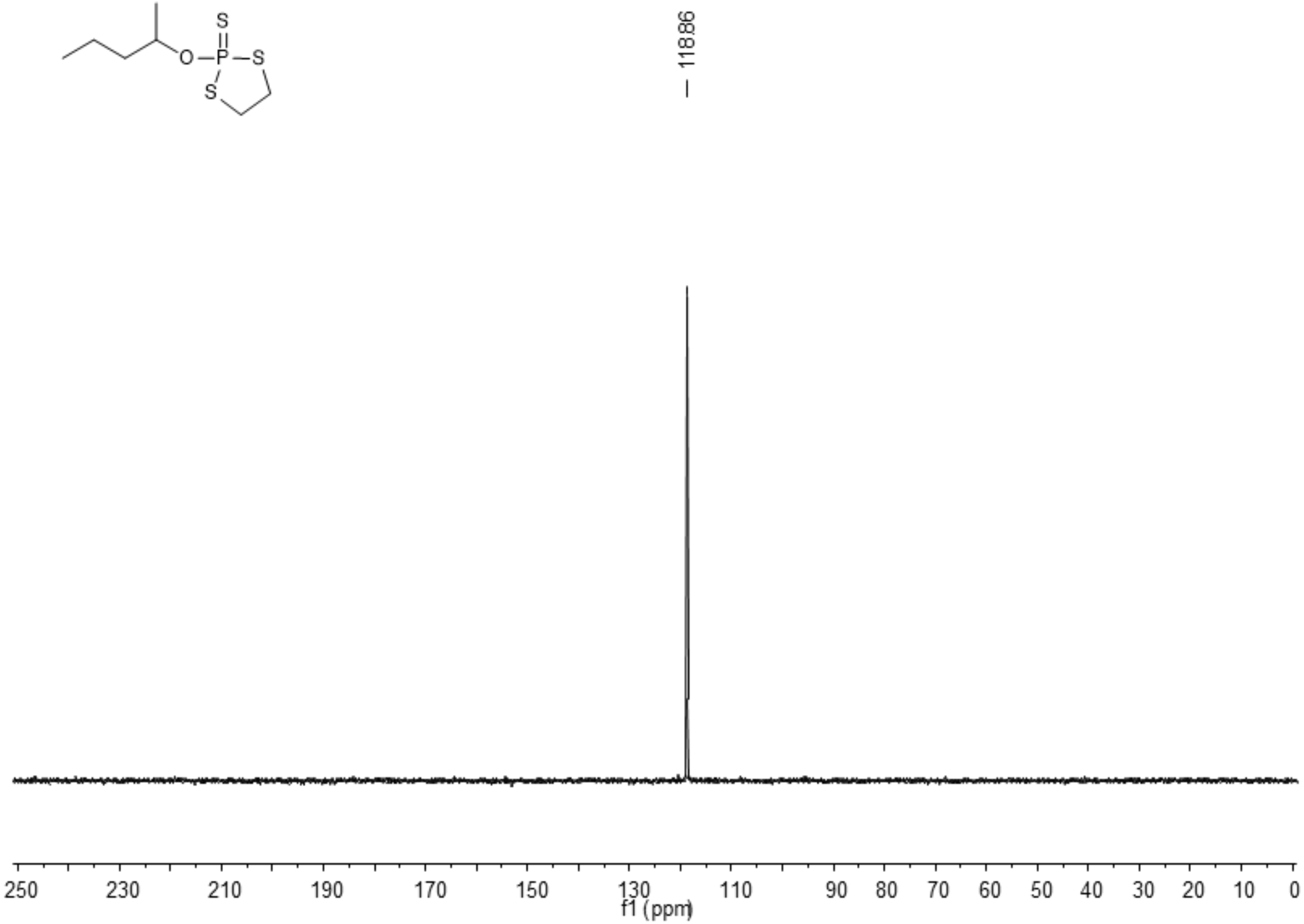

Figure A53. ^{31}**P NMR (162 MHz, $CDCl_3$)** spectrum of **8b**.

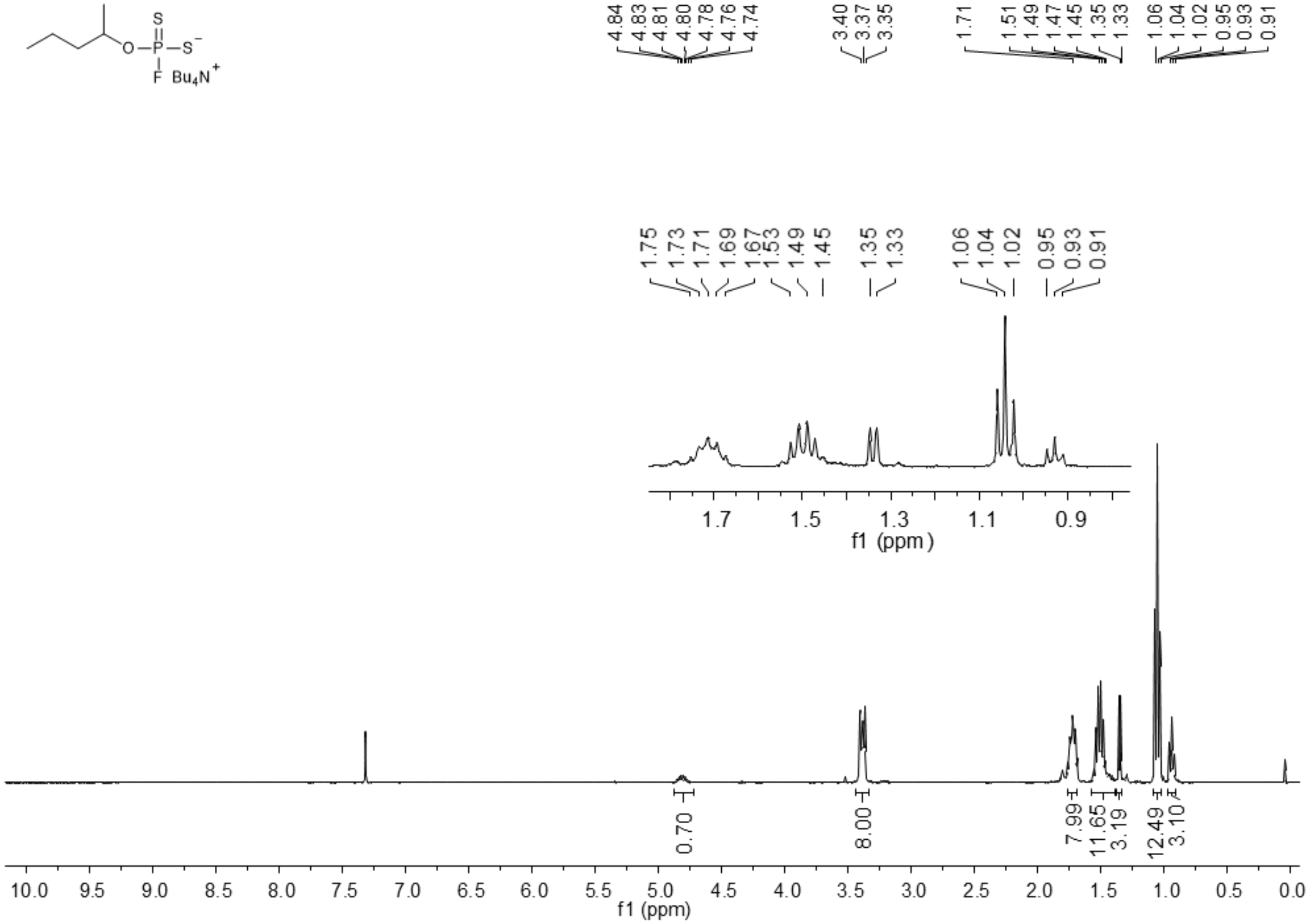

Figure A54. **^{1}H NMR (400 MHz, $CDCl_3$)** spectrum of **8**.

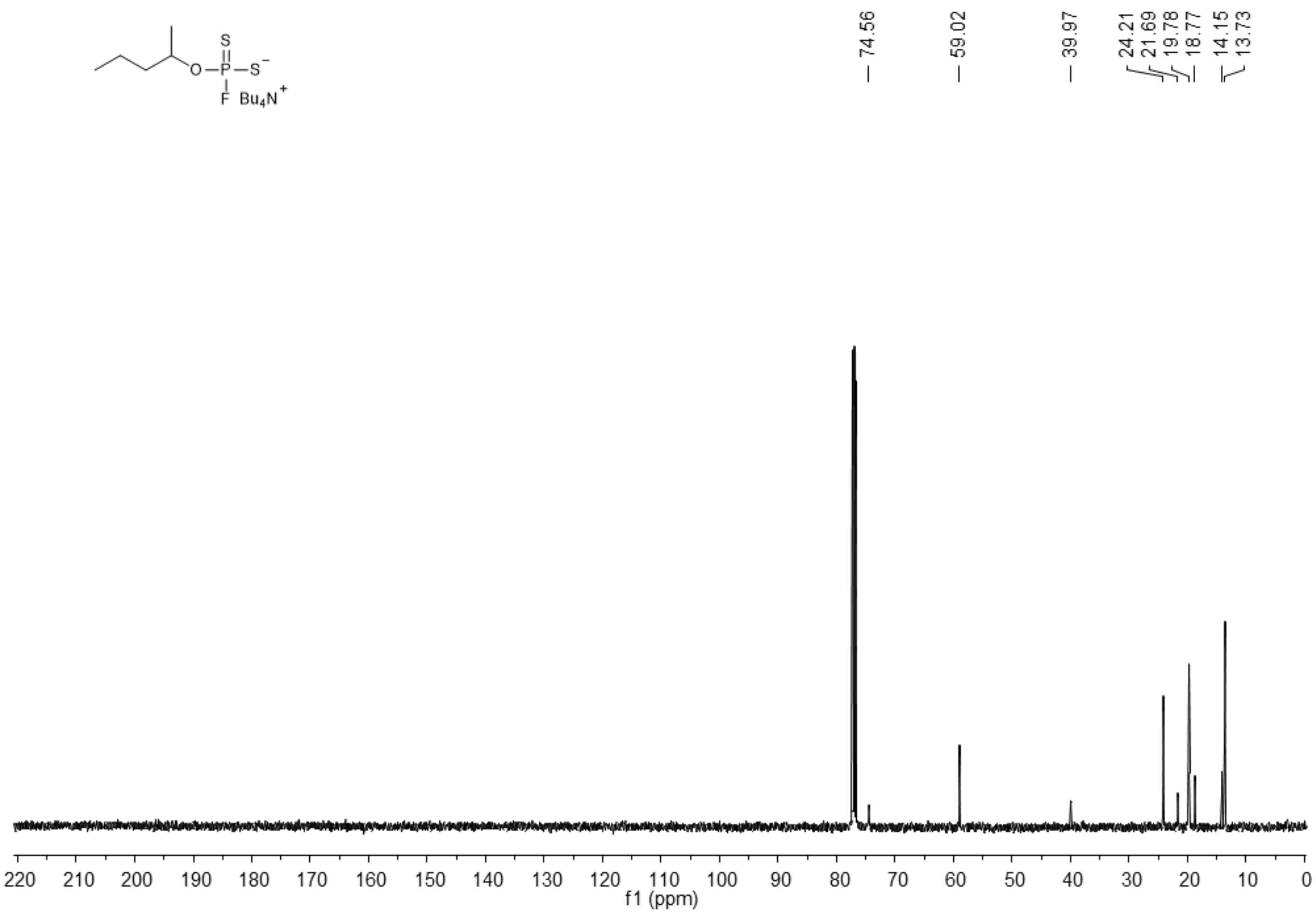

Figure A55. **^{13}C NMR (101 MHz, $CDCl_3$)** spectrum of **8**.

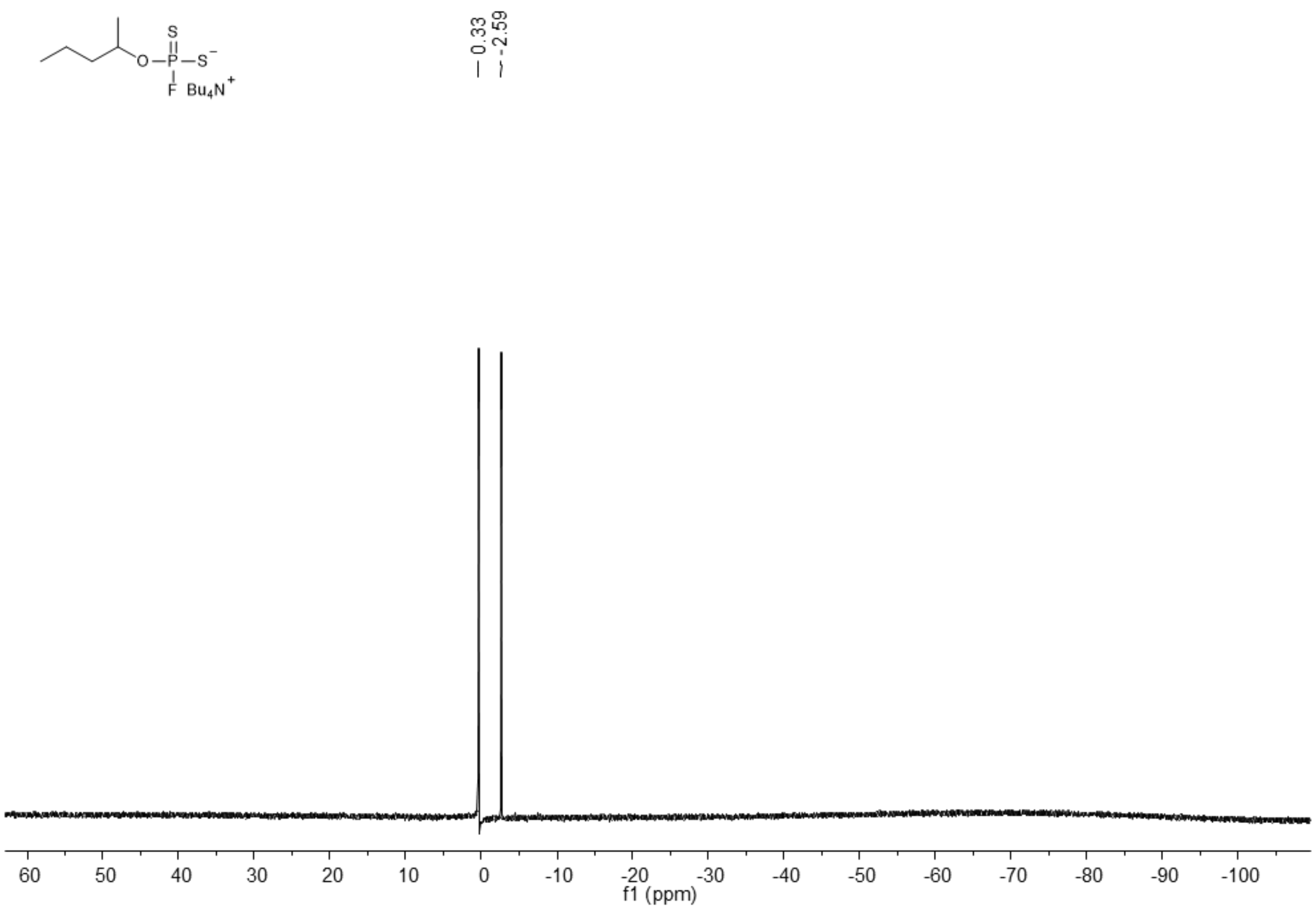

Figure A56. **^{19}F NMR (376 MHz, $CDCl_3$)** spectrum of **8**.

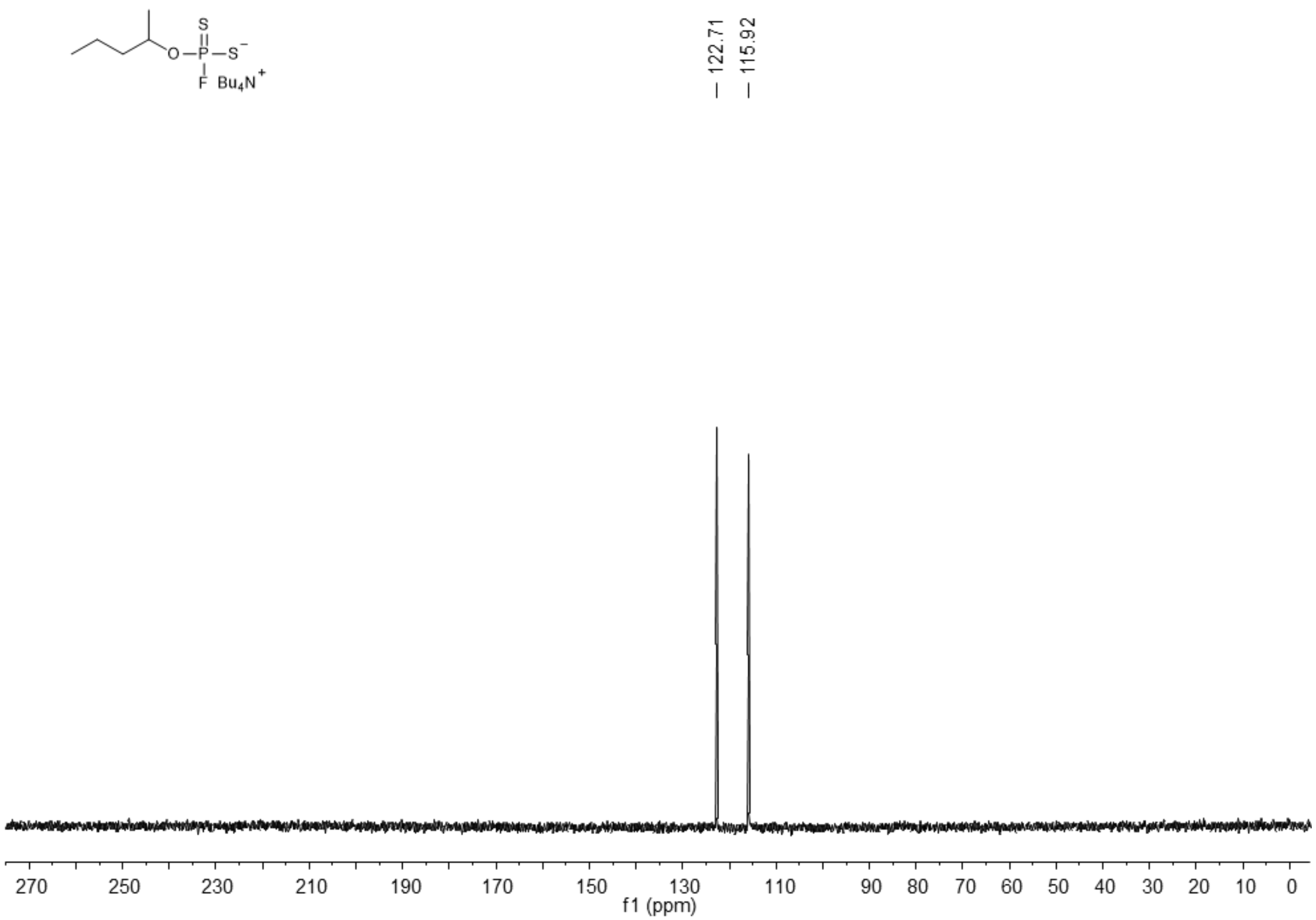

Figure A57. **^{31}P NMR (162 MHz, $CDCl_3$)** spectrum of **8**.

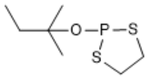

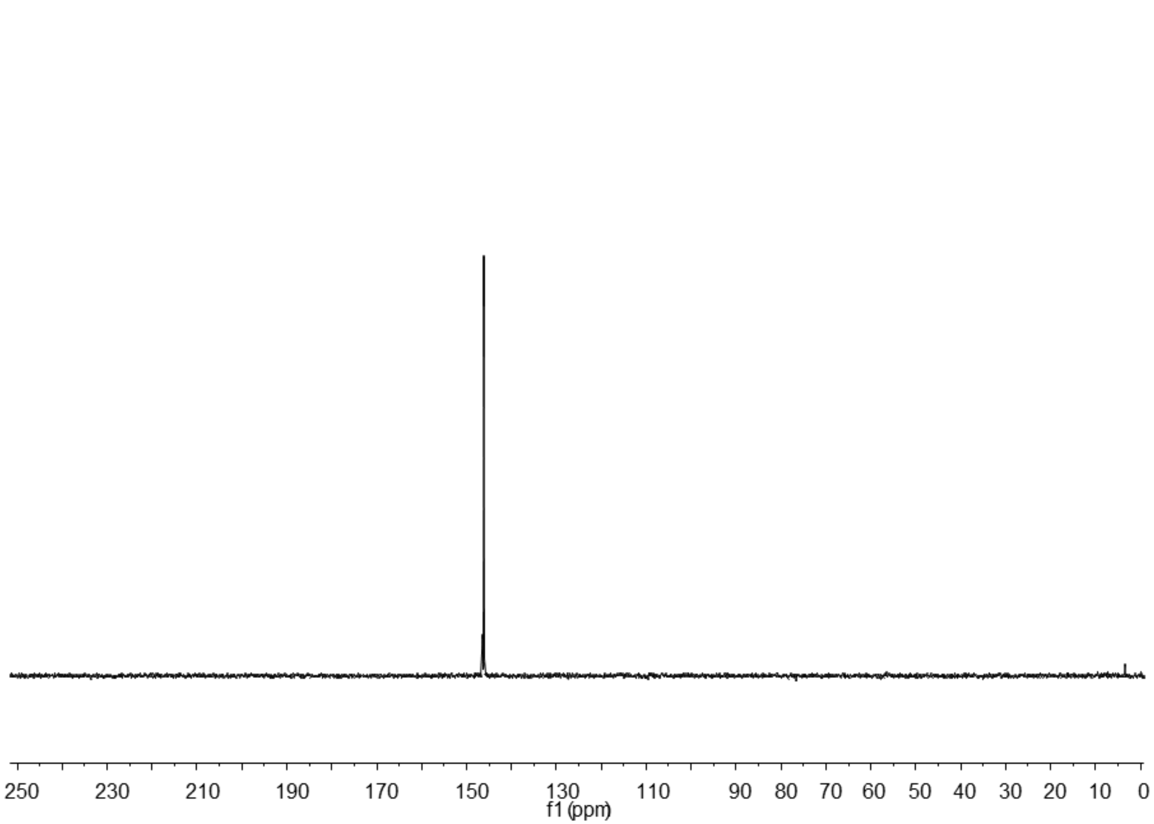

Figure A58. **^{31}P NMR (162 MHz, $CDCl_3$)** spectrum of **9a**.

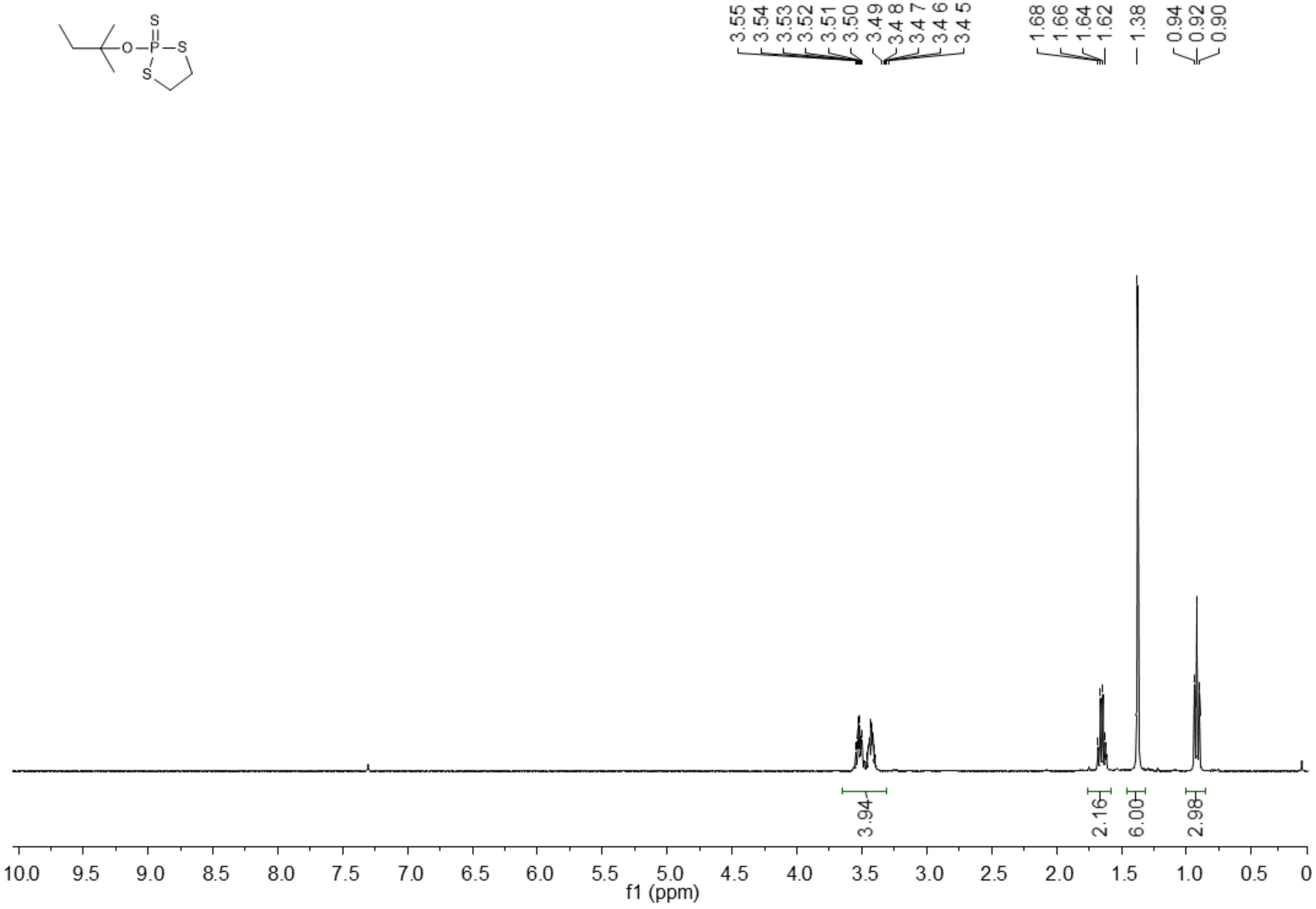

Figure A59. [1]**H NMR (400 MHz, $CDCl_3$)** spectrum of **9b**.

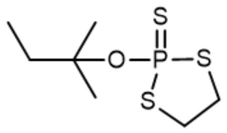

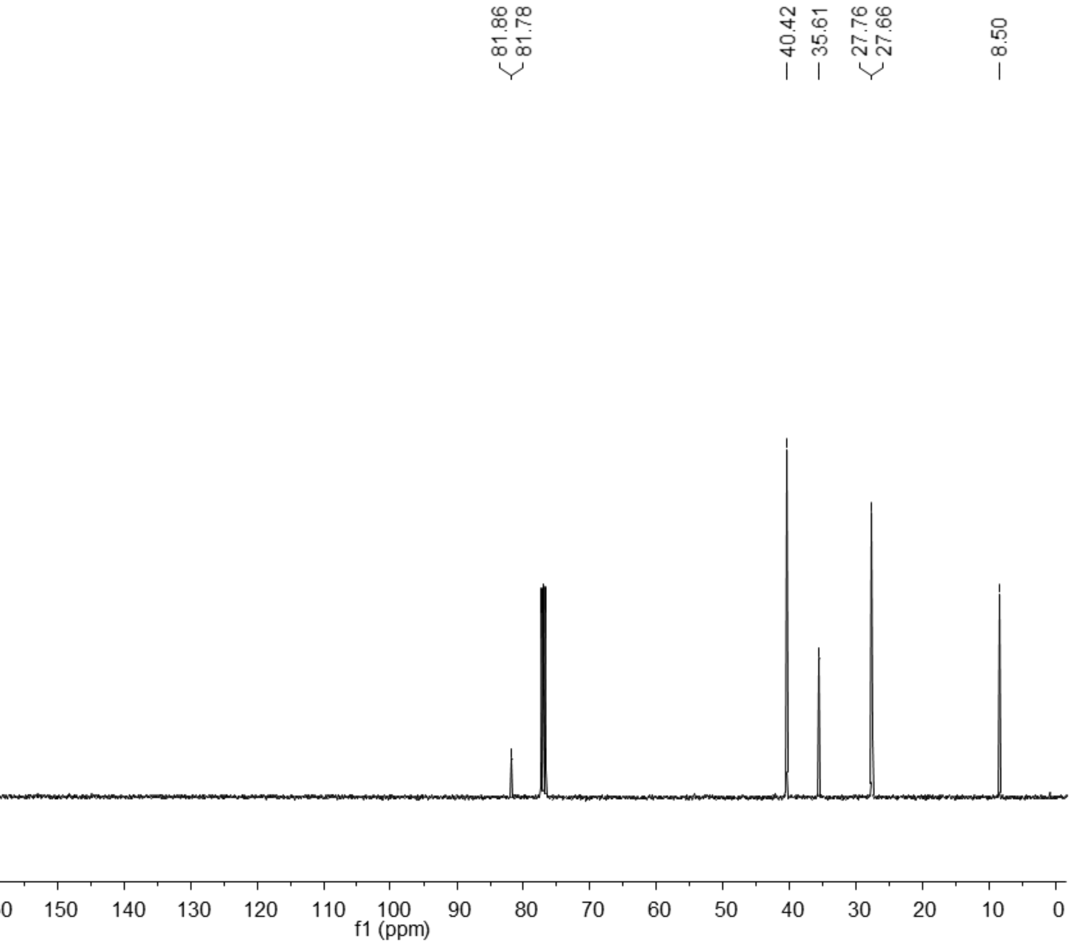

Figure A60. **^{13}C NMR (101 MHz, $CDCl_3$)** spectrum of **9b**.

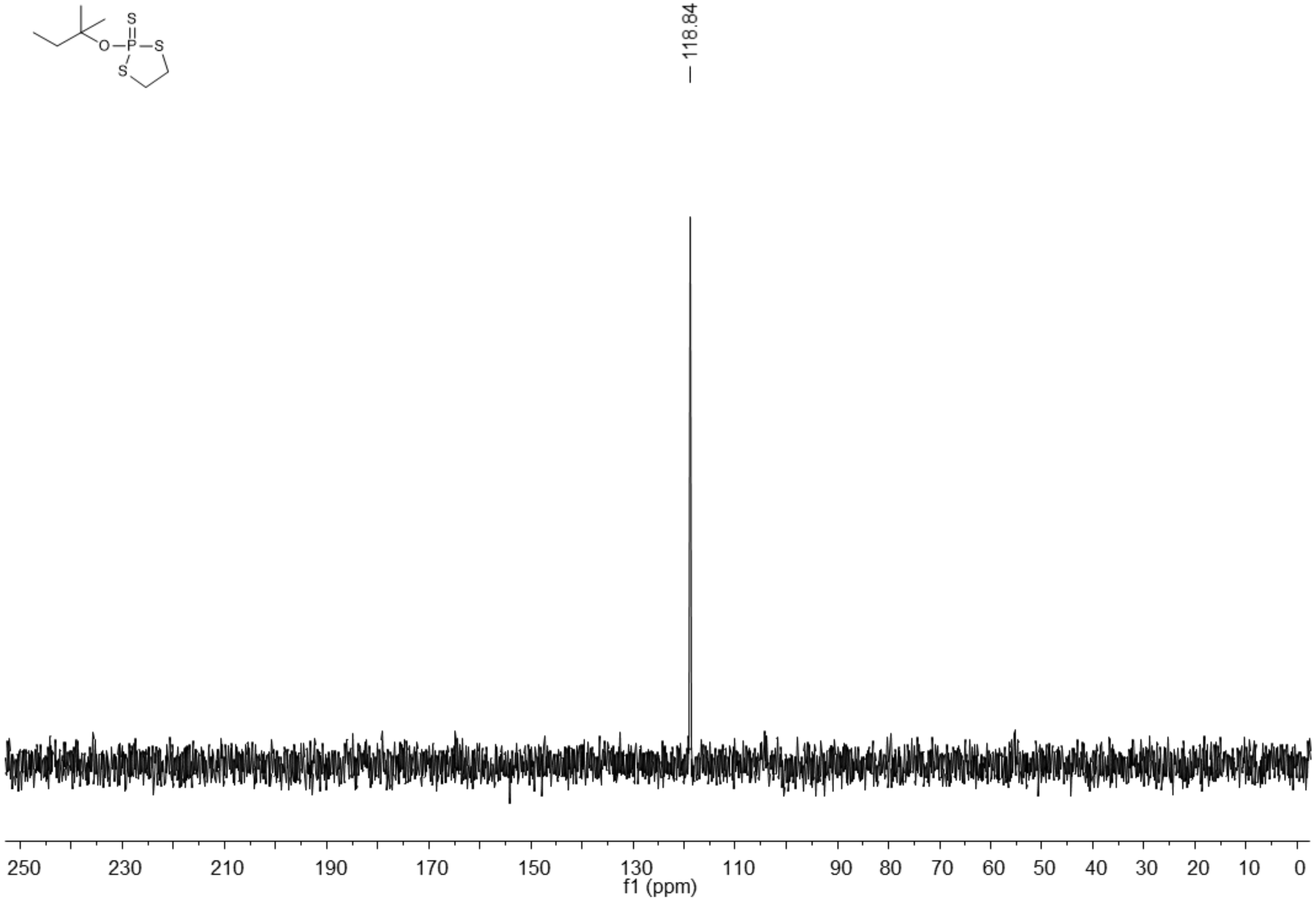

Figure A61. **^{31}P NMR (162 MHz, $CDCl_3$)** spectrum of **9b**.

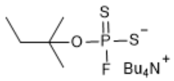

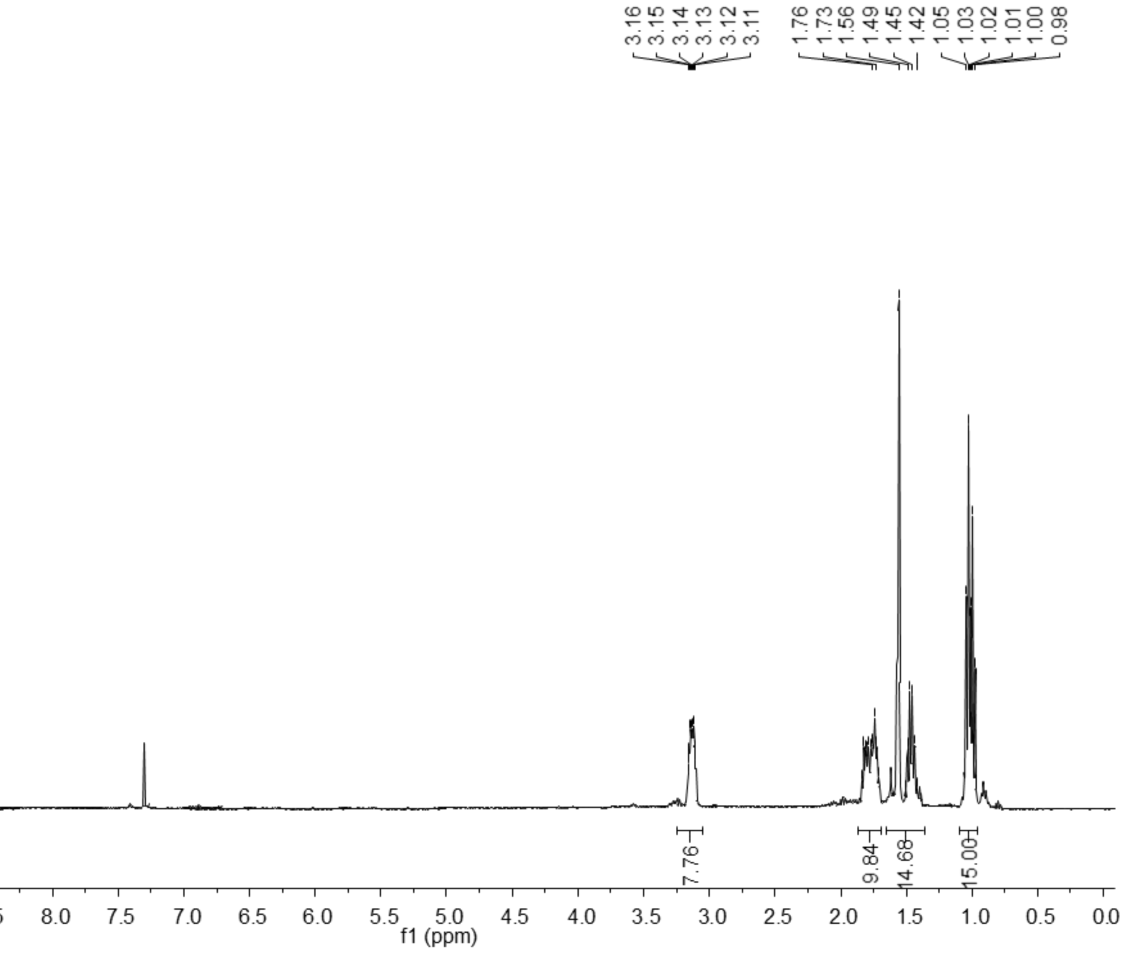

Figure A62. **^{1}H NMR (400 MHz, $CDCl_3$)** spectrum of **9**.

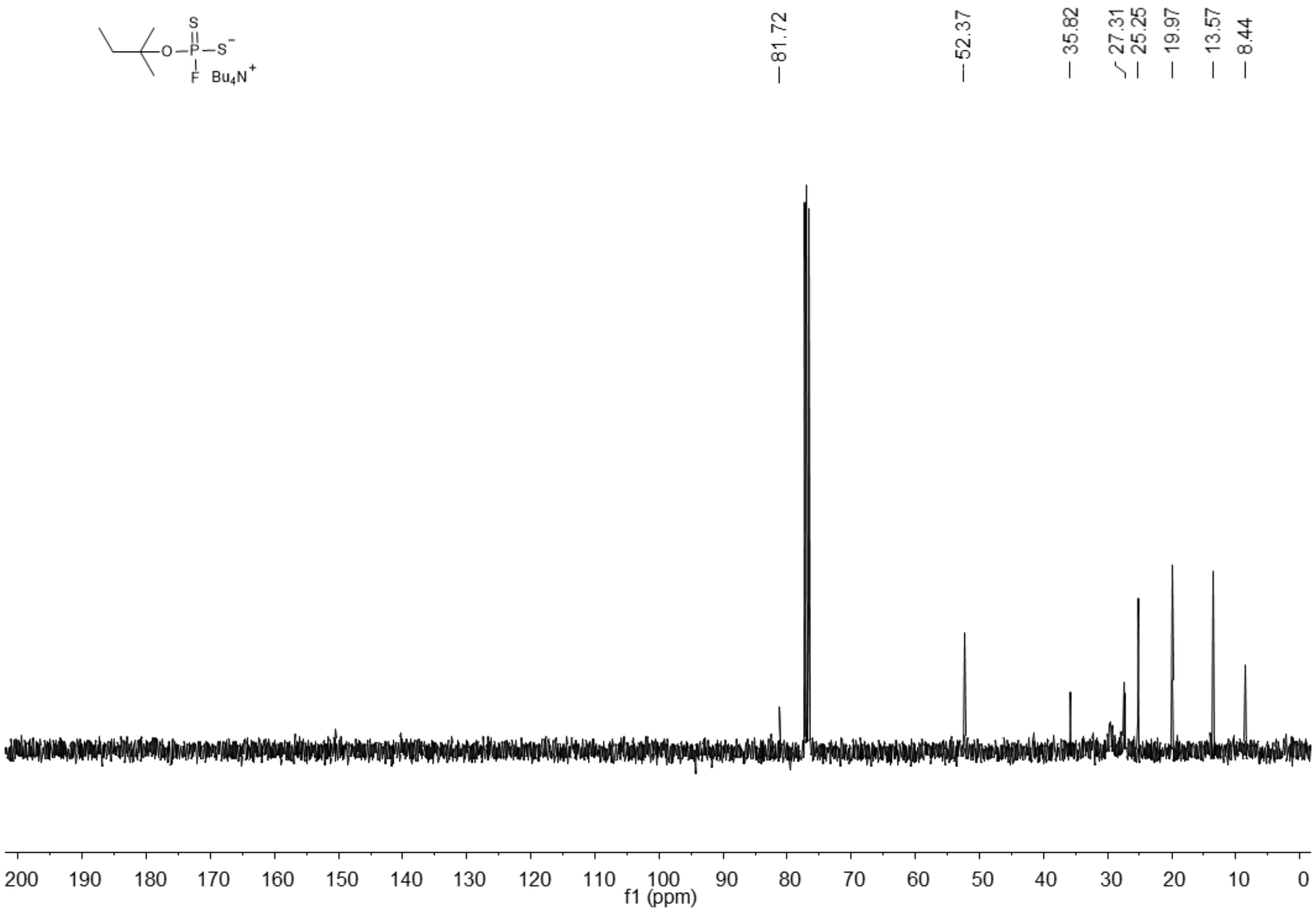

Figure A63. **^{13}C NMR (101 MHz, $CDCl_3$)** spectrum of **9**.

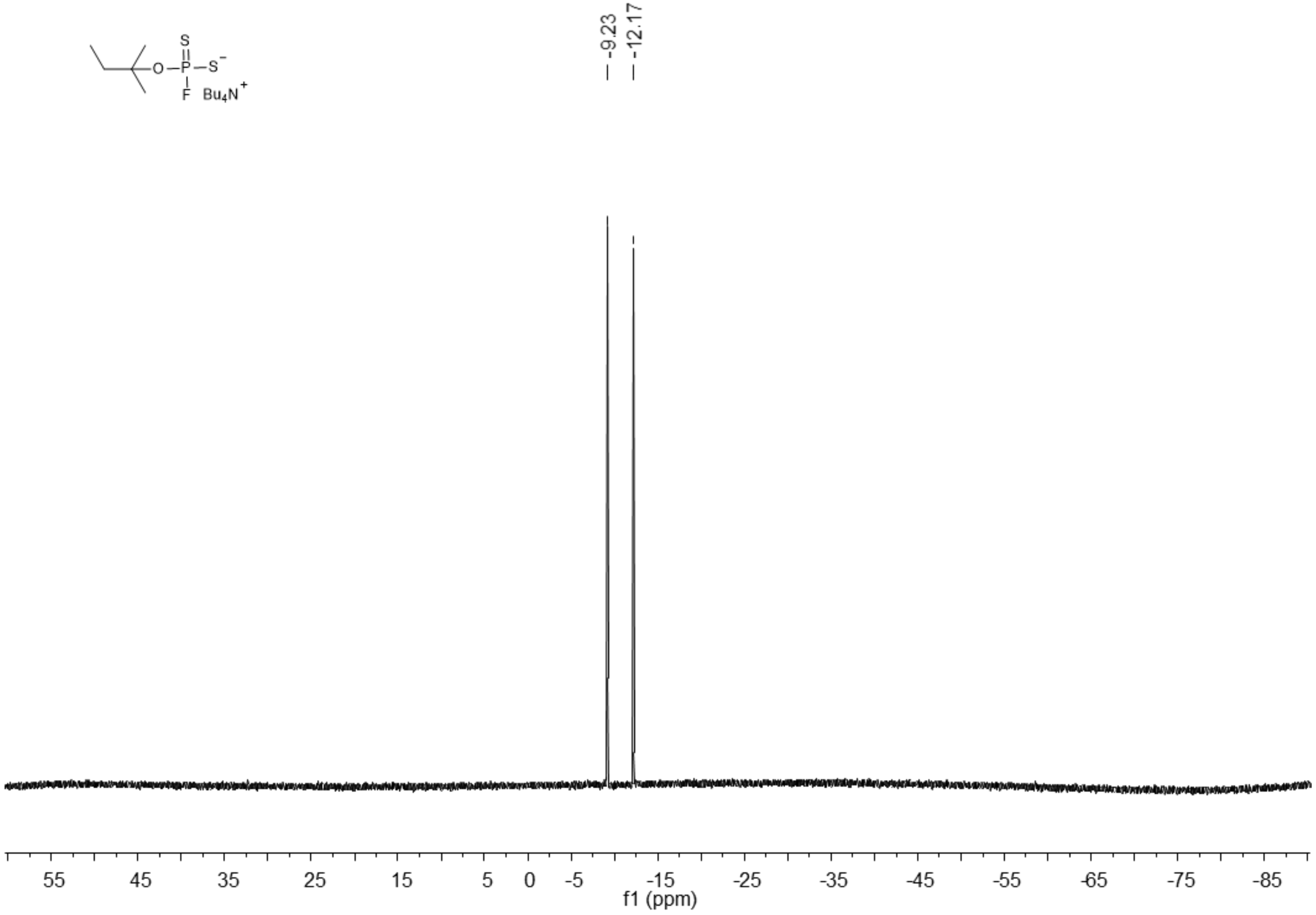

Figure A64. **^{19}F NMR (376 MHz, $CDCl_3$)** spectrum of **9**.

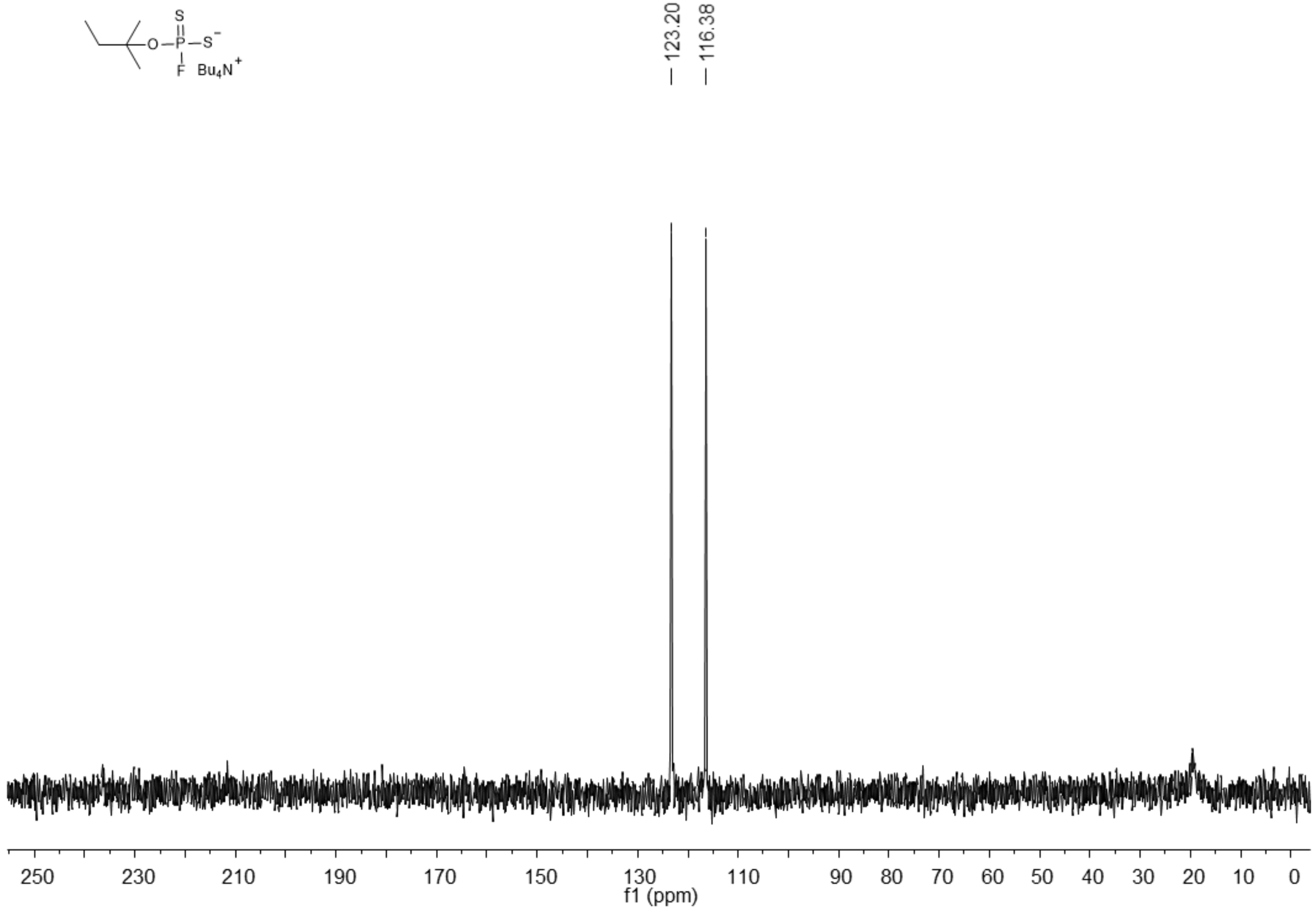

Figure A65. **^{31}P NMR (162 MHz, $CDCl_3$)** spectrum of **9**.

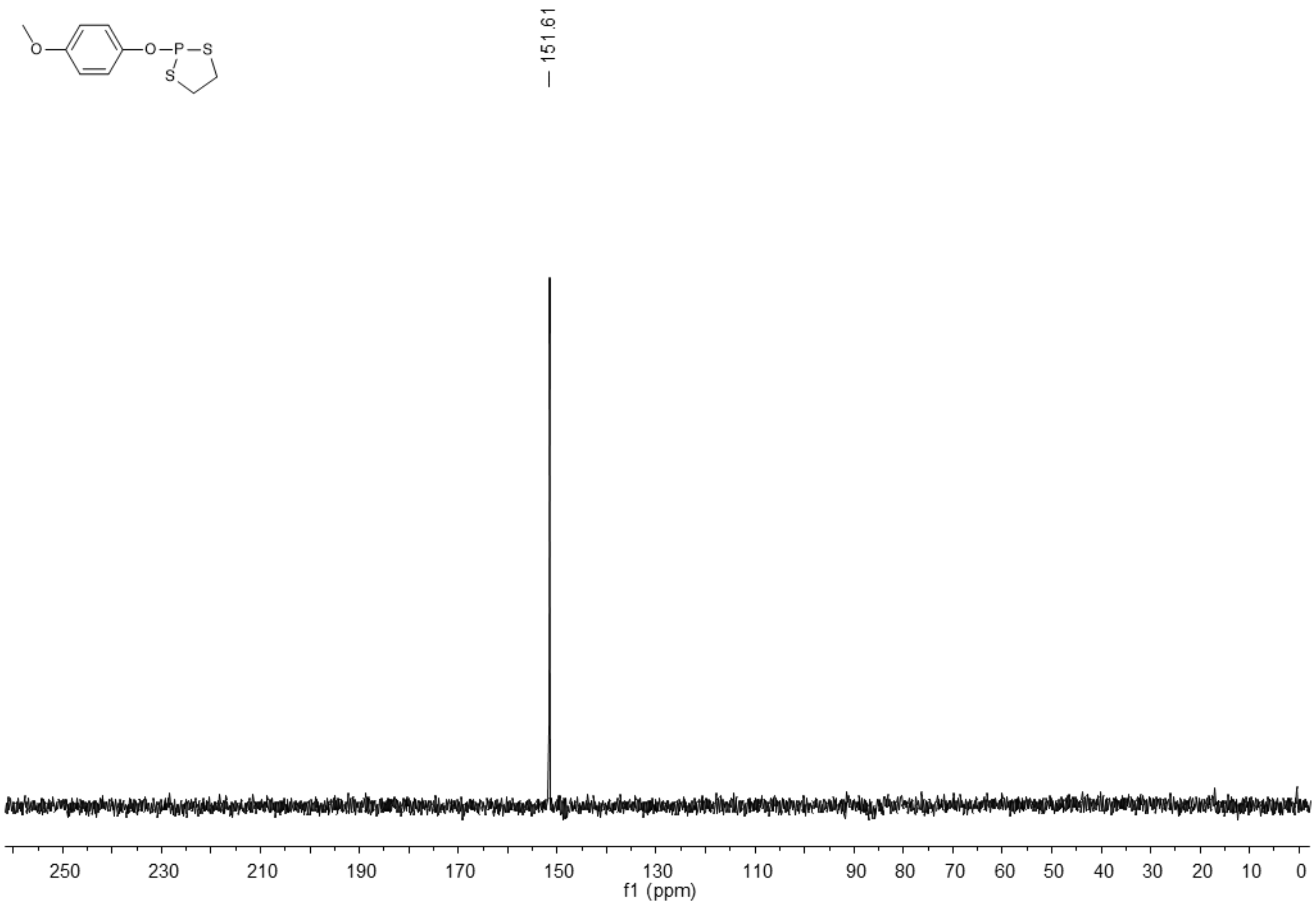

Figure A66. **^{31}P NMR (162 MHz, $CDCl_3$)** spectrum of **10a**.

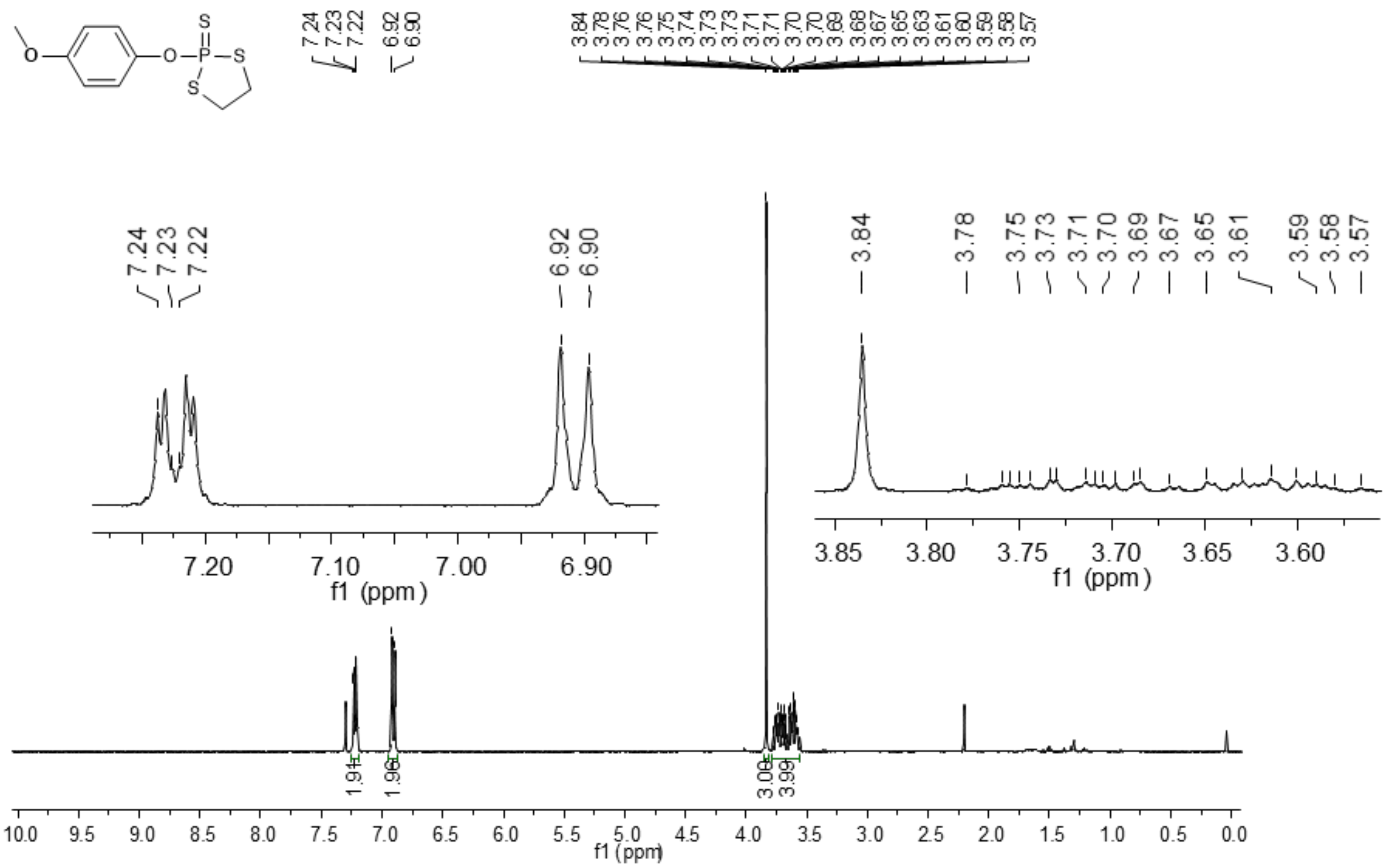

Figure A67. **^{1}H NMR (400 MHz, $CDCl_3$)** spectrum of **10b**.

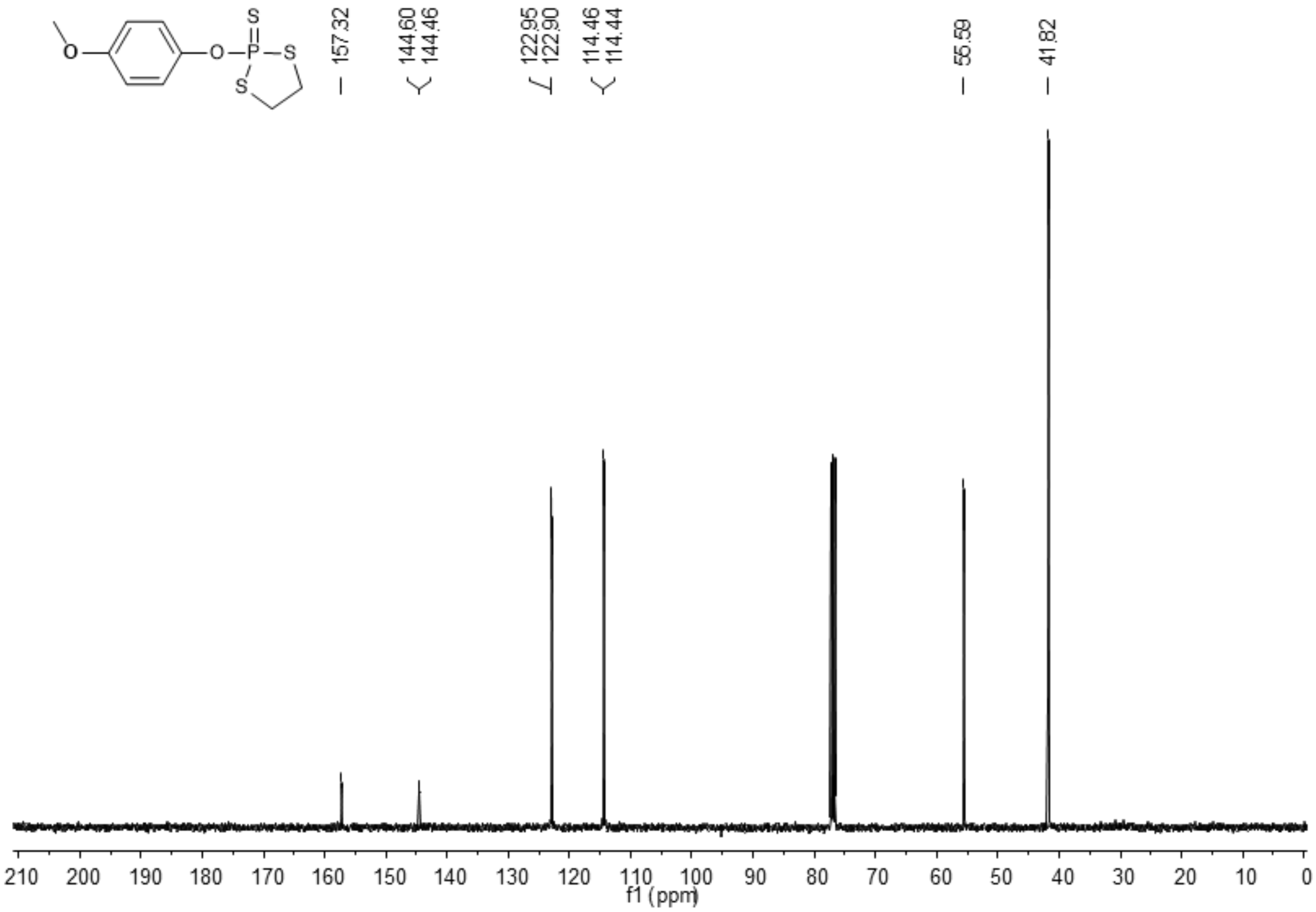

Figure A68. **^{13}C NMR (101 MHz, $CDCl_3$)** spectrum of **10b**.

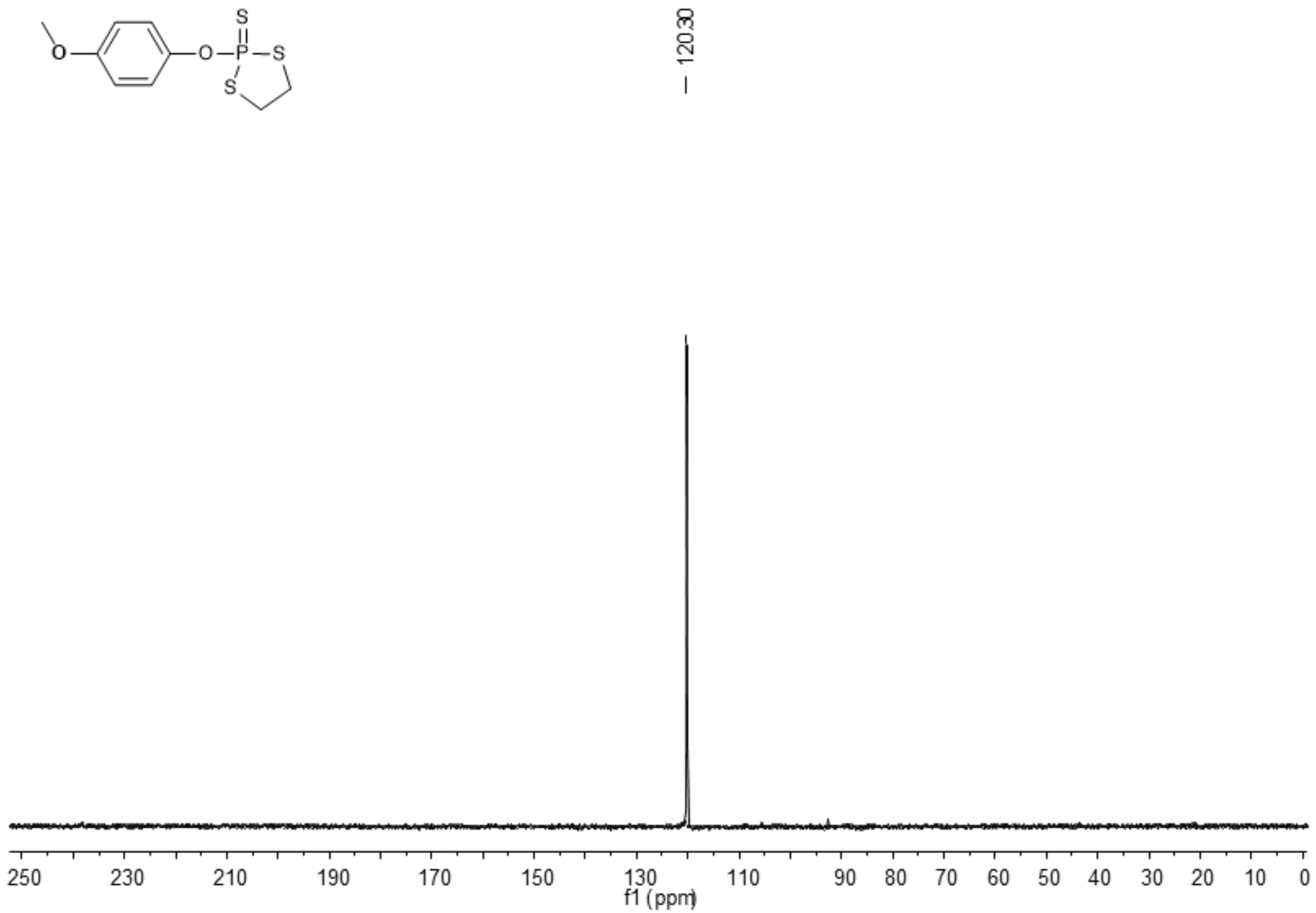

Figure A69. **^{31}P NMR (162 MHz, $CDCl_3$)** spectrum of **10b**.

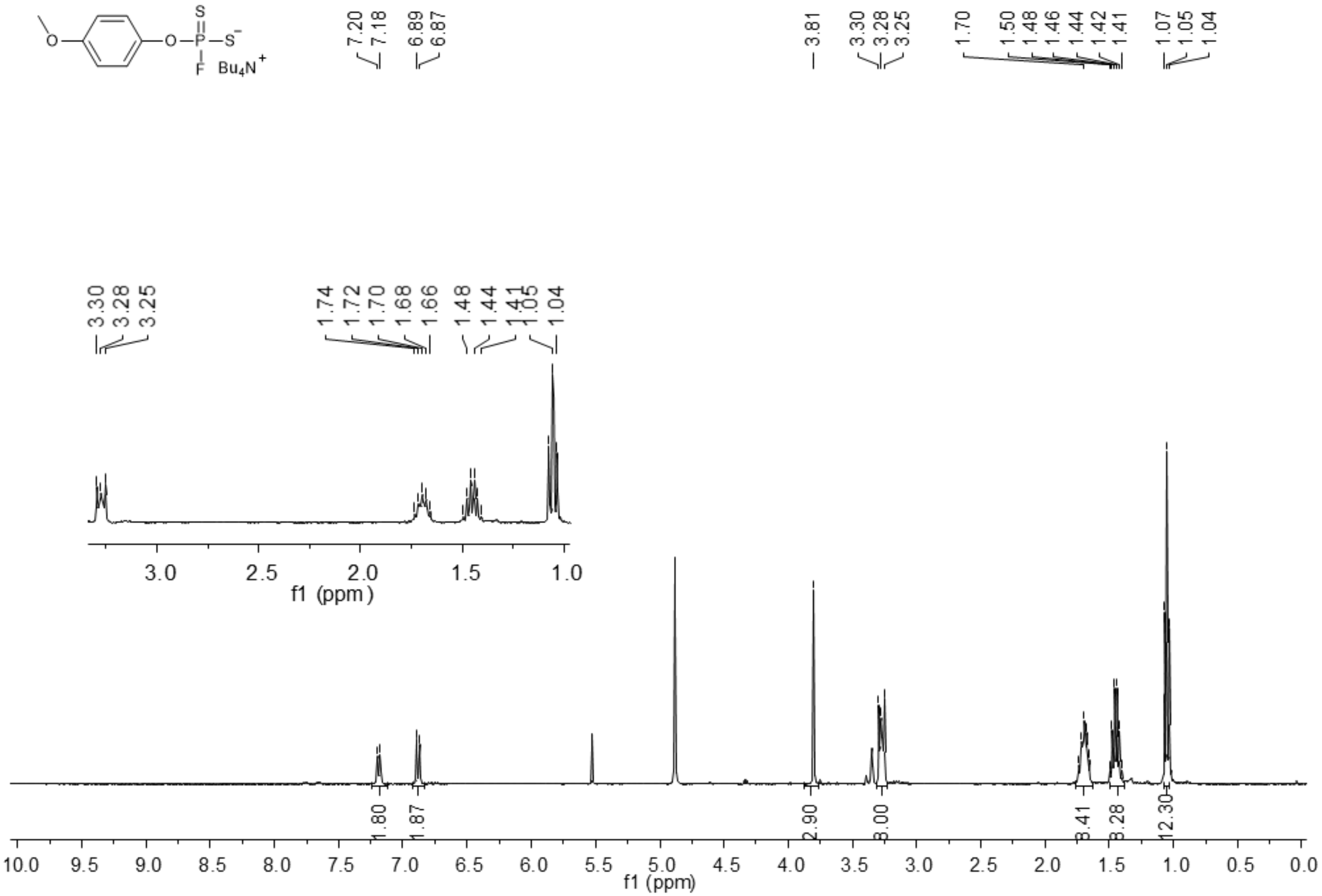

Figure A70. **^{1}H NMR (400 MHz, CD_3OD)** spectrum of **10**.

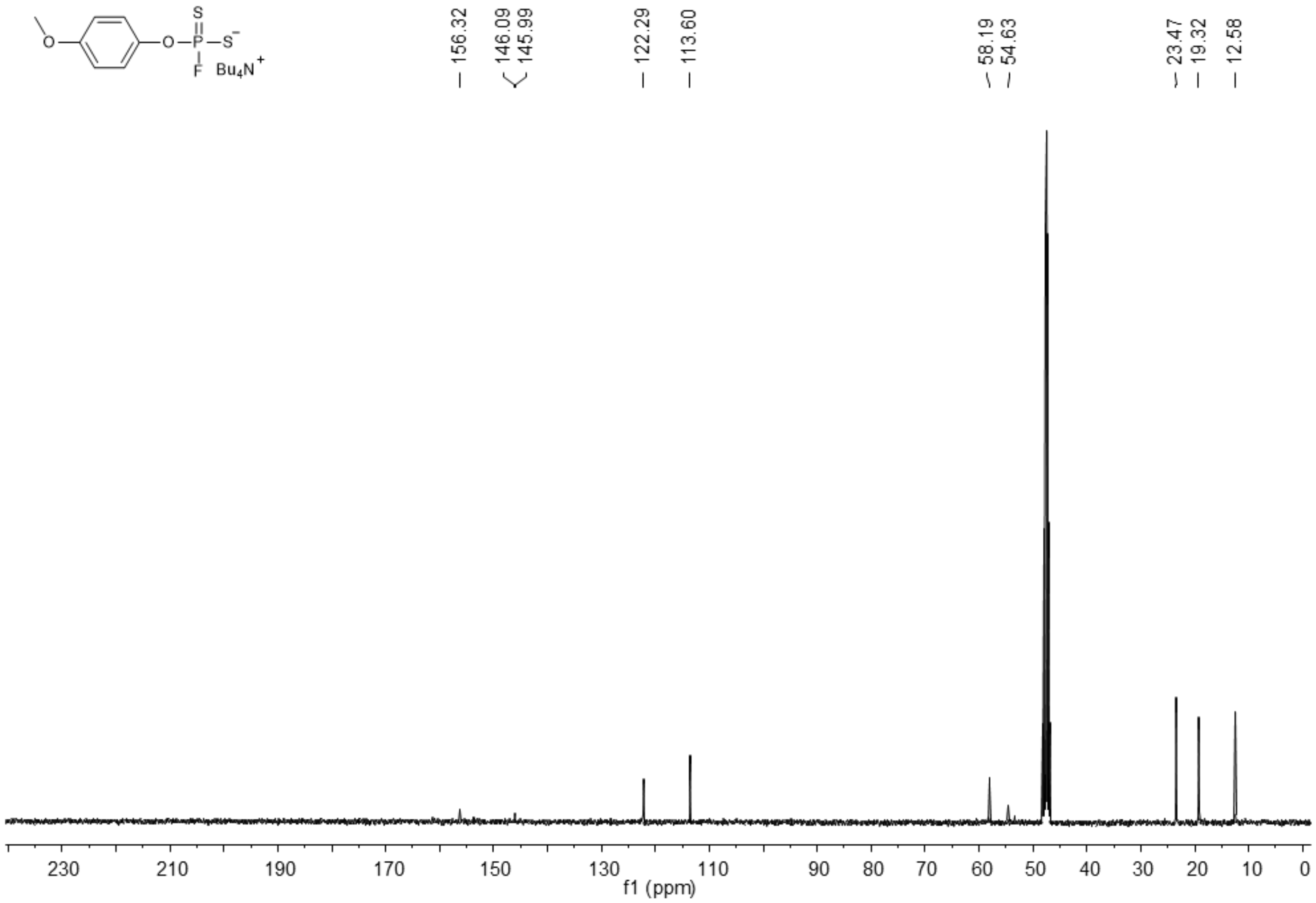

Figure A71. **^{13}C NMR (101 MHz, CD_3OD)** spectrum of **10**.

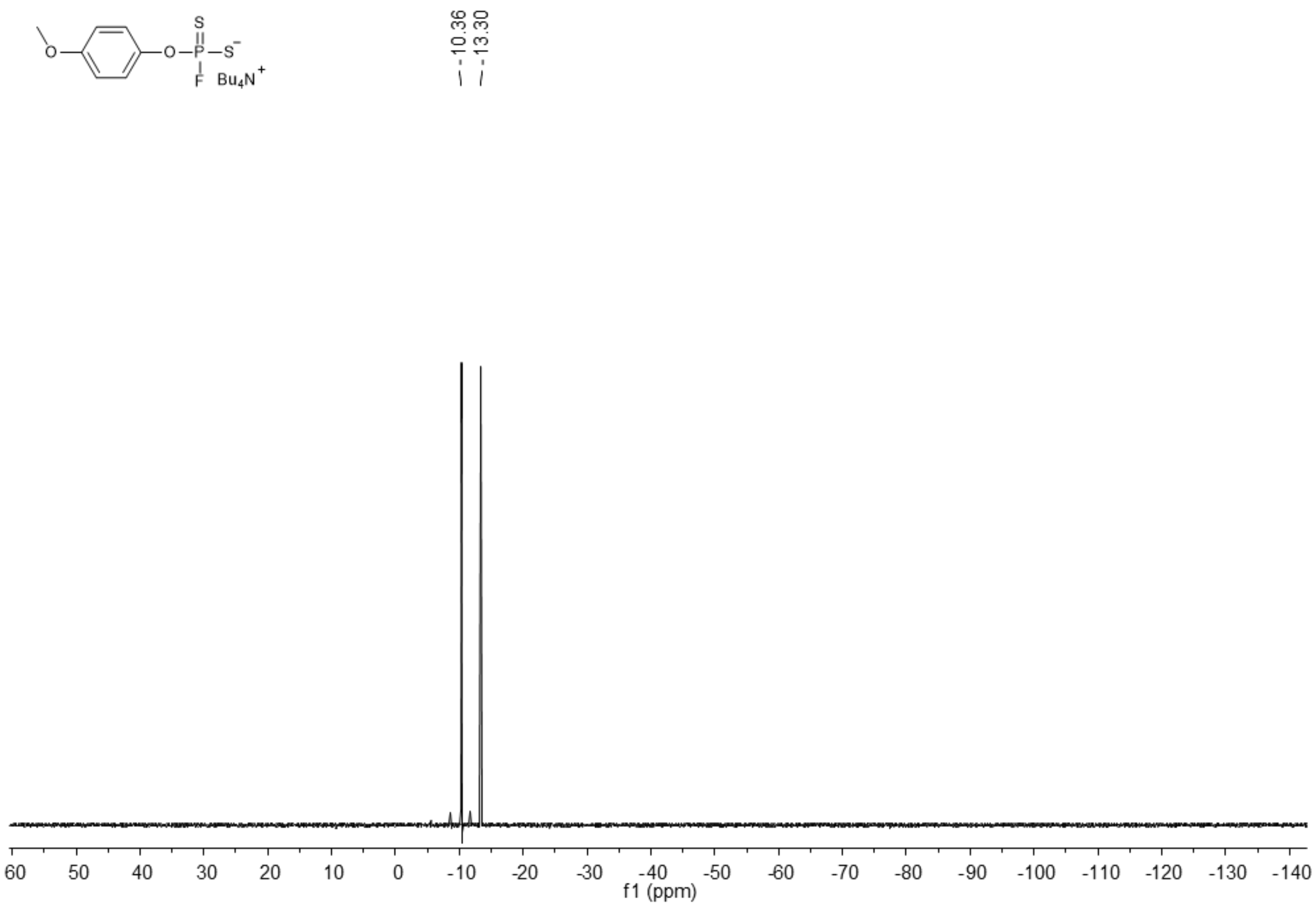

Figure A72. **^{19}F NMR (376 MHz, CD_3OD)** spectrum of **10**.

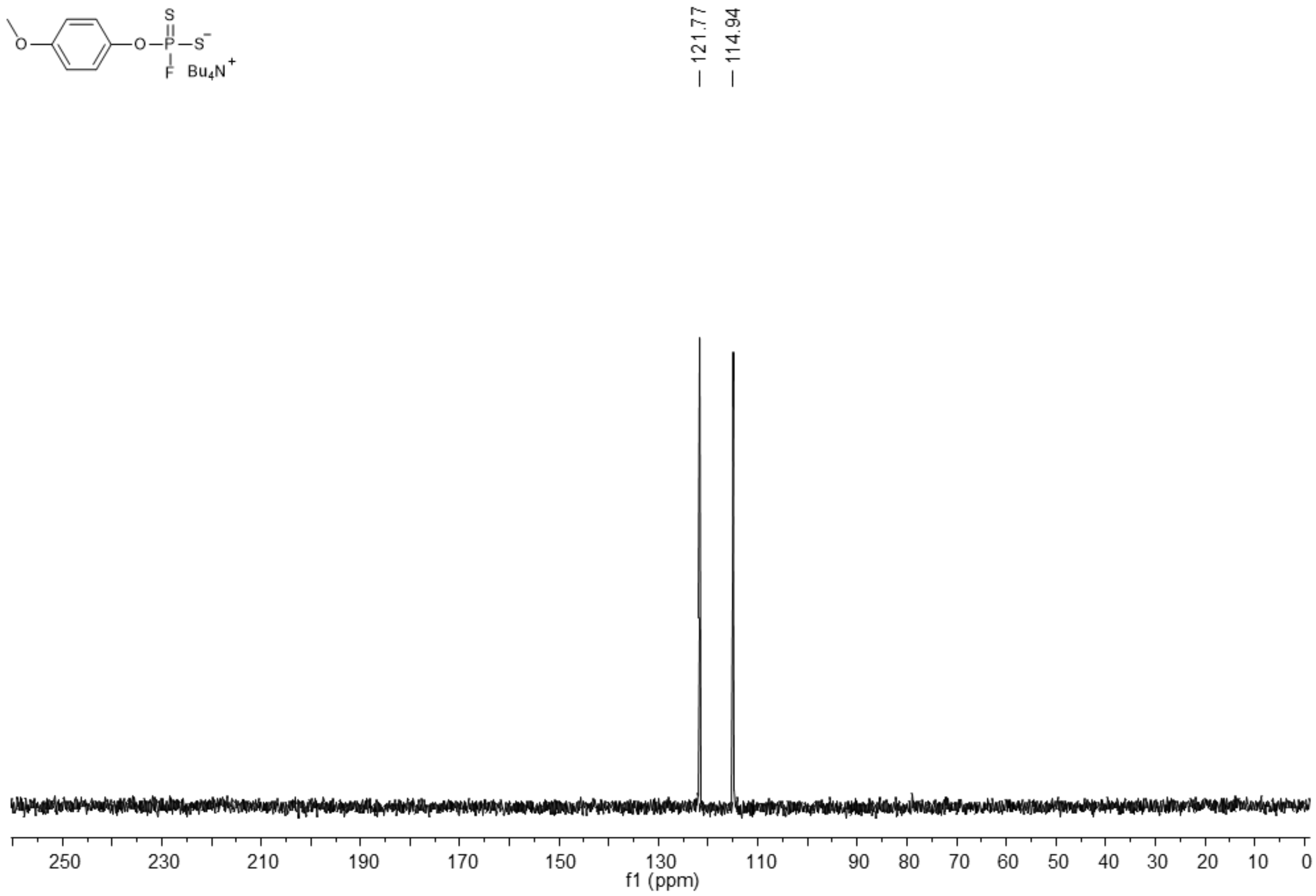

Figure A73. **^{31}P NMR (162 MHz, CD_3OD)** spectrum of **10**.

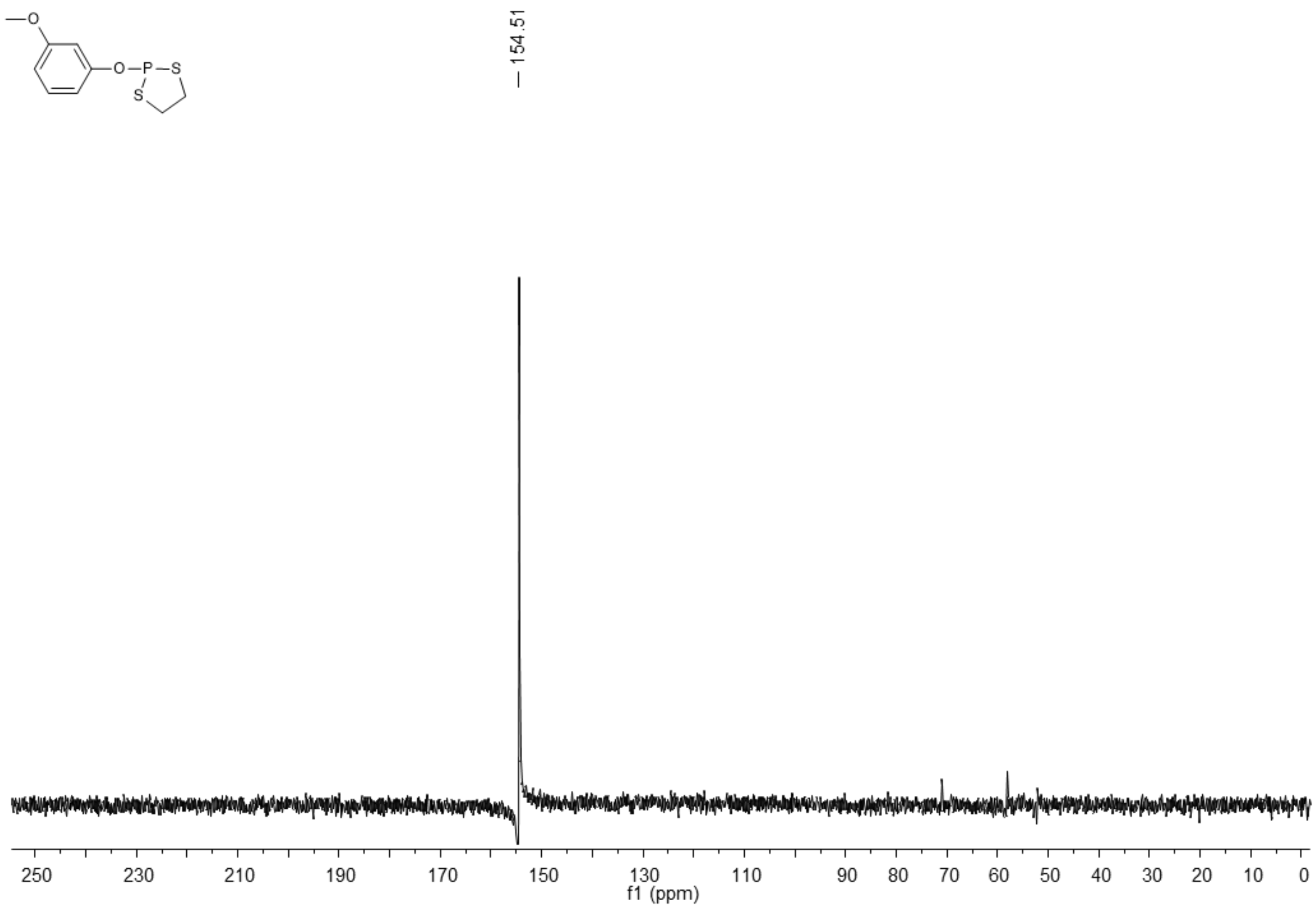

Figure A74. **^{31}P NMR (162 MHz, $CDCl_3$)** spectrum of **11a**.

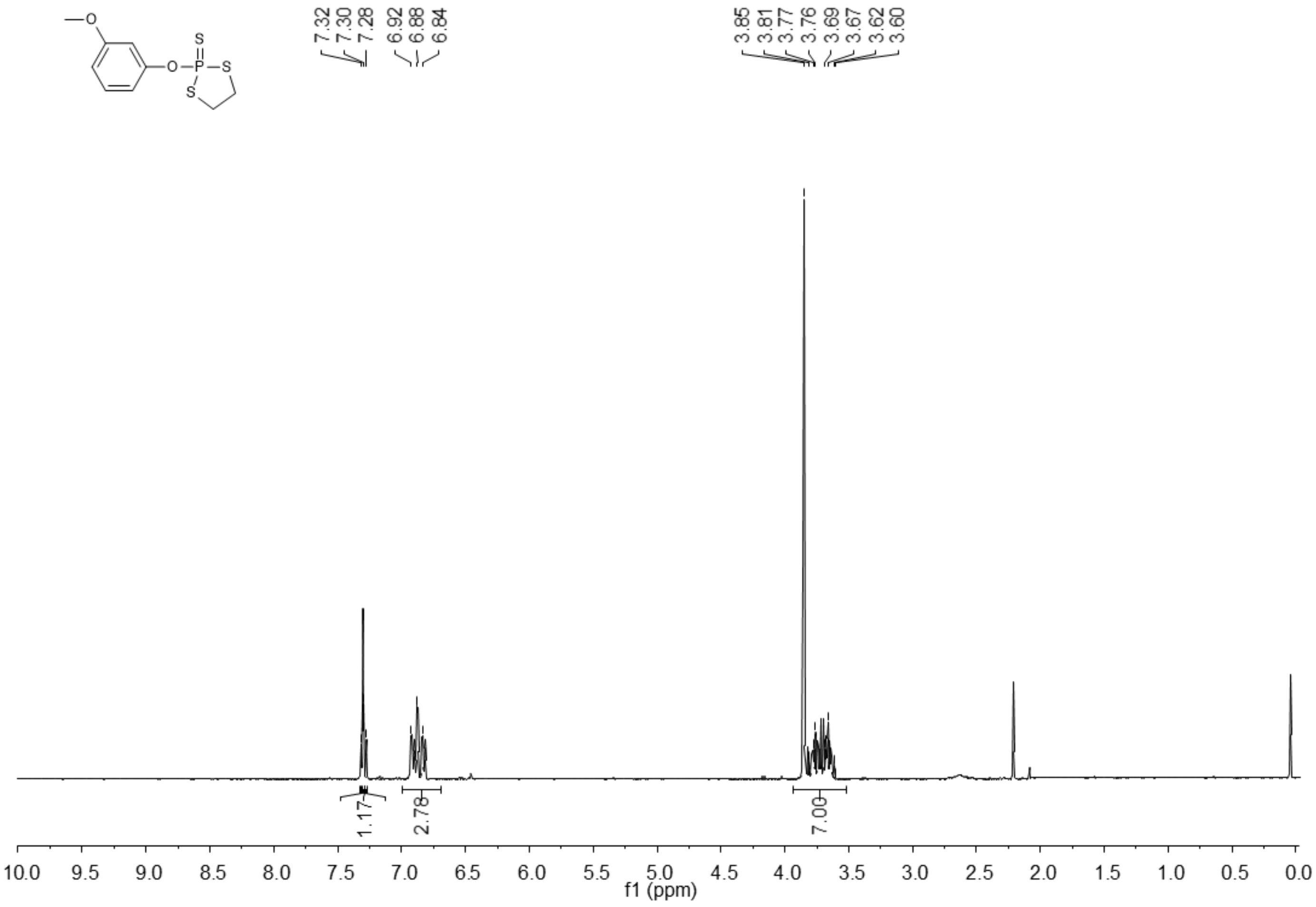

Figure A75. **^{1}H NMR (400 MHz, $CDCl_3$)** spectrum of **11b**.

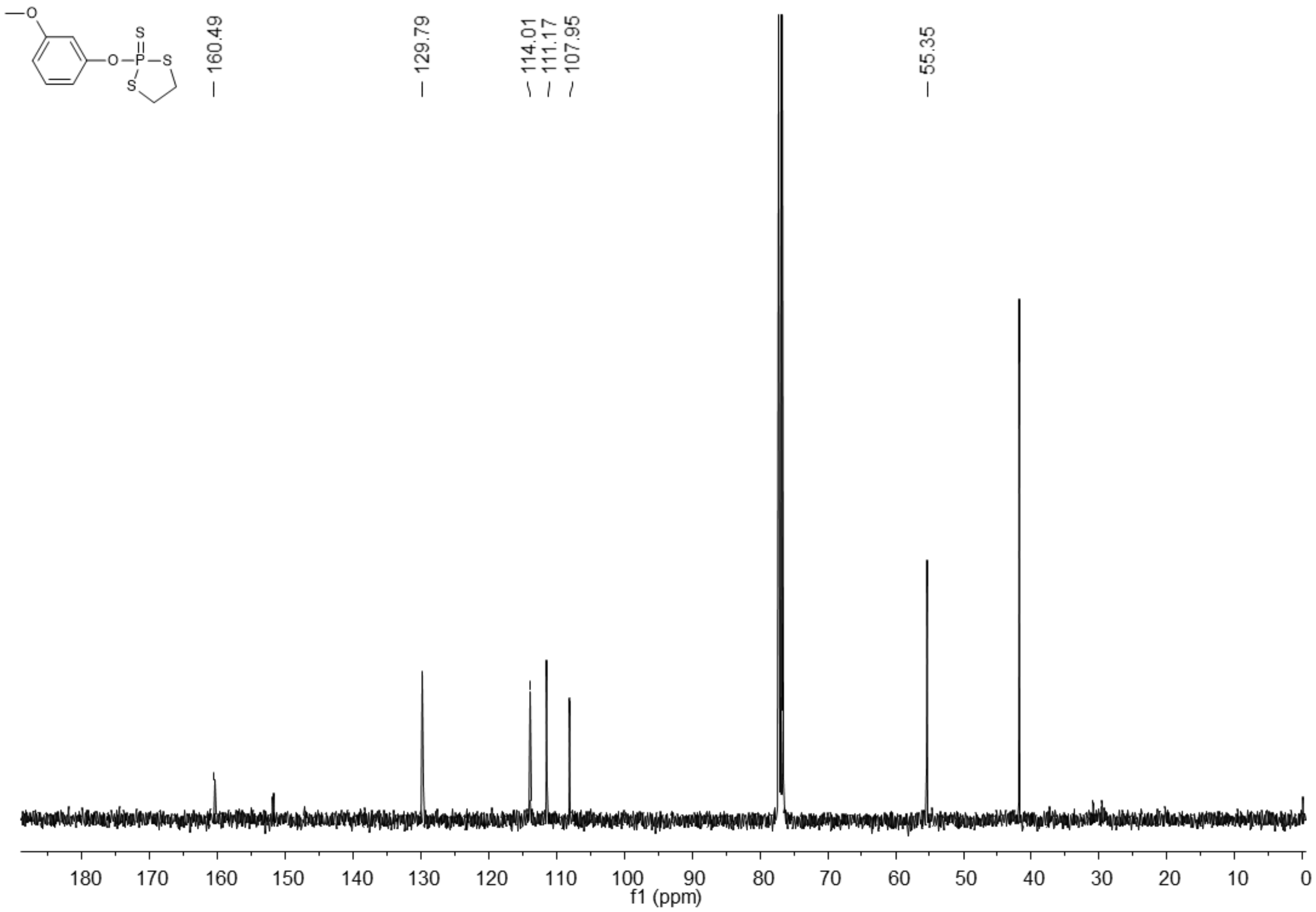

Figure A76. **^{13}C NMR (101 MHz, $CDCl_3$)** spectrum of **11b**.

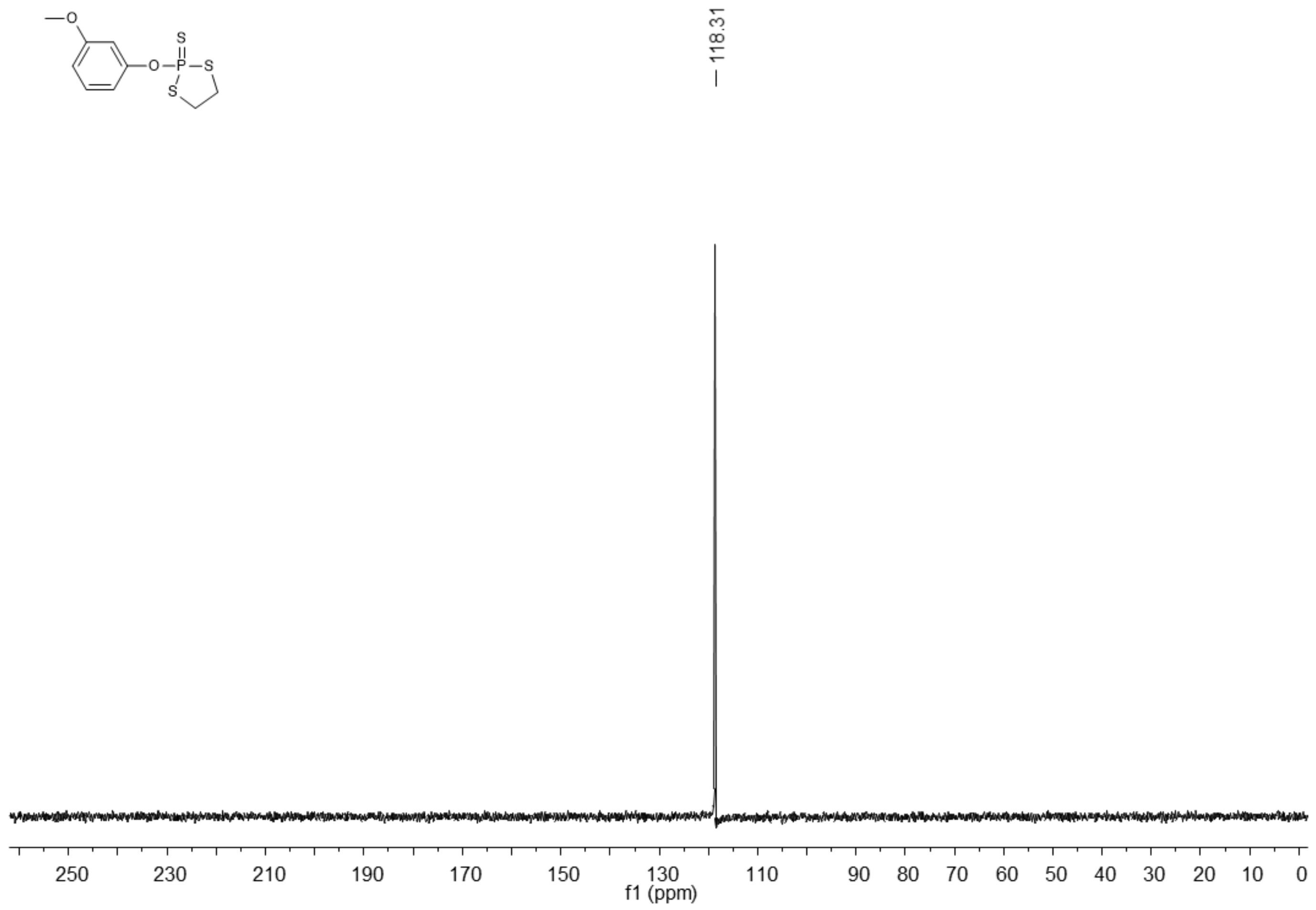

Figure A77. **^{31}P NMR (162 MHz, $CDCl_3$)** spectrum of **11b**.

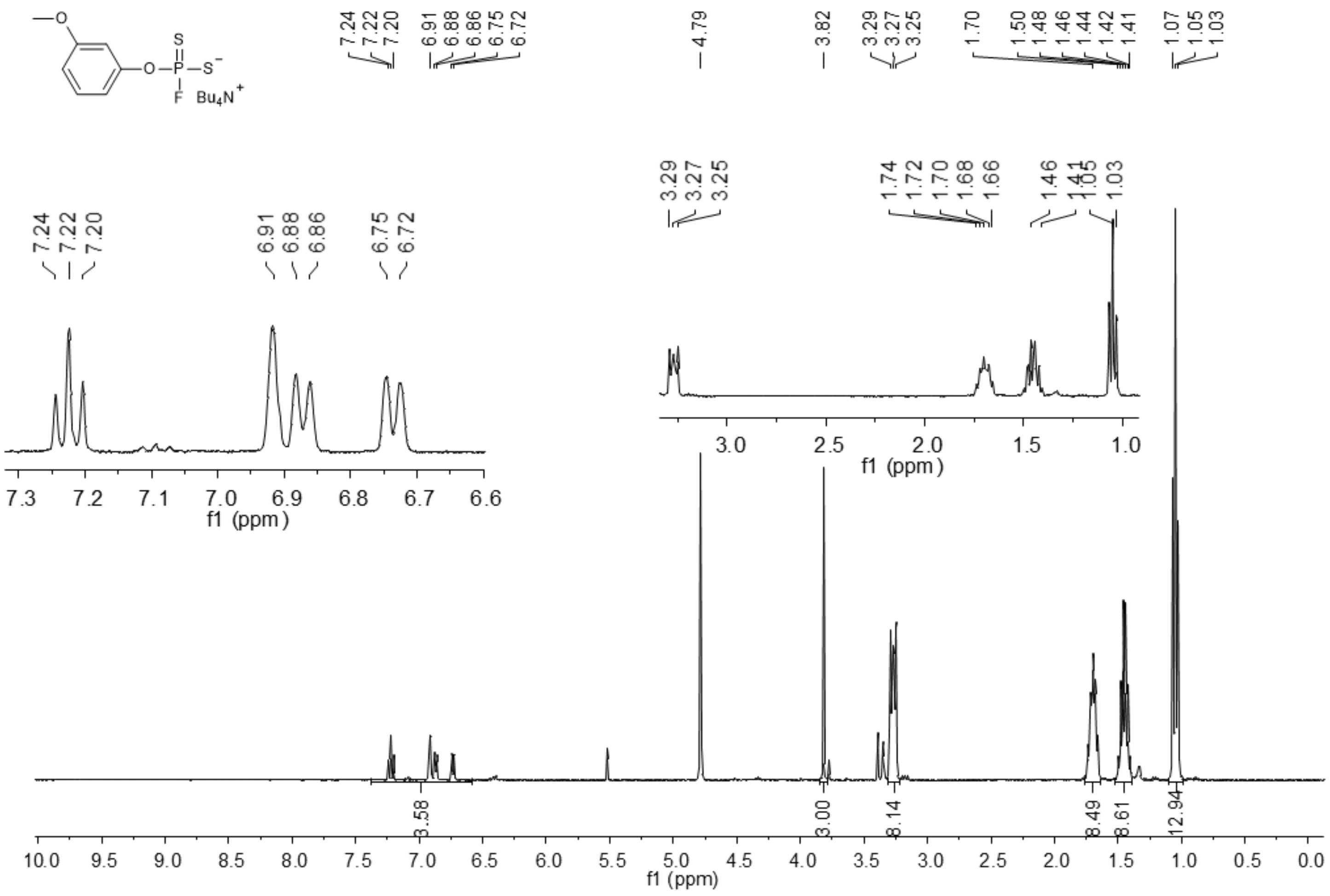

Figure A78. **^{1}H NMR (400 MHz, CD_3OD)** spectrum of **11**.

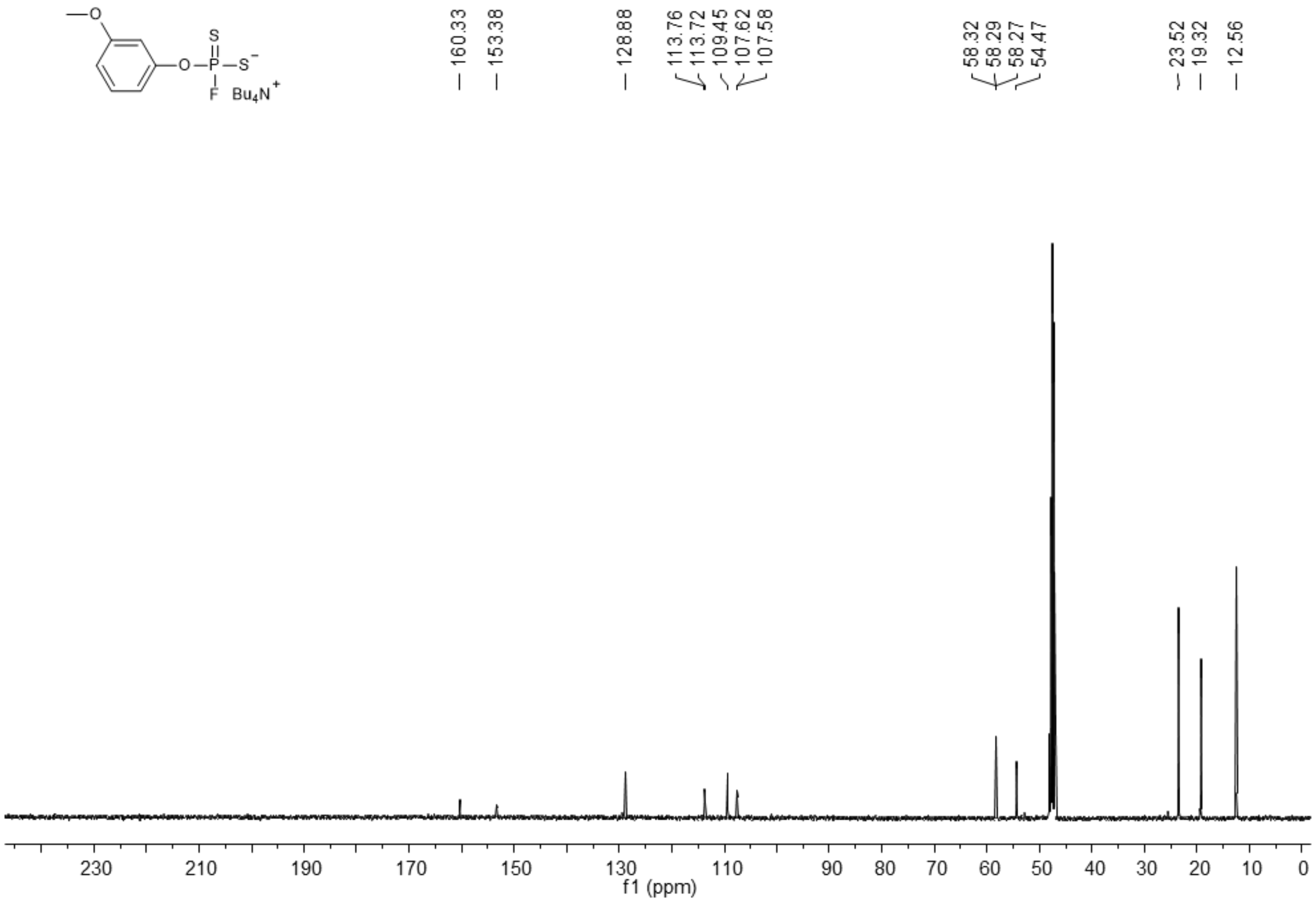

Figure A79. ^{13}**C NMR (101 MHz, CD$_3$OD)** spectrum of **11**.

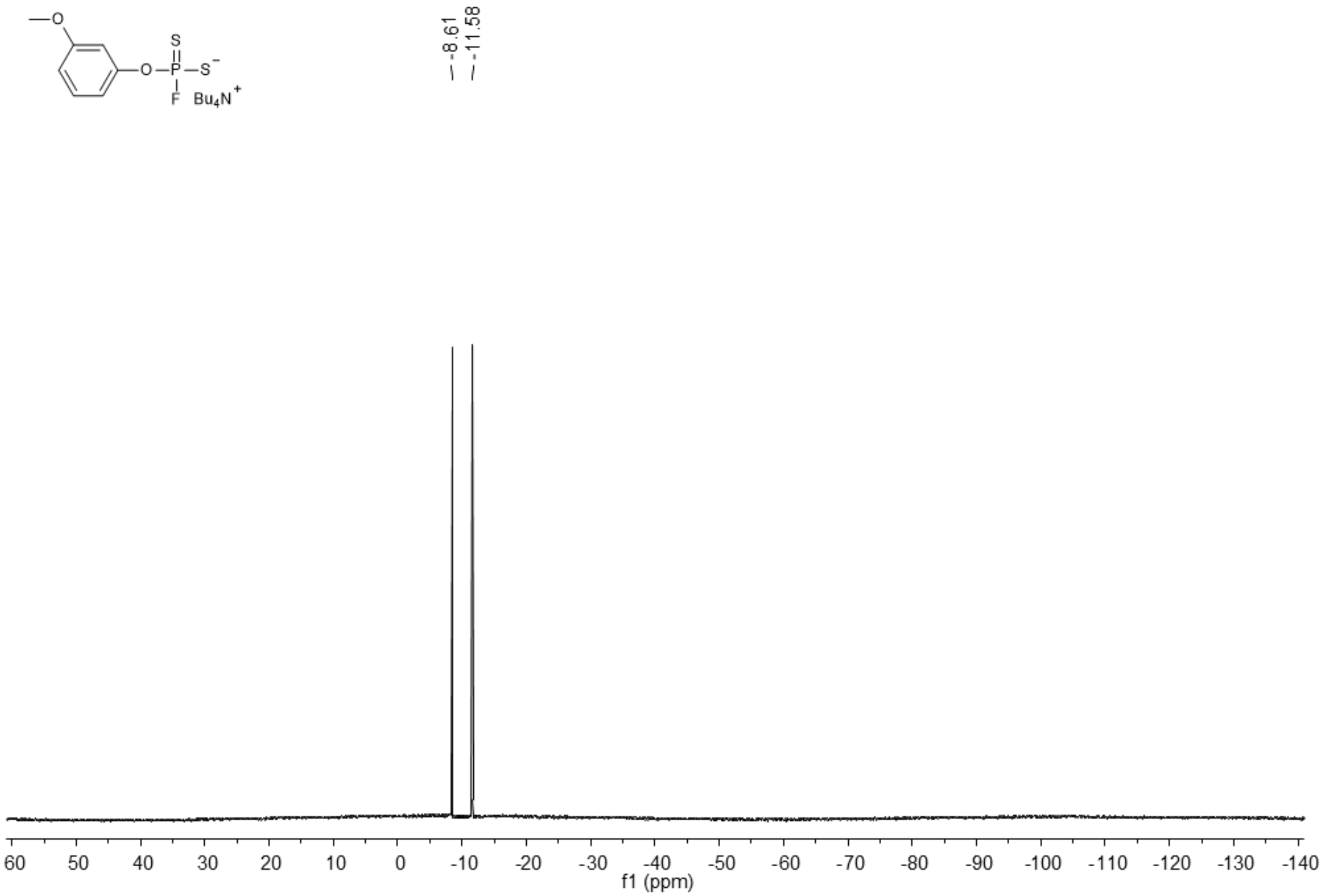

Figure A80. **^{19}F NMR (376 MHz, CD_3OD)** spectrum of **11**.

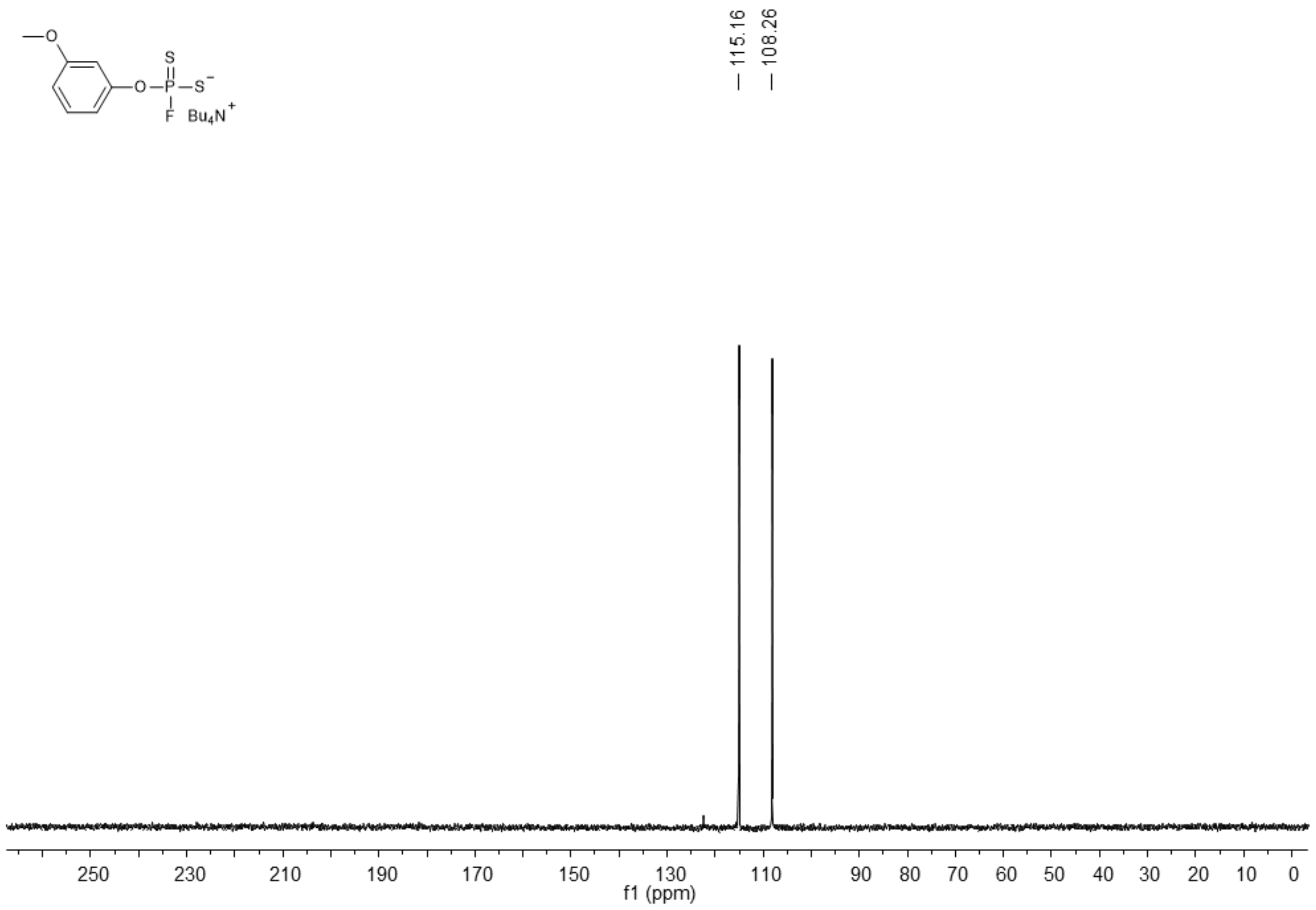

Figure A81. **^{31}P NMR (162 MHz, CD_3OD)** spectrum of **11**.

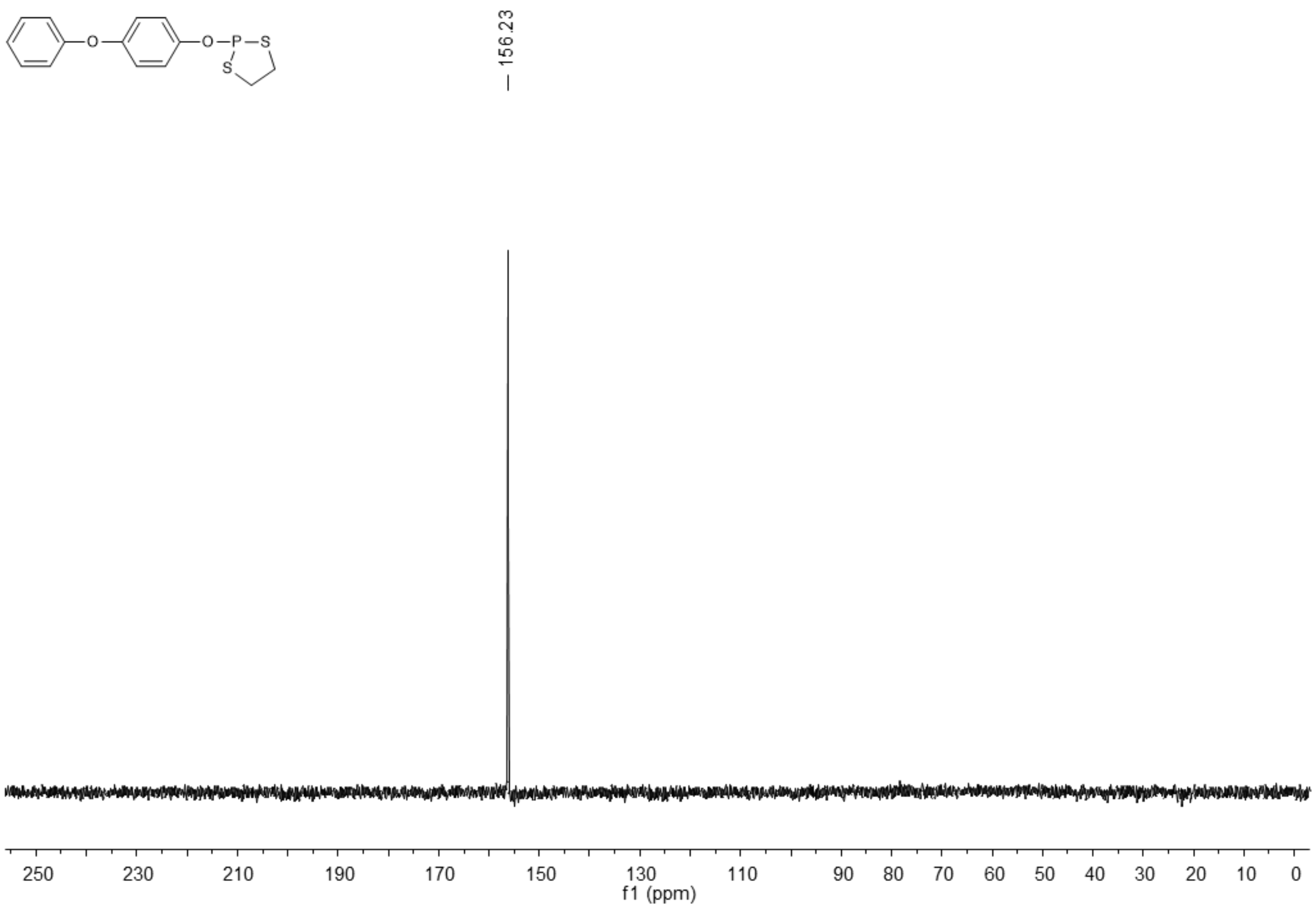

Figure A82. ^{31}**P NMR (162 MHz, CDCl$_3$)** spectrum of **12a**.

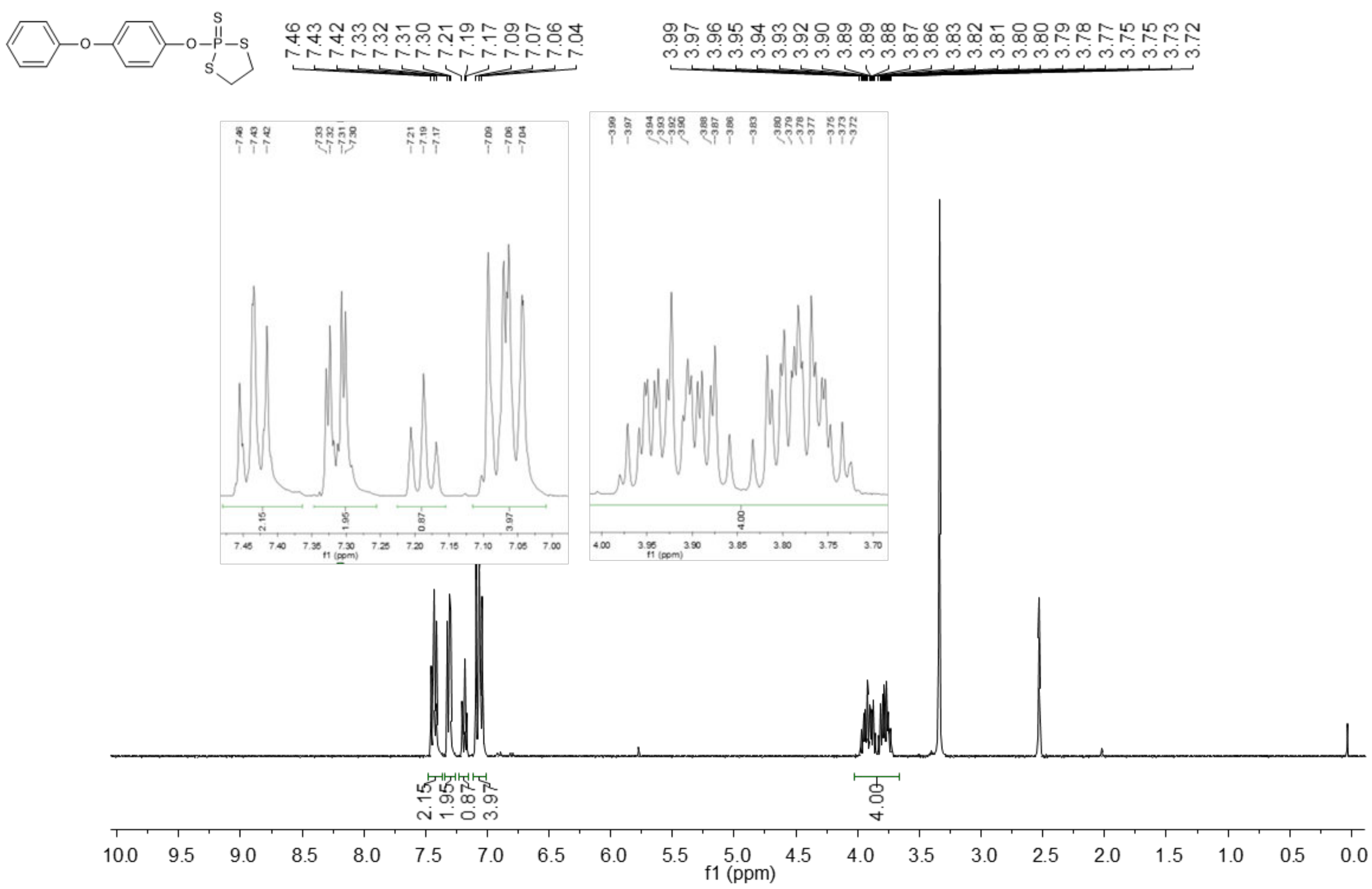

Figure A83. **^{1}H NMR (400 MHz, DMSO-d_6)** spectrum of **12b**.

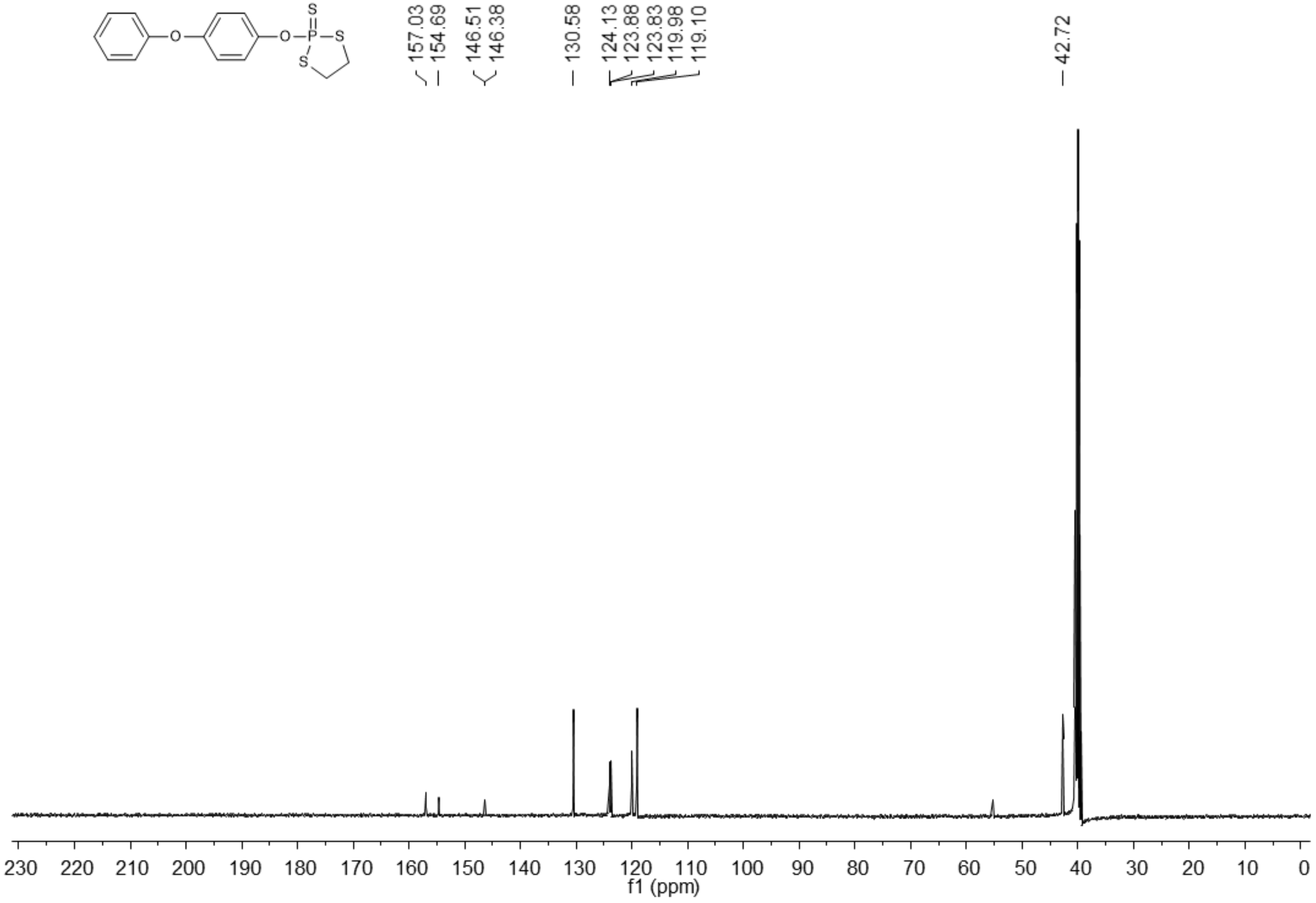

Figure A84. **^{13}C NMR (101 MHz, DMSO-*d₆*)** spectrum of **12b**.

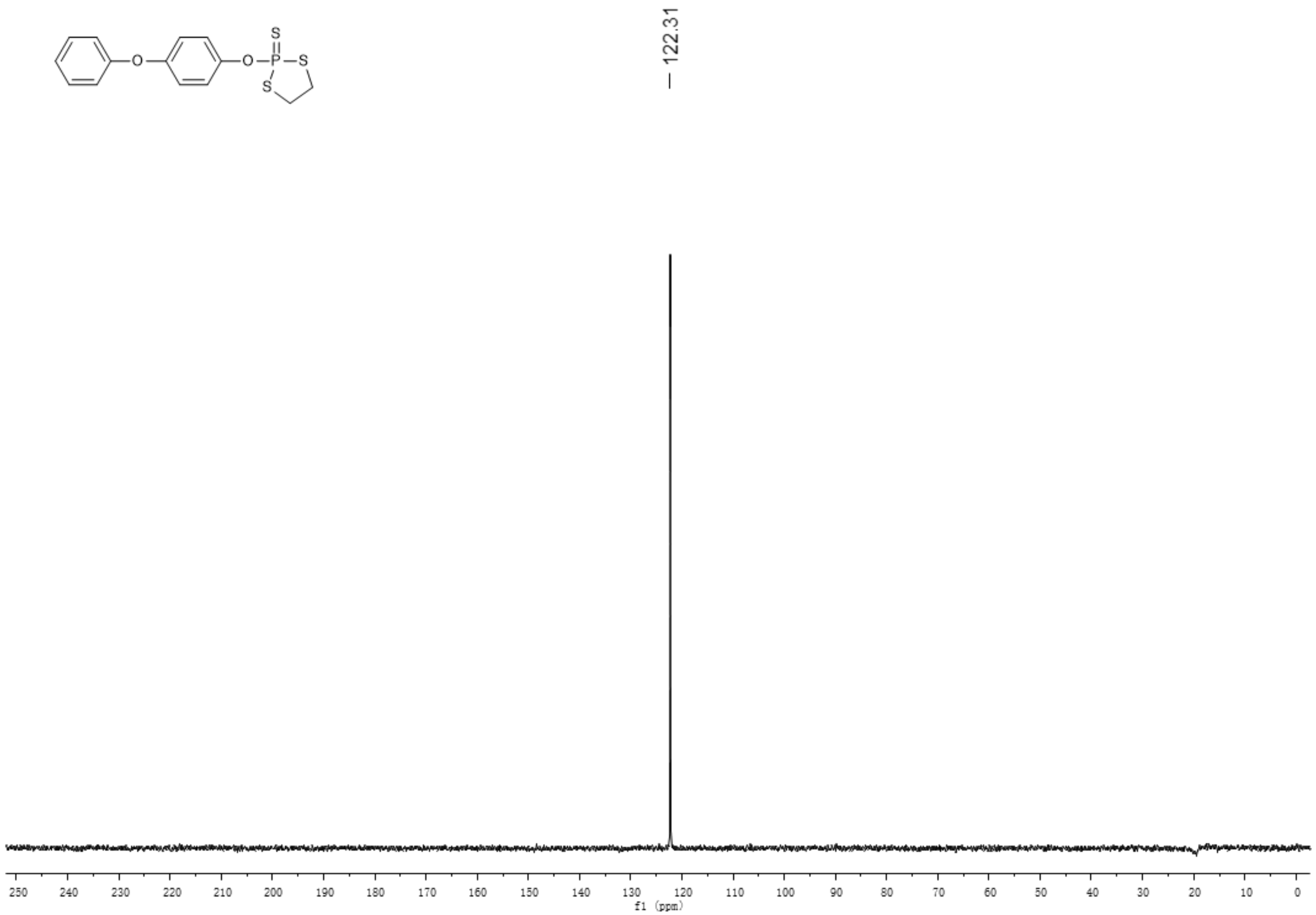

Figure A85. **^{31}P NMR (162 MHz, DMSO-*d_6*)** spectrum of **12b**.

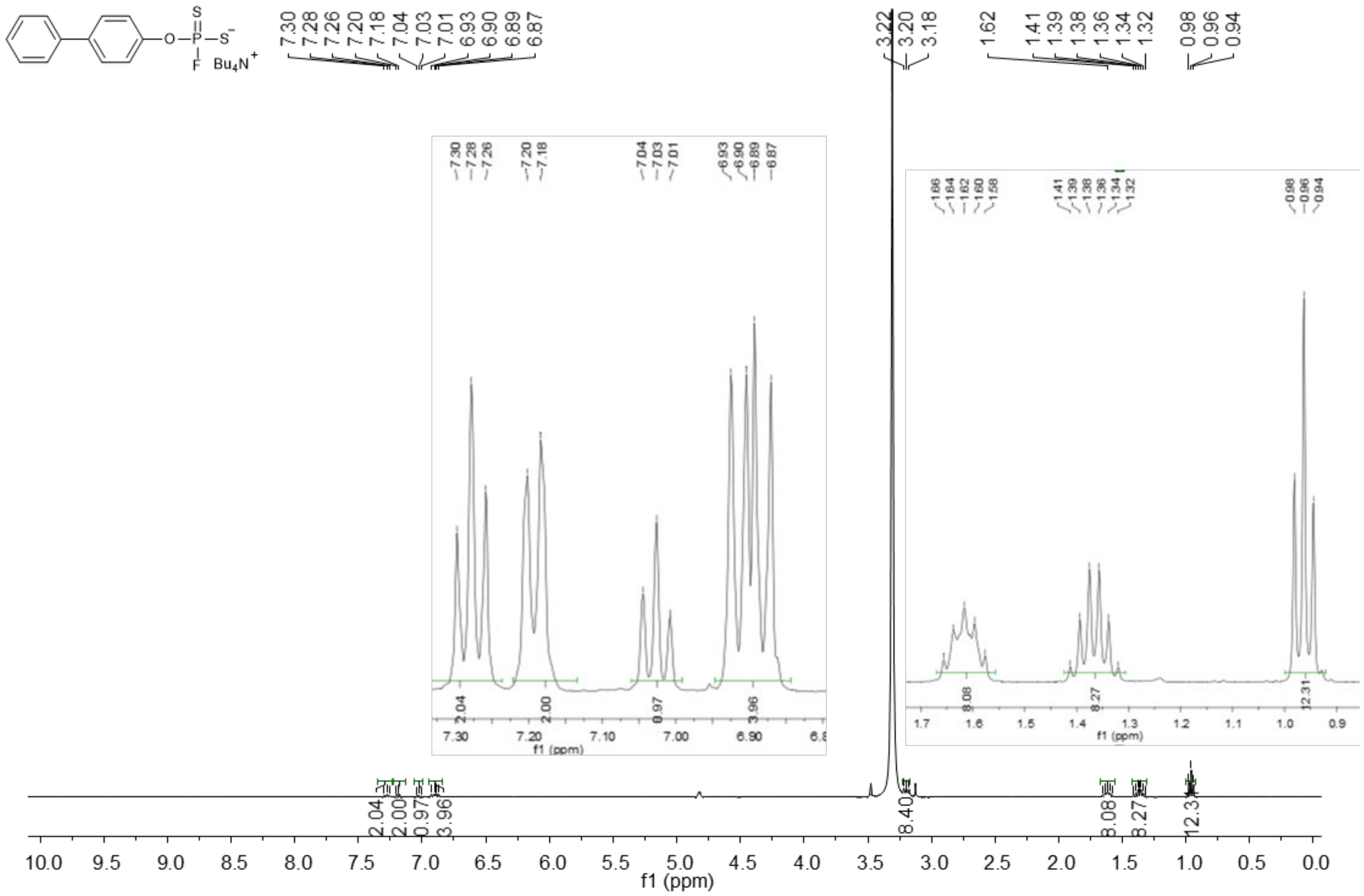

Figure A86. **^{1}H NMR (400 MHz, CD_3OD)** spectrum of **12**.

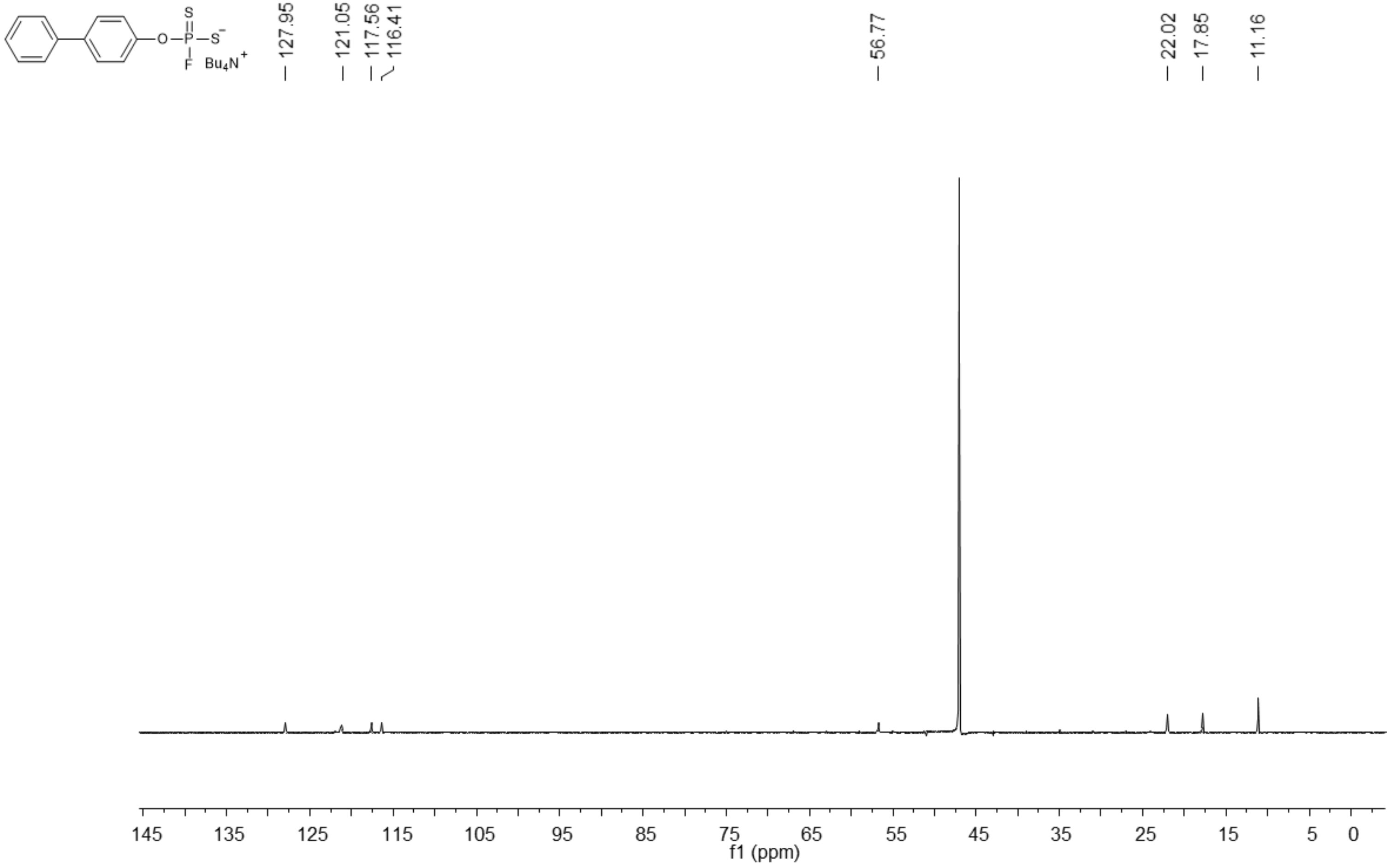

Figure A87. **^{13}C NMR (101 MHz, CD_3OD)** spectrum of **12**.

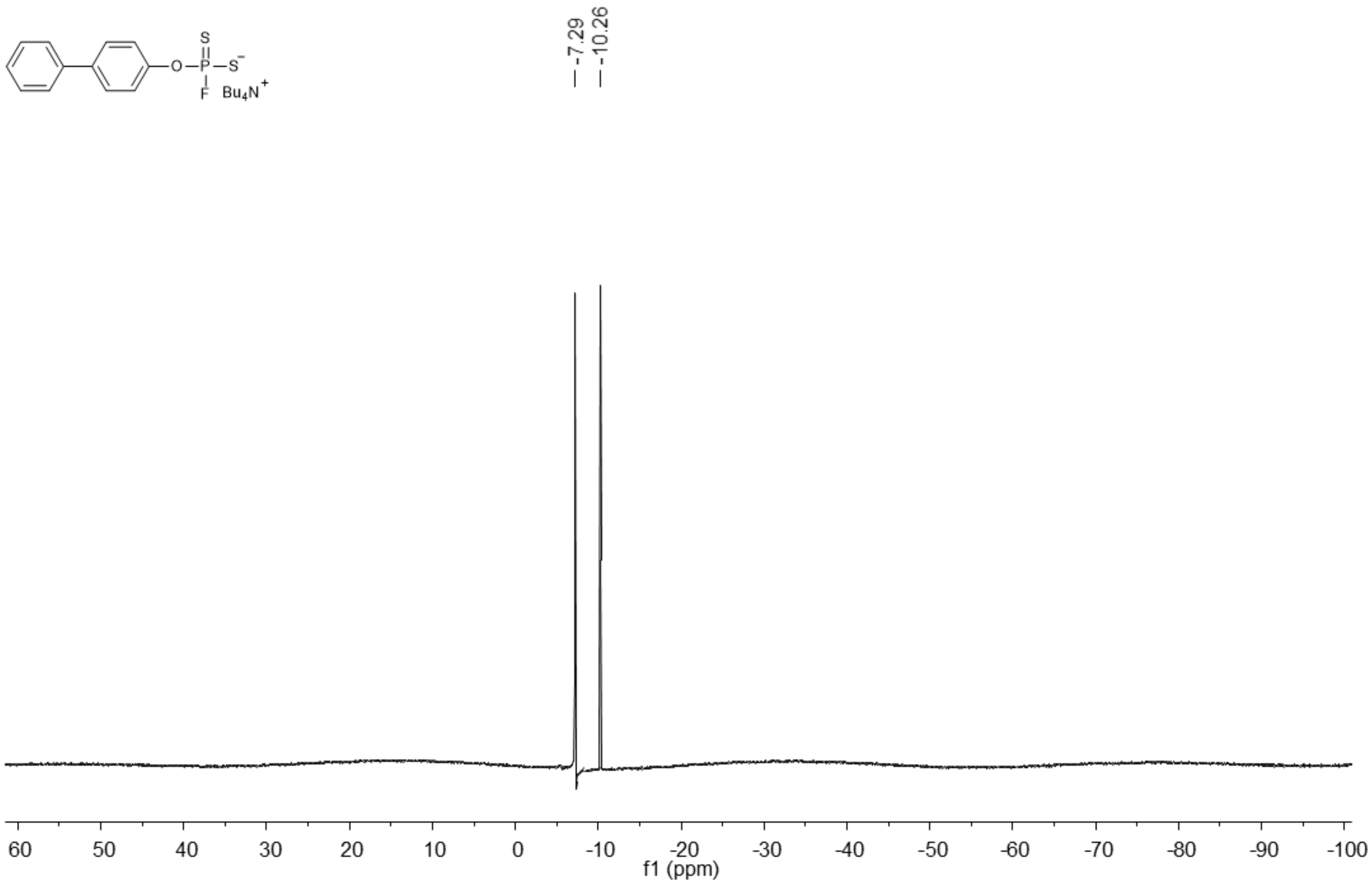

Figure A88. **^{19}F NMR (376 MHz, CD_3OD)** spectrum of **12**.

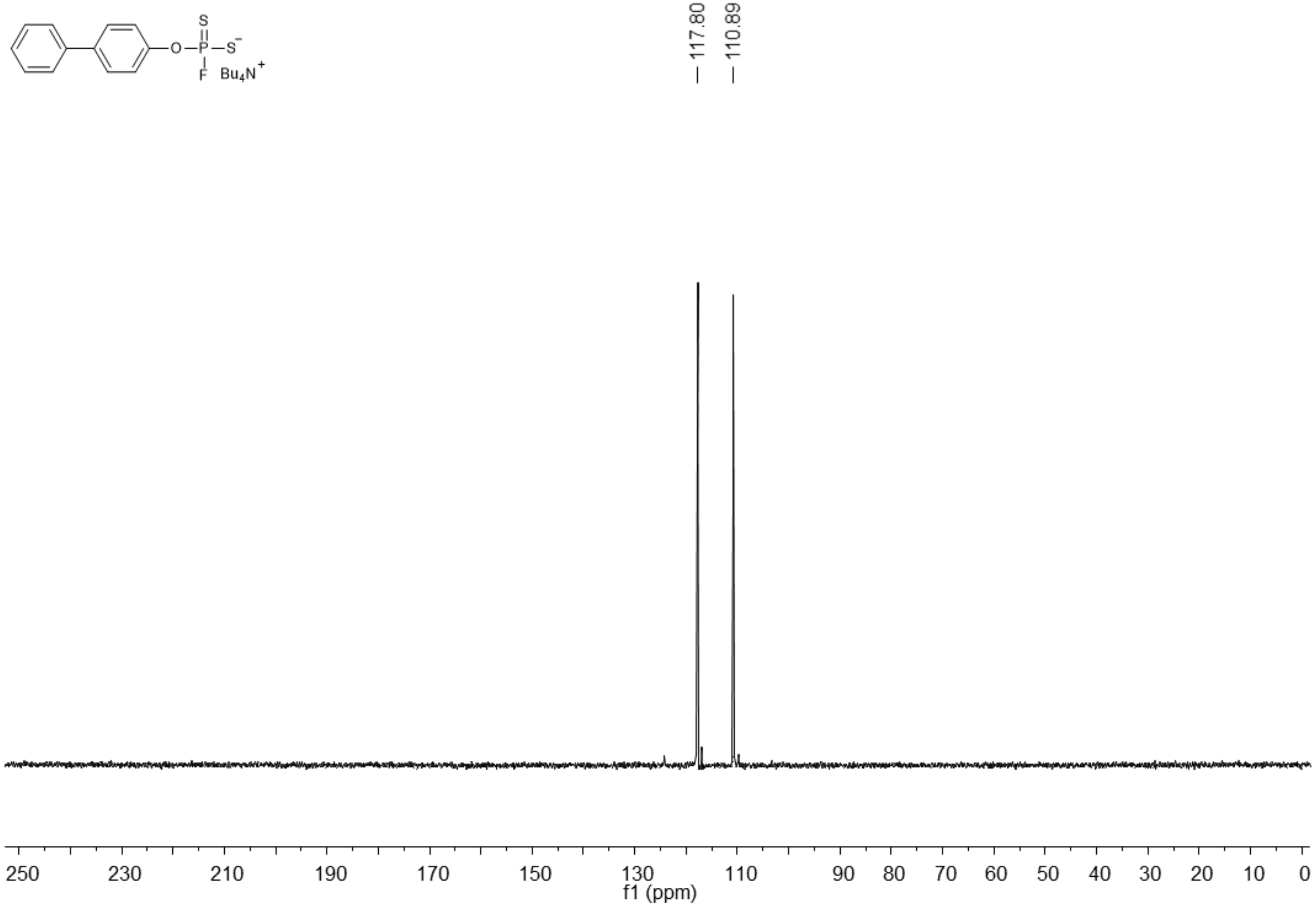

Figure A89. **^{31}P NMR (162 MHz, CD_3OD)** spectrum of **12**.

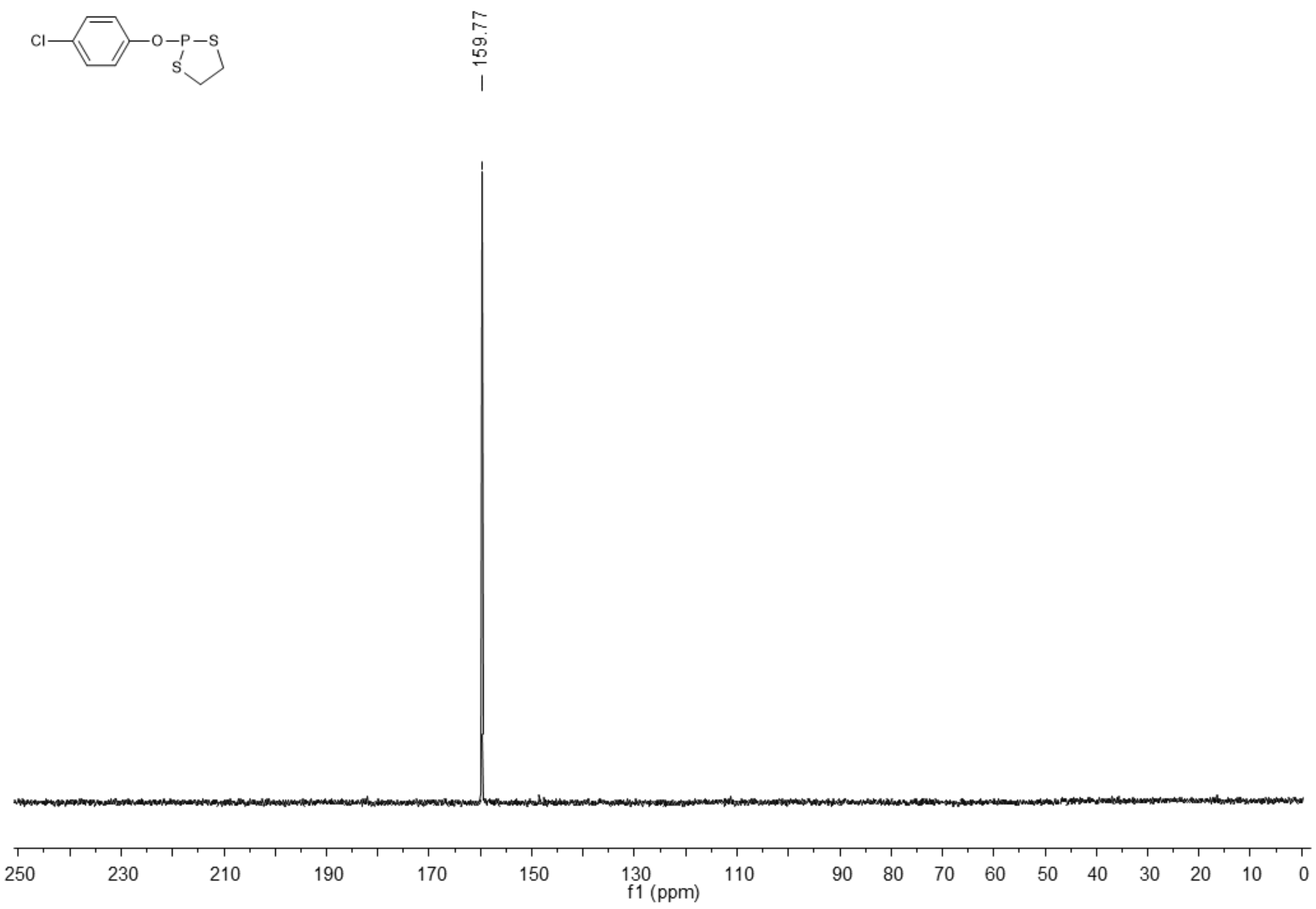

Figure A90. **^{31}P NMR (162 MHz, $CDCl_3$)** spectrum of **13a**.

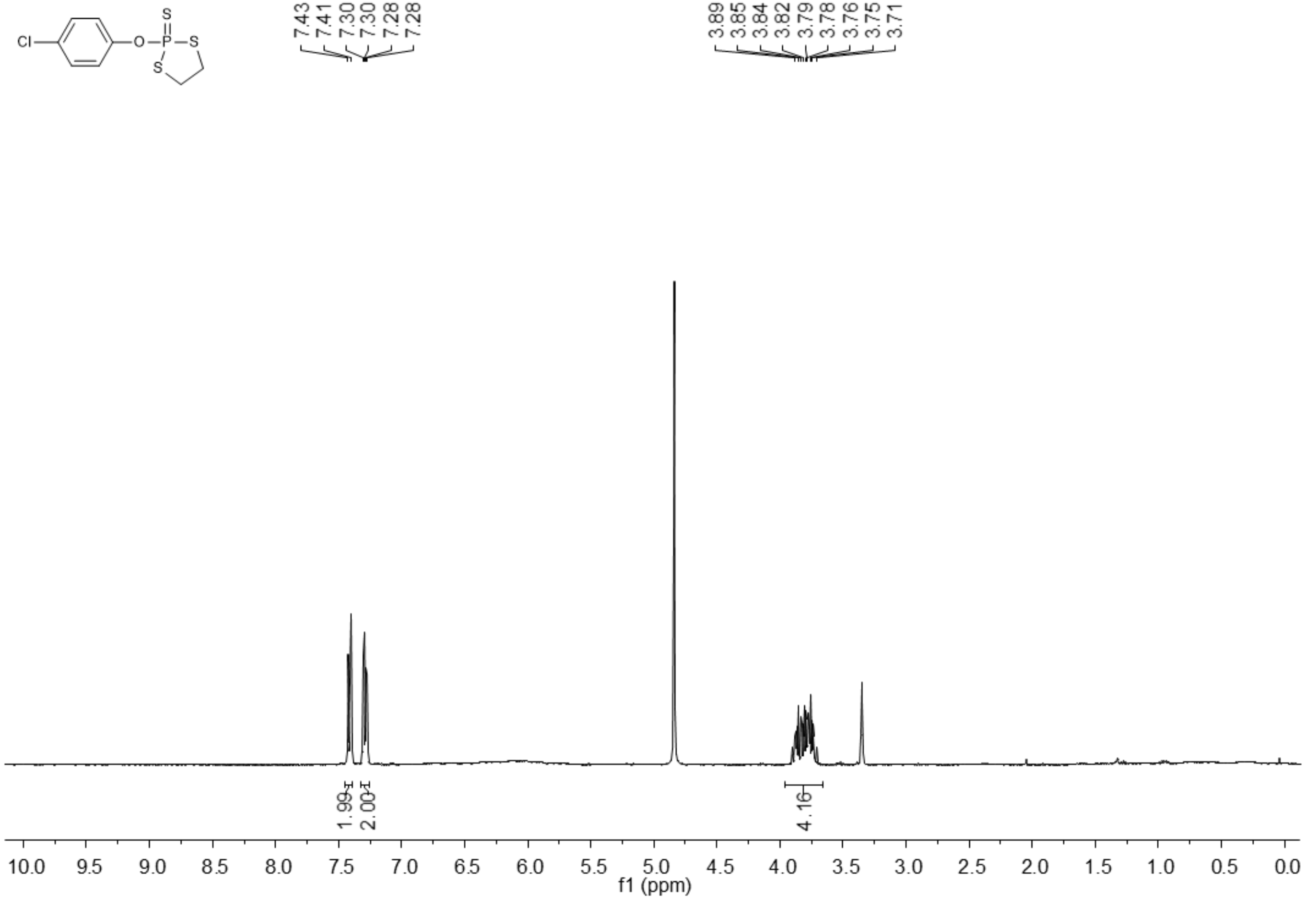

Figure A91. **^{1}H NMR (400 MHz, CD_3OD)** spectrum of **13b**.

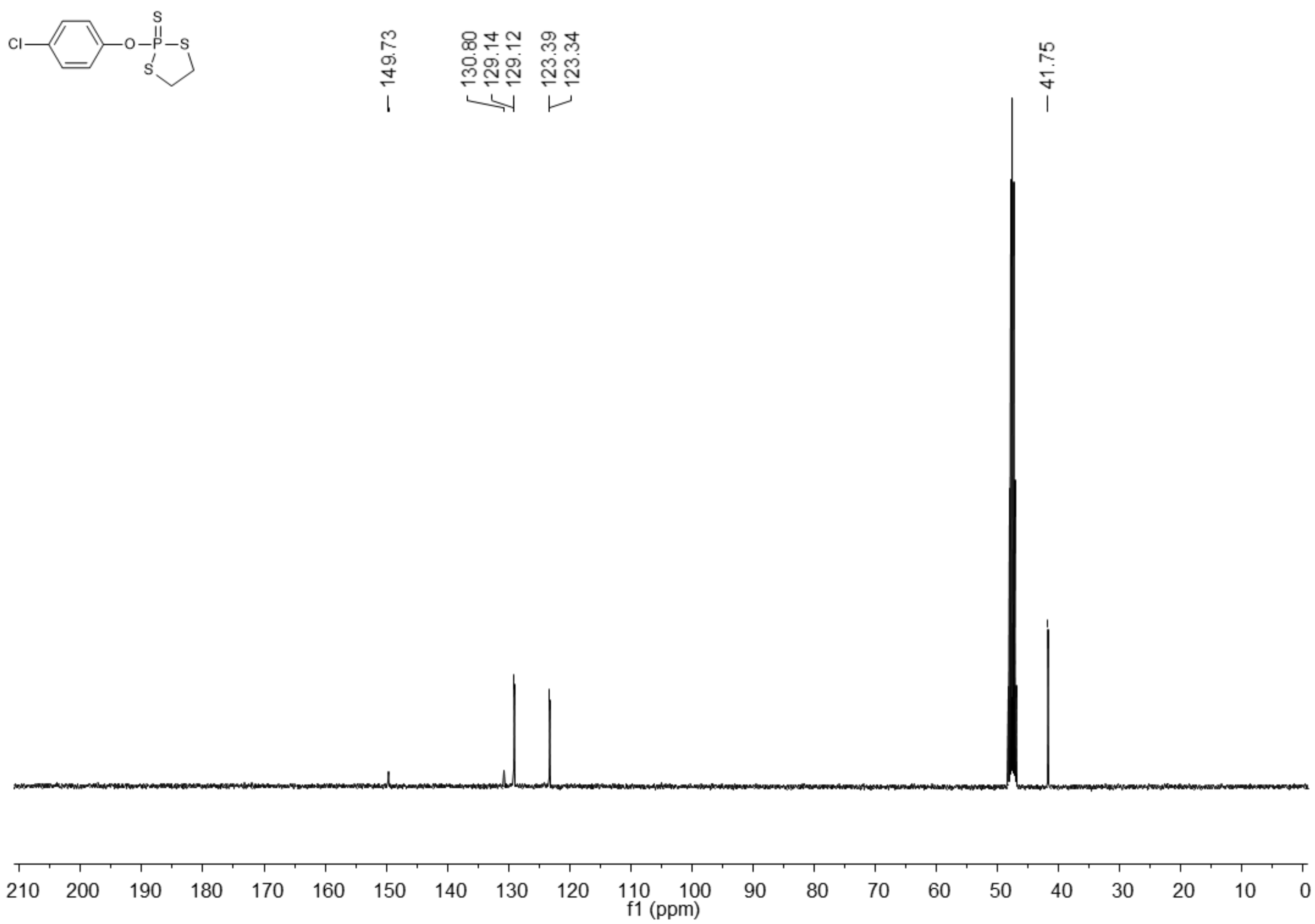

Figure A92. ^{13}**C NMR (101 MHz, CD$_3$OD)** spectrum of **13b**.

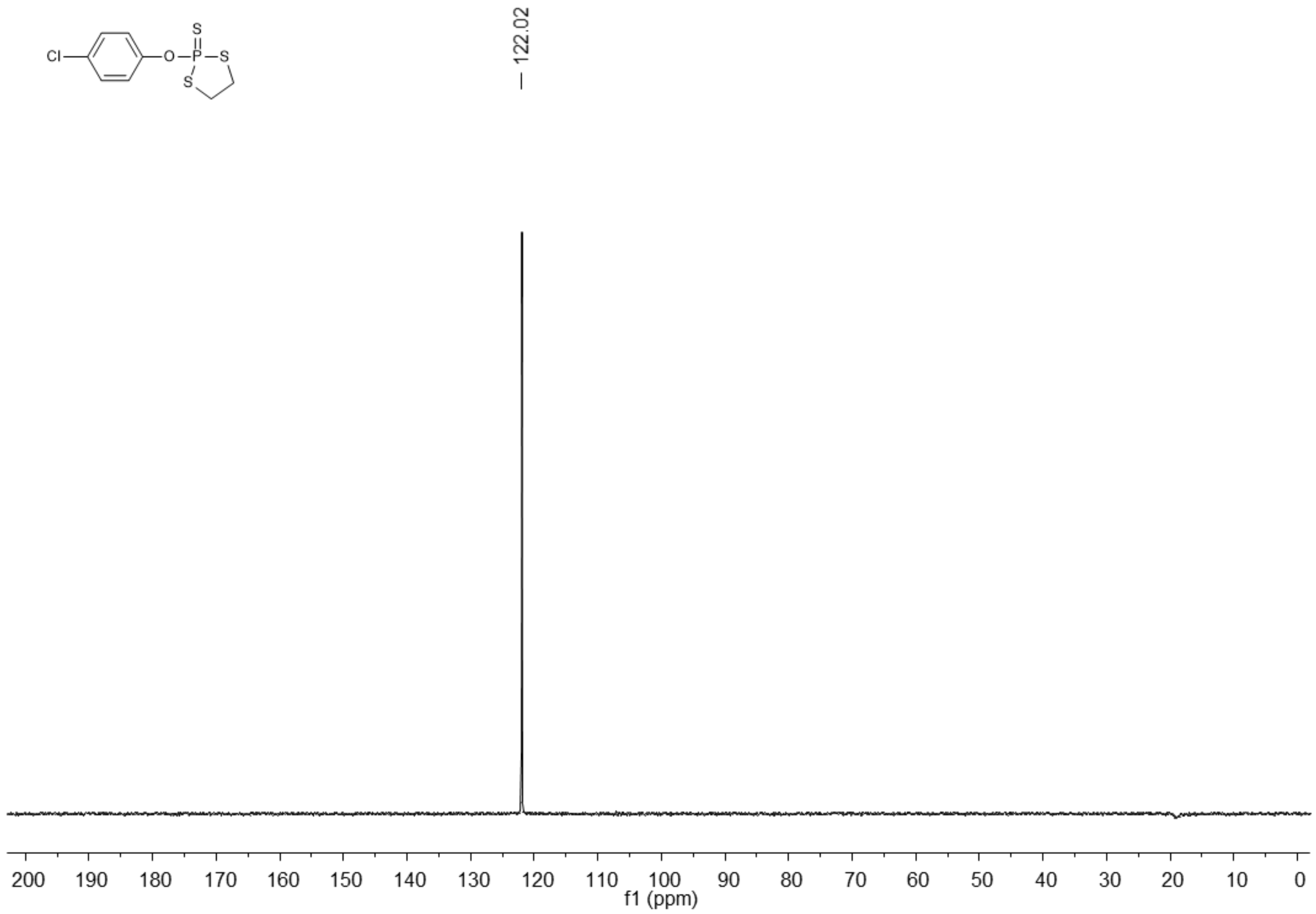

Figure A93. **^{31}P NMR (162 MHz, CD_3OD)** spectrum of **13b**.

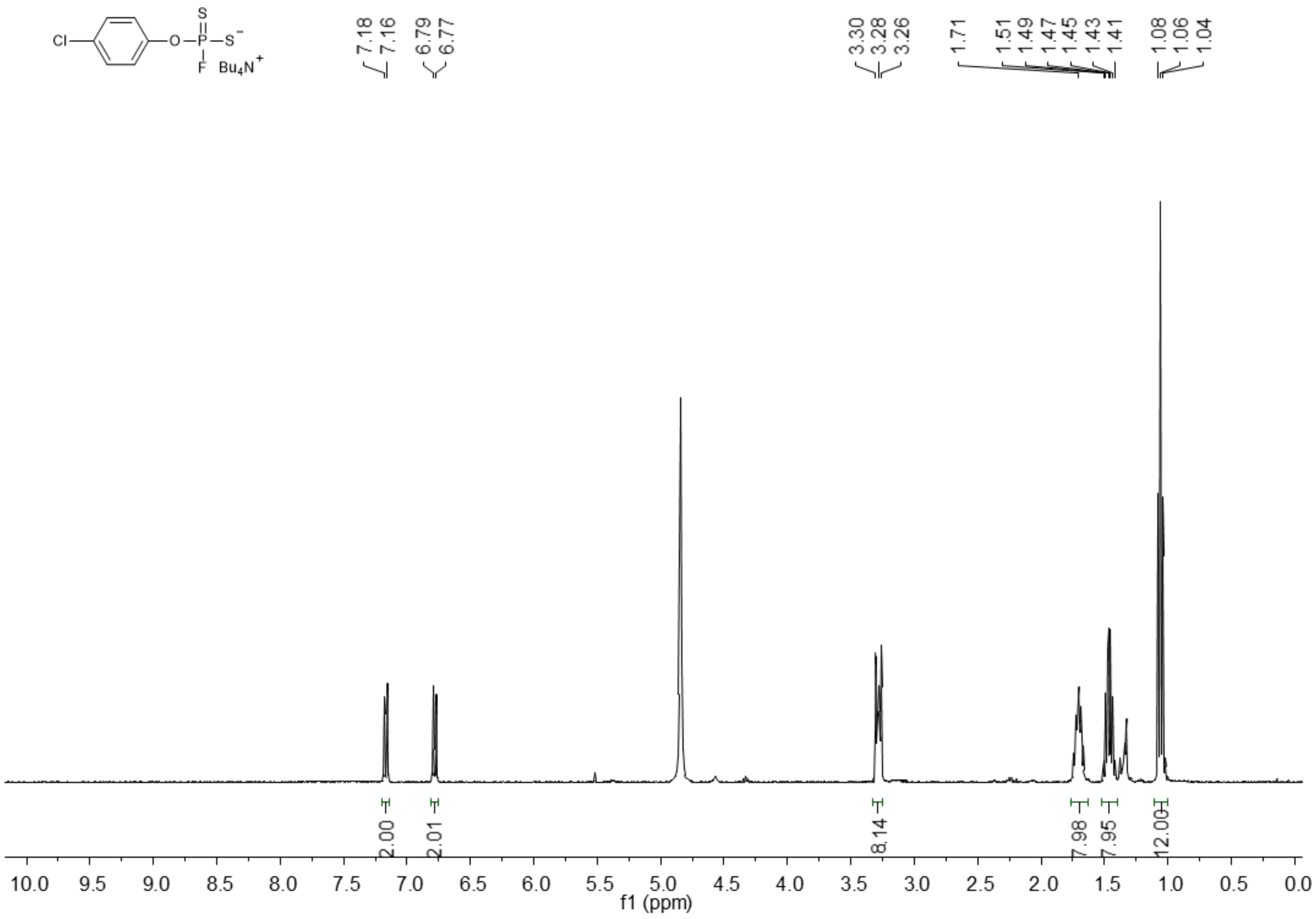

Figure A94. **^{1}H NMR (400 MHz, CD_3OD)** spectrum of **13**.

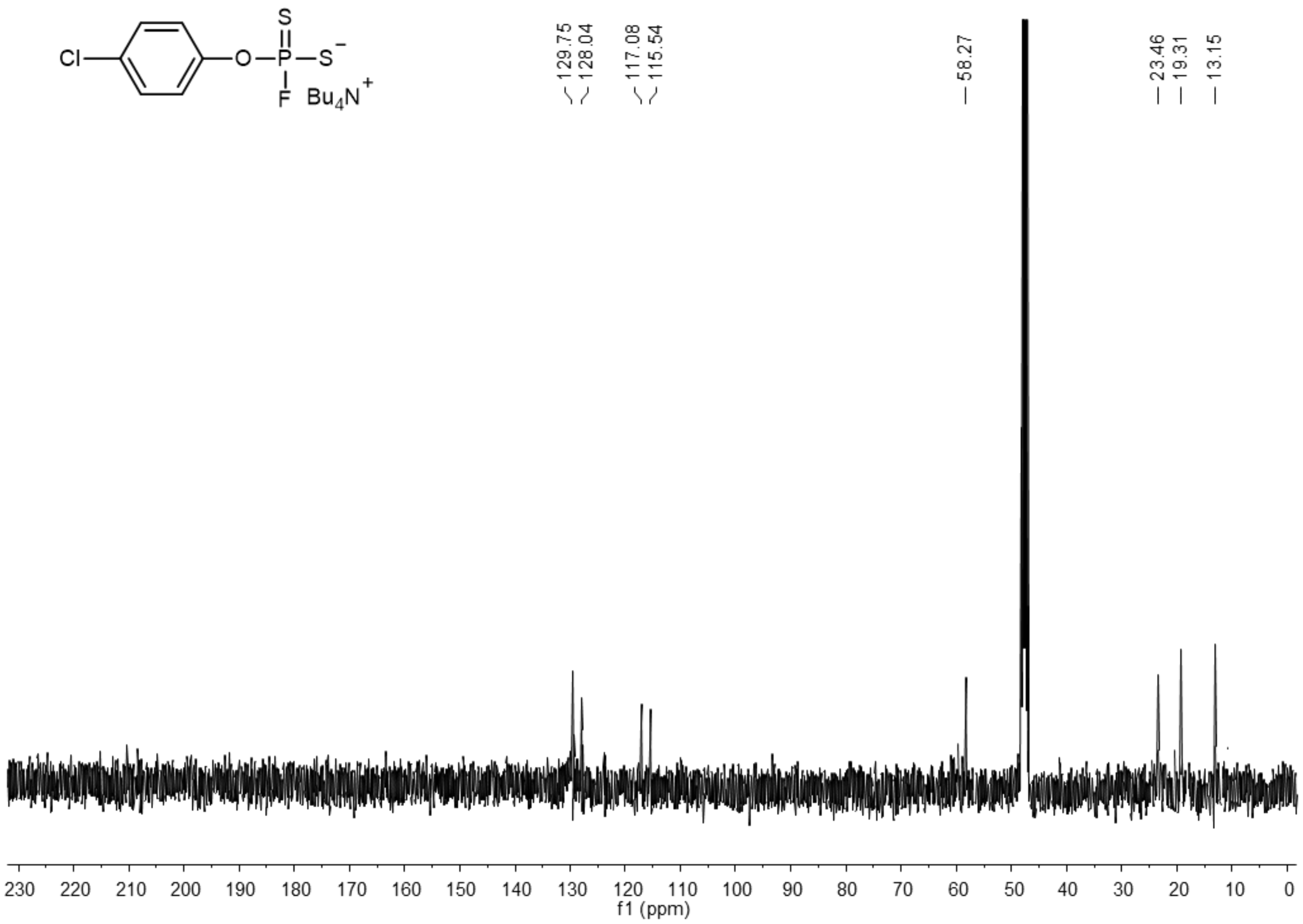

Figure A95. **^{13}C NMR (101 MHz, CD_3OD)** spectrum of **13**.

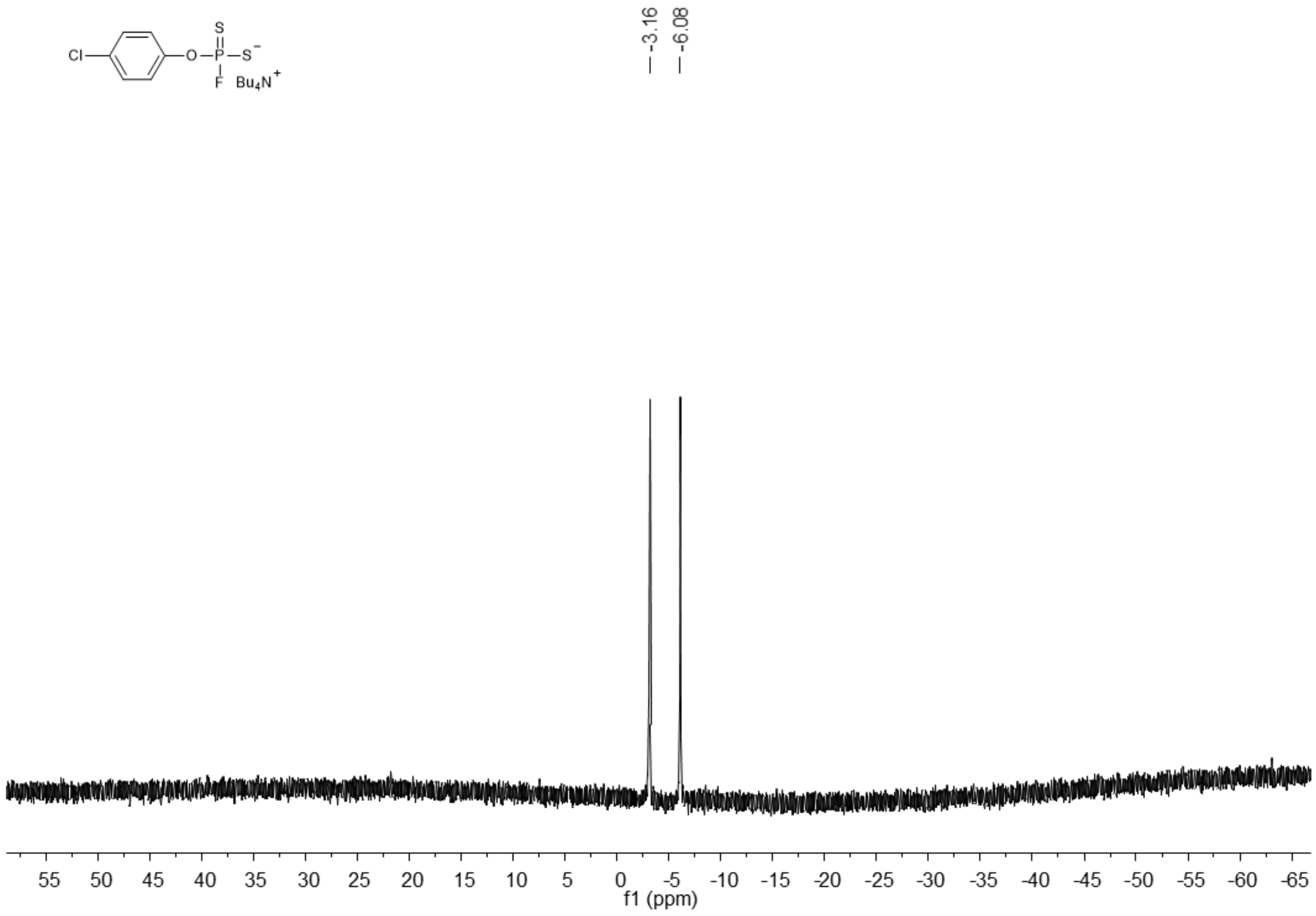

Figure A96. **^{19}F NMR (376 MHz, CD_3OD)** spectrum of **13**.

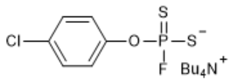

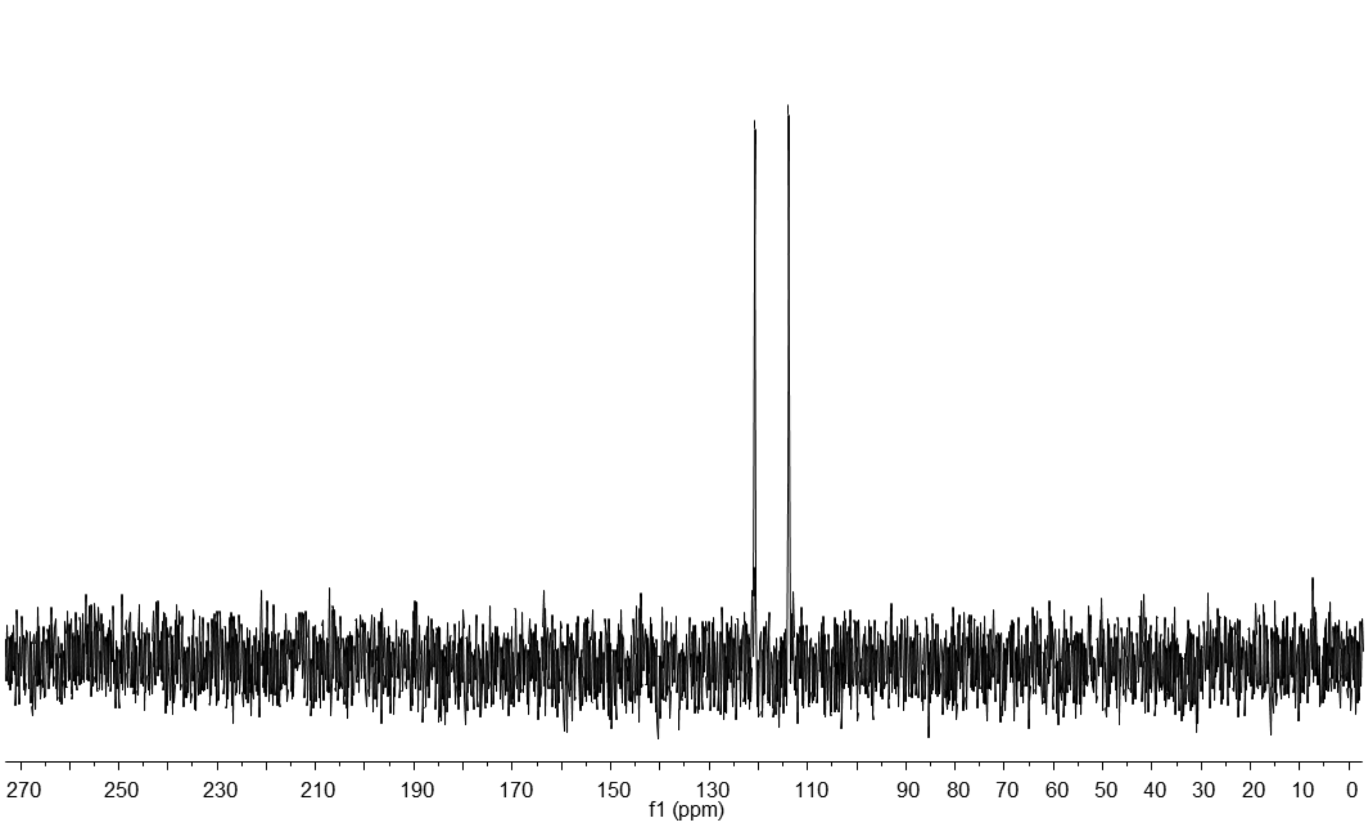

Figure A97. **^{31}P NMR (162 MHz, CD_3OD)** spectrum of **13**.

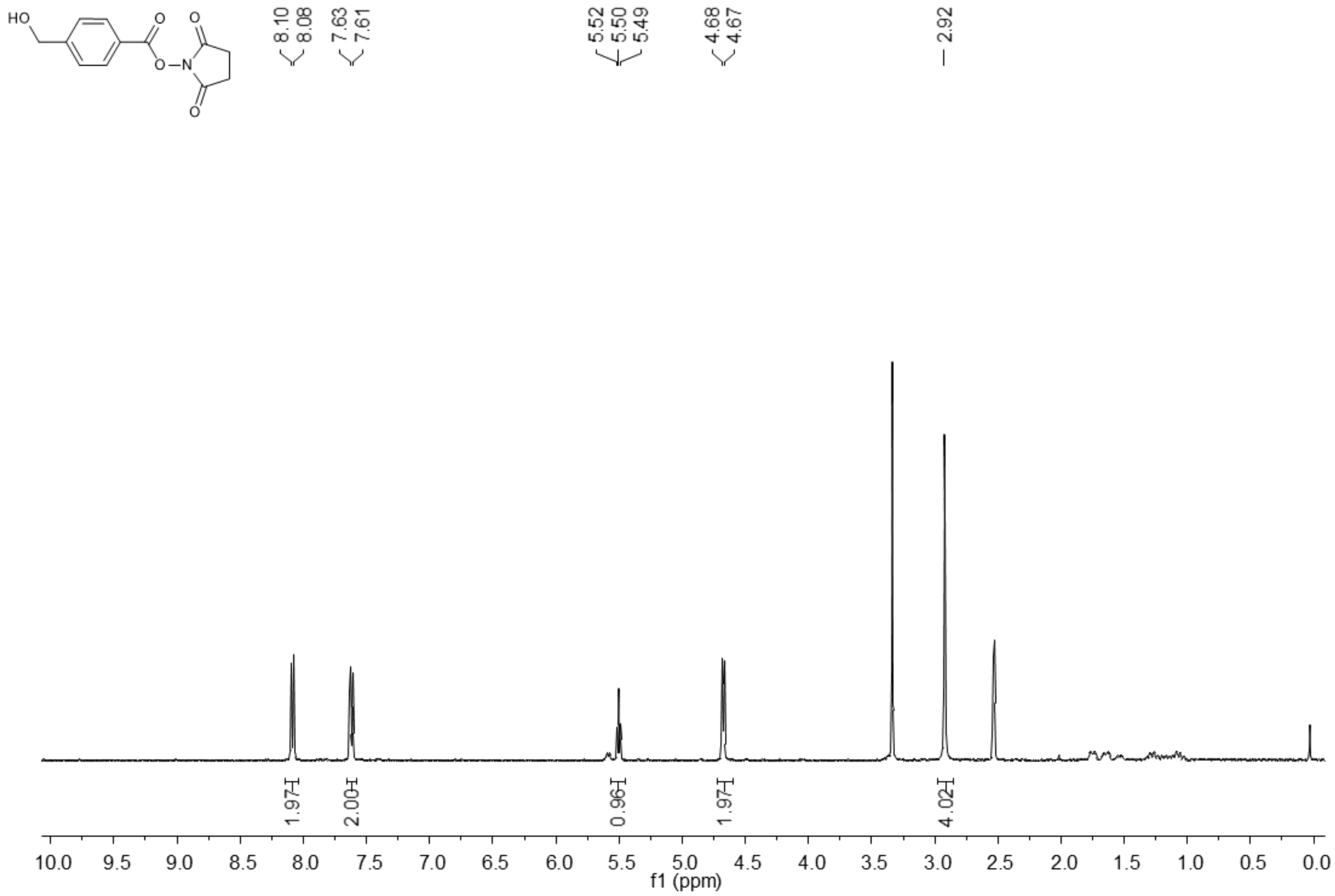

Figure A98. **^{1}H NMR (400 MHz, DMSO-*d_6*)** spectrum of **21**.

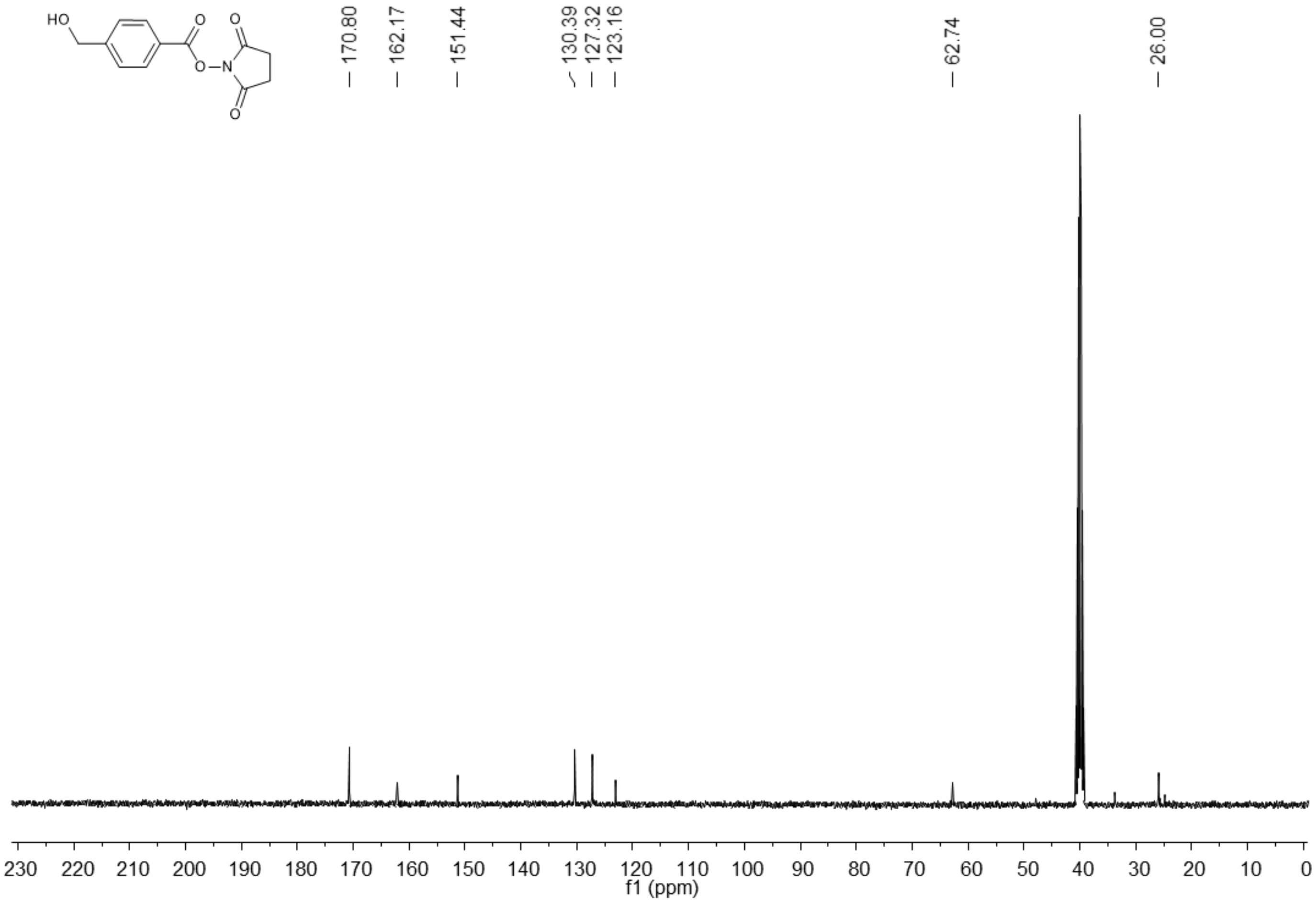

Figure A99. ^{13}**C NMR (101 MHz, DMSO-*d*$_6$**) spectrum of **21**.

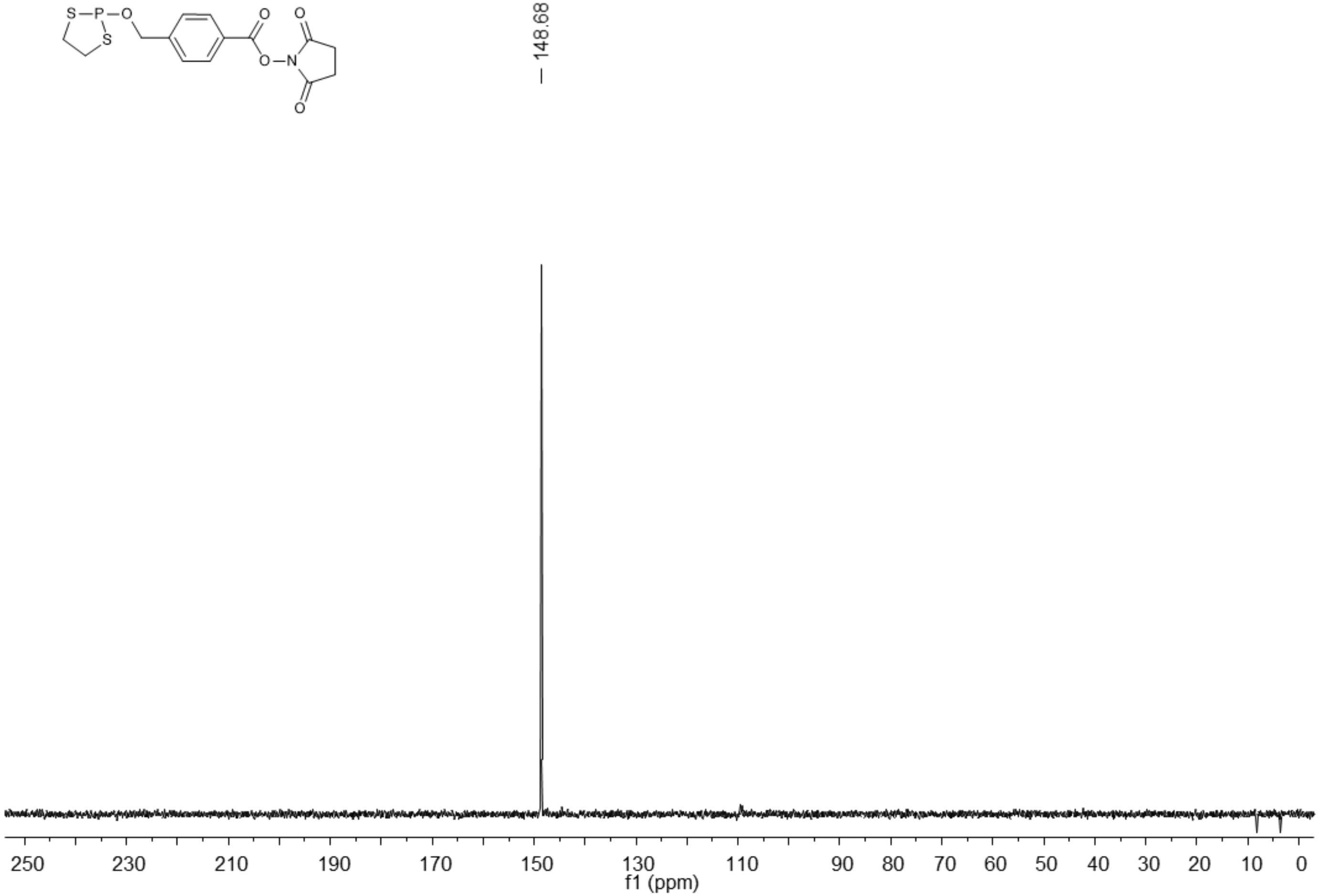

Figure A100. **^{31}P NMR (162 MHz, CDCl$_3$)** spectrum of **15a**.

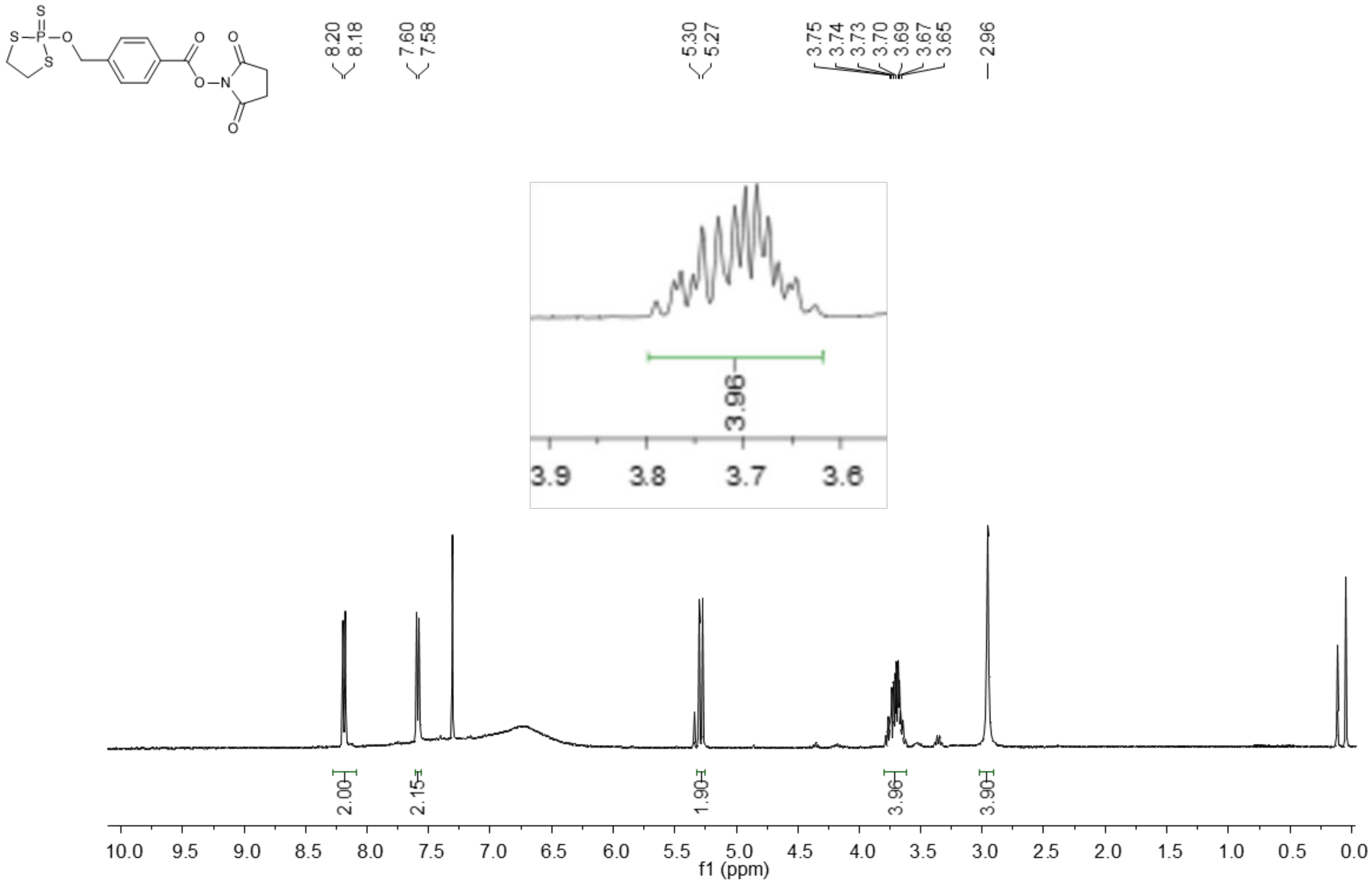

Figure A101. **^{1}H NMR (400 MHz, $CDCl_3$)** spectrum of **15b**.

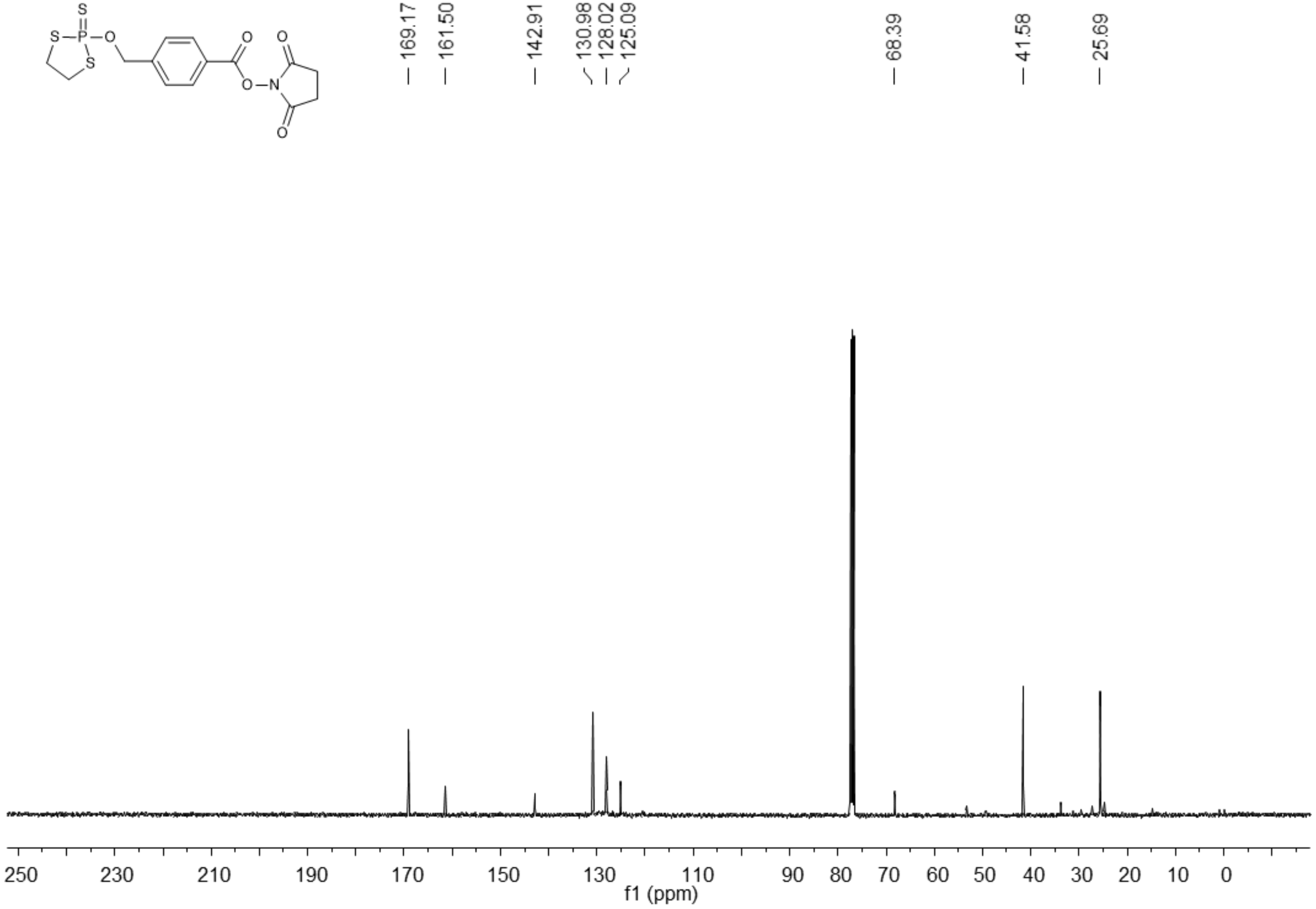

Figure A102. **^{13}C NMR (101 MHz, $CDCl_3$)** spectrum of **15b**.

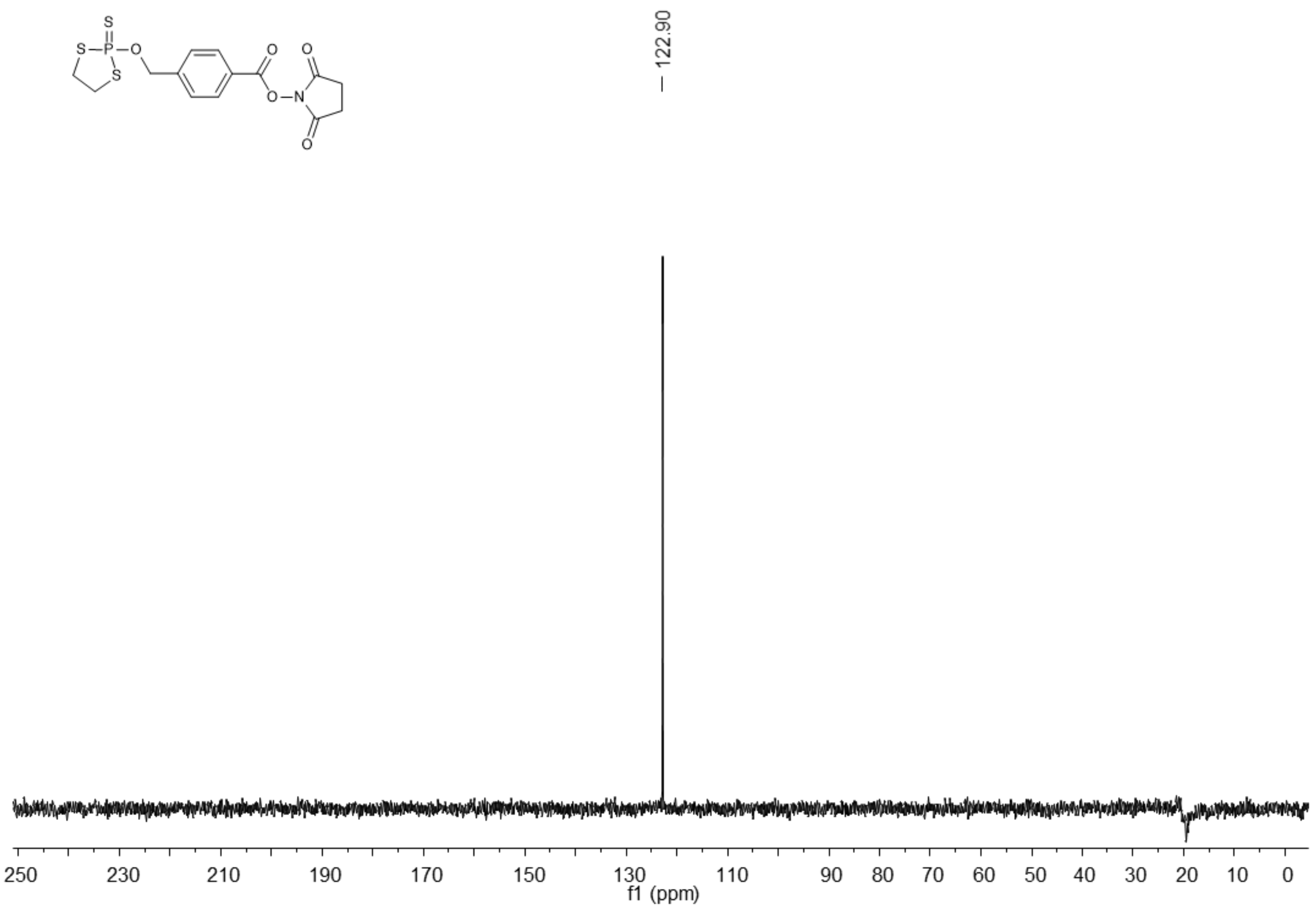

Figure A103. **^{31}P NMR (162 MHz, $CDCl_3$)** spectrum of **15b**.

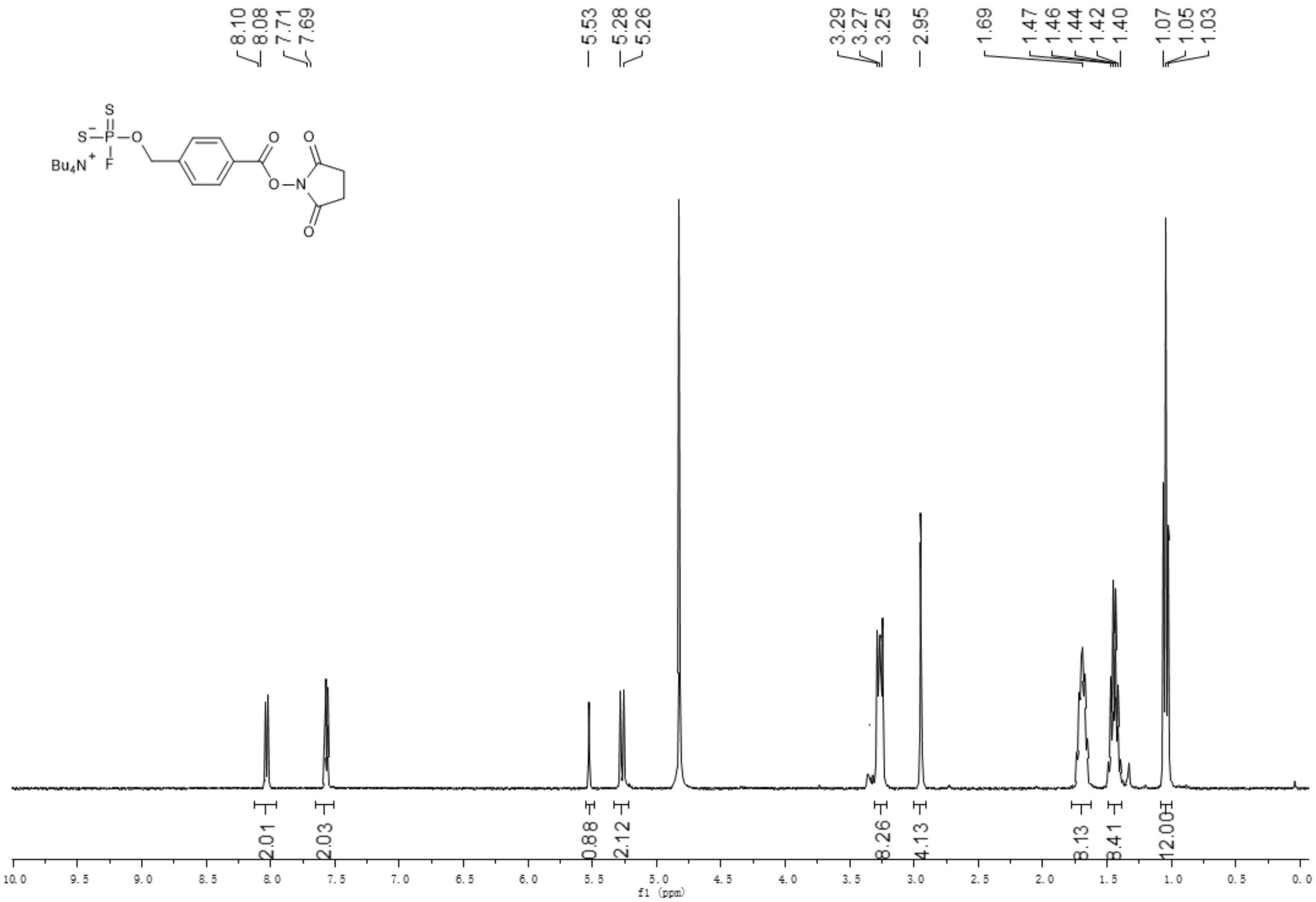

Figure A104. **^{1}H NMR (400 MHz, CD_3OD)** spectrum of **15**.

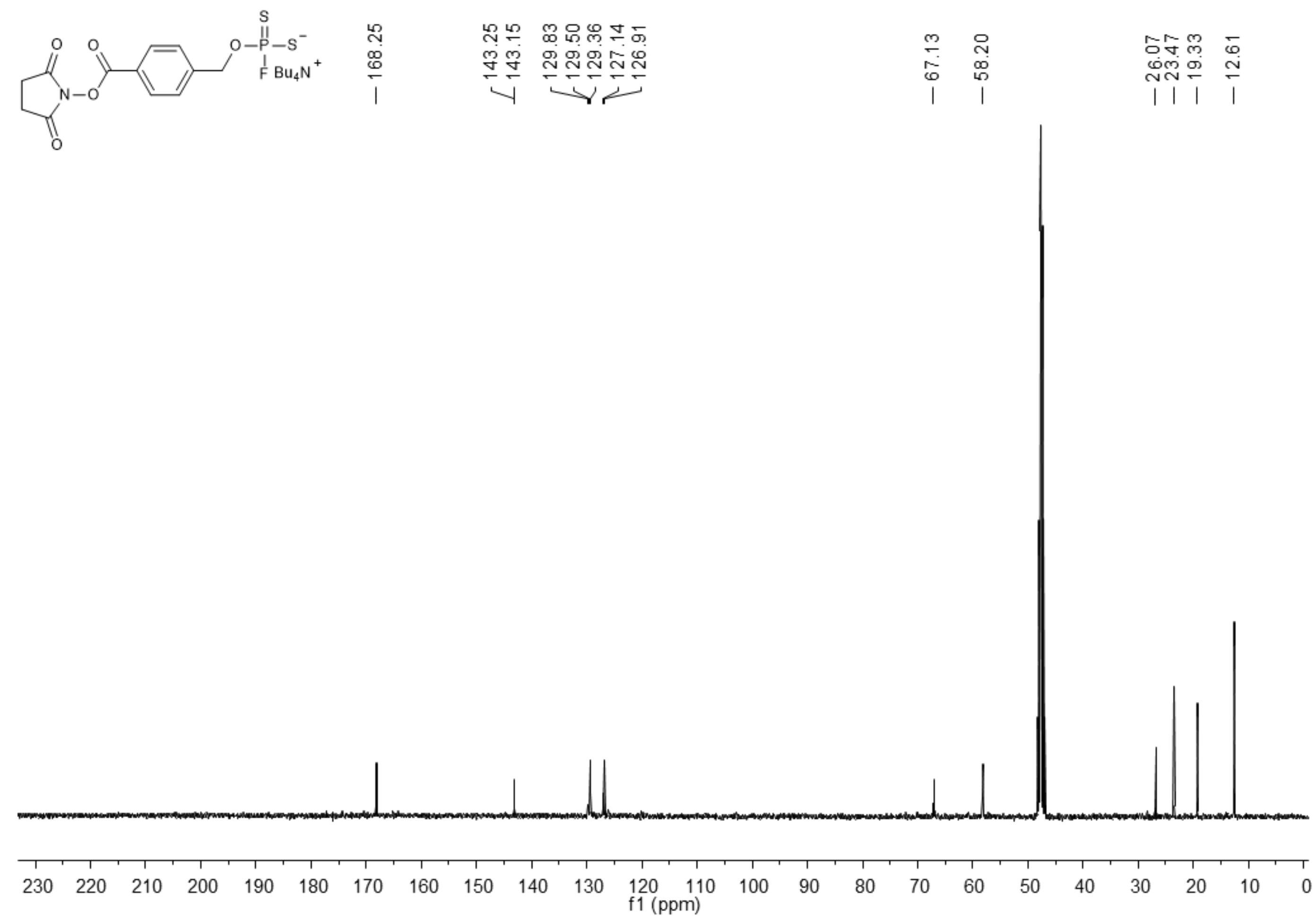

Figure A105. **^{13}C NMR (101 MHz, CD_3OD)** spectrum of **15**.

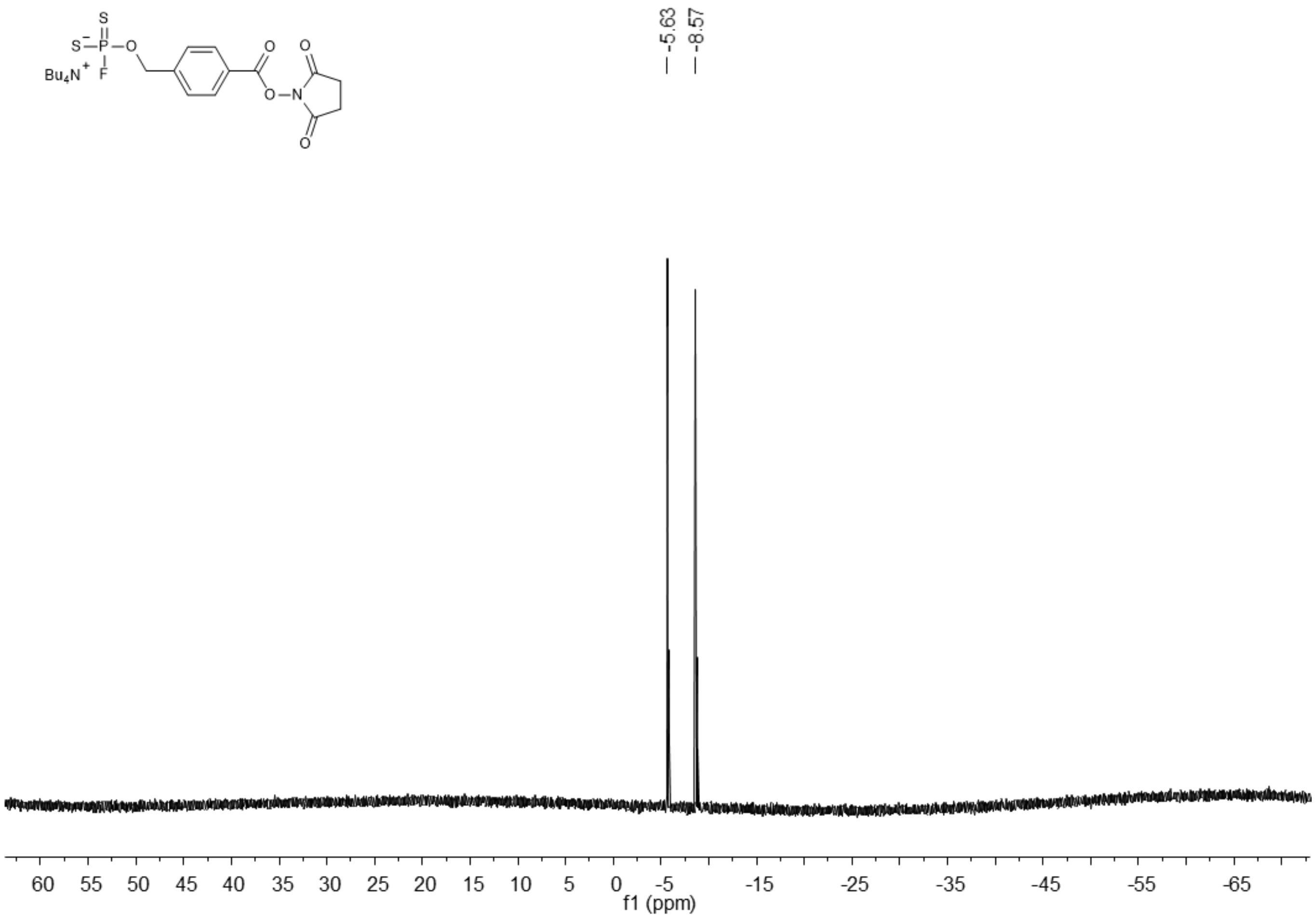

Figure A106. **^{19}F NMR (376 MHz, CD_3OD)** spectrum of **15**.

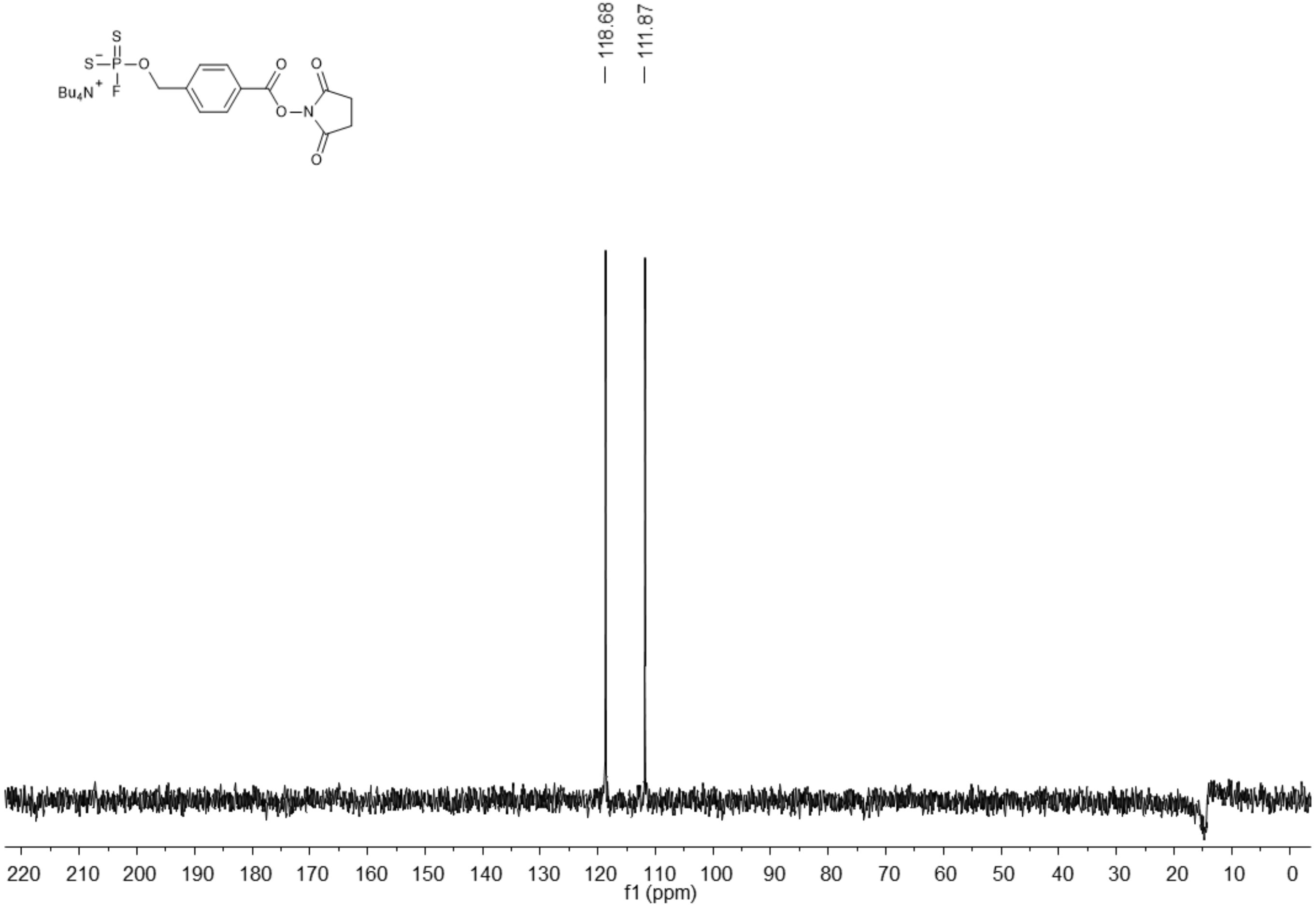

Figure A107. **^{31}P NMR (162 MHz, CD$_3$OD)** spectrum of **15**.

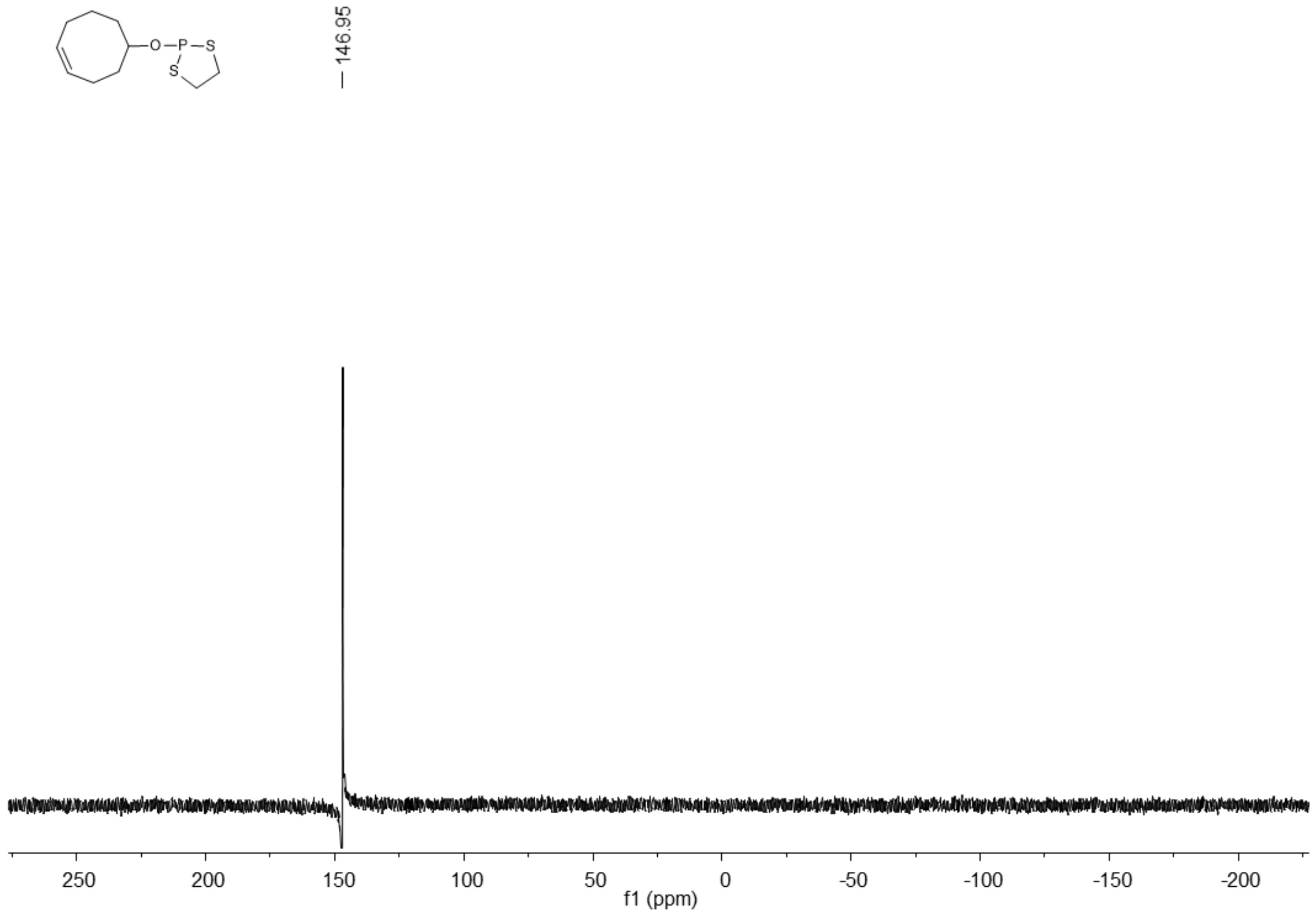

Figure A108. **^{31}P NMR (162 MHz, $CDCl_3$)** spectrum of **16a**.

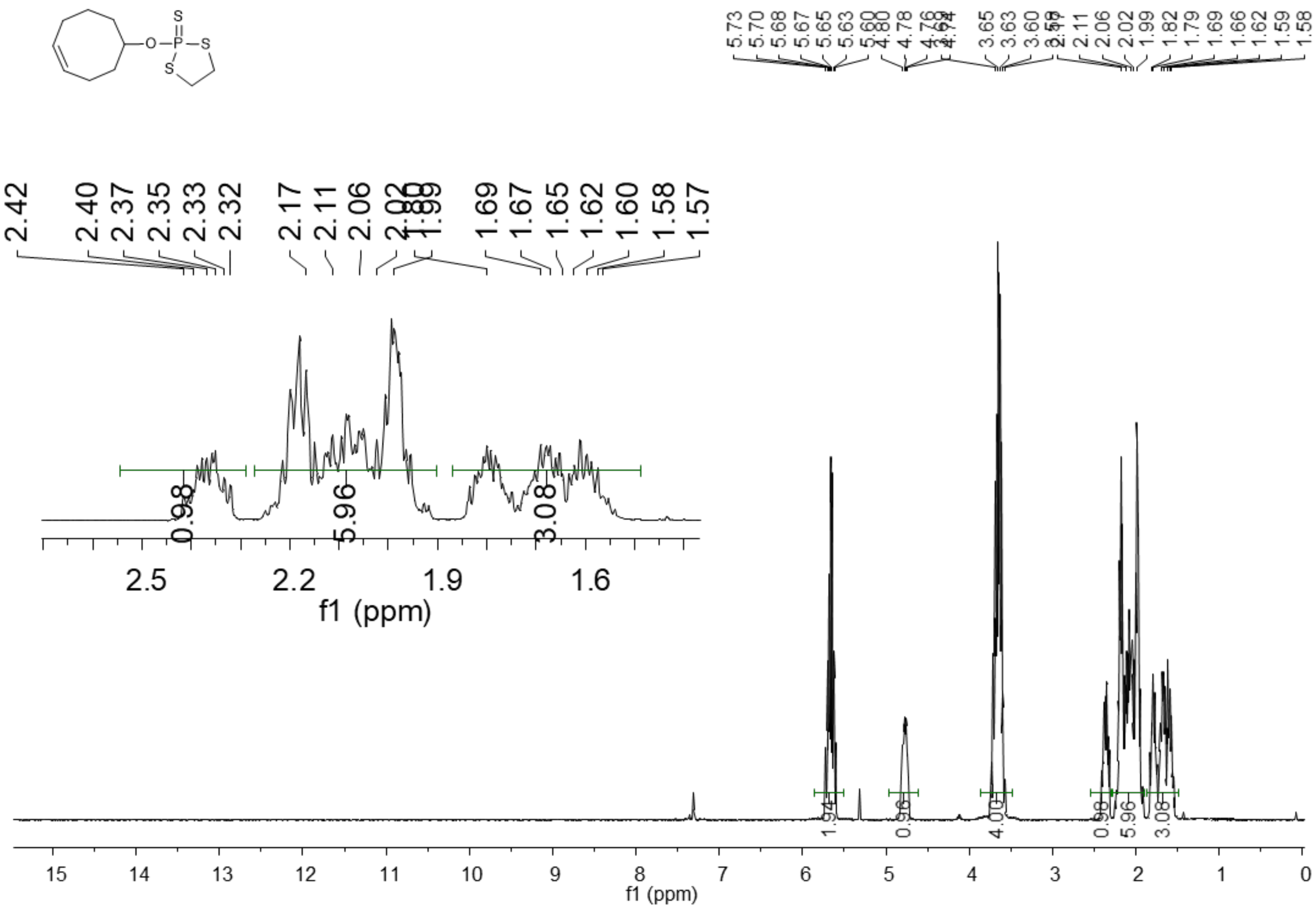

Figure A109. **^{1}H NMR (400 MHz, $CDCl_3$)** spectrum of **16b**.

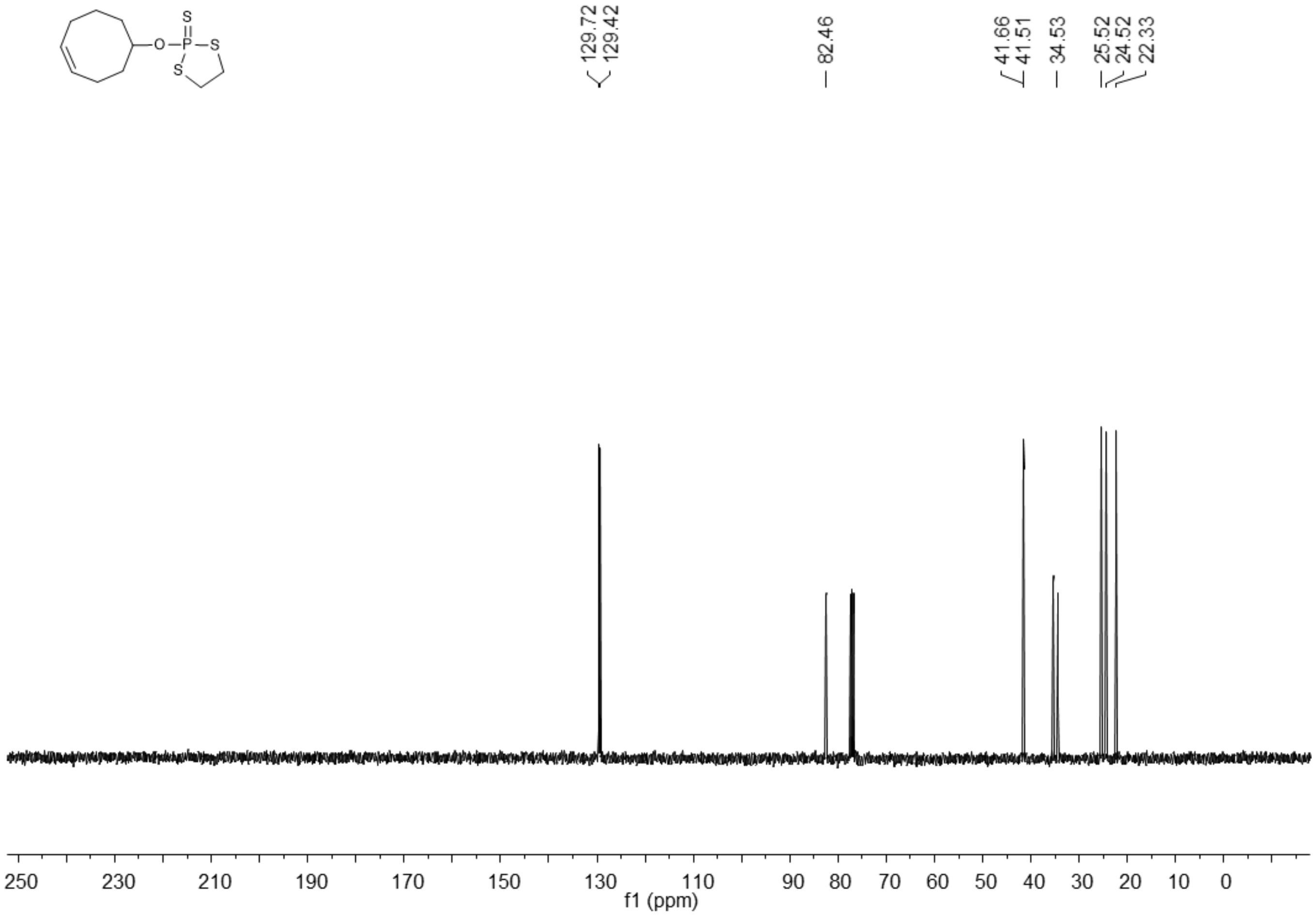

Figure A110. **^{13}C NMR (101 MHz, $CDCl_3$)** spectrum of **16b**.

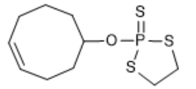

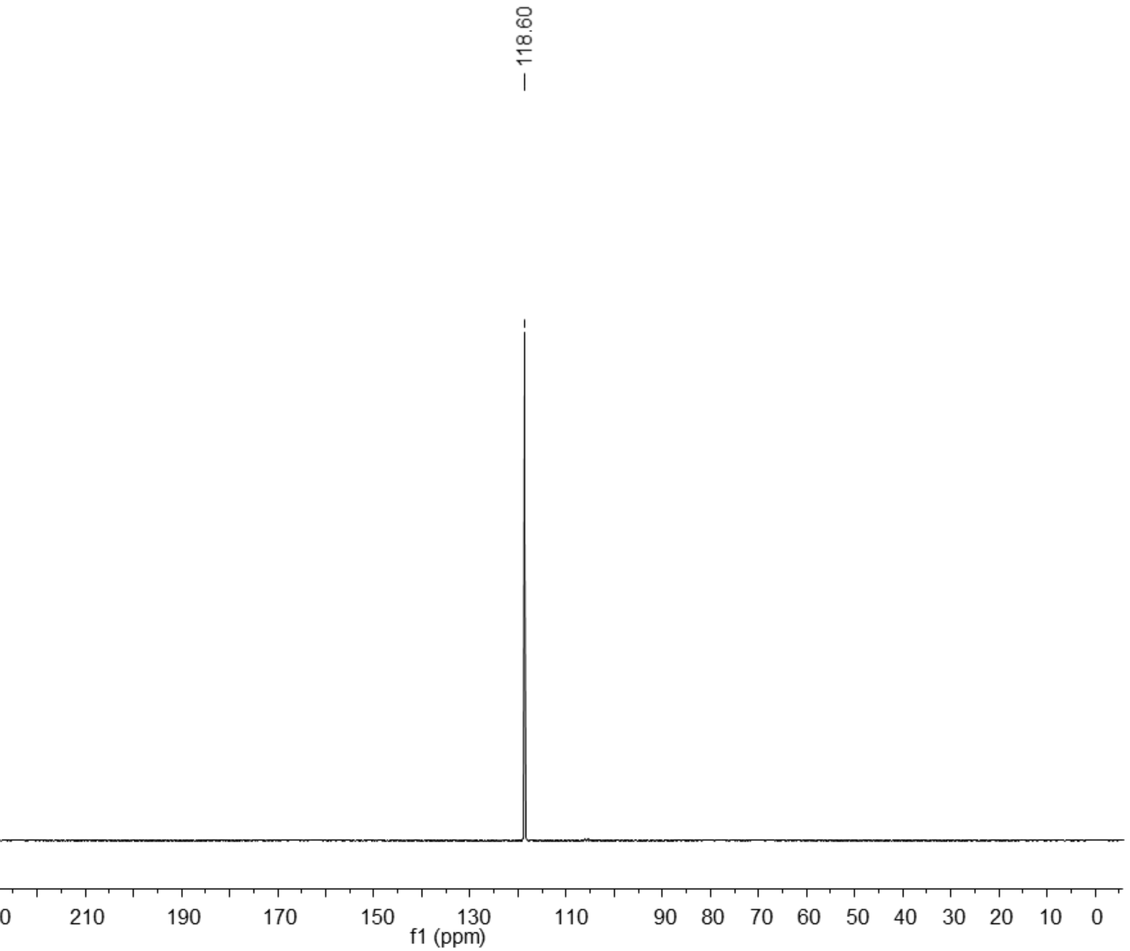

Figure A111. **^{31}P NMR (162 MHz, $CDCl_3$)** spectrum of **16b**.

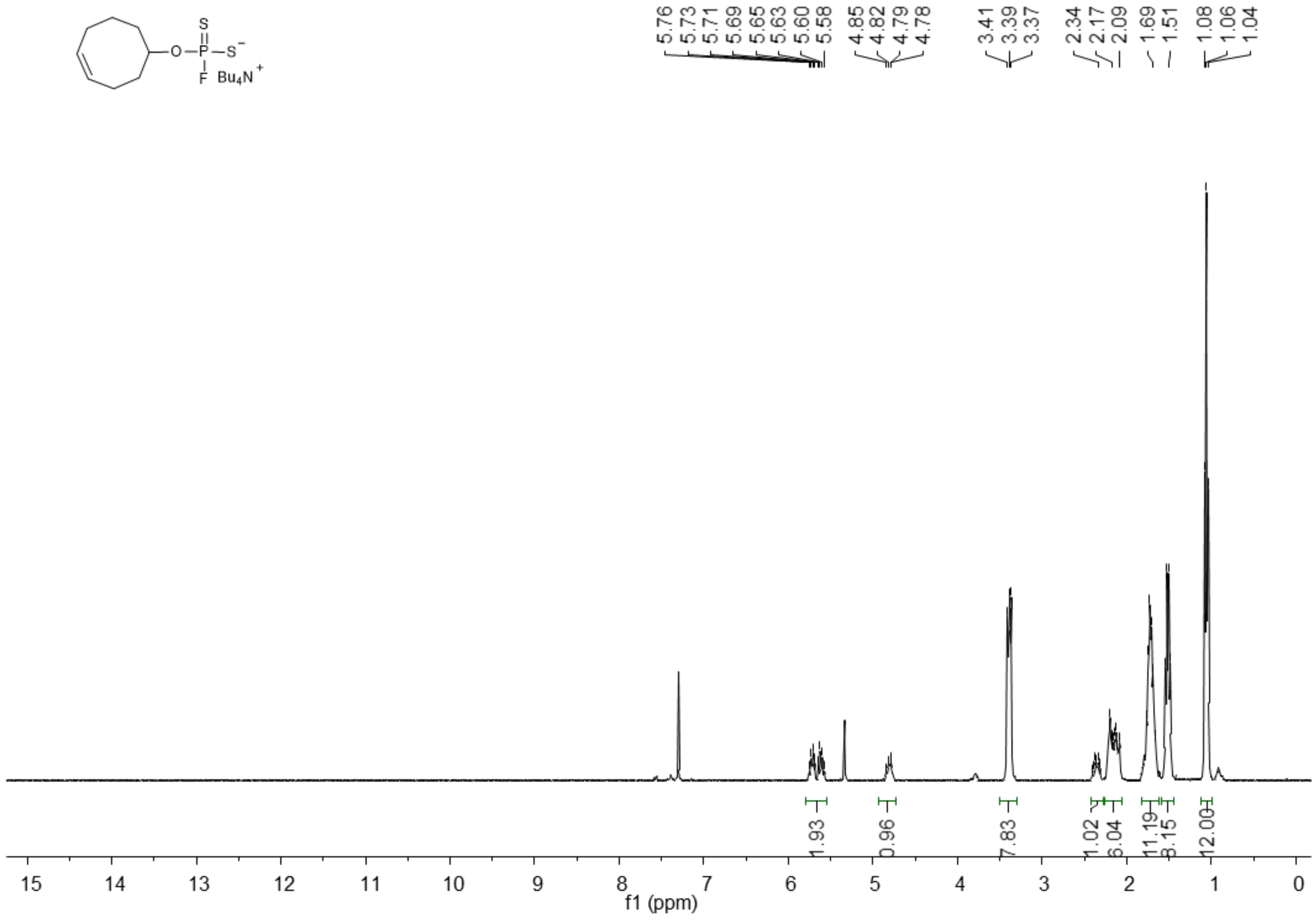

Figure A112. **^{1}H NMR (400 MHz, CDCl3)** spectrum of **16**.

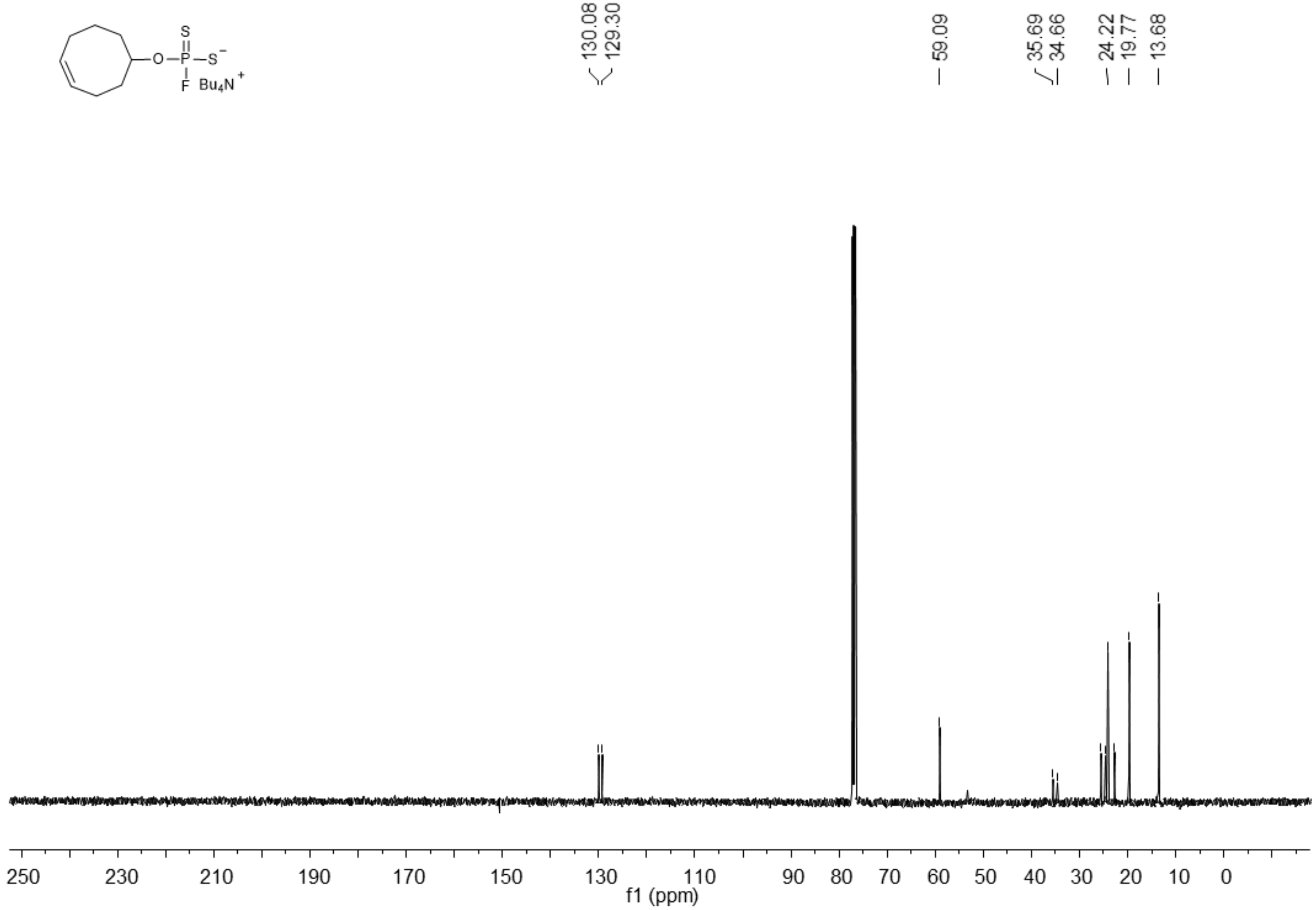

Figure A113. **^{13}C NMR (101 MHz, $CDCl_3$)** spectrum of **16**.

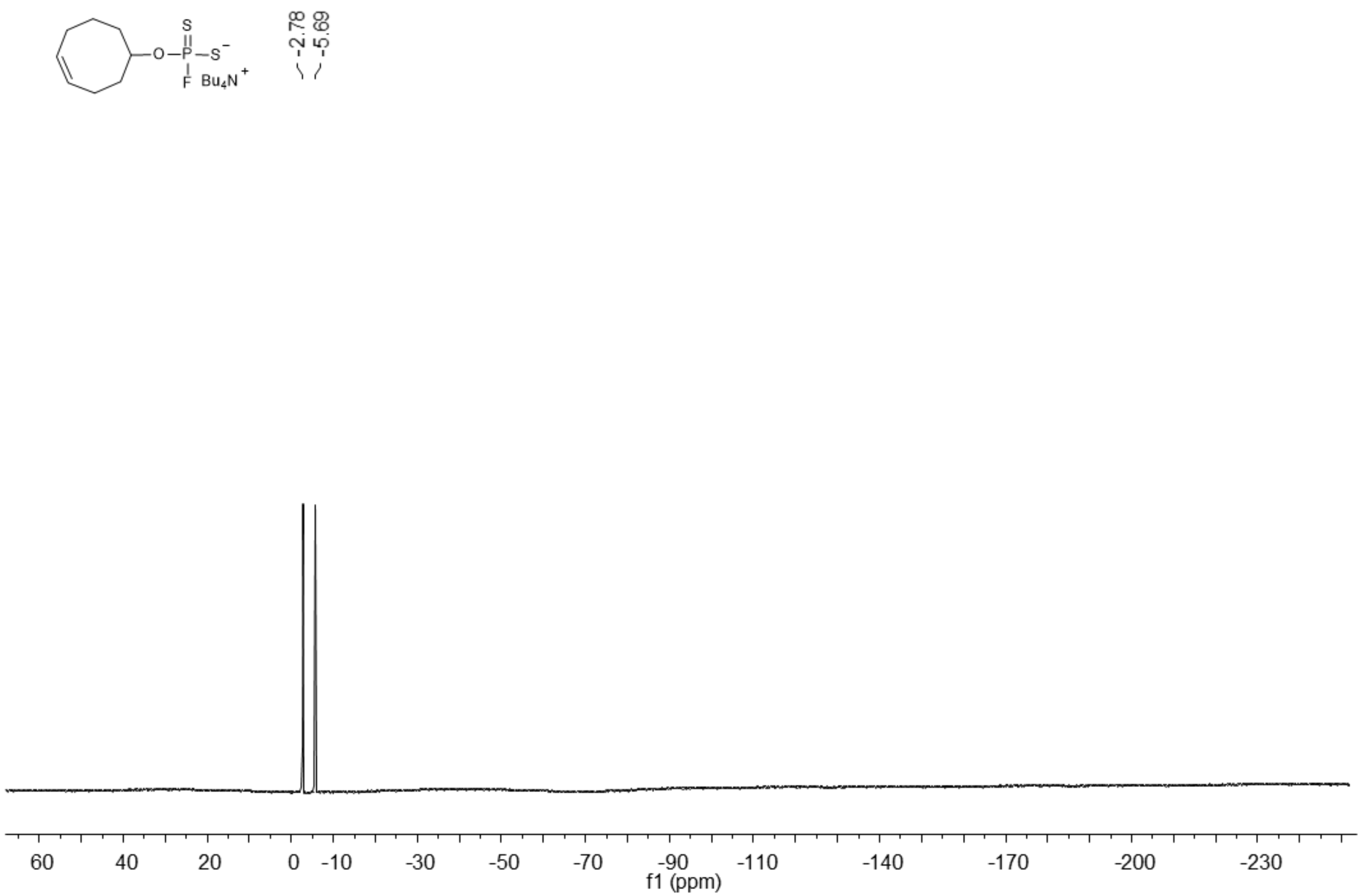

Figure A114. **^{19}F NMR (376 MHz, $CDCl_3$)** spectrum of **16**.

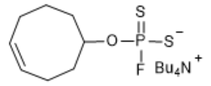

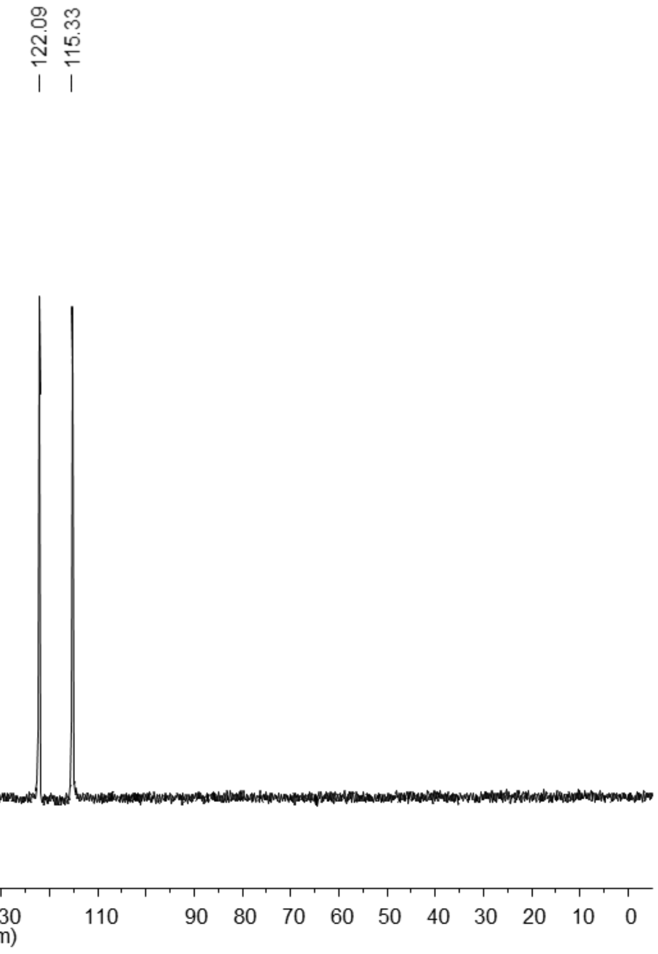

Figure A115. **^{31}P NMR (162 MHz, CDCl$_3$)** spectrum of **16**.

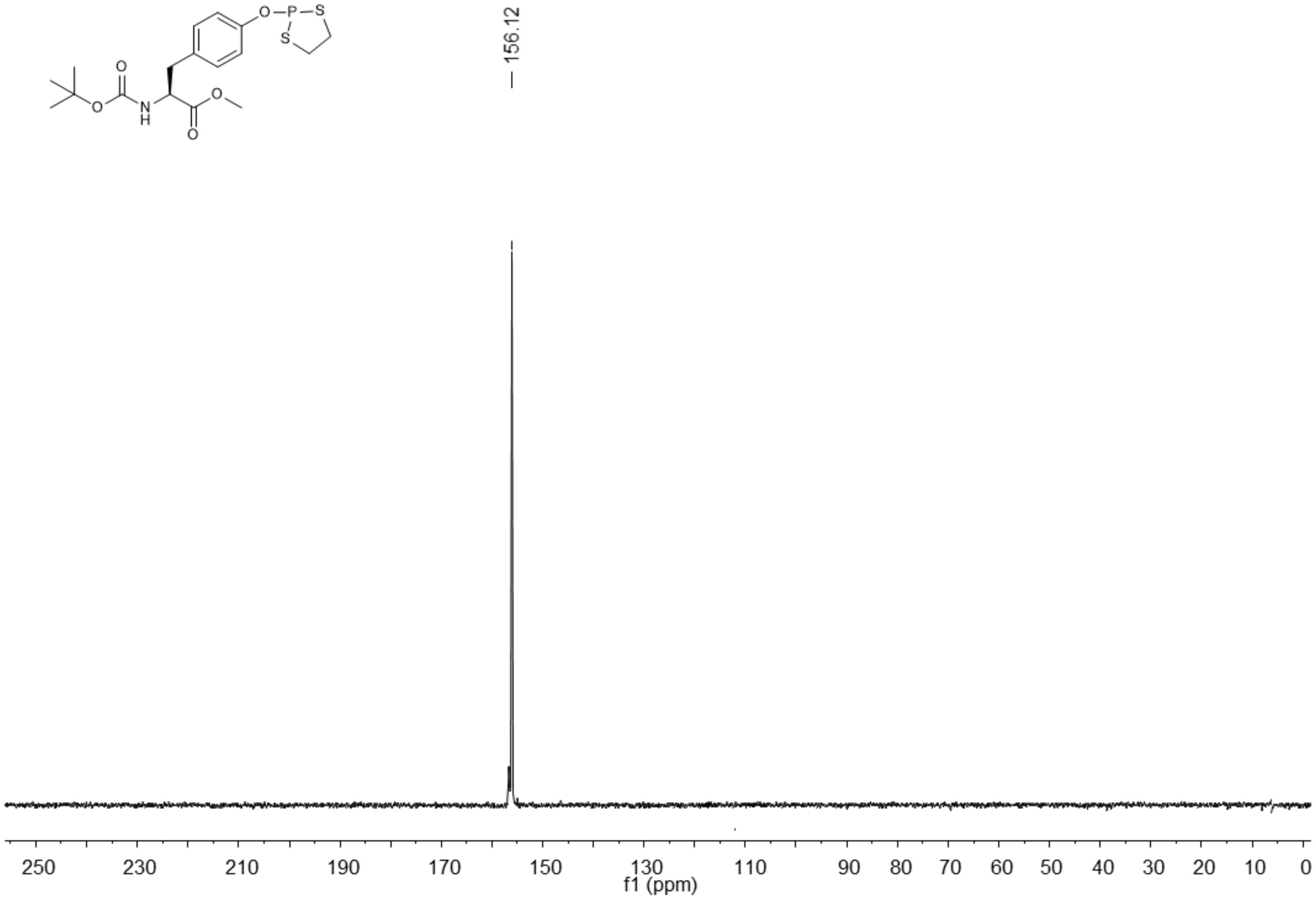

Figure A116. **^{31}P NMR (162 MHz, $CDCl_3$)** spectrum of **17a**.

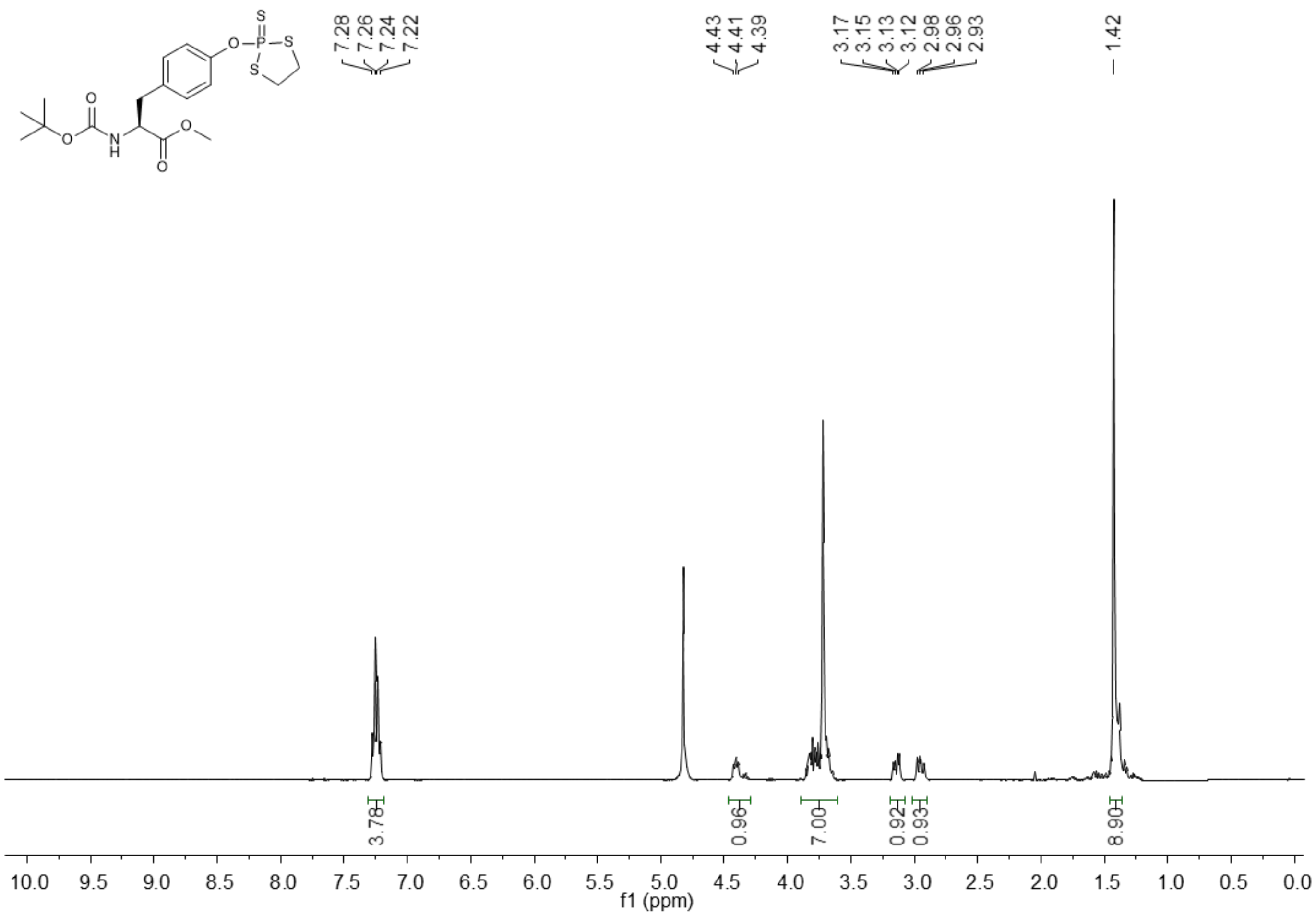

Figure A117. **^{1}H NMR (400 MHz, CD_3OD)** spectrum of **17b**.

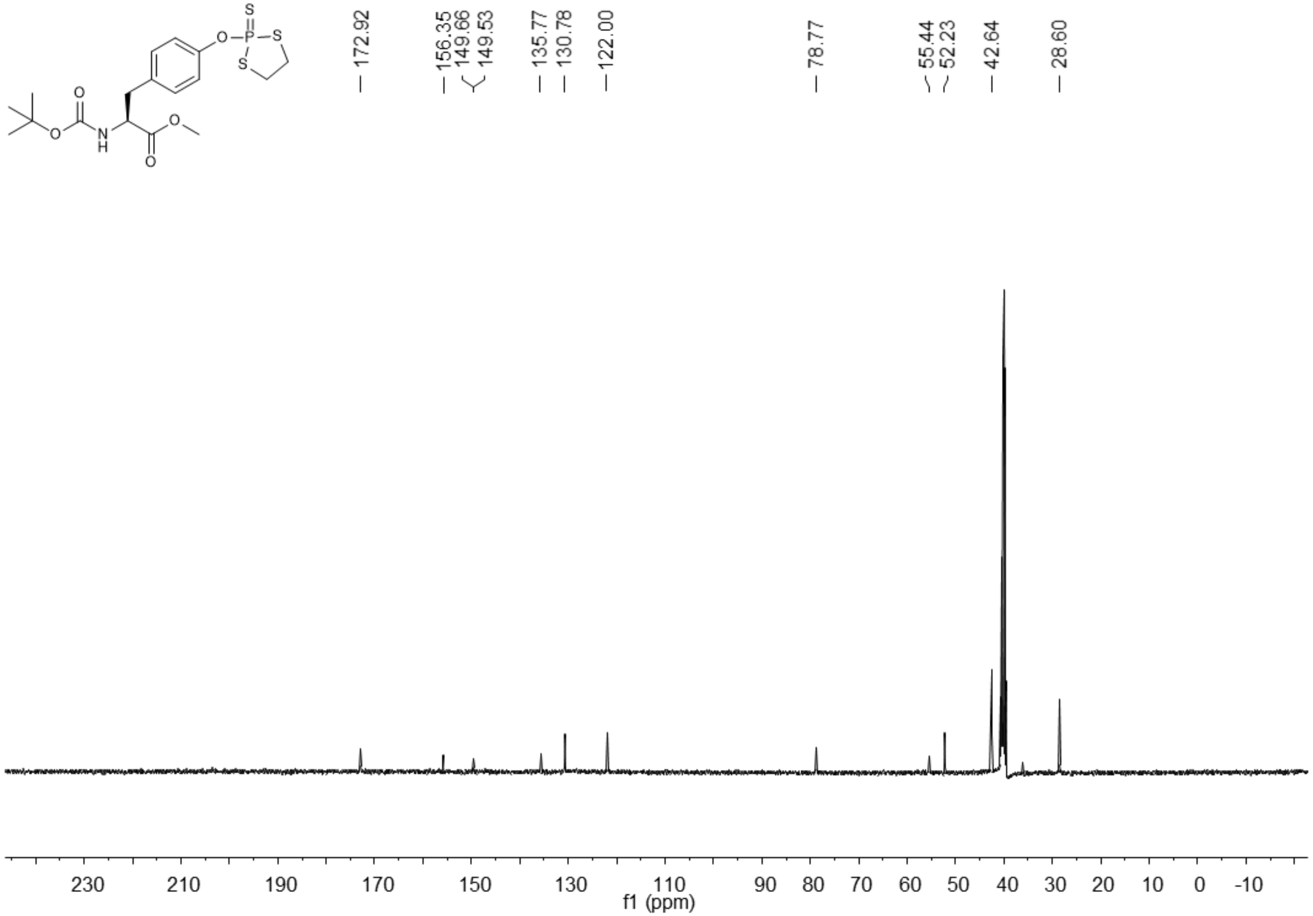

Figure A118. **^{13}C NMR (101 MHz, CD_3OD)** spectrum of **17b**.

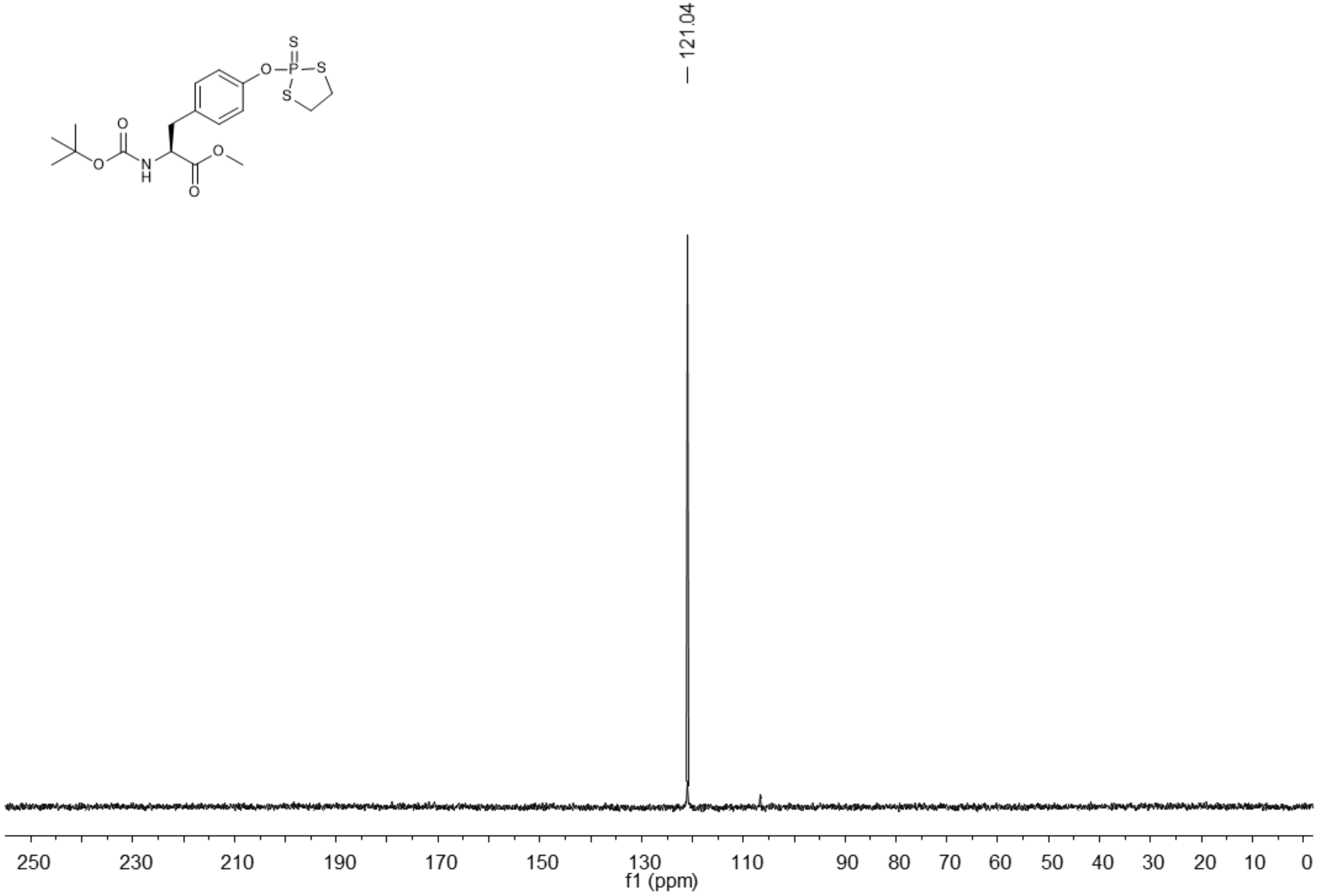

Figure A119. **^{31}P NMR (376 MHz, CD_3OD)** spectrum of **17b**.

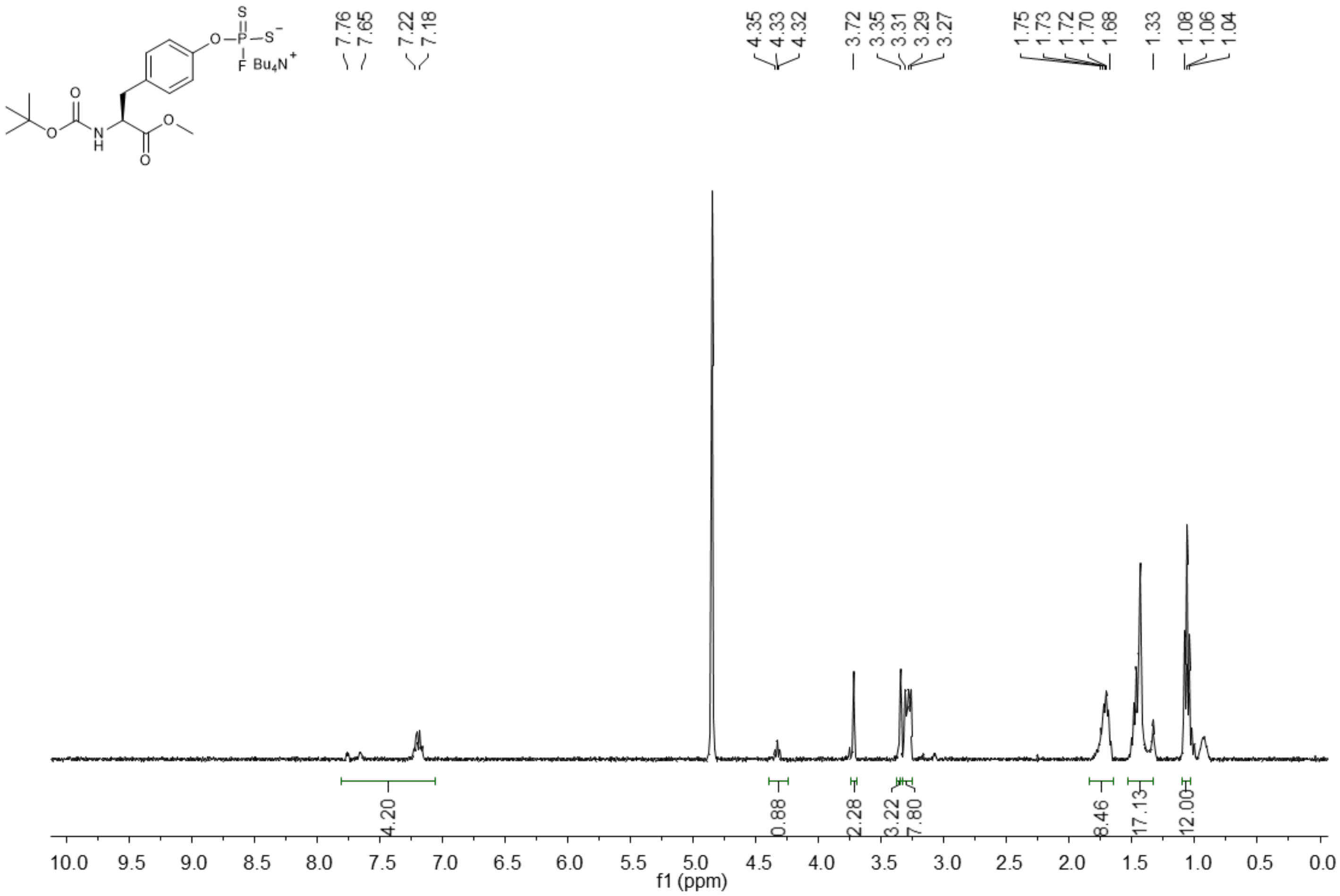

Figure A120. **^{1}H NMR (400 MHz, CD_3OD)** spectrum of **17c**.

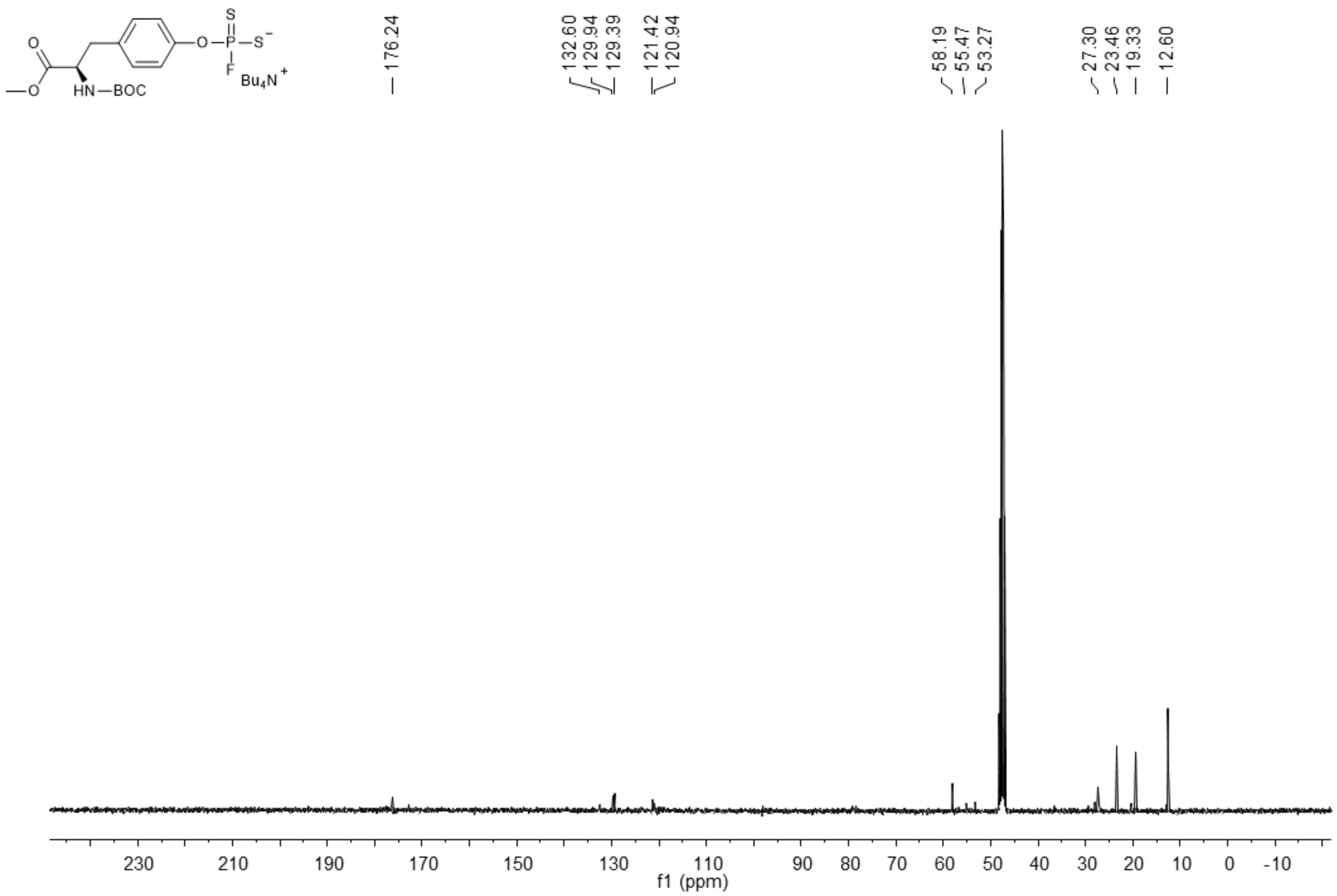

Figure A121. **^{13}C NMR (101 MHz, CD_3OD)** spectrum of **17c**.

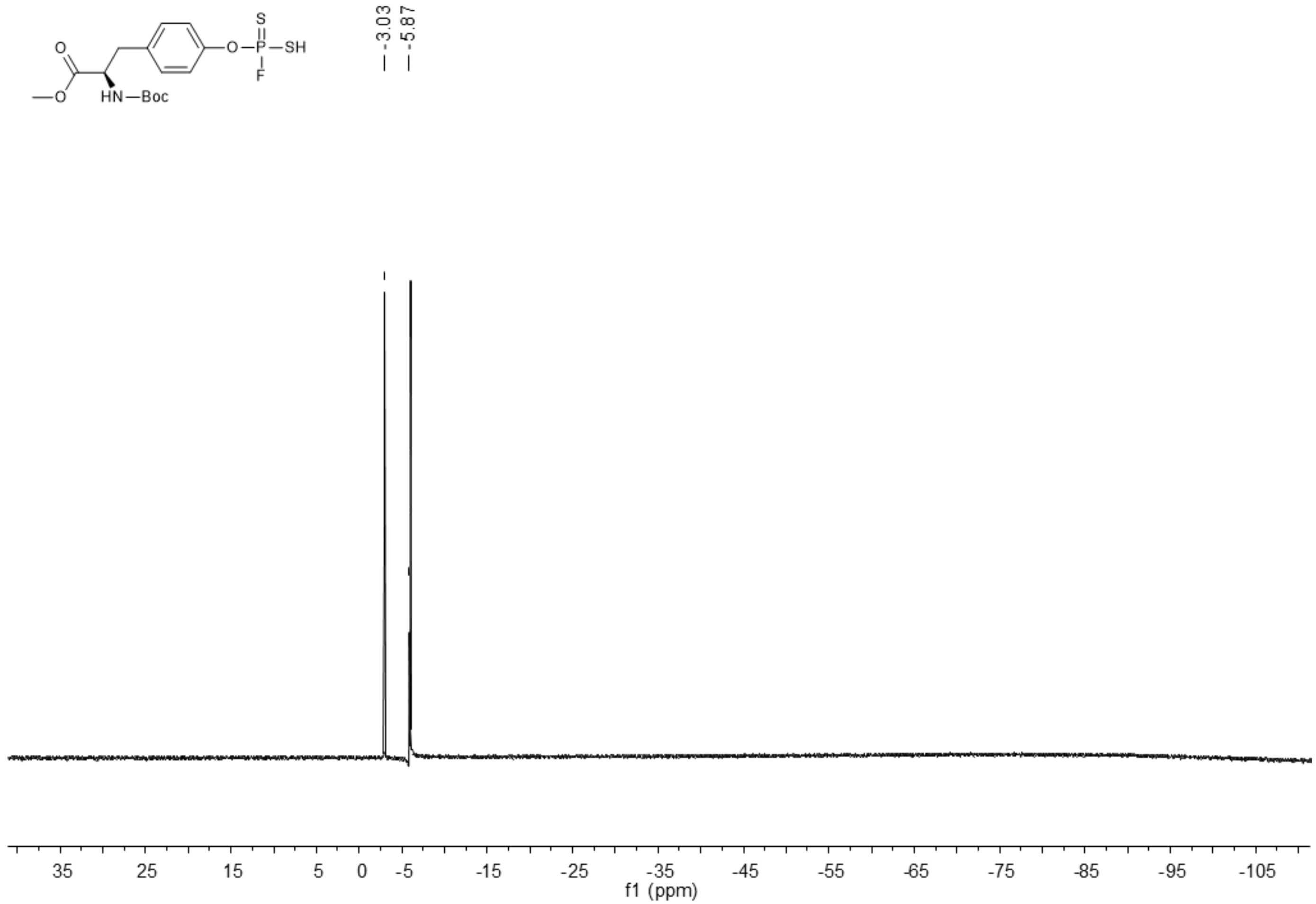

Figure A122. **^{19}F NMR (376 MHz, CD_3OD)** spectrum of **17c**.

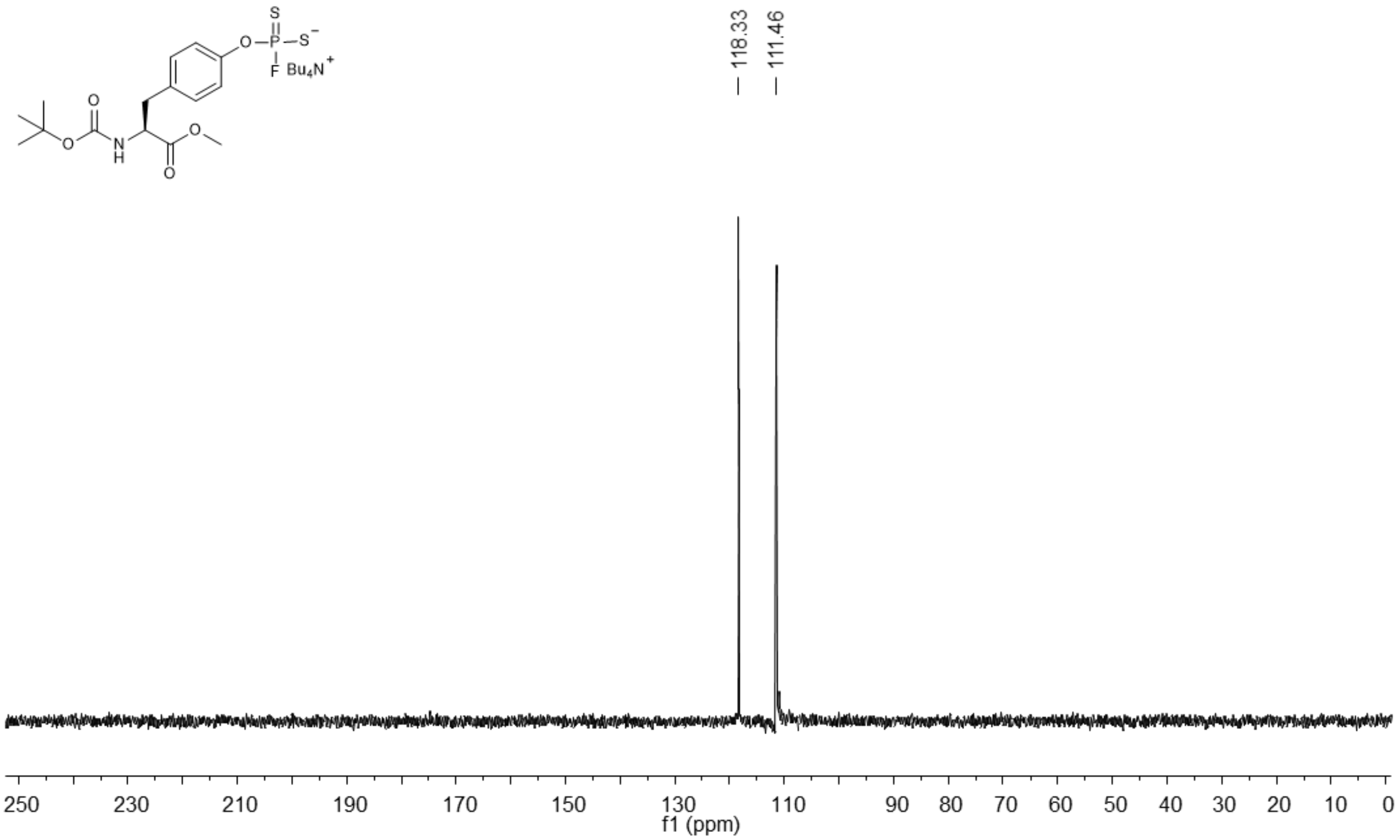

Figure A123. **^{31}P NMR (162 MHz, CD$_3$OD)** spectrum of **17c**.

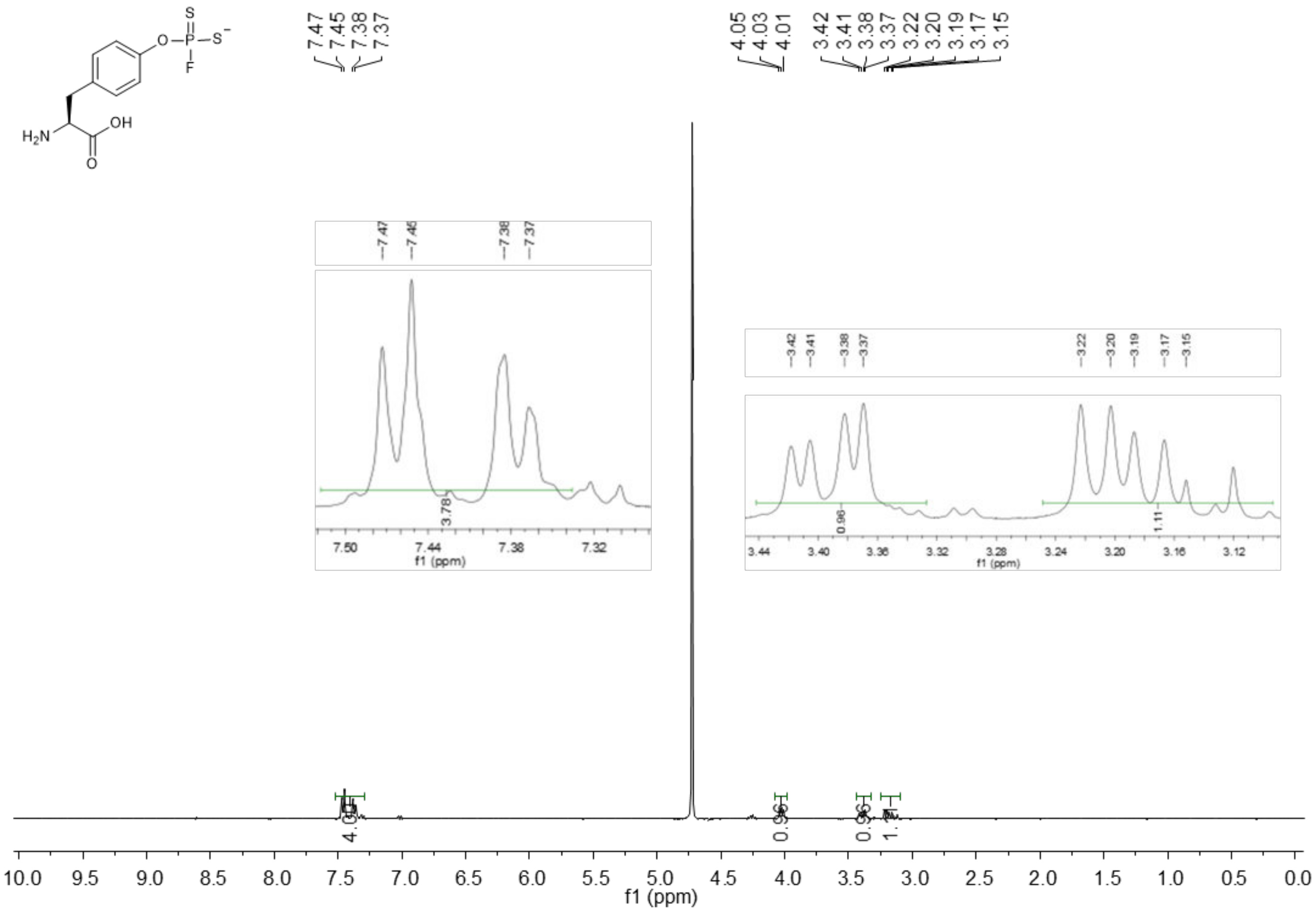

Figure A124. **^{1}H NMR (400 MHz, D_2O)** spectrum of **17**.

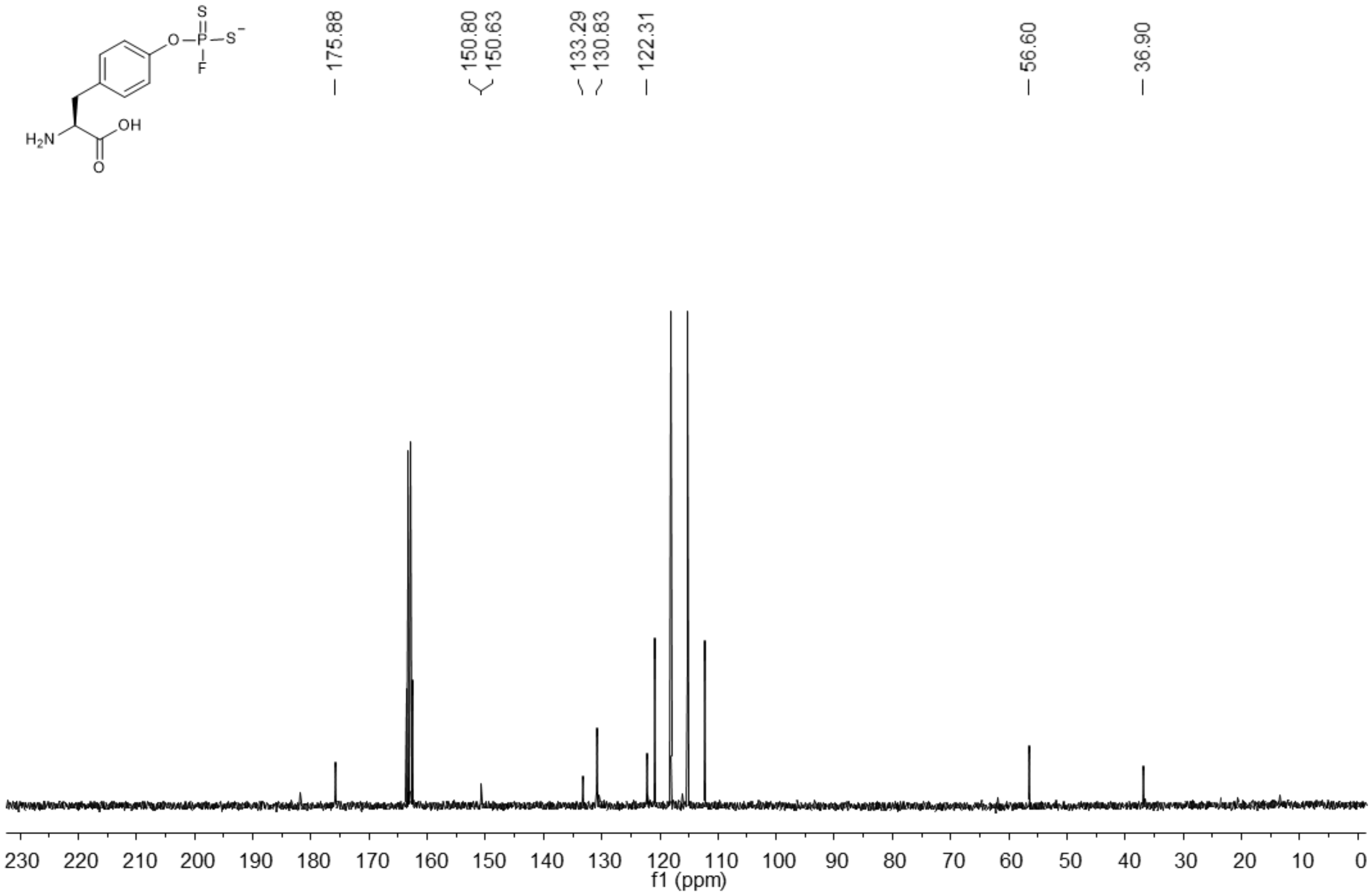

Figure A125. **^{13}C NMR (101 MHz, D_2O)** spectrum of **17**.

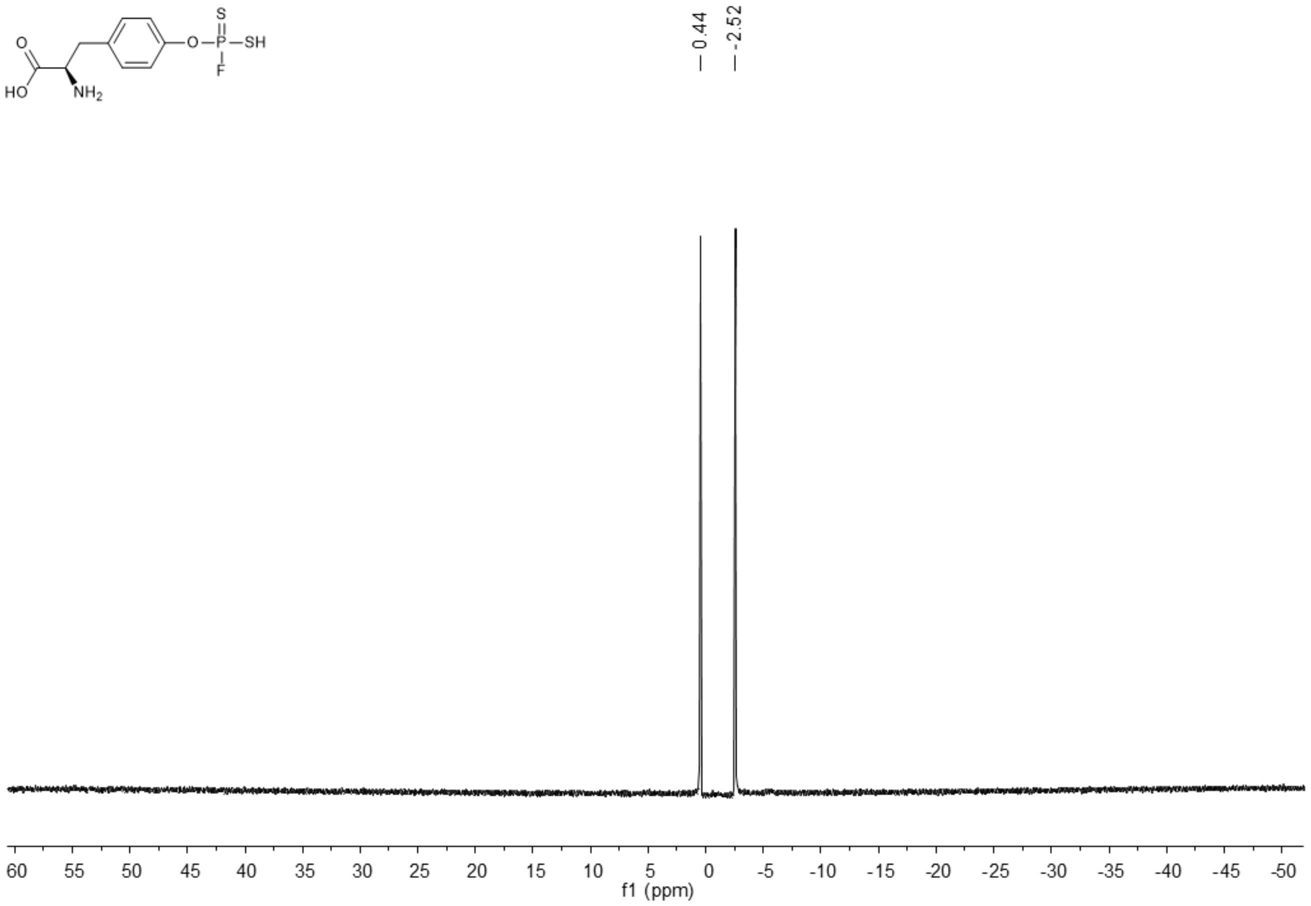

Figure A126. **^{19}F NMR (376 MHz, $CDCl_3$)** spectrum of **17**.

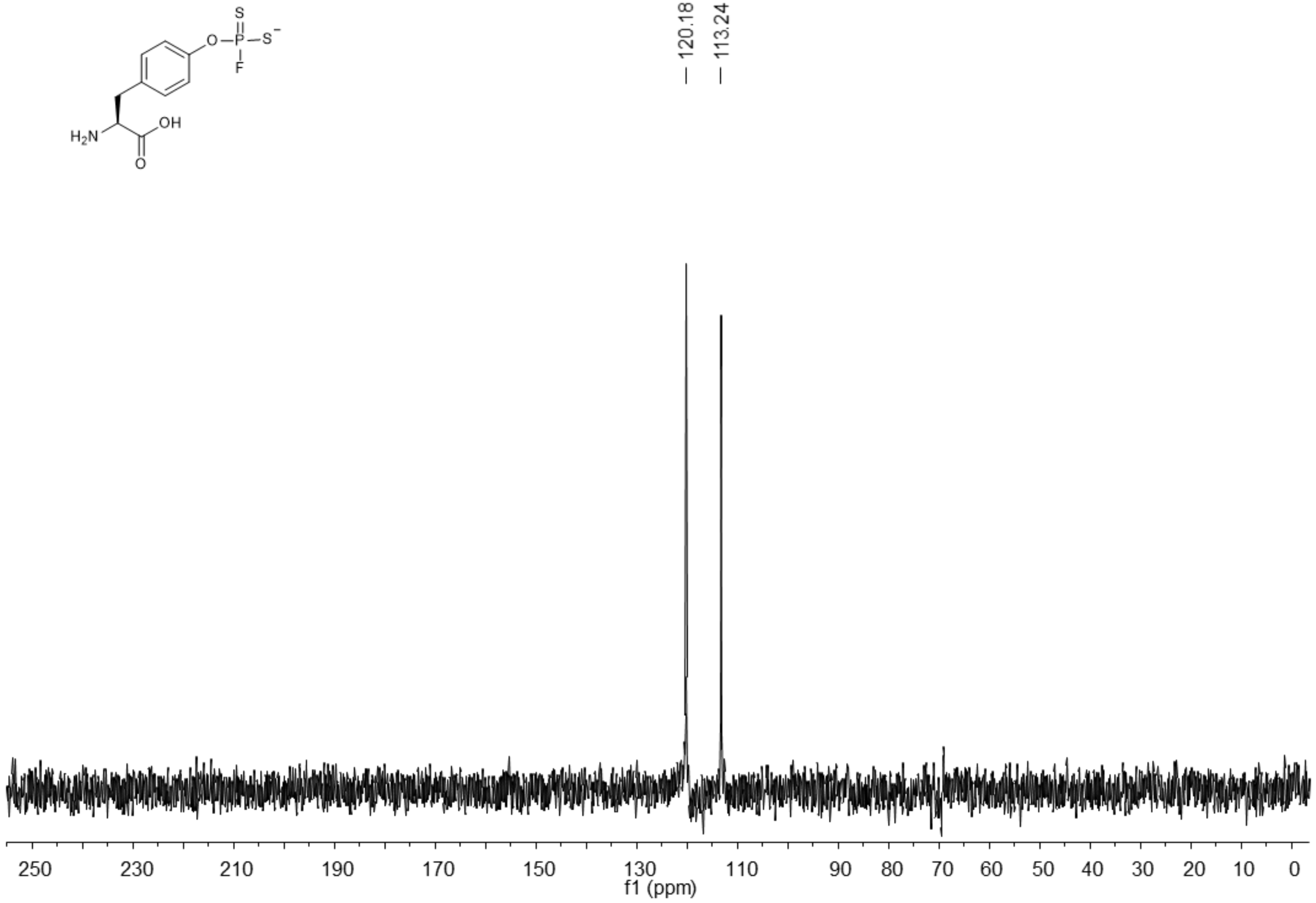

Figure A127. **^{31}P NMR (162 MHz, D_2O)** spectrum of **17**.

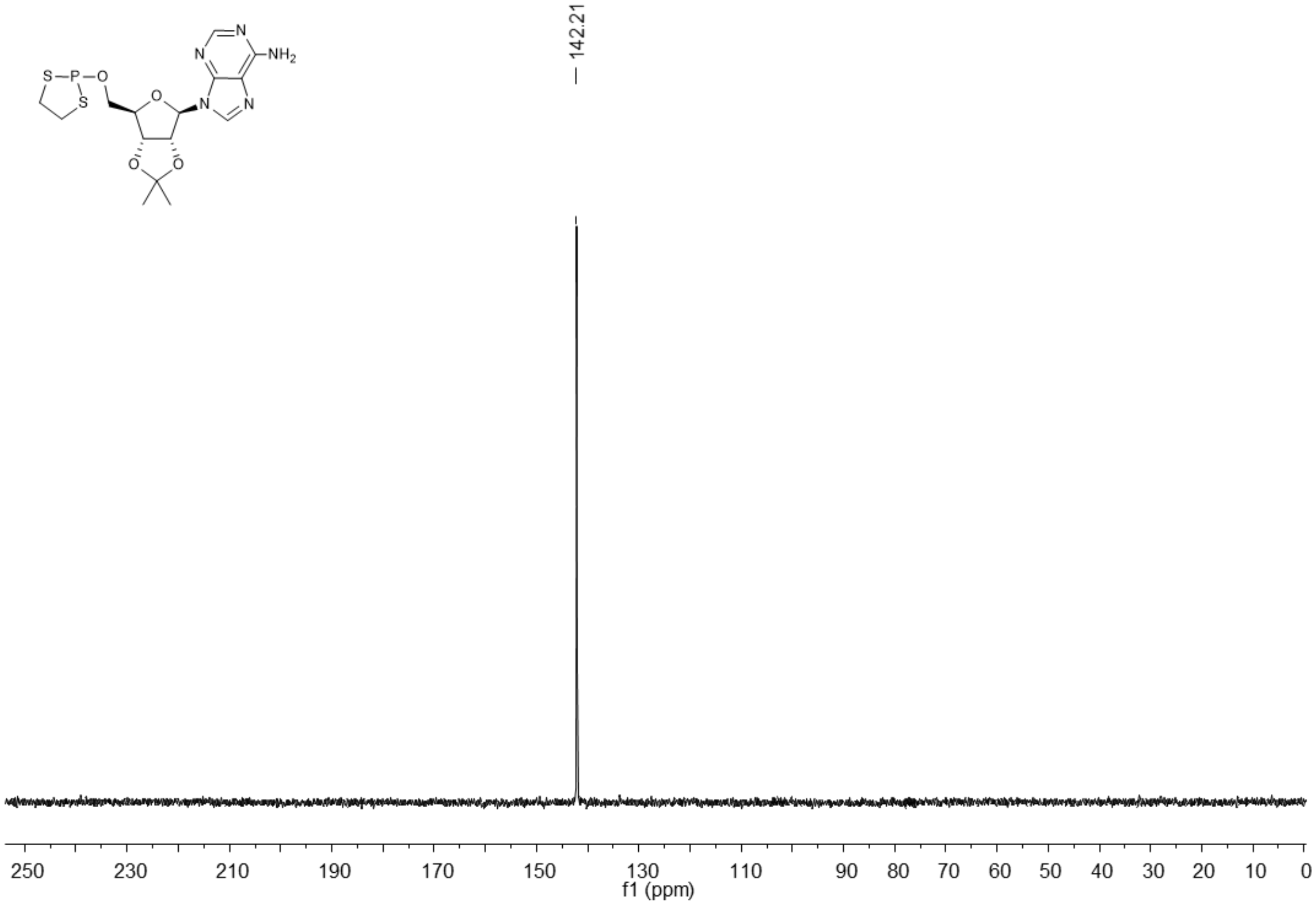

Figure A128. **^{31}P NMR (162 MHz, CDCl$_3$)** spectrum of **18a**.

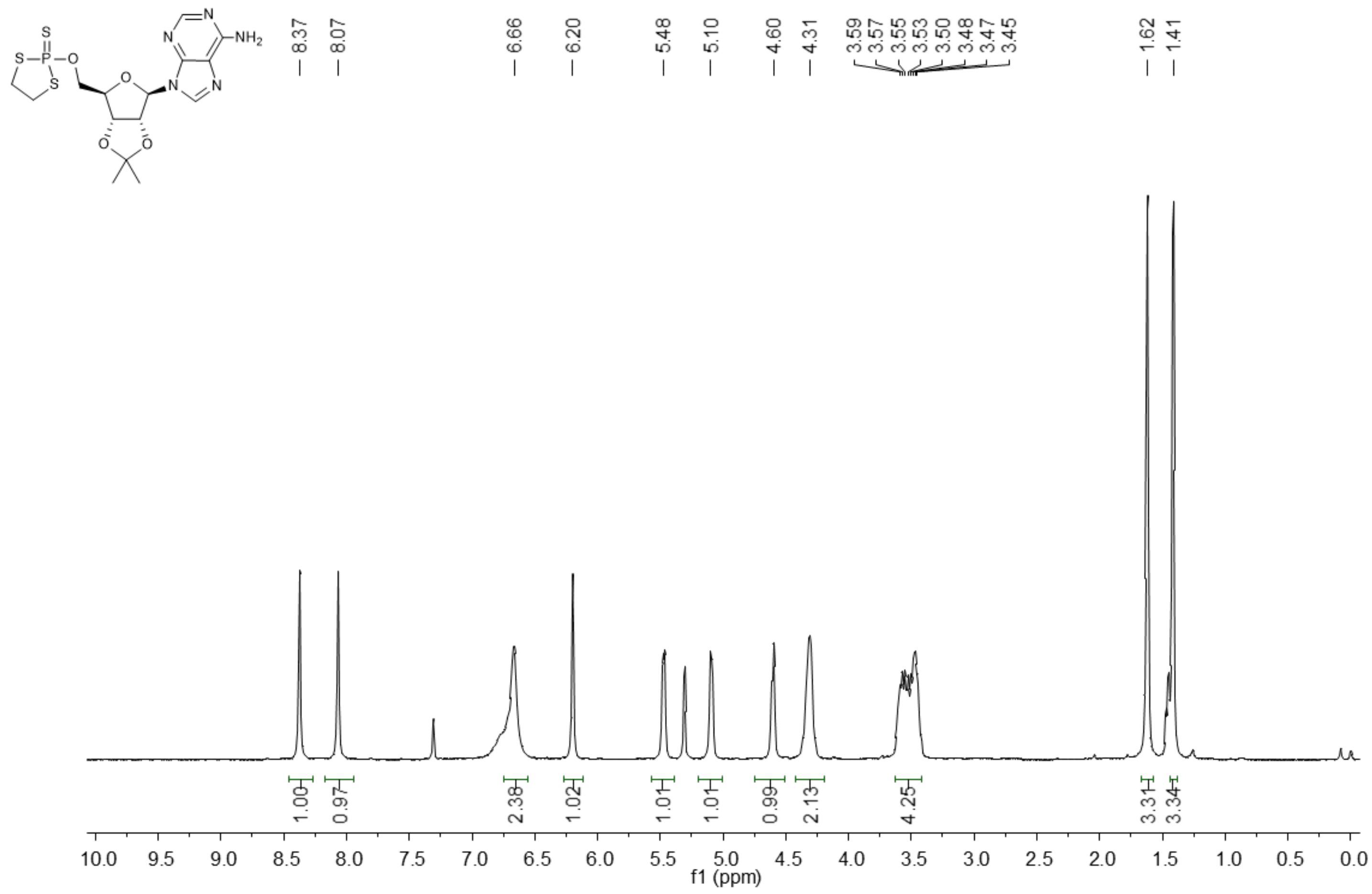

Figure A129. **^{1}H NMR (400 MHz, $CDCl_3$)** spectrum of **18b**.

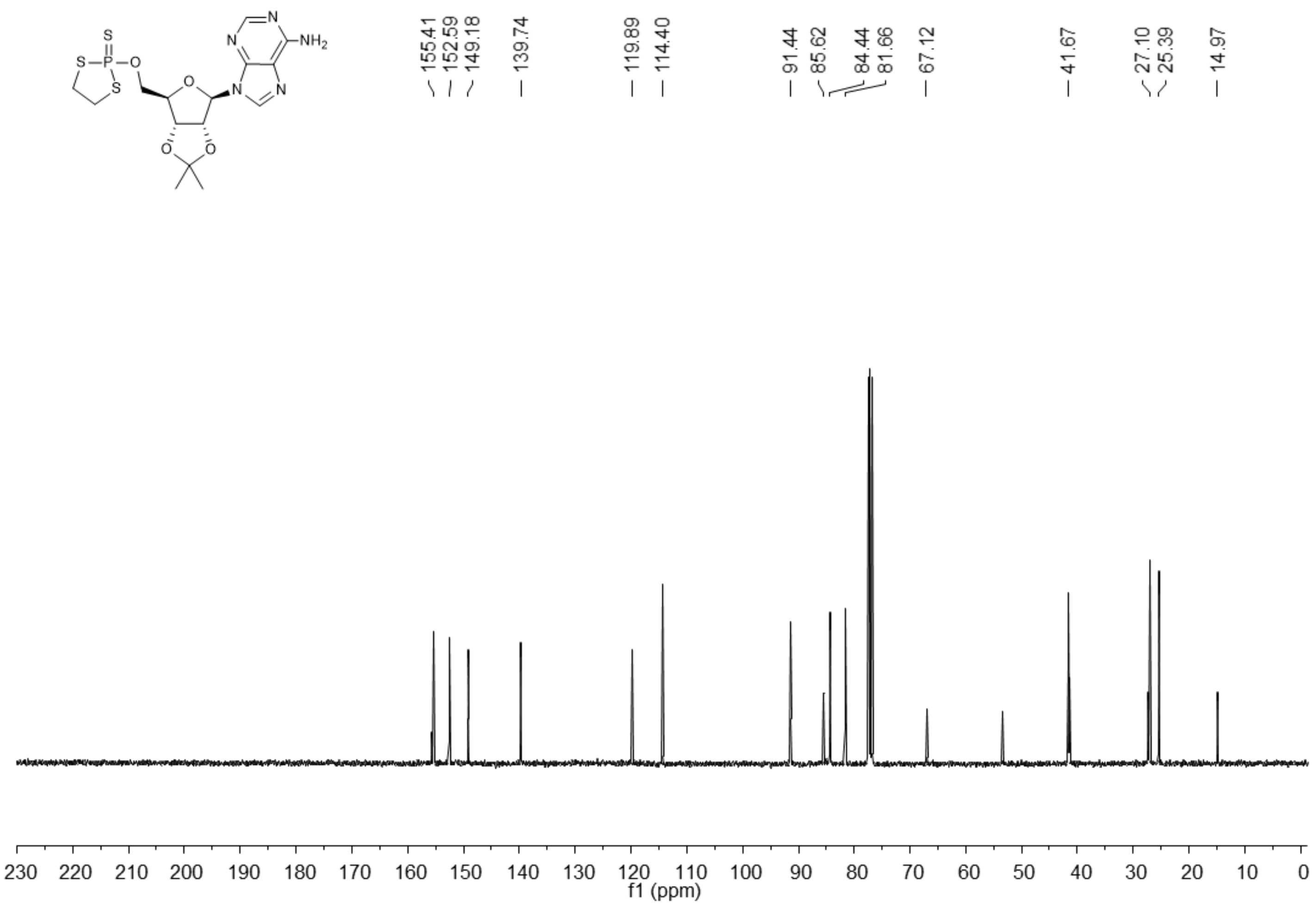

Figure A130. **^{13}C NMR (101 MHz, $CDCl_3$)** spectrum of **18b**.

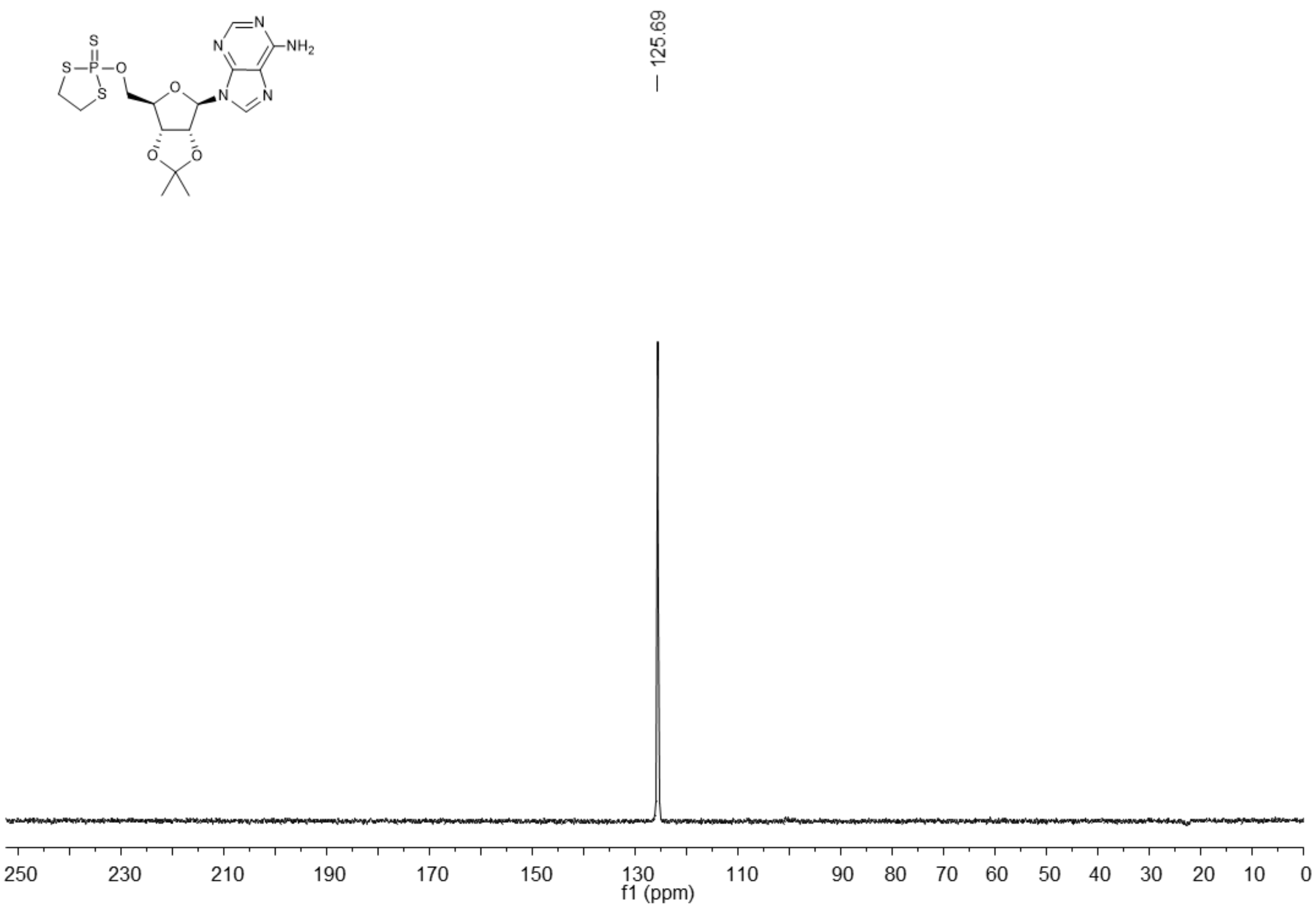

Figure A131. **^{31}P NMR (162 MHz, $CDCl_3$)** spectrum of **18b**.

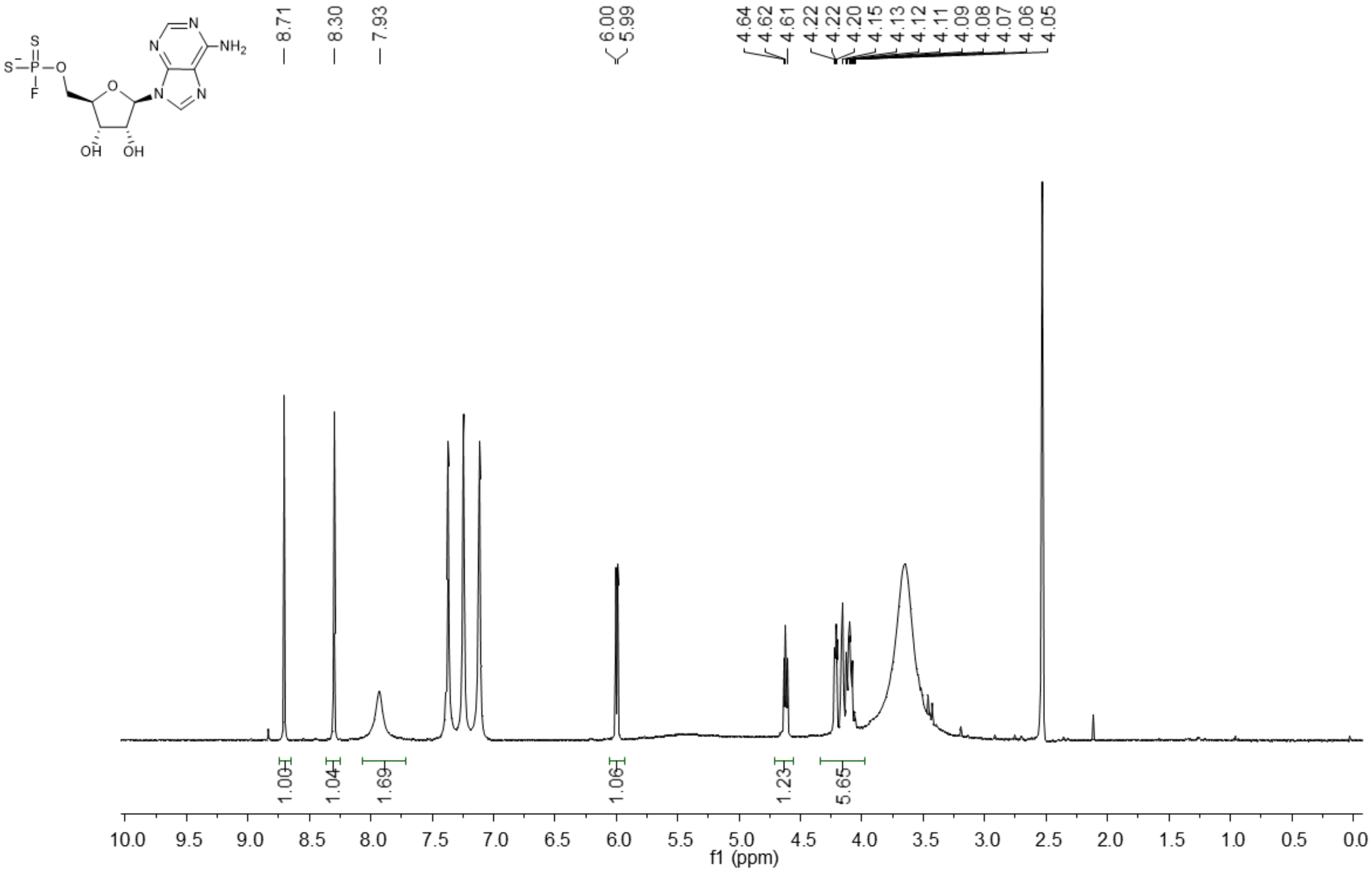

Figure A132. [1]**H NMR (400 MHz, DMSO-*d*$_6$)** spectrum of **18**.

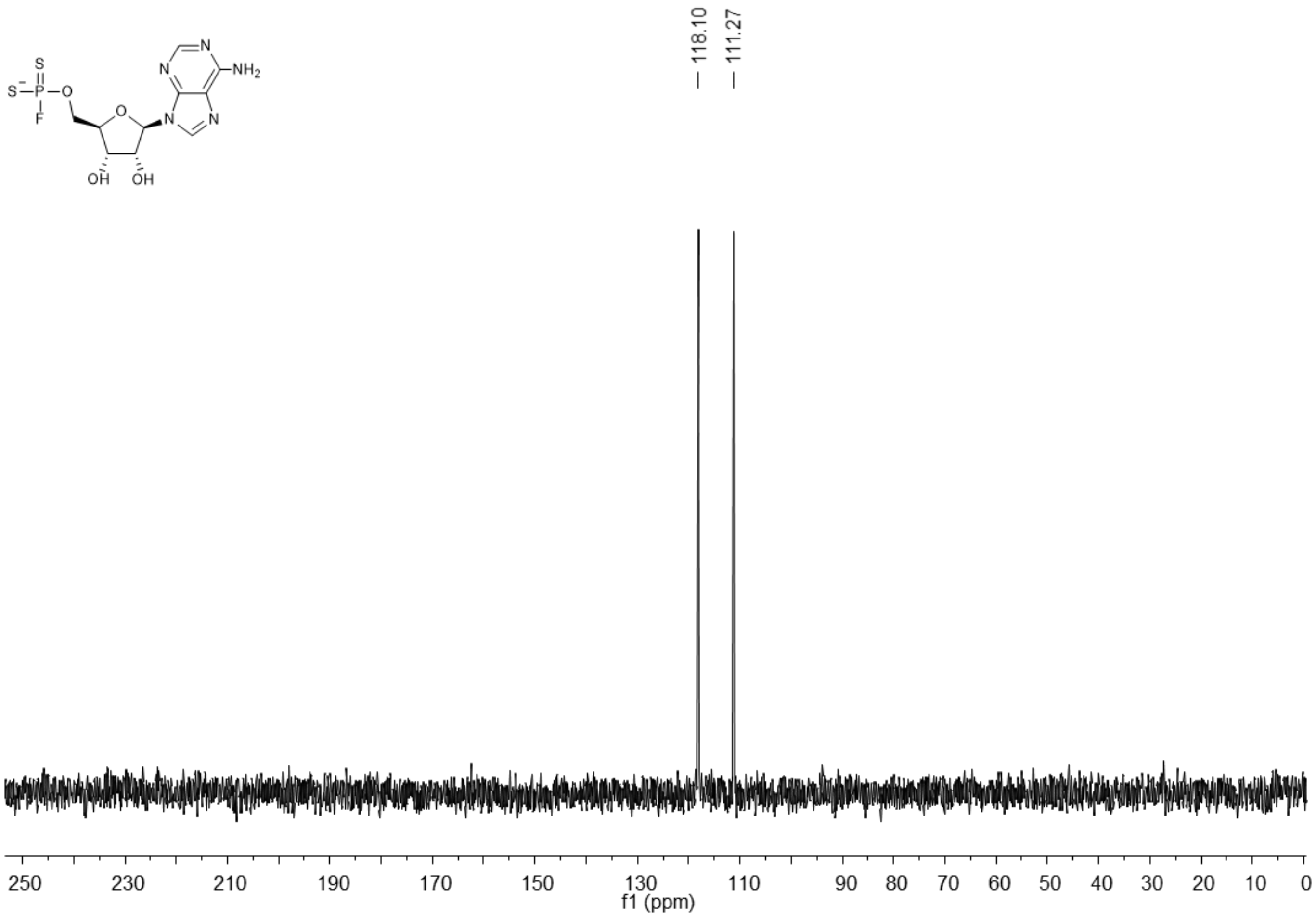

Figure A133. ^{19}**F NMR (376 MHz, DMSO-*d*$_6$)** spectrum of **18**.

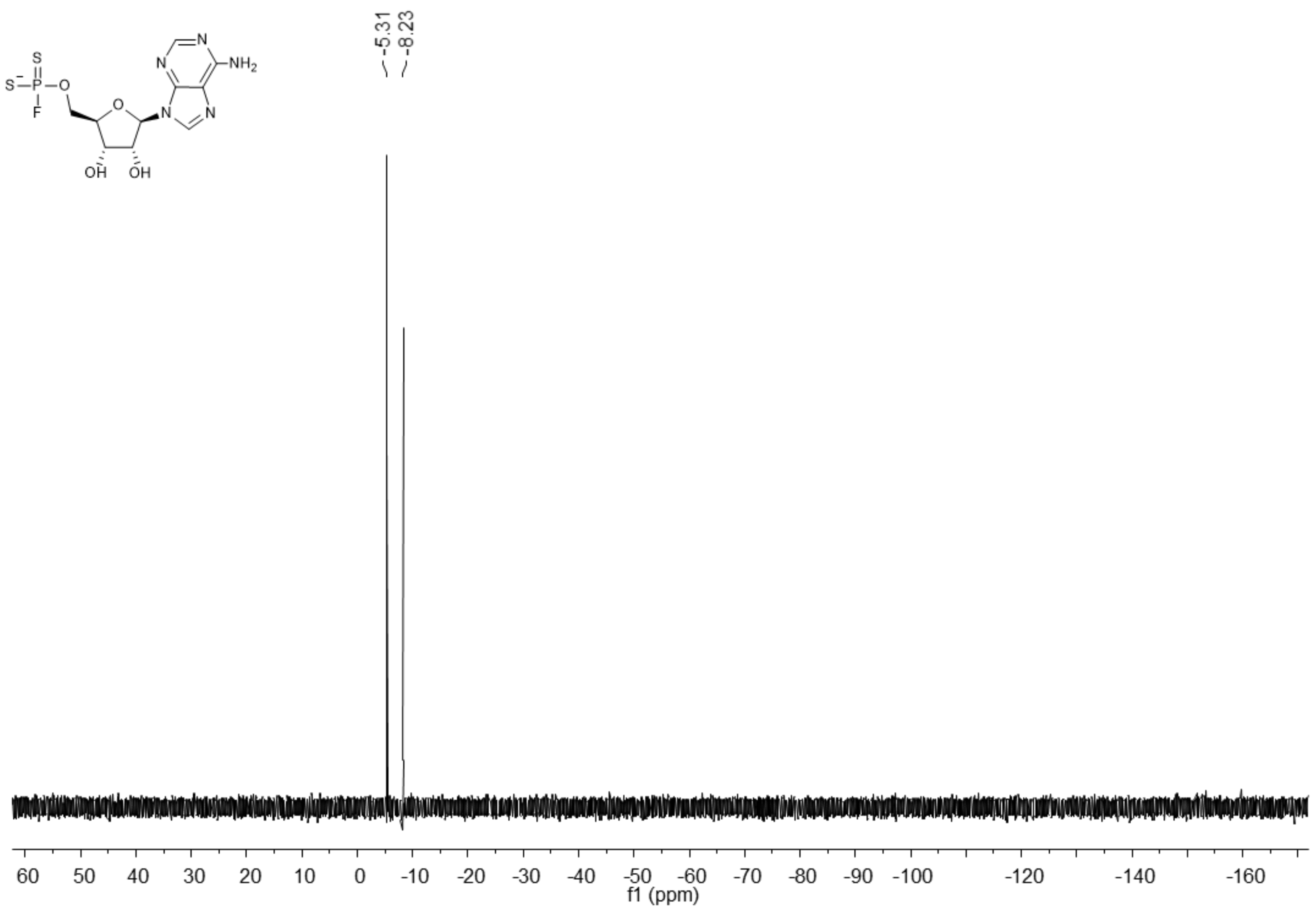

Figure A134. **^{31}P NMR (162 MHz, DMSO-*d₆*)** spectrum of **18**.
